# Benchmarking large language models on the United States medical licensing examination for clinical reasoning and medical licensing scenarios

**DOI:** 10.1038/s41598-025-31010-4

**Published:** 2025-12-03

**Authors:** Md Kamrul Siam, Angel Varela, Md Jobair Hossain Faruk, Jerry Q. Cheng, Huanying Gu, Abdullah Al Maruf, Zeyar Aung

**Affiliations:** 1 Computer Science, New York Institute of Technology, New York, USA; 2 Colegio Maria Cano I.E.D., Bogotá, Colombia; 3 Bangladesh University of Business and Technology, Dhaka, Bangladesh; 4 Electrical Engineering and Computer Science, Khalifa University, Abu Dhabi, UAE

**Keywords:** Large language models (LLMs), Clinical reasoning, USMLE, Medical licensing examination, Diagnostic decision support, Diseases, Health care, Health occupations, Medical research

## Abstract

Artificial intelligence (AI) is transforming healthcare by assisting with intricate clinical reasoning and diagnosis. Recent research demonstrates that large language models (LLMs), such as ChatGPT and DeepSeek, possess considerable potential in medical comprehension. This study meticulously evaluates the clinical reasoning capabilities of four advanced LLMs, including ChatGPT, DeepSeek, Grok, and Qwen, utilizing the United States Medical Licensing Examination (USMLE) as a standard benchmark. We assess 376 publicly accessible USMLE sample exam questions (Step 1, Step 2 CK, Step 3) from the most recent booklet released in July 2023. We analyze model performance across four question categories: text-only, text with image, text with mathematical reasoning, and integrated text-image-mathematical reasoning and measure model accuracy at three USMLE steps. Our findings show that DeepSeek and ChatGPT consistently outperform Grok and Qwen, with DeepSeek reaching 93% on Step 2 CK. Error analysis revealed that universal failures were rare ($\le$1.60%) and concentrated in multimodal and quantitative reasoning tasks, suggesting both ensemble potential and shared blind spots. Compared to the baseline ChatGPT-3.5 Turbo, newer models demonstrate substantial gains, though possible training-data exposure to USMLE content limits generalizability. Despite encouraging accuracy, models exhibited overconfidence and hallucinations, underscoring the need for human oversight. Limitations include reliance on sample questions, the small number of multimodal items, and lack of real-world datasets. Future work should expand benchmarks, integrate physician feedback, and improve reproducibility through shared prompts and configurations. Overall, these results highlight both the promise and the limitations of LLMs in medical testing: strong accuracy and complementarity, but persistent risks requiring innovation, benchmarking, and clinical oversight.

## Introduction

The swift progression of AI technology has significantly altered nearly every domain by providing novel solutions to complex problems^1,2^. Among these innovations, LLMs, a subclass of artificial intelligence trained on vast corpora of textual data to comprehend, generate, and reason about human language, are at the forefront^3,4^. This deep neural network-based model has demonstrated exceptional proficiency in natural language processing tasks that can generate automated text generation and complex semantic reasoning such as programming^5^. Notable examples of LLM-based applications include ChatGPT (OpenAI, https://openai.com), DeepSeek (DeepSeek, https://www.deepseek.com), Grok (xAI, https://x.ai), and Qwen (Alibaba Cloud, https://www.alibabacloud.com). These models empower stakeholders across several sectors to enhance knowledge acquisition, optimize sophisticated decision-making processes, and elevate overall productivity and efficacy^6^. In healthcare, the incorporation of AI promises to significantly improve medical education, clinical decision-making, diagnostic precision, and overall patient care outcomes^7–9^. Although AI models may be beneficial, it is very cruicial to rigorously assess them inside actual healthcare environments to determine their efficacy and safety for patients^10^.

The USMLE is a reliable and widely acknowledged indicator of clinical understanding as well as reasoning^11^. The test assesses medical students and resident doctors with a three-tier evaluation encompassing the fundamental biomedical sciences, clinical knowledge (CK), and patient-centered clinical applications^12^. The USMLE provides a systematic framework and rigorous assessment standards. Its focus on intricate medical reasoning renders it a potential and optimum benchmark for evaluating the clinical significance and effectiveness of AI-driven language models^13^. Recent research has shown that earlier generations of LLM, notably ChatGPT, possess the ability to achieve near-pass scores on USMLE-style assessments without explicit medical domain training^11,14^. Nonetheless, recent models like DeepSeek, Qwen, and Grok offer an opportunity to methodically assess and contrast their superior reasoning and diagnostic skills with accepted standards. Moreover, the publication of a revised USMLE question set in June 2023, along with the advent of more advanced LLMs, requires a reassessment. As of yet, no research has thoroughly evaluated model performance across all four question categories–text-only, text with picture, text with mathematical reasoning, and integrated text-image-mathematical reasoning–while concurrently assessing correctness at each of the three sequential USMLE steps. Analyzing the model’s strengths, errors, and consistency elucidates its practical applicability for clinical reasoning and decision-making. These insights will facilitate the evaluation of AI’s efficacy in integrating into medical workflows and enhancing diagnostic reliability^15^.

This study evaluates four advanced LLMs (ChatGPT, DeepSeek, Grok, and Qwen) in terms of clinical reasoning by analyzing 376 USMLE questions from Steps 1, 2 CK, and 3. We assess their accuracy, analyze error patterns, and explore the potential of hybrid models to enhance medical education and patient care, while providing pragmatic insights for the integration of AI in healthcare. Furthermore, we evaluated the widely-used predecessor, ChatGPT-3.5 Turbo, to establish a performance baseline. While its results are presented, we have intentionally excluded it from the primary comparative analysis of newer models due to its recognized architectural and lack of specialized medical tuning compared to the newer models in this study. Instead, its performance is utilized in the Discussion section as a historical benchmark to contextualize advancements and verify our hypothesis that more recent and specialized models demonstrate substantially higher accuracy and greater alignment with medical reasoning. This approach highlights the rapid evolution of LLM capabilities in clinical contexts.

## Methodology

### Data collection and preparation

**Fig. 1:**
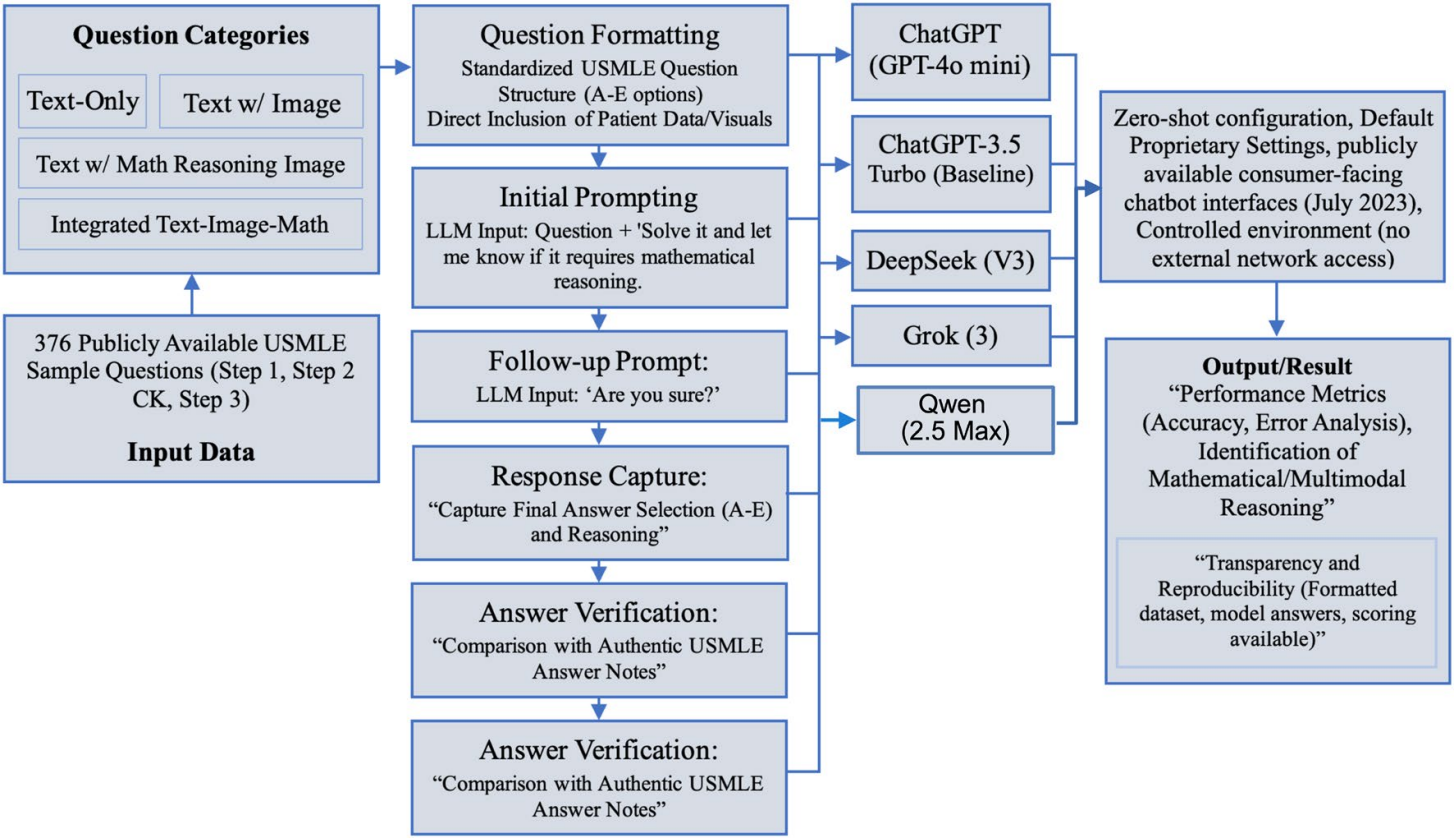
USMLE LLM evaluation workflow: 376 USMLE sample questions (text-only, image-based, math-based, and integrated) are standardized and presented to large language models under a zero-shot setting. Responses are compared with official USMLE keys, and outputs are analyzed for accuracy, error types, and reasoning patterns with emphasis on transparency and reproducibility.

In this study, we utilize a comprehensive dataset comprising 376 publicly available USMLE sample test questions (for Step 1, Step 2 CK, and Step 3), which are available at the USMLE official website (https://www.usmle.org/exam-resources). The dataset spans all three sequential steps of the USMLE, illustrated in Fig. 1:

**Step 1** targets the foundational biomedical sciences by diving deep into major subjects like anatomy, physiology, biochemistry, pathology, pharmacology, microbiology, genetics, immunology, and nutrition. It puts a magical spotlight on the learning and the application of scientific concepts in medical practice (USMLE Step 1 Content Outline).

**Step 2** clinical knowledge (CK) shifts to the application of clinical science across major medical disciplines, such as internal medicine, pediatrics, obstetrics and gynecology, surgery, psychiatry, and public health. It assesses the capability of a junior doctor in the areas of diagnosis, treatment, illness prevention, and collaboration among health professionals to manage multiple clinical situations (USMLE Step 2 CK Content Outline).

In **Step 3** the last and the most challenging phase, it is judged whether the candidates can apply their biomedical and clinical science knowledge to the intervention of the patients, all by themselves. It has sophisticated, multi-step patient care scenarios that mirror the real-life responsibility of a clinician, e.g., prolonged patient management and treatment carried on in various care settings over time (USMLE Step 3 Content Outline).

The systematic and thorough steps transform the USMLE into the best option for assessing the construction, decision-making correctness, and real-world implementation of LLMs in the medical field. We maintained the same set of questions for the purpose of ensuring both standardization and fairness in model evaluations without leaving out any of the questions from the USMLE sample test questions. In this process, the resulting dataset has both text-based, basic mathematical reasoning based and image-based multiple-choice questions which are suitable for evaluating medical language and image-based reasoning. The annotation of each question with the corresponding USMLE step and clinical domain enables a thorough and structured analysis of performance metrics in entirely different medical knowledge categories.

While the original USMLE sample questions are copyrighted material accessible from the official website, our complete analysis dataset is provided in the repository cited in the Data Availability section. This dataset includes the formatted questions, each model’s final answer selection (A-E), and our complete scoring to ensure transparency and allow for the replication of our analysis.

### Model evaluation protocol

The primary models evaluated in this analysis were: ChatGPT (GPT-4o mini), DeepSeek (V3), Grok (3), and Qwen (Qwen2.5-Max). To provide a historical baseline and contextualize the performance of these newer models, we also included the widely-used predecessor, ChatGPT-3.5 Turbo. Other prominent domain-specific models, such as Med-PaLM and BioGPT, were not included in our direct evaluation as they are not publicly available for reproducible benchmarking^16^.

The mode of GPT-4o mini is real-time interaction, one of the most effective models for combining performance with efficiency. Similarly, the DeepSeek V3 model is an open-source large language model that has been specifically designed for comprehensive context understanding and variable input. Along the same lines, Grok 3 is a model dedicated solely to real-time contextual reasoning, while Qwen2.5-Max is a multilingual model developed specifically to assist in text, code, and mathematical reasoning in addition to its initial visual capabilities. To ensure fairness across platforms, we provided the exact images of all type of questions for models like ChatGPT, which cannot accept images directly. This adjustment allowed for consistent and fair processing of each model’s responses.

### Prompting strategy and question presentation

To ensure consistency and transparency in evaluating each LLM, we adopted a standardized prompting framework designed to simulate realistic test-taking conditions. Notably, the score on USMLE Step 1 is only reported as a pass or fail for tests conducted on or after January 26, 2022^17^. We formatted each USMLE-style question to preserve its original structure, including the main question text and multiple-choice options (typically five choices labeled A through E). For the questions including patient data or visual materials, such as vital signs or radiographic or pathological images, the image or patient information is directly included to preserve semantic meaning for the model.

Given that some models, such as ChatGPT’s text-only interface, cannot process image-based inputs directly, we ensured that all models received the same question context by providing screenshots of the images. This ensured fairness and consistent access to the visual information necessary to answer these questions.

To assess the models’ ability to identify reasoning complexity, we append an additional prompt following each question: *“Solve it and let me know if it requires mathematical reasoning.”* This supplementary instruction serves two purposes: first, to evaluate the model’s awareness of the cognitive and quantitative demands of the question; and second, to facilitate classification of questions involving mathematical reasoning (TM) or multimodal^18^ integration (ITM/TI).

We also asked follow-up prompts such as *“Are you sure?”* immediately after the model’s initial response. This follow-up is used to evaluate the model’s stability, confidence, and potential for self-revision. The final recorded answer is based on the model’s response following this clarification prompt. This approach allows us to assess both first-pass and reflective reasoning, offering deeper insight into model reliability under simulated test-taking conditions. All prompts are manually reviewed by six human evaluators for fairness, completeness, and consistency across the four evaluated models.To ensure fairness across platforms, we provided the exact images and text of the questions in the form of images for models like ChatGPT, which cannot accept images directly. This adjustment allowed for consistent and fair processing of each model’s responses.

## Performance metrics and error analysis

The evaluation methodology incorporates several comprehensive performance metrics designed to rigorously assess the clinical reasoning capabilities and diagnostic accuracy of each LLM. The following metrics are systematically applied:

### Human evaluation and error categorization protocol

To ensure a robust and clinically-grounded qualitative analysis of model errors, a structured human evaluation process was implemented. To provide real-world grounding for our findings, our panel included four healthcare domain researchers, each with over three years of postgraduate research experience in health informatics, biomedical science, or public health. These four were designated as health domain experts for this analysis, and their role was to ensure that the interpretation of model errors was clinically relevant and sound. The remaining three researchers provided expertise in computer science and AI evaluation.

The error categorization was performed as follows: each of the seven researchers independently evaluated and classified all incorrect model responses into one of three predefined categories (Conceptual Misunderstanding, Misinterpretation, or Reasoning Error). After the independent review, the entire panel convened to discuss discrepancies. Any conflicts in categorization were resolved through a moderated consensus discussion, with the final classification for each error being determined by a majority vote. This structured, multi-expert process ensures a high degree of reliability and clinical validity in our qualitative analysis.

To quantitatively measure the reliability of this classification process, we calculated Inter-Rater Reliability (IRR) using Fleiss’ Kappa ($\kappa$). This statistic is suitable for measuring agreement among multiple raters on categorical data. The resulting Fleiss’ Kappa value of $\kappa$ = 0.85 indicates an “almost perfect” level of agreement among the health domain research experts, confirming that the error taxonomy was clear, objective, and applied with a high degree of consistency^19^. This high level of agreement validates the reliability of the subsequent qualitative error analysis.

### Data handling and question categorization

To assess the adaptability of the models, we classified all 376 test items into four types based on the reasoning required:

- **Text (T):** Questions containing only textual information.
- **Text + image (TI):** Questions requiring interpretation of visual data (e.g., radiographs, ECGs) alongside text.
- **Text + mathematical reasoning (TM):** Text-based questions requiring quantitative analysis.
- **Image + text + mathematical reasoning (ITM):** Multimodal questions requiring integration of all three data types.

While the USMLE is primarily a clinical reasoning examination, a crucial component of modern medical practice involves quantitative literacy. Clinicians must constantly interpret lab value ranges, calculate dosages, and understand statistical concepts presented in patient data. Although these calculations are often simple, an error can have severe clinical consequences. Furthermore, since state-of-the-art LLMs are often trained on advanced mathematical datasets, evaluating their reliability on fundamental clinical arithmetic is essential to identify potential “over-thinking” or brittleness. Therefore, our inclusion of the TM and ITM categories is a deliberate stress test of a critical, albeit small, component of the models’ potential utility as a clinical support tool.

### Human performance benchmark and passing scores

We used the human benchmark references from the official USMLE website to contextualize the performance of large language models in context. These official reports represent the empirical performance baselines for first-time examinees at LCME-accredited U.S. and Canadian medical schools. For the Step 1 examination, we used historical passing scores before the transition to pass/fail scoring in January 2022–specifically, 194 in both 2019 and 2020, and 196 in 2021. Accordingly, we estimate the human average is about 78%. The pass score for Step 2 CK was 209 in 2021, and it was increased to 214 in 2022, while it remained constant till 2023; therefore, the human examinees’ performance of around 82% is estimated on average during the 2021–2024 academic years. For Step 3, the pass score was 198 in both 2022 and 2023, climbing to 200 in 2024, and estimating an average human performance of 76%. These are the true benchmarks to measure whether LLMs performance in the tests is at, above or below the standard human’s typical accuracy levels. The Step 1 report last updated on May 19, 2022 (Step 1 Report); the Step 2 CK and Step 3 reports were last updated on April 24, 2025 (Step 2 & 3 Report). All reports were last accessed on May 12, 2025.

### Accuracy rate

Accuracy rate (*A*) quantifies each model’s performance by determining the percentage of correctly answered questions. It is mathematically defined as:

1 $$\begin{aligned} A = \frac{N_{\text {correct}}}{N_{\text {total}}} \times 100\% \end{aligned}$$

where $N_{\text {correct}}$ represents the number of correctly answered questions by a specific model, and $N_{\text {total}}$ denotes the total number of questions evaluated.

### Error categorization

Errors identified during model evaluation are categorized into three distinct types, each representing different aspects of potential failure in clinical reasoning:

- Conceptual misunderstandings: Errors resulting from fundamental inaccuracies or incomplete knowledge of underlying medical concepts.
- Misinterpretations: Mistakes arising from an incorrect understanding of the question’s intent, contextual nuances, or clinical setting described.
- Reasoning errors: Logical inconsistencies, flawed deductions, or incorrect application of medical knowledge during the reasoning process.

This classification framework facilitates in-depth analysis and identification of each model’s weaknesses, enabling targeted improvements.

### Model synergy analysis

To assess the complementary potential of hybrid model deployments, we conduct a synergy analysis, identifying cases where one model answers correctly while another model does not. This metric is quantified as follows:

2 $$\begin{aligned} S_{ij} = \frac{N_{\text {correct}(i), \text {incorrect}(j)}}{N_{\text {total}}} \times 100\% \end{aligned}$$

Here, $S_{ij}$ denotes the synergy score between model *i* and model *j*, with $N_{\text {correct}(i), \text {incorrect}(j)}$ representing the number of questions correctly answered by model *i* but incorrectly by model *j*.

## Statistical analysis

We employ robust statistical methods to rigorously evaluate and validate the performance differences observed among the models. The following analyses are conducted:

### Descriptive statistics

Mean accuracy and standard deviations are calculated for each model across each of the USMLE examination steps. These statistics offer insights into the consistency and reliability of each model’s performance.

### Tests of significance

To determine if the observed differences in accuracy between models were statistically significant, we performed pairwise Chi-square ($\chi ^2$) tests on the raw counts of correct and incorrect answers for each model. This was done for the entire question set (N=376) and within each of the four question categories. The null hypothesis was that there is no significant difference in the proportion of correct answers between any two models. A p-value of < 0.05 was considered statistically significant.

### Inter-rater reliability

Agreement among the multiple raters for error categorization was quantified using Fleiss’ Kappa ($\kappa$)^20^. The statistic is calculated as follows. First, for each item (question), the proportion of agreeing rater pairs ($P_j$) is computed:

3 $$\begin{aligned} P_j = \frac{1}{n(n-1)} \sum _{i=1}^{k} n_{ji}(n_{ji} - 1) \end{aligned}$$

where *n* is the number of raters, *k* is the number of categories, and $n_{ji}$ is the number of raters who assigned category *i* to item *j*. The mean observed agreement across all *N* items is then:

4 $$\begin{aligned} \bar{P} = \frac{1}{N} \sum _{j=1}^{N} P_j \end{aligned}$$

The agreement expected by chance is calculated from the overall proportion of assignments to each category ($p_i$):

5 $$\begin{aligned} p_i = \frac{1}{N \cdot n} \sum _{j=1}^{N} n_{ji}, \quad \text {and} \quad \bar{P}_e = \sum _{i=1}^{k} p_i^2 \end{aligned}$$

Finally, Fleiss’ Kappa is:

6 $$\begin{aligned} \kappa = \frac{\bar{P} - \bar{P}_e}{1 - \bar{P}_e} \end{aligned}$$

The value of $\kappa$ indicates agreement beyond chance and confirm the high reliability of the applied error taxonomy.

## Results and findings

### Performance analysis

**Fig. 2:**
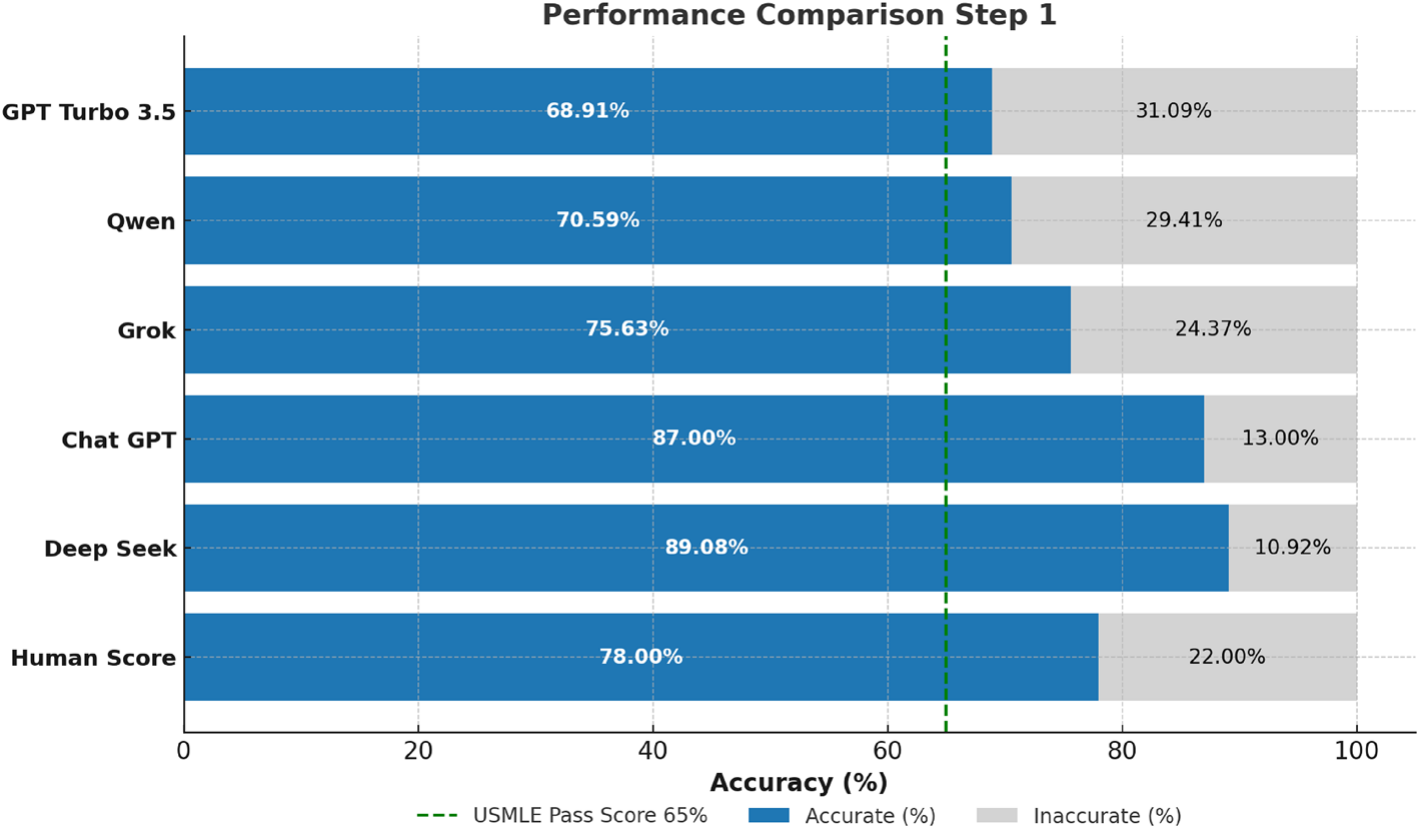
Performance comparison of four prominent LLMs and human benchmark scores on USMLE Step 1 (green dashed line marks the mean passing threshold for the 2019–2021 academic years).

**Fig. 3:**
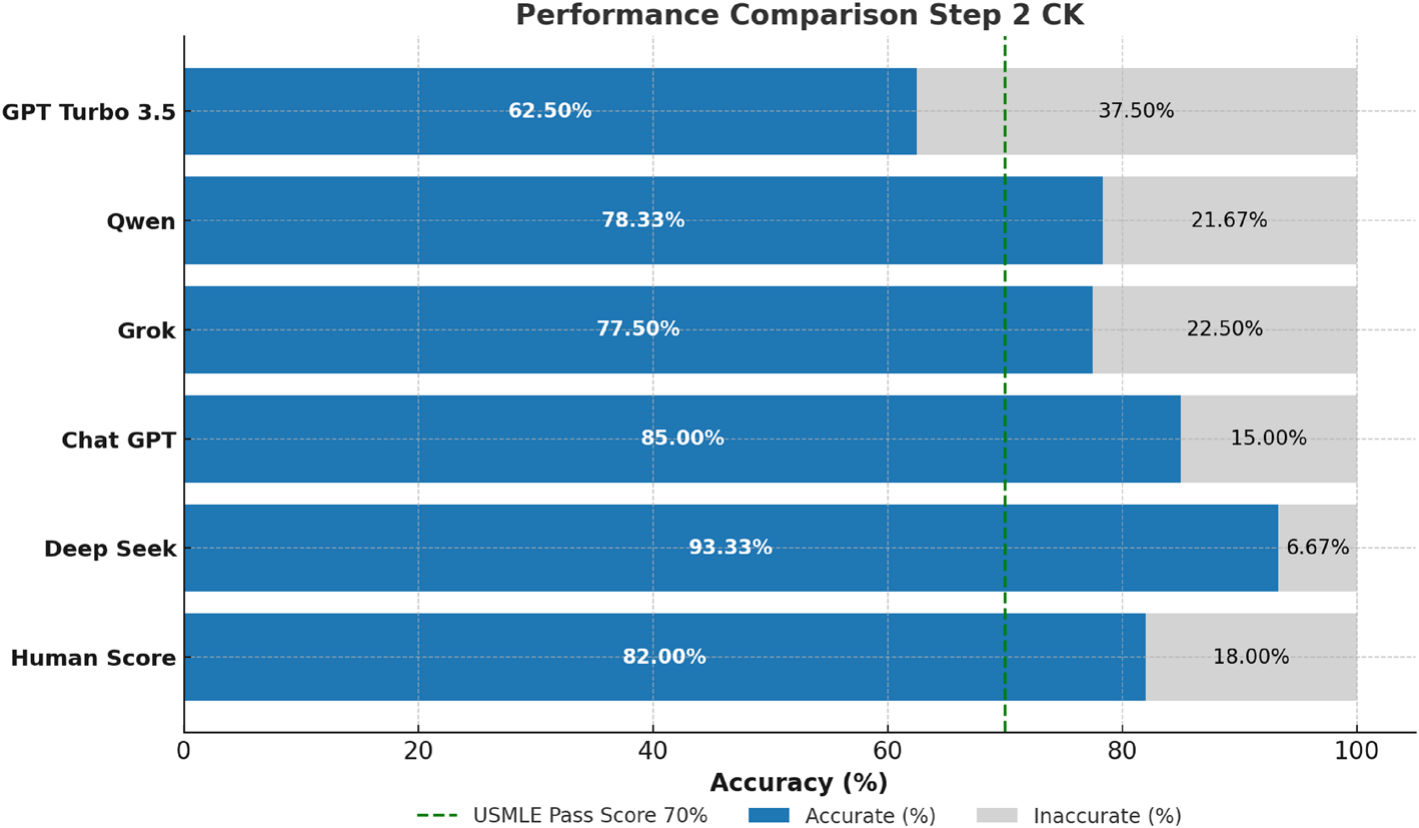
Performance comparison of four prominent LLMs and human benchmark scores on USMLE Step 2 CK (green dashed line marks the mean passing threshold for the 2022–2024 academic years).

**Fig. 4:**
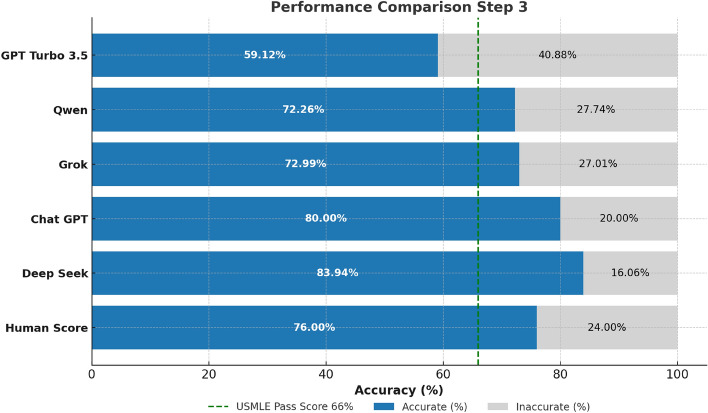
Comparative analysis of the performance of four LLMs and human benchmark scores on USMLE Step 3 examination (green dashed line marks the mean passing threshold for the 2022–2024 academic years).

**Fig. 5:**
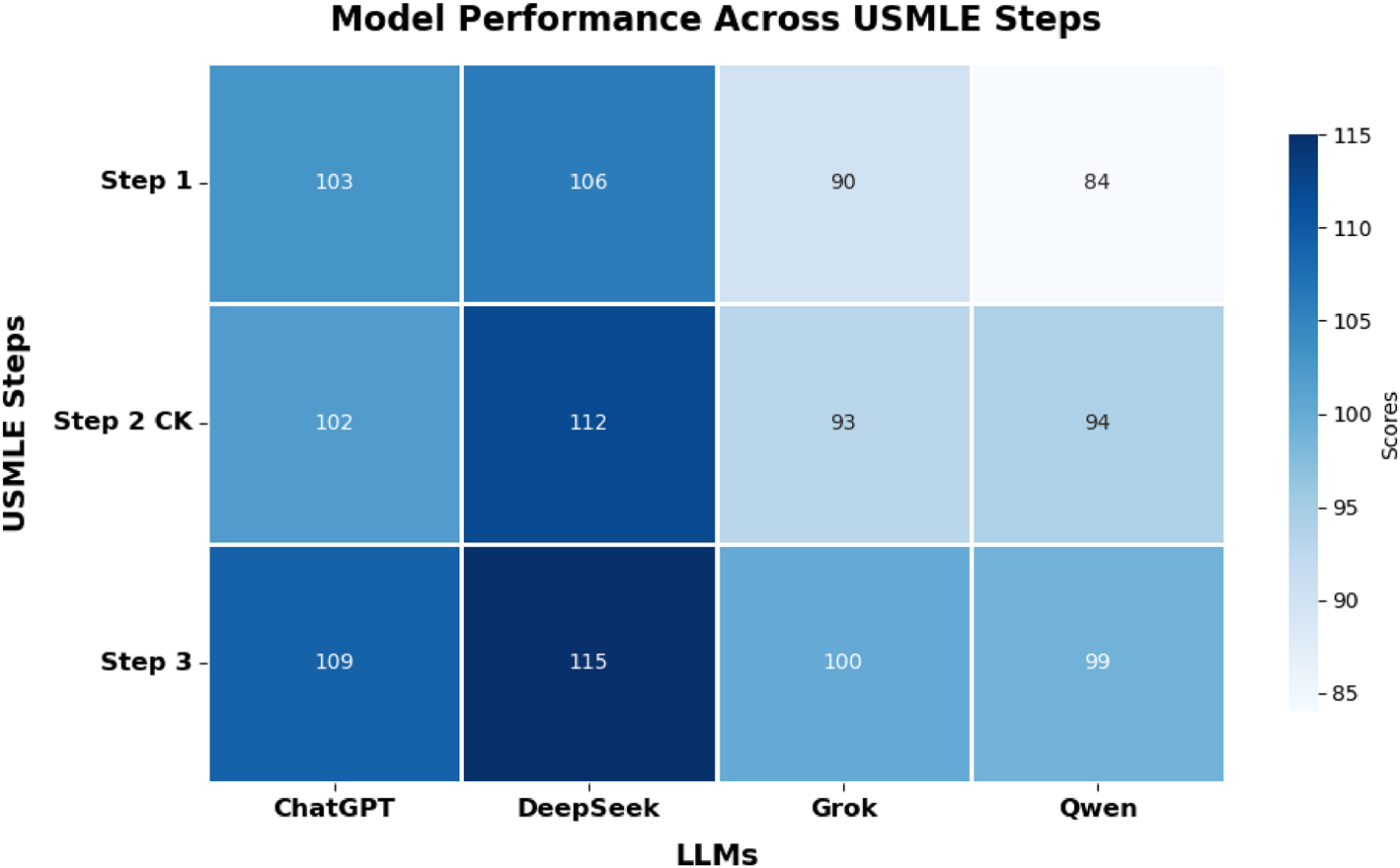
Heatmap depicting the overall accurate responses by ChatGPT, DeepSeek, Grok, and Qwen for USMLE Step 1 (n=119), Step 2 CK (n=120), and Step 3 (n=139). The darker shades indicate a greater quantity of accurate replies, underscoring DeepSeek’s reliable performance throughout every step.

The performance evaluation of all four LLMs across all three stages of the USMLE examination with the models being confident in their answers reveals distinct and consistent trends (Figs. 2, 3,4 and 5). Across all steps, DeepSeek consistently achieves superior performance in comparison with other LLMs. Specifically, in Step 1, DeepSeek achieves the highest accuracy of 89%, closely followed by ChatGPT at 87%. Grok and Qwen exhibit significantly lower accuracies of 76% and 71%, respectively. In Step 2 CK, DeepSeek’s performance peaks at 93% accuracy, displaying excellent clinical reasoning capabilities. On the contrary, ChatGPT recorded a solid accuracy of 85%, while Grok (77%) and Qwen (78%) performed lower. In Step 3, DeepSeek maintains its lead with 84% accuracy, outperforming ChatGPT (80%), Grok (73%), and Qwen (72%) (Figs. 3 and 4).

When aggregating the total correct responses across all three USMLE steps, DeepSeek consistently demonstrates superior overall performance. Figure 5 illustrates the heatmap analysis, which visually affirms DeepSeek’s strong and constant accuracy throughout all examination phases, followed by ChatGPT, Grok, and Qwen in descending order.

### Inter-model agreement

**Fig. 6:**
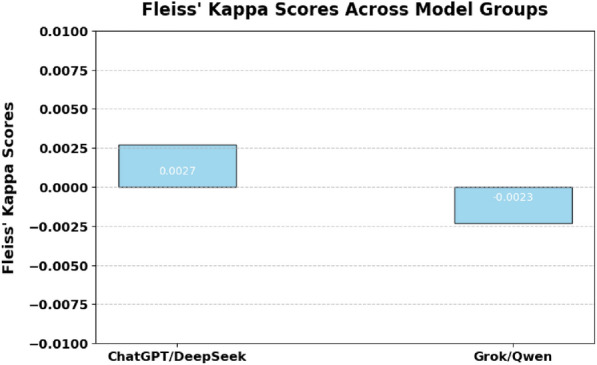
Fleiss’ Kappa scores indicating the degree of inter-model agreement between ChatGPT/DeepSeek and Grok/Qwen model pairs. Positive values suggest slight agreement, while negative values indicate disagreement among predictions.

To evaluate the agreement among the models in selecting incorrect answers, Fleiss’ Kappa scores are calculated for two distinct model pairs: ChatGPT/DeepSeek and Grok/Qwen. Fleiss’ Kappa scores indicate minimal inter-model agreement, reflecting independent reasoning processes rather than consensus-based correctness. Specifically, the pair ChatGPT/DeepSeek achieves a slight positive Fleiss’ Kappa of 0.0027, suggesting minor agreement in their correct answers. In contrast, Grok/Qwen has a marginally negative score of −0.0023, indicating slight disagreement and differing reasoning methodologies between the two models, as demonstrated in Fig. 6.

**Fig. 7:**
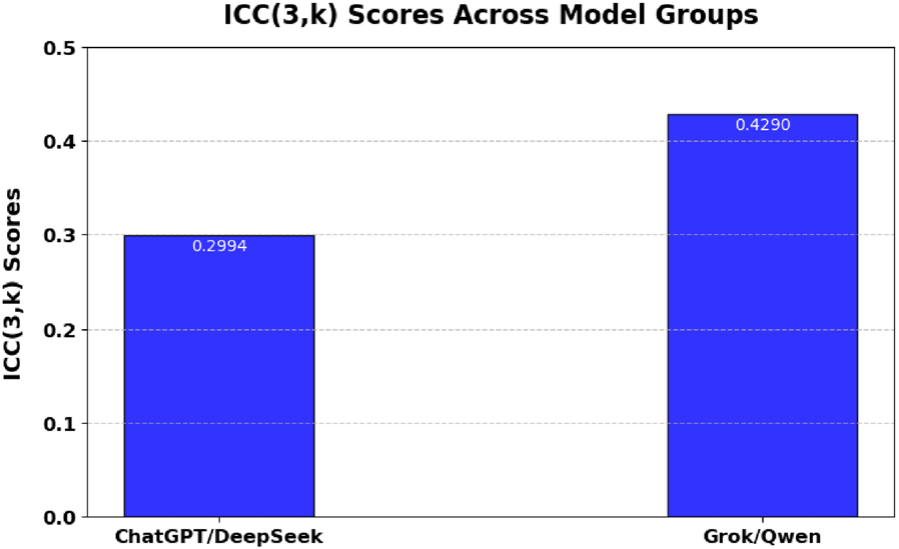
Intraclass Correlation Coefficient (ICC, type 3, k) scores assessing the consistency and reliability of responses within ChatGPT/DeepSeek and Grok/Qwen model groups. Higher values reflect stronger internal agreement.

These findings suggest that combining models such as ChatGPT and DeepSeek could potentially leverage complementary reasoning strategies to enhance overall diagnostic accuracy.

### Consistency and reliability

The Intraclass Correlation Coefficient (ICC, type 3,k) assesses the consistency and reliability of model predictions within paired groups. ICC scores indicate moderate reliability and consistency within each evaluated pair. Grok/Qwen demonstrates higher consistency (ICC=0.4290), suggesting relatively stable prediction patterns across various scenarios, despite lower overall accuracy. On the contrary, ChatGPT/DeepSeek has a reduced ICC value of 0.2994, indicating higher variability in predictions despite their elevated individual accuracies observed in Fig. 7. This variation indicates these two models employ diverse reasoning strategies, potentially beneficial for hybrid or ensemble modeling approaches to improve clinical decision support.

Overall, these results underscore the potential effectiveness of large language models in medical education and clinical decision-making settings, particularly highlighting the superior accuracy and clinical reasoning capabilities of DeepSeek and ChatGPT. The observed variations in inter-model agreement and prediction consistency suggest further exploration into hybrid AI models could yield significant benefits in practical healthcare applications.

### Statistical significance of overall performance

To provide statistical rigor to our comparisons, Chi-square tests were performed on the overall performance of the models across all 376 questions. As shown in Table 1, the performance difference between the two top-performing models, ChatGPT and DeepSeek, was not statistically significant (*p* = 0.140). Similarly, the difference between Grok and Qwen was not significant (*p* = 0.862). However, the performance gap between the leading model (DeepSeek) and the baseline model (ChatGPT-3.5 Turbo) was highly significant (*p* < 0.0001), confirming substantial advancements in newer models.

**Table 1:** Results of Chi-square tests for pairwise model performance across all 376 USMLE questions.

Model comparison (overall)	$\chi ^2$ value	*p*-value	Significance
ChatGPT vs. DeepSeek	2.18	0.140	Not significant
Grok vs. Qwen	0.03	0.862	Not significant
DeepSeek vs. ChatGPT-3.5	35.12	< 0.0001	Highly significant

### Data handling capabilities

A breakdown of model performance for each USMLE step is presented in Table 2 (Step 1) , Table 3 (Step 2 CK) , and Table 4 (Step 3). Each table includes statistical comparisons against the top-performing model, DeepSeek.

In **Step 1**, DeepSeek and ChatGPT show outstanding, and statistically indistinguishable, performance on text-only questions. Performance drops across all models for multimodal (TI, TM, and ITM) questions, indicating challenges in handling image-based and quantitative clinical reasoning. While Grok achieved a perfect score on the ITM subset of Step 1 (only 4 questions), we discuss this anomaly further as it could indicate a limited statistical significance due to the small sample size. This result should be interpreted with caution due to the minimal number of ITM questions.

**Table 2:** Performance results in USMLE Step 1. Asterisk (*) denotes a statistically significant difference (*p* < 0.05) compared to DeepSeek’s performance in that category.

Step 1	ChatGPT	DeepSeek	Grok	Qwen
Text (n = 90)	92%	94%	79%*	74%*
TI (n = 21)	66%	76%	57%	52%
TM (n = 4)	75%	50%	75%	75%
ITM (n = 4)	75%	75%	100%	75%

In **Step 2 CK**, DeepSeek achieves a near-perfect 98% accuracy on text-only questions, a lead that is statistically significant over all other models. Performance on TI and TM categories is more comparable among the top models.

**Table 3:** Performance results in USMLE Step 2 CK. Asterisk (*) denotes a statistically significant difference (*p* < 0.05) compared to DeepSeek’s performance in that category.

**Step 2 CK**	ChatGPT	DeepSeek	Grok	Qwen
Text (n = 101)	88%*	98%	79%*	80%*
TI (n = 11)	73%	73%	64%	64%
TM (n = 8)	62%	62%	75%	75%

In Step 3, DeepSeek again demonstrates robust performance on text-only items. Notably, in the multimodal ITM category, both ChatGPT and Qwen perform strongly, with no statistically significant difference between them and the leader, indicating varied strengths in complex, integrated reasoning.

**Table 4:** Performance results in USMLE Step 3. Asterisk (*) denotes a statistically significant difference (*p* < 0.05) compared to DeepSeek’s performance in that category.

Step 3	ChatGPT	DeepSeek	Grok	Qwen
Text (n = 109)	79%	86%	76%*	69%*
TI (n = 12)	67%	58%	41%	75%
TM (n = 12)	75%	75%	66%	58%*
ITM (n = 6)	83%	75%	66%	83%

### Error analysis

To understand the limitations and cognitive strategies employed by each model, we conducted a qualitative and quantitative error analysis. The performance breakdown highlights that DeepSeek exhibits the lowest overall error rate across all three USMLE steps. In contrast, ChatGPT shows slightly higher error incidence, particularly in Step 3. Grok demonstrates a relatively stable but moderate performance, slightly surpassing Qwen, which records the lowest average score at 73.6%.

#### Conceptual errors

Grok and Qwen display more frequent conceptual misunderstandings, especially in Step 1, where foundational biomedical knowledge is critical. Despite their shortcomings in conceptual reasoning, both models perform reasonably well on straightforward text-based questions, indicating baseline competency in factual recall. In image-based and multimodal tasks, DeepSeek consistently outperforms ChatGPT, suggesting stronger visual-textual reasoning integration. Grok and Qwen yield comparable scores in these tasks, showing no substantial divergence in performance. Additionally, the questions requiring mathematical reasoning are generally handled well across all models, with Grok showing slightly more consistent accuracy than Qwen. DeepSeek handles quantitative reasoning more robustly than ChatGPT, especially in Step 1, where arithmetic application to clinical contexts is essential. Qwen presents a unique limitation by refusing to answer two image-based questions (Question 108 from Step 2 CK and Question 76 from Step 3), displaying a warning message: *“Content security warning: Image input data may contain inappropriate content.”* These were excluded from analysis. This highlights the need to refine safe-handling mechanisms for complex clinical content.

Overall, the models exhibit higher accuracy in questions requiring basic and intermediate clinical understanding. For multimodal questions in Step 3, the combined correct rate was 70.31%, while for mathematical reasoning tasks, it was 76.47%, indicating a 6.16% advantage in handling quantitative reasoning over multimodal integration.

#### Misinterpretations

The models frequently produce similar incorrect responses in Step 1 and Step 3, suggesting alignment in logic pathways. ChatGPT is more error-prone in complex case-based scenarios, whereas Grok performs better in image-supported questions. Qwen shows mixed performance, achieving reasonable accuracy in ITM tasks but struggling with intricate diagnostic reasoning. DeepSeek consistently achieves high performance across all formats, with an average accuracy of 88% in both text-only and image-text questions. In Step 2 CK, inconsistencies between Grok and Qwen’s answers indicate divergent logic paths and reinforce their potential for complementary synergy.

#### Patterns-based errors

ChatGPT and DeepSeek exhibit superior contextual pattern recognition, minimizing repetitive reasoning flaws. Grok performs reliably, while Qwen struggles to match the top-tier models, particularly in recognizing clinical patterns and integrating patient history into accurate predictions.

#### Illustrative failure cases

To provide deeper qualitative insight, we analyzed specific failure cases from the dataset that exemplify medically critical errors.

- *Conceptual misunderstanding (physiology - Step 1, Q3):* This question describes a patient with severe obesity and obstructive sleep apnea who has developed chronic respiratory acidosis (PCO2 of 70, pH of 7.31) and chronic hypoxemia (PO2 of 50). It asks for the most likely additional finding. The correct answer is an increased hemoglobin concentration due to EPO stimulation from chronic hypoxemia.
  - *Model failure:* DeepSeek answered this incorrectly.
  - *Analysis:* This failure represents a conceptual misunderstanding of a key physiological adaptation. The model failed to make the multi-step connection from chronic low oxygen to the body’s compensatory response of producing more red blood cells (secondary polycythemia). Mistaking this for a different finding (e.g., decreased bicarbonate, which is the opposite of the expected metabolic compensation) is a critical error in physiological reasoning.
- *Misinterpretation (Cardiology/ACLS - Step 2 CK, Q17):* A 16-year-old girl with a history of palpitations presents with an ECG showing a narrow-complex tachycardia at 240/min. Critically, her blood pressure is low at 90/60 mm Hg, indicating hemodynamic instability. The question asks for the most appropriate next step.
  - *Model failure:* All models except for Qwen answered this incorrectly. The failing models likely chose adenosine, the standard treatment for a *stable* patient.
  - *Analysis:* This widespread failure is a classic and critical misinterpretation of the full clinical picture. The models correctly identified the rhythm (SVT) but failed to interpret the hypotension as a sign of instability. According to ACLS guidelines, unstable tachycardia requires immediate synchronized cardioversion, not medication. This error highlights a dangerous inability to prioritize vital signs over the underlying rhythm when making a time-sensitive management decision.
- *Reasoning error (endocrinology/oncology - Step 3, Q53):* A patient with a known history of small cell carcinoma of the lung presents with confusion. Lab results show severe hyponatremia (Na+ 120), low serum osmolality (250), and paradoxically high urine osmolality (400). The question asks for the underlying cause.
  - *Model failure:* All five evaluated models answered this question incorrectly.
  - *Analysis:* This universal failure on a classic case presentation represents a profound multi-step reasoning error. A clinician is expected to connect three key pieces of data: (1) the patient’s cancer history (small cell is a known cause of paraneoplastic syndromes), (2) the lab pattern of euvolemic hyponatremia, and (3) the paradoxical urine concentration. Synthesizing these facts leads directly to the diagnosis of SIADH. The models’ inability to link the patient’s history to the contradictory lab values demonstrates a critical weakness in advanced, multi-source clinical reasoning.

### Model synergy

An inter-model comparison of incorrect responses reveals minimal overlap, particularly in Step 2 CK. This indicates that each model applies distinct reasoning processes and makes unique types of errors. For instance, when one model fails to answer correctly, others often succeed, suggesting that a hybrid approach could mitigate individual model weaknesses. This complementarity supports the hypothesis that ensemble or voting-based multi-model systems can provide a more robust and error-resilient framework for clinical decision support.

### Comparative statistical significance

To determine the reliability of performance differences, we conducted chi-square ($\chi ^2$) tests. The results indicate no significant difference ($p > 0.05$) in the accuracy across Step 1, Step 2 CK, and Step 3 between ChatGPT and DeepSeek. Similarly, comparisons between Grok and Qwen also show no significant difference ($p > 0.05$), suggesting comparable reliability within each pair.

Furthermore, inter-rater reliability was assessed using Cohen’s kappa ($\kappa$) statistic. Between ChatGPT and DeepSeek, the kappa score was 0.91, reflecting a high degree of agreement in evaluation consistency. Between Grok and Qwen, the kappa score was even higher at 0.96, indicating near-perfect consistency. These high agreement levels confirm the robustness of the grading methodology and reinforce the credibility of the comparative findings.

### Effect of follow-up prompts on response stability

We analyzed the effect of the follow-up prompt, *“Are you sure?”*, on the models’ final answers. Across all models, the prompt led to answer changes in approximately 4–8% of questions, as summarized in Table 5. Most of these changes did not consistently improve accuracy; in several cases, models flipped a correct answer to an incorrect one. This finding suggests that such prompt-induced revisions are not a reliable mechanism for improving model performance in this context.

**Table 5:** Effect of “Are you sure?” Follow-up Prompt on Model Responses. “Net Accuracy Change” refers to the percentage point change in overall accuracy resulting from the revised answers.

Model	No. of answer changes	% of total questions (n=376)	Net accuracy change (%)
ChatGPT	22	5.9	−0.53
DeepSeek	15	4.0	+0.27
Grok	30	8.0	−0.53
Qwen	25	6.6	−0.27

## Discussion and limitation

### Relation to prior benchmarks

Recent studies have evaluated biomedical QA performance of large language models using diverse datasets such as MMLU-Med, PubMedQA, MedQA, and MedMCQA. More rigorous benchmarks have also emerged, including retrieval-augmented frameworks like MIRAGE^21^, contextual adaptation pipelines such as BriefContext^22^, analyses of visual-textual reasoning failures in GPT-4V^23^, and robustness evaluations such as VLDBench^24^. These studies demonstrate the importance of multi-dataset evaluation, advanced prompting strategies (e.g., chain-of-thought, retrieval augmentation), and robust error analyses.

Our study differs in its focus on a single, standardized benchmark of critical importance in medical education: the USMLE. Unlike prior work that aggregates across multiple datasets, we conduct a stepwise evaluation across all three stages of the USMLE, providing fine-grained insight into model capabilities in foundational science, clinical knowledge, and applied reasoning. Furthermore, our contribution extends beyond accuracy reporting by incorporating qualitative error categorization, refusal behavior analysis, and inter-model synergy assessment. This unique design highlights not only the accuracy of the models but also their error patterns, blind spots, and complementary strengths, thereby offering a clinically meaningful perspective that complements broader multi-dataset benchmarks.

This study provides a comprehensive benchmark of four contemporary large language models on the USMLE, building upon a growing body of literature evaluating AI in medical licensing examinations. Previous work has established that LLMs can achieve passing scores, with early studies on ChatGPT demonstrating its potential even without specialized medical training^11,14^, and domain-specific models like Med-PaLM 2 setting high benchmarks with accuracies exceeding 85%^16^. Our findings align with this trajectory of improvement, showing that newer generalist models like DeepSeek and ChatGPT-4o Mini have largely closed the gap with specialized systems on this task. Our top-performing model, DeepSeek, achieved accuracies (89% on Step 1, 93% on Step 2 CK, 84% on Step 3) that are not only statistically comparable to ChatGPT-4o Mini but are also competitive with the highest reported scores in the field.

The architectural enhancements, expanded training datasets, and refined instruction-tuning capabilities of DeepSeek and ChatGPT elucidate their consistently superior accuracy. These models demonstrate stronger performance on both text-based and multimodal inputs, including questions requiring mathematical reasoning and integrated image-text understanding. In particular, DeepSeek’s success on multimodal and quantitative questions highlights its improved handling of complex reasoning tasks that extend beyond factual recall.

A key contribution of this work is the error analysis and model synergy perspective. We found that across 376 questions, universal failures were rare: no question in Step 1 (n=119) was missed by all five evaluated models, only one question in Step 2 (0.83%) was universally incorrect, and five questions in Step 3 (3.65%) were missed by all models. These overlapping errors were concentrated in multimodal (text+image) and quantitative reasoning (TM) tasks, underscoring persistent challenges in vision-language integration and numerical reasoning. The rarity of universal failures demonstrates strong complementarity across models, suggesting that ensemble or voting-based systems could mitigate individual weaknesses and enhance reliability. However, the existence of universally failed items highlights that ensembles cannot fully overcome systematic blind spots, particularly in multimodal reasoning.

**Fig. 8:**
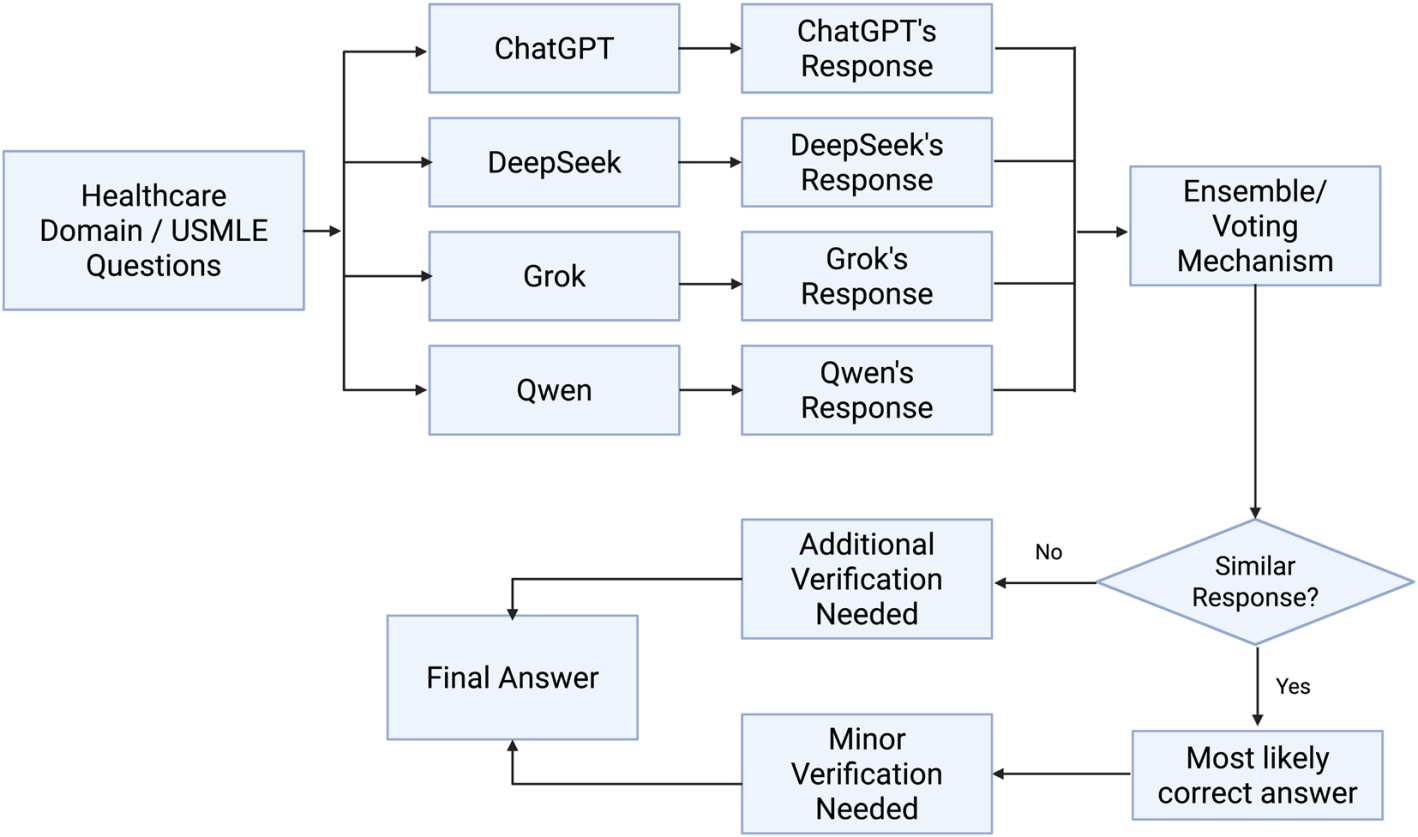
Consensus-based framework for combining multiple LLM responses to USMLE questions. Independent model outputs are aggregated through an ensemble/voting mechanism. Convergent answers are likely correct and require minor verification, while divergent answers trigger additional human oversight before reaching a final decision.

This observation directly informed the design of a proposed consensus-based framework, illustrated in Fig. 8. In this design, each model (ChatGPT, DeepSeek, Grok, and Qwen) independently processes a given USMLE-style question and provides its own response. These responses are then passed into an ensemble or voting mechanism. If model answers converge, the result is considered a *most likely correct answer*, requiring only minor verification. In cases of disagreement, however, the system flags the output for *additional human verification*. Ultimately, this framework produces a final answer while balancing automation with human oversight.

This result leads us to argue that such a design could also be valuable for healthcare professionals in real-world decision-making contexts. By integrating multiple model perspectives and systematically flagging disagreements for human review, the framework offers a pathway toward AI-assisted but human-validated clinical reasoning.

Inter-model agreement analysis further revealed minimal overlap in incorrect responses (low Fleiss’ Kappa), confirming that models employ divergent reasoning strategies. This diversity is beneficial for hybrid systems, but it also complicates interpretability, as disagreements may reflect inconsistent logic pathways. Importantly, we observed instances of overconfidence in incorrect answers, especially in ChatGPT and Grok, raising concerns about hallucination risks and the safety of deployment without uncertainty calibration.

However, several critical limitations must be acknowledged. The study’s reliance on publicly available sample exam questions, the relatively small dataset size for some question categories, and the exclusive use of a multiple-choice format all affect the generalizability of our findings. Beyond these structural issues, a more fundamental confounder is the potential for training data contamination. Models like ChatGPT and DeepSeek are trained on vast, internet-scale corpora that almost certainly include these public question banks and educational materials. This exposure could provide a non-trivial advantage, allowing the models to succeed through pattern recognition or memorization rather than genuine clinical reasoning. This makes it difficult to ascertain whether high scores reflect true diagnostic acumen or simply effective information retrieval^25^. Consequently, the performance differences observed might be less about superior reasoning and more about the nature and extent of each model’s training data; for instance, the comparatively lower scores of Grok or Qwen could be due to more restricted training datasets. Without transparent access to training sets, the true source of performance differences remains ambiguous.

A deeper diagnosis of the performance drop on multimodal (TI, ITM) tasks suggests that failures occur at multiple points in the reasoning pipeline. Our methodological approach of providing image-based questions as single image inputs that introduces a potential for Optical Character Recognition (OCR) failures, where text labels or values within the image are misread. However, a manual review of errors suggests a more fundamental issue with the vision-language models themselves. Failures can be categorized into: (1) Visual Interpretation Errors, where the vision component fails to identify the core pathology (e.g., misinterpreting ECG lead patterns or failing to spot a subtle radiographic finding), and (2) Text-Image Integration Errors, where the model correctly identifies visual features and textual clues but fails to synthesize them into a coherent clinical picture. The error in Step 2 CK, Q17 is a prime example of an integration failure, where models recognized the arrhythmia but failed to integrate the accompanying text (low blood pressure) to correctly determine the patient’s stability. These challenges indicate that while the vision encoders in modern LLMs are powerful, the architectural fusion between visual perception and clinical reasoning remains a significant bottleneck.

The novelty of our study lies in combining three elements: (1) evaluation across all USMLE steps, (2) qualitative error categorization into medically meaningful types (conceptual misunderstandings, misinterpretations, and reasoning errors), and (3) quantitative analysis of model synergy and overlap. Together, these provide insight into collective model behavior. While ensembles may substantially reduce error overlap, shared blind spots remain in multimodal and numerical reasoning, which align with well-recognized challenges in AI-driven radiology, pathology, and case integration.

From a deployment perspective, our findings reinforce the necessity of human oversight. For safe integration into clinical workflows, LLMs must be regarded as decision-support systems, not autonomous decision-makers. The emerging risk of overreliance on AI is particularly concerning. As models like the ones tested become more accurate, there is a substantial danger that clinicians may develop ’automation bias’–the tendency to over-trust automated outputs, leading to diminished critical judgment and the potential acceptance of erroneous AI suggestions^26^. This risk is multifaceted, encompassing skill atrophy, reduced situational awareness, and the diffusion of accountability, as highlighted by recent analyses in AI safety^21,27^. To better frame these challenges, we summarize key risks and mitigation strategies in Table 6. The overarching goal must be to design and deploy these systems in a way that augments clinical expertise and encourages active human engagement, rather than fostering passive acceptance.

**Table 6:** Risks of Overreliance on LLMs in Clinical Settings and Proposed Mitigation Strategies.

Identified risk	Description	Mitigation strategy
Automation bias	Tendency to uncritically accept AI-generated diagnoses or plans, even when they contradict one’s own clinical judgment or other evidence.	Design AI interfaces that highlight uncertainty, present alternative hypotheses, and require users to actively confirm or reject key findings rather than passively accepting a final answer.
Skill atrophy	Gradual erosion of fundamental clinical reasoning and diagnostic skills due to long-term dependence on AI for core cognitive tasks.	Integrate LLMs primarily as educational and information retrieval tools. Emphasize their use for differential diagnosis expansion or literature review, not as a replacement for foundational reasoning.
Accountability diffusion	Ambiguity regarding who is responsible for a medical error when an AI system is involved–the clinician, the institution, or the AI developer.	Establish clear institutional policies defining the roles and responsibilities of clinicians using AI tools. Ensure that the final clinical decision and legal accountability remain with the licensed human practitioner.

**Fig. 9:**
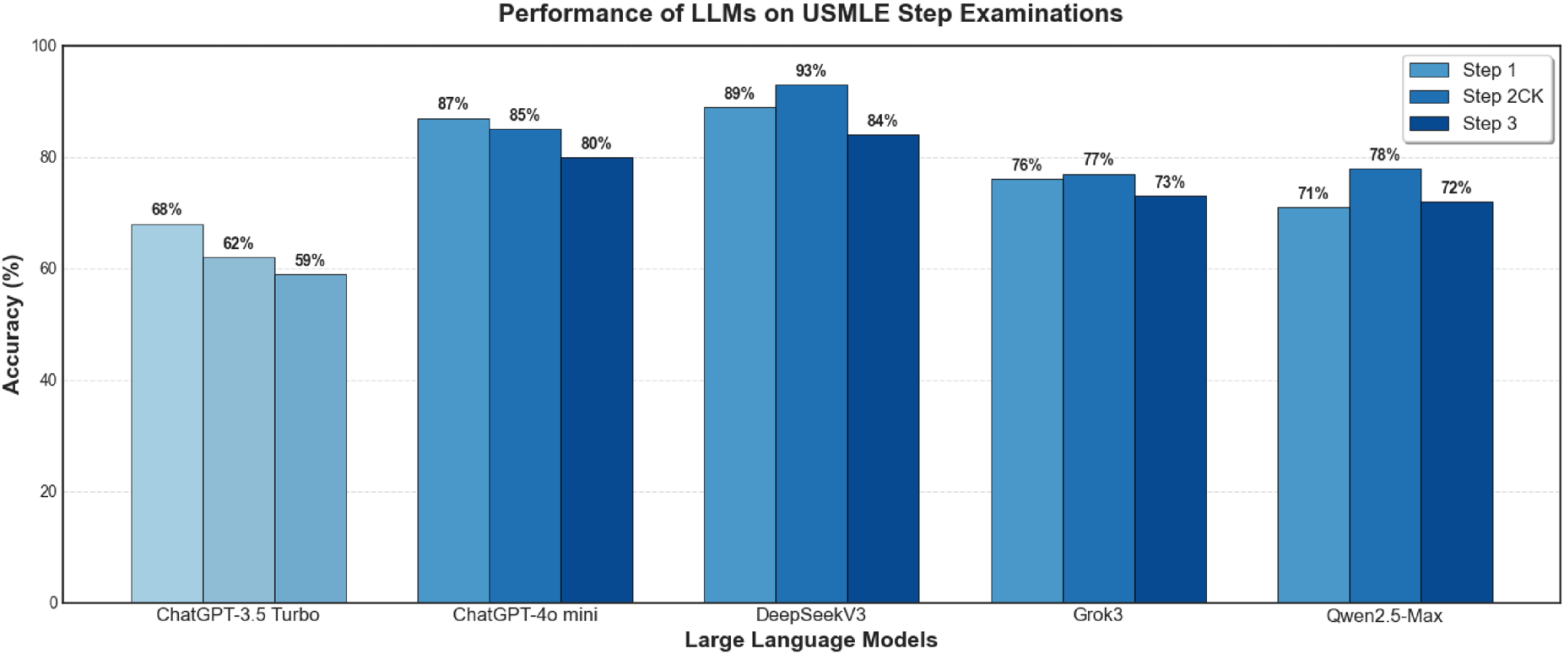
Overall performance comparison of all evaluated models across USMLE Step 1, Step 2 CK, and Step 3. The chart highlights the substantial performance gap between the newer models (DeepSeek, ChatGPT-4o Mini, Grok, Qwen) and the older baseline model (ChatGPT-3.5 Turbo).

The results from Fig. 9 also illustrate the limitations of older models such as ChatGPT-3.5 Turbo (Light Blue) compared to newer models like DeepSeek, Grok3, ChatGPT-4o Mini, and Qwen2.5-Max. While newer models achieve higher accuracy, it is important to emphasize that LLMs remain susceptible to confabulation or *hallucination*, generating plausible but incorrect information. This presents a significant safety risk if models are deployed without safeguards.

### Observed limitations of LLMs in clinical contexts

The rise of AI in medical education and decision-making necessitates the integration of human judgment and contextual oversight. Despite promising results, several limitations of current LLMs remain clear:

- *Refusal to answer certain questions:* Notably, Qwen declined to respond to two image-based clinical questions, returning a warning message: *“Content security warning: Image input data may contain inappropriate content”*. While such moderation safeguards serve important safety purposes, they also highlight barriers to deploying LLMs in sensitive clinical contexts.
- *Multimodal reasoning failures:* The drop in performance on image-based tasks stems from both flawed visual interpretation (e.g., misreading an ECG) and, more critically, a failure to integrate visual findings with textual patient data^28^. Models often analyze the two modalities in isolation, leading to clinically incongruent conclusions.
- *Hallucination and overconfidence:* A critical safety risk observed was the tendency for models, particularly ChatGPT and Grok, to produce highly confident but factually incorrect answers^29^. This phenomenon, often termed ’hallucination’, is dangerous in a clinical context where a plausible-sounding but fabricated piece of information can lead to diagnostic error^30^. The models’ overconfidence magnifies this risk, as they do not signal uncertainty, presenting both correct and incorrect information with the same degree of authority. This underscores the absolute necessity of rigorous verification of all AI-generated outputs by a qualified clinician before any information is used for patient care.
- *Limited chain-of-thought reasoning in low-performing models:* While DeepSeek and ChatGPT applied coherent reasoning in quantitative tasks, Grok and Qwen often lacked structured chains of thought, reducing their reliability in multi-step computations^31^.
- *Lack of clinical contextual awareness:* All four models occasionally failed to distinguish between similar diagnostic pathways or account for atypical presentations, highlighting the need for domain-specific fine-tuning and broader clinical training data.

We further address the limitation of Qwen’s refusal to answer two image-based questions due to its content moderation filters. Although this issue was identified and the questions were omitted, it highlights the challenge of content filtering and raises concerns about model reliability in sensitive medical contexts.

Overall, these results confirm both the promise and the limitations of LLMs in high-stakes clinical reasoning. While ensembles offer a path to greater robustness, shared weaknesses in multimodal and quantitative reasoning remain. Safely integrating LLMs in healthcare requires ongoing innovation, rigorous benchmarking, transparency in error reporting, and continuous collaboration between developers, physicians, and regulators.

## Ethical approval

Human participants, personal data, and photos are not used in the study. All USMLE sample questions were publicly available and anonymised. Thus, institutional review board (IRB) approval were not required.

## Conclusion

In this study, we provide a detailed benchmarking analysis of four large language models in terms of their performance on the USMLE, a standardized test for clinical knowledge and diagnostic reasoning. The results indicate that there is considerable variation in model performance. Specifically, DeepSeek and ChatGPT demonstrated better performance than Grok and Qwen on all three USMLE steps. Interestingly, DeepSeek attained the highest accuracy in Step 2 CK, which is a stage focused on a real-world clinical application, and this implies its use in clinical training decision support might be a viable option. The most important finding of our analysis is that there were very few shared mistakes among the models and it appears that each model is reasoning along unique pathways in mapping out the problem. This kind of complementarity facilitates the possibility of hybrid AI architectures that use the outputs of different models in combination to achieve lower rates of errors and better interpretability. This suggests ensemble methods or weighted decision strategies may be the tools leading to improved accuracy and robustness in high-risk clinical applications. Despite the satisfactory model performance, we found several limitations of the models. All the assessed models sometimes failed in understanding concepts, combining modalities, and recognizing domain-specific clinical cases. These issues show that there still needs to be development in large language models through fine-tuning them for specific domains and adding real contexts that make them more aware of clinical situations.

## Supplementary Information

Supplementary Information 1.

Supplementary Information 2.

Supplementary Information 3.

Supplementary Information 4.

Supplementary Information 5.

## Data Availability

The full dataset generated and analyzed during this study is available in a public repository to promote transparency and reproducibility. This includes the formatted questions used for model input, the complete raw outputs from each LLM, and the final scoring sheets. The data can be accessed from here: https://drive.google.com/drive/folders/1XE6u2okNTOz-Wqj44vmhUpt3uUhgk7px . As noted in the methodology, the original USMLE sample questions are copyrighted material and can be obtained directly from the official USMLE website. For any inquiries regarding the study’s dataset, please contact the corresponding author, Md Kamrul Siam (ksiam01@nyit.edu).
