# Supplementary material for: Benchmarking large language models on the United States medical licensing examination for clinical reasoning and medical licensing scenarios
Source: Sci Rep. 2025 Dec 3;16:1387. doi: 10.1038/s41598-025-31010-4 (PMC12796295; doi:10.1038/s41598-025-31010-4)
Supplement: Supplementary file 1 — Supplementary Information 1. [file 41598_2025_31010_MOESM1_ESM.pdf]

Sample Test Questions

Step 1

A Joint Program of the Federation of State Medical Boards of the United States, Inc. (FSMB), and National Board of Medical Examiners® (NBME®)

This booklet was updated January 2024.
For Public Release

Copyright © 2024 by the Federation of State Medical Boards of the United States, Inc. (FSMB), and National Board of Medical Examiners (NBME). All rights reserved. Printed in the United States of America. The United States Medical Licensing Examination (USMLE®) is a joint program of the FSMB and NBME.

CONTENTS

USMLE Step 1 Test Question Formats 3

Introduction to USMLE Step 1 Sample Test Questions 4

USMLE Laboratory Values 5

USMLE Step 1 Sample Test Questions... 8

Answer Form for USMLE Step 1 Sample Test Questions... 52

Answer Key for USMLE Step 1 Sample Test Questions... 53

USMLE STEP 1 TEST QUESTION FORMATS

Single-Item Questions
A single patient-centered vignette is associated with one question followed by four or more response options. The response options are lettered (A, B, C, D, E). A portion of the questions require interpretation of graphic or pictorial materials. You are required to select the best answer to the question. Other options may be partially correct, but there is only ONE BEST answer. This is the traditional, most frequently used multiple-choice question format on the examination.

Strategies for Answering Single One-Best-Answer Test Questions

The following are strategies for answering one-best-answer items:

- Read each patient vignette and question carefully. It is important to understand what is being asked.
- Try to generate an answer and then look for it in the response option list.
- Alternatively, read each response option carefully, eliminating those that are clearly incorrect.
- Of the remaining options, select the one that is most correct.
- If unsure about an answer, it is better to guess since unanswered questions are automatically counted as wrong answers.

Example Item

A 32-year-old woman with type 1 diabetes mellitus has had progressive renal failure during the past 2 years. She has not yet started dialysis. Examination shows no abnormalities. Her hemoglobin concentration is 9 g/dL, hematocrit is 28%, and mean corpuscular volume is 94 μm^3. A blood smear shows normochromic, normocytic cells. Which of the following is the most likely cause?

(A) Acute blood loss
(B) Chronic lymphocytic leukemia
(C) Erythrocyte enzyme deficiency
(D) Erythropoietin deficiency
(E) Immunohemolysis
(F) Microangiopathic hemolysis
(G) Polycythemia vera
(H) Sickle cell disease
(I) Sideroblastic anemia
(J) β-Thalassemia trait

(Answer: D)

NOTE: Some item types that appear on the Step 1 examination are NOT depicted in the sample items provided in this booklet, eg, items with multimedia features, such as audio. Also, when additional item formats are added to the exam, notice will be provided at the USMLE website: http://www.usmle.org. You must monitor the website to stay informed about the types of items that occur in the exam, and you must practice with the downloadable sample test items available on the USMLE website to be fully prepared for the examination.

INTRODUCTION TO USMLE STEP 1 SAMPLE TEST QUESTIONS

The following pages include 119 sample test questions. Most of these questions are the same as those you can install on your computer from the USMLE website. Please note that reviewing the sample questions as they appear on pages 8–51 is not a substitute for practicing with the test software. You should download and run the Step 1 tutorial and practice test items that are provided on the USMLE website well before your test date. The sample materials available on the USMLE website include an additional item with associated audio findings that does not appear in this booklet. You should become familiar with this item format that will be used in the actual examination.

Although the sample questions exemplify content on the Step 1 examination overall, they may not reflect the content coverage on individual examinations. In the actual examination, questions will be presented in random order; they will not be grouped according to specific content. The questions will be presented one at a time in a format designed for easy on-screen reading, including use of the USMLE Laboratory Values table (included here on pages 5–7) and some pictorials. Photographs, charts, and x-rays in this booklet are not of the same quality as the pictorials used in the actual examination. In addition, you will be able to adjust the brightness and contrast of pictorials on the computer screen.

To take the following sample test questions as they would be timed in the actual examination, you should allow a maximum of 1 hour for each 40-item block, and a maximum of 58 minutes, 30 seconds, for the 39-item block, for a total of 2 hours, 58 minutes, 30 seconds. Please note that the third block has 39 items instead of 40 because the multimedia item has been removed, and the recommended time to complete the block has been adjusted accordingly. Please be aware that most examinees perceive the time pressure to be greater during an actual examination. All examinees are strongly encouraged to practice with the downloadable version to become familiar with all item formats and exam timing. An answer form for recording answers is provided on page 52. An answer key is provided on page 53. In the actual examination, answers will be selected on the screen; no answer form will be provided.

	Reference Range	SI Reference Intervals
**SERUM**		
General Chemistry:		
Electrolytes		
Sodium (Na^+)	136–146 mEq/L	136–146 mmol/L
Potassium (K^+)	3.5–5.0 mEq/L	3.5–5.0 mmol/L
Chloride (Cl^-)	95–105 mEq/L	95–105 mmol/L
Bicarbonate (HCO_3^-)	22–28 mEq/L	22–28 mmol/L
Urea nitrogen	7–18 mg/dL	2.5–6.4 mmol/L
Creatinine	0.6–1.2 mg/dL	53–106 µmol/L
Glucose	Fasting: 70–100 mg/dL	3.8–5.6 mmol/L
	Random, non-fasting: <140 mg/dL	<7.77 mmol/L
Calcium	8.4–10.2 mg/dL	2.1–2.6 mmol/L
Magnesium (Mg^{2+})	1.5–2.0 mg/dL	0.75–1.0 mmol/L
Phosphorus (inorganic)	3.0–4.5 mg/dL	1.0–1.5 mmol/L
Hepatic:		
Alanine aminotransferase (ALT)	10–40 U/L	10–40 U/L
Aspartate aminotransferase (AST)	12–38 U/L	12–38 U/L
Alkaline phosphatase	25–100 U/L	25–100 U/L
Bilirubin, total // direct	0.1–1.0 mg/dL // 0.0–0.3 mg/dL	2–17 µmol/L // 0–5 µmol/L
Proteins, total	6.0–7.8 g/dL	60–78 g/L
Albumin	3.5–5.5 g/dL	35–55 g/L
Globulin	2.3–3.5 g/dL	23–35 g/L
Other, serum:		
Amylase	25–125 U/L	25–125 U/L
Lipase	13–60 U/L	13–60 U/L
Creatinine clearance	Male: 97–137 mL/min	97–137 mL/min
	Female: 88–128 mL/min	88–128 mL/min
Creatine kinase	Male: 25–90 U/L	25–90 U/L
	Female: 10–70 U/L	10–70 U/L
Lactate dehydrogenase	45–200 U/L	45–200 U/L
Osmolality	275–295 mOsmol/kg H_2O	275–295 mOsmol/kg H_2O
Troponin I	≤0.04 ng/mL	≤0.04 µg/L
Uric acid	3.0–8.2 mg/dL	0.18–0.48 mmol/L
Lipids:		
Cholesterol		
Total	Normal: <200 mg/dL	<5.2 mmol/L
	High: >240 mg/dL	>6.2 mmol/L
HDL	40–60 mg/dL	1.0–1.6 mmol/L
LDL	<160 mg/dL	<4.2 mmol/L
Triglycerides	Normal: <150 mg/dL	<1.70 mmol/L
	Borderline: 151–199 mg/dL	1.71–2.25 mmol/L
Iron Studies:		
Ferritin	Male: 20–250 ng/mL	20–250 µg/L
	Female: 10–120 ng/mL	10–120 µg/L
Iron	Male: 65–175 µg/dL	11.6–31.3 µmol/L
	Female: 50–170 µg/dL	9.0–30.4 µmol/L
Total iron-binding capacity	250–400 µg/dL	44.8–71.6 µmol/L
Transferrin	200–360 mg/dL	2.0–3.6 g/L

Continued on Next Page

	Reference Range	SI Reference Intervals
Endocrine:		
Follicle-stimulating hormone	Male: 4–25 mIU/mL	4–25 IU/L
	Female: premenopause 4–30 mIU/mL	4–30 IU/L
	midcycle peak 10–90 mIU/mL	10–90 IU/L
	postmenopause 40–250 mIU/mL	40–250 IU/L
Luteinizing hormone	Male: 6–23 mIU/mL	6–23 IU/L
	Female: follicular phase 5–30 mIU/mL	5–30 IU/L
	midcycle 75–150 mIU/mL	75–150 IU/L
	postmenopause 30–200 mIU/mL	30–200 IU/L
Growth hormone - arginine stimulation	Fasting: <5 ng/mL	<5 µg/L
	Provocative stimuli: >7 ng/mL	>7 µg/L
Prolactin (hPRL)	Male: <17 ng/mL	<17 µg/L
	Female: <25 ng/mL	<25 µg/L
Cortisol	0800 h: 5–23 µg/dL	138–635 nmol/L
	1600 h: 3–15 µg/dL	82–413 nmol/L
	2000 h: <50% of 0800 h	Fraction of 0800 h: <0.50
TSH	0.4–4.0 µU/mL	0.4–4.0 mIU/L
Triiodothyronine (T_3) (RIA)	100–200 ng/dL	1.5–3.1 nmol/L
Triiodothyronine (T_3) resin uptake	25%–35%	0.25–0.35
Thyroxine (T_4)	5–12 µg/dL	64–155 nmol/L
Free T_4	0.9–1.7 ng/dL	12.0–21.9 pmol/L
Thyroidal iodine (^{123}I) uptake	8%–30% of administered dose/24 h	0.08–0.30/24 h
Intact PTH	10–60 pg/mL	10–60 ng/L
17-Hydroxycorticosteroids	Male: 3.0–10.0 mg/24 h	8.2–27.6 µmol/24 h
	Female: 2.0–8.0 mg/24 h	5.5–22.0 µmol/24 h
17-Ketosteroids, total	Male: 8–20 mg/24 h	28–70 µmol/24 h
	Female: 6–15 mg/24 h	21–52 µmol/24 h
Immunoglobulins:		
IgA	76–390 mg/dL	0.76–3.90 g/L
IgE	0–380 IU/mL	0–380 kIU/L
IgG	650–1500 mg/dL	6.5–15.0 g/L
IgM	50–300 mg/dL	0.5–3.0 g/L
**GASES, ARTERIAL BLOOD (ROOM AIR)**		
P_{O_2}	75–105 mm Hg	10.0–14.0 kPa
P_{CO_2}	33–45 mm Hg	4.4–5.9 kPa
pH	7.35–7.45	[H^+] 36–44 nmol/L
**CEREBROSPINAL FLUID**		
Cell count	0–5/mm^3	0–5 × 10^6/L
Chloride	118–132 mEq/L	118–132 mmol/L
Gamma globulin	3%–12% total proteins	0.03–0.12
Glucose	40–70 mg/dL	2.2–3.9 mmol/L
Pressure	70–180 mm H_2O	70–180 mm H_2O
Proteins, total	<40 mg/dL	<0.40 g/L

Continued on Next Page

	Reference Range	SI Reference Intervals
**HEMATOLOGIC**		
Complete Blood Count:		
Hematocrit	Male: 41%–53%	0.41–0.53
	Female: 36%–46%	0.36–0.46
Hemoglobin, blood	Male: 13.5–17.5 g/dL	135–175 g/L
	Female: 12.0–16.0 g/dL	120–160 g/L
Mean corpuscular hemoglobin (MCH)	25–35 pg/cell	0.39–0.54 fmol/cell
Mean corpuscular hemoglobin conc. (MCHC)	31%–36% Hb/cell	4.8–5.6 mmol Hb/L
Mean corpuscular volume (MCV)	80–100 μm^3	80–100 fL
Volume		
Plasma	Male: 25–43 mL/kg	0.025–0.043 L/kg
	Female: 28–45 mL/kg	0.028–0.045 L/kg
Red cell	Male: 20–36 mL/kg	0.020–0.036 L/kg
	Female: 19–31 mL/kg	0.019–0.031 L/kg
Leukocyte count (WBC)	4500–11,000/mm^3	4.5–11.0 × 10^9/L
Neutrophils, segmented	54%–62%	0.54–0.62
Neutrophils, bands	3%–5%	0.03–0.05
Lymphocytes	25%–33%	0.25–0.33
Monocytes	3%–7%	0.03–0.07
Eosinophils	1%–3%	0.01–0.03
Basophils	0%–0.75%	0.00–0.0075
Platelet count	150,000–400,000/mm^3	150–400 × 10^9/L
Coagulation:		
Partial thromboplastin time (PTT/aPTT) (activated)	25–40 seconds	25–40 seconds
Prothrombin time (PT)	11–15 seconds	11–15 seconds
D-Dimer	≤250 ng/mL	≤1.4 nmol/L
Other, Hematologic:		
Reticulocyte count	0.5%–1.5%	0.005–0.015
Erythrocyte count (RBC)	Male: 4.3–5.9 million/mm^3	4.3–5.9 × 10^{12}/L
	Female: 3.5–5.5 million/mm^3	3.5–5.5 × 10^{12}/L
Erythrocyte sedimentation rate (Westergren)	Male: 0–15 mm/h	0–15 mm/h
	Female: 0–20 mm/h	0–20 mm/h
CD4+ T-lymphocyte count	≥500/mm^3	≥0.5 × 10^9/L
Endocrine:		
Hemoglobin A_{1c}	≤6%	≤42 mmol/mol
**URINE**		
Calcium	100–300 mg/24 h	2.5–7.5 mmol/24 h
Osmolality	50–1200 mOsmol/kg H_2O	50–1200 mOsmol/kg H_2O
Oxalate	8–40 µg/mL	90–445 µmol/L
Proteins, total	<150 mg/24 h	<0.15 g/24 h
**BODY MASS INDEX (BMI)**	Adult: 19–25 kg/m^2	

USMLE STEP 1 SAMPLE TEST QUESTIONS

BLOCK 1, ITEMS 1-40

1. A 14-year-old boy is brought to the emergency department by his parents because of a 1-month history of intermittent right knee pain that has worsened during the past day. He rates his current pain as a 6 on a 10-point scale and says that it worsens when he walks and lessens when he sits. During the past 2 weeks, he has been walking 1 mile daily in preparation for participation in the school marching band. He has not taken any medications for his pain. He sustained a right tibia and fibula fracture at the age of 8 years after a skateboarding accident, which was treated with internal fixation and casting. He has asthma treated with inhaled budesonide daily and inhaled albuterol as needed. His mother has type 2 diabetes mellitus, and his maternal grandmother has osteoporosis. The patient is 170 cm (5 ft 7 in; 77th percentile) tall and weighs 88 kg (195 lb; >95th percentile); BMI is 31 kg/m^2 (98th percentile). Temperature is 37.0°C (98.6°F), pulse is 95/min, and blood pressure is 130/80 mm Hg. Physical examination shows hyperpigmented, thickened skin at the nape of the neck. There is tenderness to palpation of the anterior aspect of the right hip and limited range of motion on abduction, internal rotation, and flexion of the right hip. The left hip and knees are nontender; range of motion is full in all directions. The remainder of the examination discloses no abnormalities. Which of the following factors in this patient’s history most increased his risk for developing this condition?

(A) BMI
(B) Family history
(C) Medication use
(D) Previous fractures
(E) Recent physical activity

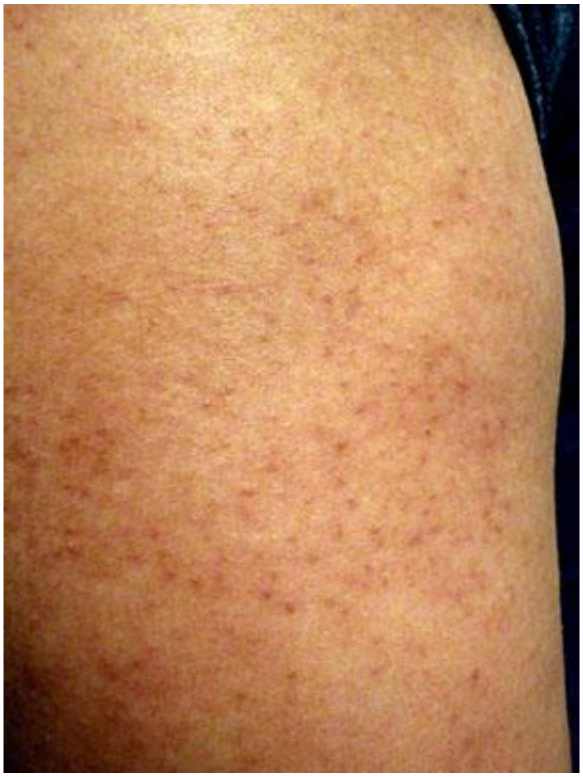

2. A 14-year-old girl is brought to the office by her mother because of a 3-month history of red bumps on her skin. The patient says the bumps are not itchy or painful but that she finds them embarrassing. She has no history of major medical illness and takes no medications. Her vital signs are within normal limits. Physical examination shows the findings in the photograph. Which of the following is the most likely diagnosis?

(A) Eczema
(B) Folliculitis
(C) Hidradenitis
(D) Keratosis pilaris
(E) Urticaria

---

3. A 50-year-old man comes to the office because of a 2-month history of increasing daytime somnolence. He has obstructive sleep apnea for which he has only intermittently used a continuous positive airway pressure device. He is 170 cm (5 ft 7 in) tall and weighs 181 kg (400 lb); BMI is 63 kg/m^2. His temperature is 37°C (98.6°F), pulse is 100/min, respirations are 12/min, and blood pressure is 135/80 mm Hg. Physical examination shows a gray-blue tinge to the lips, earlobes, and nail beds. Cardiac examination shows no other abnormalities. Arterial blood gas analysis on room air shows a pH of 7.31, P_{CO_2} of 70 mm Hg, and P_{O_2} of 50 mm Hg. Which of the following additional findings would be most likely in this patient?

(A) Decreased serum bicarbonate concentration
(B) Increased hemoglobin concentration
(C) Increased total lung capacity
(D) Left ventricular hypertrophy

4. A 32-year-old man comes to the office because of a 1-day history of cough productive of small amounts of blood and a 2-day history of shortness of breath and swelling of his ankles. He also has a 2-week history of progressive fatigue and episodes of dark urine. He has no history of major medical illness and takes no medications. His temperature is 37°C (98.6°F), pulse is 90/min, respirations are 18/min, and blood pressure is 175/110 mm Hg. Pulse oximetry on room air shows an oxygen saturation of 91%. Diffuse inspiratory crackles are heard over all lung bases. There is 2+ pitting edema of both ankles. Results of laboratory studies are shown:

Hemoglobin	8.9 g/dL
Hematocrit	27%
Serum	
Urea nitrogen	55 mg/dL
Creatinine	2.9 mg/dL
Urine RBC	20–40/hpf

Urinalysis also shows some dysmorphic RBCs and rare RBC casts. Examination of a kidney biopsy specimen shows crescentic glomerulonephritis and linear deposition of IgG along the glomerular capillaries. This patient most likely has antibodies directed against which of the following antigens?

(A) Collagen
(B) Double-stranded DNA
(C) Nucleolar protein
(D) Phospholipid
(E) Proteins in neutrophil cytoplasm

---

5. A 5-year-old girl is brought to the emergency department because of a 2-day history of fever, urinary urgency, and burning pain with urination. She has had four similar episodes during the past year. A diagnosis of urinary tract infection is made. Subsequent renal ultrasonography shows one large U-shaped kidney. Which of the following is the most likely embryologic origin of this patient's condition?

(A) Failure of the kidneys to rotate 90 degrees medially
(B) Failure of normal kidney ascent
(C) Failure of one ureteric bud to develop normally
(D) Fusion of the inferior poles of the metanephros during ascent

6. A 78-year-old man comes to the office for a follow-up examination. He was discharged from the hospital 1 week ago after being treated for a nontuberculous mycobacterial infection. He started treatment with ciprofloxacin and rifampin at that time. He also has hypertension and underwent placement of a mechanical aortic valve 6 years ago for aortic stenosis. Other current medications are hydrochlorothiazide, lisinopril, and warfarin. His warfarin dose was doubled 4 days ago. He says that he is trying to follow a healthier diet. He drinks two 12-oz beers daily. Results of laboratory studies done 4 days ago and today are shown

	**4 Days Ago**	**Today**
Prothrombin time	11 sec (INR=1)	11.2 sec (INR=1.1)
Partial thromboplastin time	29 sec	27 sec

Which of the following is the most likely cause of this patient's laboratory findings?

(A) Decreased protein binding
(B) Eradication of gut flora
(C) Increased alcohol intake
(D) Increased vegetable consumption
(E) Induction of cytochrome enzymes

---

7. A 32-year-old man comes to the office because of a 2-week history of fever and throat pain. He is 173 cm (5 ft 8 in) tall and weighs 63 kg (140 lb); BMI is 21 kg/m^2. His pulse is 110/min, respirations are 16/min, and blood pressure is 98/68 mm Hg. Physical examination shows scattered 2- to 4-cm lymph nodes in the neck, axillae, and inguinal regions. There is a bilateral tonsillar exudate but no ulcerations. Results of laboratory studies are shown:

Hemoglobin	9.6 g/dL
Hematocrit	29%
Leukocyte count	1500/mm^3
Platelet count	60,000/mm^3

A heterophile antibody test result is negative. Which of the following is the most likely diagnosis?

(A) Epstein-Barr virus infection
(B) Gonococcal pharyngitis
(C) HIV infection
(D) Lymphogranuloma venereum infection
(E) Streptococcal pharyngitis

---

8. A 50-year-old man comes to the office for a follow-up examination. He has a 2-month history of headache and shortness of breath with exertion. He also has hypertension treated with hydrochlorothiazide for the past 2 years. His blood pressure is 180/105 mm Hg. Ophthalmoscopic examination is most likely to show which of the following in this patient?

(A) Arteriovenous nicking
(B) Melanocytes in the uvea
(C) Optic neuritis
(D) Posterior subcapsular cataracts
(E) Tractional retinal detachment

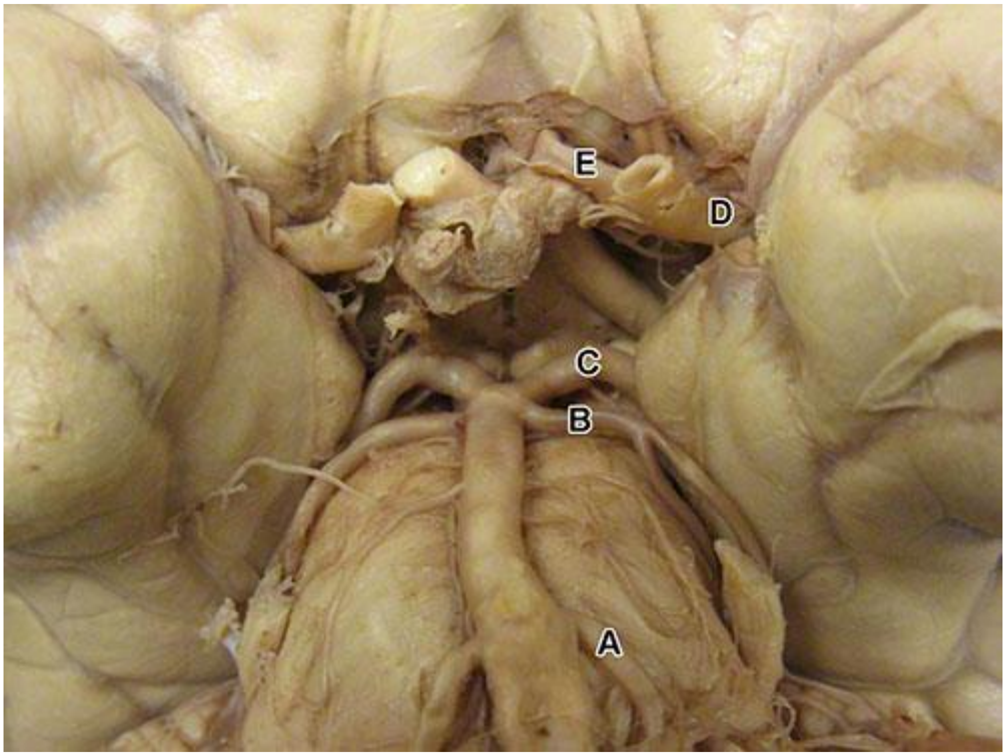

9. A 65-year-old woman with a history of rheumatic mitral valve disease is brought to the emergency department 30 minutes after the sudden onset of right-sided weakness and inability to speak. Neurologic examination shows weakness of the right lower side of the face and difficulty swallowing. Muscle strength is 3/5 on the right side. She can understand what is said to her, but she cannot repeat words or answer questions. An ECG shows atrial fibrillation. The most likely cause of the neurologic findings in this patient is occlusion of which of the following labeled arteries in the photograph of a normal brain?

(A) A
(B) B
(C) C
(D) D
(E) E

---

10. A 51-year-old man with a 10-year history of gastroesophageal reflux and suspected Barrett esophagus comes to the office because his omeprazole dose "doesn't work around the Christmas holidays." He states that he prides himself on having a large appetite and "holding his liquor" during the holidays. He currently takes the maximum dose of omeprazole. Which of the following is the most appropriate initial action by the physician?

(A) Ask the patient how much he is eating and drinking during the holidays
(B) Explain the hazards of untreated reflux in the presence of Barrett esophagus
(C) Order an upper endoscopy
(D) Refer the patient to a gastroenterologist
(E) Switch the omeprazole to pantoprazole

11. A 60-year-old man comes to the office because of weakness, tingling of his hands and feet, irritability, and forgetfulness for 4 months. Physical examination shows pallor, weakness, and spasticity. Deep tendon reflexes are increased. Sensation to vibration is absent in the lower extremities. Laboratory studies show megaloblastic anemia, serum antiparietal cell antibodies, and increased serum concentrations of methylmalonic acid and total homocyst(e)ine. The synthesis of which of the following amino acids is most likely impaired in this patient?

(A) Cysteine
(B) Glutamine
(C) Methionine
(D) Phenylalanine
(E) Tyrosine

12. A 65-year-old woman comes to the office for a follow-up examination 1 year after she underwent operative resection of the right colon and chemotherapy for stage III colon cancer. She reports fatigue. Physical examination shows no abnormalities. A staging CT scan of the chest and abdomen shows five new 2- to 3-cm masses in the liver and both lungs. This patient's cancer most likely spread to the lungs via which of the following structures?

(A) Inferior mesenteric vein
(B) Inferior vena cava
(C) Left colic vein
(D) Middle colic artery
(E) Pulmonary vein
(F) Superior mesenteric artery
(G) Superior vena cava

13. A 26-year-old man comes to the office because of a 1-week history of increased urinary frequency accompanied by excessive thirst. He says he has been urinating hourly. Physical examination shows no abnormalities. Serum chemistry studies are within the reference ranges. Urine osmolality is 50 mOsmol/kg H_2O. After administration of ADH (vasopressin), his urine osmolality is within the reference range. The most likely cause of this patient's symptoms is dysfunction of which of the following structures?

(A) Anterior pituitary gland
(B) Bowman capsule
(C) Glomerulus
(D) Hypophysial portal system
(E) Loop of Henle
(F) Supraoptic nucleus

14. A 52-year-old woman comes to the office because of a 6-month history of intermittent headaches. Sometimes the pain improves when the patient lies down in a quiet room. Her temperature is 37.5°C (99.5°F), pulse is 86/min, respirations are 16/min, and blood pressure is 154/100 mm Hg. The lungs are clear. Cardiac examination shows the point of maximal impulse displaced to the left and occasional skipped beats; there are no murmurs or rubs. There is no S_3. Resting electrocardiography shows left axis deviation with R waves greater than 30 mm in leads V_5 through V_6. Which of the following processes best explains the development of the left ventricular abnormalities in this patient?

(A) Excessive accumulation of glycogen
(B) Fibrosis of intraventricular conduction pathways
(C) Increased synthesis of contractile filaments
(D) Misfolding and aggregation of cytoskeletal proteins
(E) Myocyte hyperplasia as a result of induction of embryonic genes

15. A 53-year-old man comes to the physician because of a dry scaly rash on his body for the past year. He has had a 15-kg (33-lb) weight loss during the past year. He is 178 cm (5 ft 10 in) tall and now weighs 54 kg (120 lb); BMI is 17 kg/m^2. His stools have a large volume and float. Which of the following nutrient deficiencies is most likely?

(A) Magnesium
(B) Vitamin A
(C) Vitamin B_{12} (cobalamin)
(D) Vitamin C
(E) Zinc

16. Serum LDL-cholesterol concentrations are measured in blood samples collected from 25 healthy volunteers. The data follow a normal distribution. The mean and standard deviation for this group are 130 mg/dL and 25 mg/dL, respectively. The standard error of the mean is 5.0. With a 95% confidence level, the true mean for the population from which this sample was drawn falls within which of the following ranges (in mg/dL)?

(A) 105-155
(B) 120-140
(C) 125-135
(D) 128-132
(E) 129-131

17. A 39-year-old man comes to the physician because of a 6-month history of progressive shortness of breath. He has had a cough productive of white sputum for 2 years. He smoked 1 pack of cigarettes daily for 16 years but quit 10 years ago. He is in mild respiratory distress with pursed lips and a barrel chest; he is using the accessory muscles of respiration. Breath sounds are distant and crackles are present in the lower lung fields bilaterally. Pulmonary function tests show a decreased FEV_1:FVC ratio, increased residual volume, and decreased diffusion capacity. An x-ray of the chest shows hyperinflation and hypertranslucency of the lower lobes of both lungs. Which of the following is the most likely diagnosis?

(A) Asthma
(B) Bronchiectasis
(C) Chronic pulmonary fibrosis
(D) Cystic fibrosis
(E) Emphysema

18. Investigators conduct a study that evaluates the effect of finasteride on the incidence of prostate cancer in 500 patients. The investigators recruit an additional 1000 patients for the study. Which of the following effects will this have on the research study?

(A) Greater chance of a Type I error
(B) Greater chance of a Type II error
(C) Less chance of a Type I error
(D) Less chance of a Type II error
(E) Impossible to predict

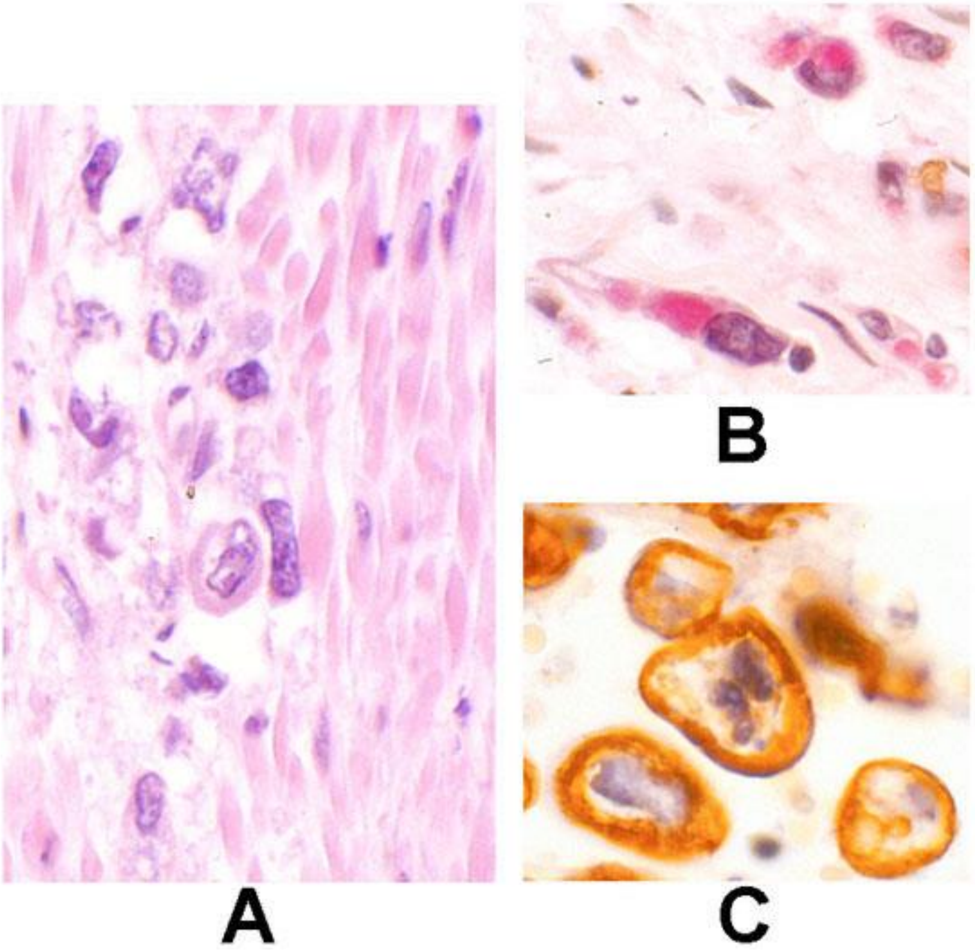

19. An 82-year-old woman has a 7-month history of increasing indigestion, upper abdominal bloating, and early satiety. Photomicrographs of her gastric wall are shown: A is a hematoxylin and eosin stain; B is a mucin stain; C is an immunostain for cytokeratin. Which of the following is most likely to have decreased expression in these cells?

(A) Cathepsin D
(B) Epithelial cadherins
(C) Heparin-binding fibroblast growth factors
(D) Integrins
(E) Type IV collagenase
(F) Vascular endothelial growth factor

---

20. A 54-year-old woman comes to the physician because she would like to lose weight. She has been on numerous diets in the past with limited success. Both her parents have type 2 diabetes mellitus. She is 160 cm (5 ft 3 in) tall and weighs 69 kg (152 lb); BMI is 27 kg/m^2. Her blood pressure is 140/90 mm Hg. Fasting serum glucose concentration is 102 mg/dL. Compared with a woman of the same age whose weight is normal, which of the following serum abnormalities is most likely in this patient?

(A) Decreased cholesterol excretion
(B) Decreased estrone concentration
(C) Decreased leptin concentration
(D) Increased fasting insulin concentration
(E) Increased growth hormone concentration
(F) Increased thyroid-stimulating hormone concentration

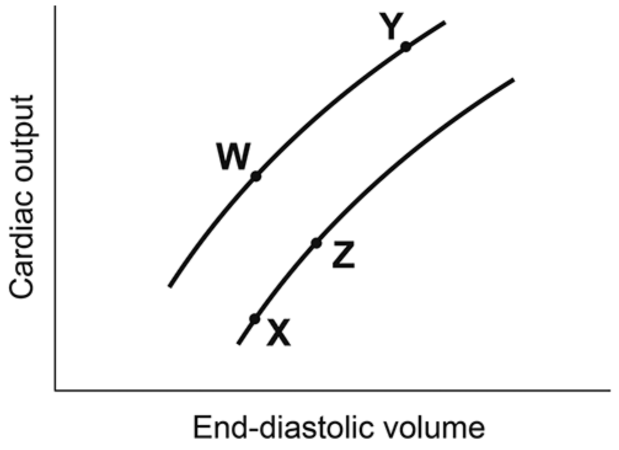

21. A 65-year-old woman is brought to the emergency department because of a 10-minute history of chest tightness and severe pain of her left arm. Physical examination shows jugular venous distention. Crackles are heard over the lung fields. An ECG shows ST-segment elevation greater than 1 mm in leads V_4 through V_6 and new Q waves. Serum studies show an increased troponin I concentration. Which of the following labeled points in the graph best represents the changes in cardiac function that occurred during the first 10 seconds after the onset of pain in this patient?

(A) W → X
(B) W → Y
(C) W → Z
(D) X → W
(E) X → Y
(F) X → Z
(G) Z → W
(H) Z → X
(I) Z → Y

---

22. A previously healthy 33-year-old woman is brought to the emergency department by the Secret Service for stalking the president of the USA for 2 months. She claims to be married to the president's twin brother and states that the president just had his twin kidnapped to avoid competition. She speaks rapidly and is difficult to interrupt. Her associations are often loose. She says, "I haven't slept for days, but I won't even try to sleep until my husband is rescued. God has been instructing me to take over the White House. I can't wait to be reunited with my husband. I hear his voice telling me what to do." When asked about drug use, she says she uses only natural substances. She refuses to permit blood or urine tests, saying, "I don't have time to wait for the results." Which of the following is the most likely diagnosis?

(A) Bipolar disorder, manic, with psychotic features
(B) Brief psychotic disorder
(C) Delusional disorder
(D) Psychotic disorder due to general medical condition
(E) Schizophrenia

23. In informing a couple that their newborn has Down syndrome, there is a specific, relatively limited amount of information that the consulting physician should give immediately. The rest can be discussed at a later time. Which of the following best explains the purpose of using this approach to disclosure?

(A) Allowing the couple's primary care physician to discuss most of the information with them
(B) Allowing the parents time to tell other family members
(C) Delaying parental distress until the information is completely disclosed
(D) Disclosing the most important information so that it can be understood as fully as possible
(E) Influencing the parents' course of action about what is medically most appropriate

24. A 62-year-old man comes to the physician because of a 6-month history of urinary hesitancy and dribbling after urination. He has to urinate two to three times nightly. Physical examination shows a diffusely enlarged, firm, and nontender prostate. Which of the following is most likely to have contributed to the development of this patient's condition?

(A) Activation of the α_1-adrenergic receptor
(B) Conversion of testosterone to dihydrotestosterone
(C) Conversion of testosterone to estradiol
(D) Inhibition of the α_1-adrenergic receptor
(E) Production of prostate-specific antigen

25. A 19-year-old man who is in the US Army is brought to the emergency department 45 minutes after he sustained a knife wound to the right side of his chest during an altercation. He has no history of major medical illness and takes no medications. His temperature is 36.9°C (98.4°F), pulse is 110/min, respirations are 24/min, and blood pressure is 114/76 mm Hg. Pulse oximetry on room air shows an oxygen saturation of 94%. On physical examination, the trachea appears to be shifted to the left. Pulmonary examination of the right chest is most likely to show which of the following findings?

	**Fremitus**	**Percussion**	**Breath Sounds**
(A)	Decreased	dull	decreased
(B)	Decreased	hyperresonant	decreased
(C)	Decreased	hyperresonant	dull
(D)	Increased	dull	bronchial
(E)	Increased	dull	decreased

---

26. A 34-year-old man comes to the office because of a 1-month history of diarrhea. He has a history of pheochromocytoma treated 2 years ago. His mother is being treated for a tumor of her parathyroid gland. He has no other history of major medical illness and takes no medications. His temperature is 37.0°C (98.6°F), pulse is 84/min, respirations are 10/min, and blood pressure is 120/75 mm Hg. Pulse oximetry on room air shows an oxygen saturation of 97%. Vital signs are within normal limits. Physical examination shows a 3-cm, palpable mass on the right side of the neck. A biopsy specimen of the mass shows a neuroendocrine neoplasm of parafollicular cell origin. The most likely cause of the findings in this patient is a mutation in which of the following types of genes?

(A) Cell cycle regulation gene
(B) DNA mismatch repair gene
(C) Metastasis suppressor gene
(D) Proto-oncogene
(E) Tumor suppressor gene

27. A 56-year-old man is brought to the emergency department by his wife 30 minutes after he had severe upper back pain and hoarseness before becoming comatose. He has hypertension treated with hydrochlorothiazide. His temperature is 37°C (98.6°F), pulse is 100/min, and blood pressure is 160/80 mm Hg in the right arm and 100/60 mm Hg in the left arm. Ophthalmologic examination shows ptosis and miosis of the left eye. There is anhidrosis of the left side of the forehead, right hemiplegia, and a decreased left radial pulse. A chest x-ray shows a widened mediastinum. Which of the following conditions is the most likely cause of these findings?

(A) Dissection of the aorta distal to the left subclavian artery
(B) Dissection of the aorta extending into the left carotid artery
(C) Dissection of the aorta extending into the left carotid artery and distal aortic arch
(D) Dissection of the proximal aorta extending into the right subclavian artery
(E) Superior sulcus tumor
(F) Thrombus of the left carotid artery

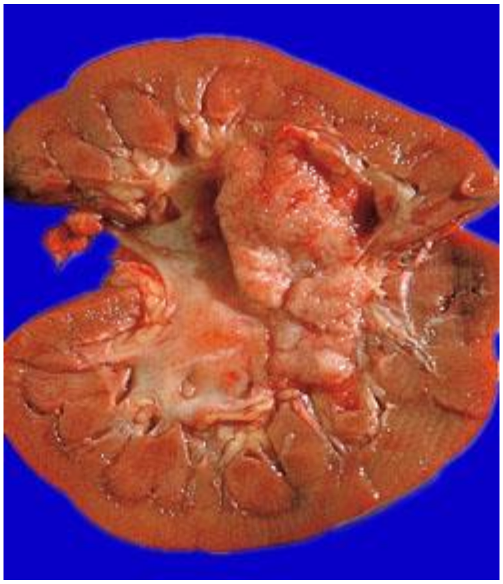

28. A 68-year-old man with alcohol use disorder comes to the office because of a 3-month history of intermittent blood in his urine; he has had no pain. He is a retired laboratory technician from a company that produces naphthylamine. He has smoked 1½ packs of cigarettes daily for 45 years. A CT scan of the abdomen shows a mass in the pelvis of the left kidney. A photograph of the surgically resected kidney is shown. The neoplastic process in this kidney is most likely to be which of the following?

(A) Angiomyolipoma
(B) Metastatic melanoma
(C) Nephroblastoma
(D) Oncocytoma
(E) Urothelial carcinoma

---

29. A 27-year-old woman comes to the emergency department because of a 1-hour history of severe shortness of breath. She has just returned from a cross-country flight. She has a history of borderline hypertension. Her temperature is 36.9°C (98.5°F), pulse is 113/min, respirations are 28/min, and blood pressure is 138/85 mm Hg. Physical examination shows that the right calf has an increased circumference compared with the left calf, and there is tenderness behind the right knee. Which of the following is the most likely underlying cause of this patient's condition?

(A) Antithrombin III deficiency
(B) Factor V Leiden mutation
(C) Glanzmann thrombasthenia
(D) Protein C deficiency
(E) von Willebrand disease

30. A 20-year-old woman is brought to the urgent care center because of a 2-month history of progressive weakness of her arms. She also has a 1-week history of moderate back pain and headache. Her only medication is ibuprofen as needed for pain. Muscle strength is 3/5 in the upper extremities. Sensation to pinprick is decreased over the upper extremities. MRI of the spine shows a central syrinx in the cervical spinal cord. It is most appropriate to obtain specific additional history regarding which of the following in this patient?

(A) Diet
(B) Family illness
(C) Recent travel
(D) Trauma
(E) Unintended weight loss

31. A 3-year-old boy is brought to the office because of a 2-day history of bulging of his left eye. He says his eye hurts. He has no history of major medical illness or recent trauma to the area, and he receives no medications. Vital signs are within normal limits. Physical examination shows exophthalmos of the left eye. MRI of the brain shows a 2-cm mass involving the ocular muscles of the left eye. A biopsy specimen of the mass shows malignant cells, some of which have striations. Which of the following is the most likely diagnosis?

(A) Neuroblastoma
(B) Pheochromocytoma
(C) Retinoblastoma
(D) Rhabdomyosarcoma
(E) Thyroid cancer

32. An 18-year-old woman with sickle cell disease is brought to the emergency department by her parents because of a 2-hour history of severe abdominal pain and nausea. Her parents say that she had a cheeseburger, milk shake, and chocolate bar for lunch. Her temperature is 37°C (98.6°F). Physical examination shows tenderness over the right upper quadrant of the abdomen, radiating to the right shoulder. Ultrasonography of the right upper quadrant of the abdomen shows gallstones. Which of the following is the most likely underlying cause of this patient's current condition?

(A) Decreased hepatic secretion of lecithin
(B) Decreased reabsorption of bile salts
(C) High ratio of cholesterol to bile acids in bile
(D) Infestation with parasites secreting β-glucuronidase
(E) Overload of unconjugated bilirubin

33. In a sample of 100 individuals, the mean leukocyte count is $7500/mm^3$, with a standard deviation of $1000/mm^3$. If the leukocyte counts in this population follow a normal (gaussian) distribution, approximately 50% of individuals will have which of the following total leukocyte counts?

(A) $5500–9500/mm^3$
(B) $<6500/mm^3$ or $>8500/mm^3$
(C) $6500–8500/mm^3$
(D) $<7500/mm^3$
(E) $>9500/mm^3$

34. A previously healthy 52-year-old woman comes to the physician because of a 2-month history of fatigue, constipation, and frequent urination. Her temperature is 37.1°C (98.8°F), pulse is 80/min, respirations are 14/min, and blood pressure is 140/90 mm Hg. Diffuse crackles are heard bilaterally. Her serum calcium concentration is 11.1 mg/dL, and serum parathyroid hormone concentration is decreased. A chest x-ray shows bilateral hilar lymphadenopathy and interstitial infiltrates. Which of the following is the most likely cause of this patient's hypercalcemia?

(A) Calcitriol production by activated macrophages
(B) Local resorption of bone by metastases
(C) Parathyroid hormone-related peptide secretion
(D) Secretion of parathyroid hormone
(E) Secretion of thyroid-stimulating hormone

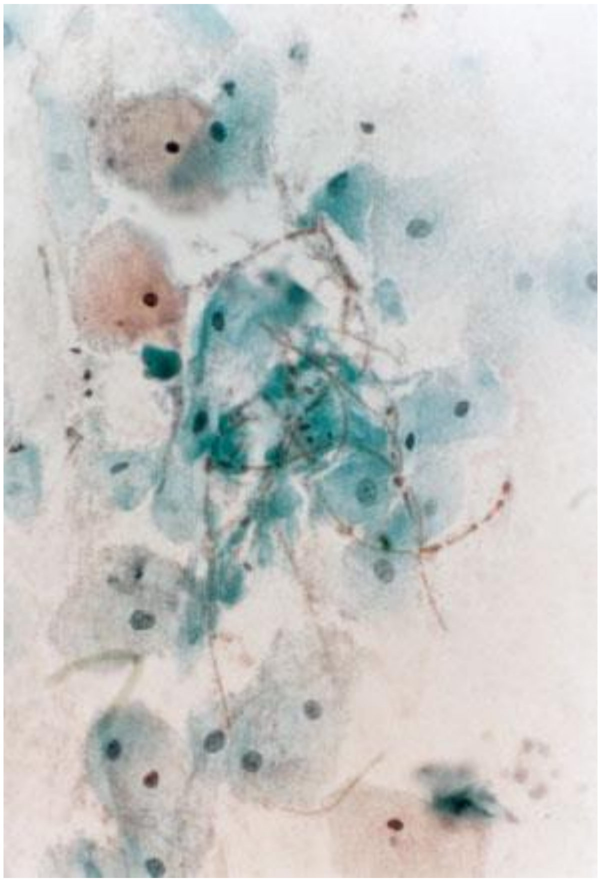

35. A 55-year-old woman comes to the clinic because of a 2-month history of increasingly severe vaginal pain and itching during sexual intercourse. She avoids intercourse with her husband because of the symptoms. She has been in a monogamous relationship with her husband for the past 25 years. She has type 2 diabetes mellitus. Her vital signs are within normal limits. Pelvic examination shows edematous and erythematous vaginal mucosa with white discharge. A photomicrograph of a vaginal smear is shown. Which of the following is the most likely causal infectious agent?

(A) *Candida albicans*
(B) *Chlamydia trachomatis*
(C) Herpes simplex virus
(D) Human papillomavirus
(E) *Trichomonas vaginalis*

---

36. A 24-year-old woman is brought to the physician 1 month after she was involved in a motor vehicle collision that left her weak and unable to walk. Physical examination shows weakness of both hands and atrophy of the intrinsic hand muscles bilaterally. There is weakness and increased muscle tone of the lower extremities on passive range of motion. Deep tendon reflexes are normal at the biceps and triceps bilaterally and are increased at the knees and ankles. Babinski sign is present bilaterally. Sensation to pinprick is absent at and below the level of the clavicles. The lesion in this patient is most likely located at which of the following spinal cord levels?

(A) C5
(B) C7
(C) T1
(D) T3
(E) T5

37. A 45-year-old man is brought to the emergency department 30 minutes after the sudden onset of crushing chest pain. His father, maternal aunt, and paternal uncle all died of myocardial infarctions under the age of 50 years. Physical examination shows tendinous xanthomas on the hands and thickened Achilles tendons. Serum lipid studies show a total cholesterol concentration of 410 mg/dL, HDL-cholesterol concentration of 30 mg/dL, and triglyceride concentration of 140 mg/dL. The diagnosis of myocardial infarction is made. This patient most likely has a deficiency of which of the following?

(A) Apo B48
(B) Apo C
(C) HMG-CoA reductase activity
(D) LDL receptor
(E) Lipoprotein lipase activity

38. A previously healthy 19-year-old man is brought to the emergency department 30 minutes after he collapsed while playing softball. He had severe, sharp, upper back pain prior to the game. He is 196 cm (6 ft 5 in) tall. His temperature is 37°C (98.6°F), pulse is 130/min, respirations are 24/min, and blood pressure is 80/50 mm Hg. Physical examination shows pallor and no jugular venous distention. Breath sounds are clear. The carotid pulses are weak. A grade 4/6, late diastolic murmur is heard at the lower left sternal border. Which of the following is the most likely cause of this patient's cardiac findings?

(A) Atrial septal defect
(B) Mitral stenosis
(C) Papillary muscle rupture
(D) Perforated tricuspid valve
(E) Stretched aortic anulus

39. A 27-year-old woman is brought to the emergency department because of a 2-week history of double vision. Neurologic examination shows that the left eye does not adduct past the midline on horizontal gaze when the patient looks to the right. Leftward horizontal gaze is normal. This patient's ocular movement deficit is most likely caused by damage to which of the following structures?

(A) Left abducens nerve
(B) Left medial longitudinal fasciculus
(C) Left nucleus of the abducens nerve
(D) Right abducens nerve
(E) Right medial longitudinal fasciculus
(F) Right nucleus of the abducens nerve

40. A 45-year-old woman is recovering from acute respiratory distress syndrome secondary to gallstone pancreatitis. A drawing of the alveolar wall is shown. Which of the following labeled cells will most likely proliferate and reestablish the injured epithelial layer in this patient?

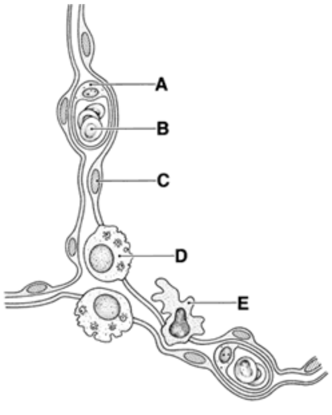

USMLE STEP 1 SAMPLE TEST QUESTIONS

BLOCK 2, ITEMS 41-80

41. An 11-year-old girl is brought to the emergency department by her parents because of a 1-week history of breast enlargement. She has not had pain or nipple discharge. She has asthma treated with inhaled albuterol as needed. She does not smoke cigarettes, drink alcoholic beverages, or use illicit drugs. She is at the 50th percentile for height and weight. Vital signs are within normal limits. Examination of the breasts shows minimal enlargement under the areolae and mild enlargement of the diameter of the areolae without nipple discharge; no masses are palpated. There is scant pubic hair. Which of the following best describes the sexual maturity rating for this patient?

(A) 1
(B) 2
(C) 3
(D) 4
(E) 5

42. A 5-year-old boy is brought to the emergency department by his mother because of an episode of bloody stool 3 hours ago. The mother says the stool was hard "like pebbles" and she noted bright red blood on the tissue when the patient cleaned himself. His previous bowel movement was 5 days ago. The patient has no abdominal or rectal pain now, but he did have abdominal pain during his bowel movement 5 days ago. He has no history of major medical illness and receives no medications. Vaccinations are up-to-date. The patient has no recent history of travel. He is at the 5th percentile for height and the 10th percentile for weight; BMI is at the 50th percentile. Vital signs are within normal limits. Abdominal examination shows hypoactive bowel sounds and a soft, slightly distended abdomen that is not tender to palpation. Rectal examination shows 1 cm of bright red rectal mucosa protruding from the right side of the anus; there is no rectal bleeding. The remainder of the examination shows no abnormalities. Which of the following is the most likely cause of this patient's physical findings?

(A) Constipation
(B) Cystic fibrosis
(C) Hirschsprung disease
(D) Hookworm infestation
(E) Intussusception

43. A 25-year-old woman comes to the office because of a 3-day history of fever, chills, severe headache, weakness, muscle pain, loss of appetite, vomiting, diarrhea, and moderate abdominal pain. She is in nursing school and returned from a medical missions trip in West Africa 10 days ago. Her symptoms began abruptly while she was shopping in a supermarket after her return. Temperature is 39.0°C (102.2°F), pulse is 100/min, respirations are 22/min, and blood pressure is 110/70 mm Hg. The patient appears ill and in mild respiratory distress. Physical examination discloses poor skin turgor and hyperactive bowel sounds. Muscle strength is 4/5 throughout. Laboratory studies show leukopenia and thrombocytopenia. Which of the following is the most sensitive and specific test for detection of the suspected viral genome in this patient?

(A) Microarray analysis
(B) Northern blot
(C) Reverse transcription-polymerase chain reaction test
(D) Southern blot
(E) Western blot

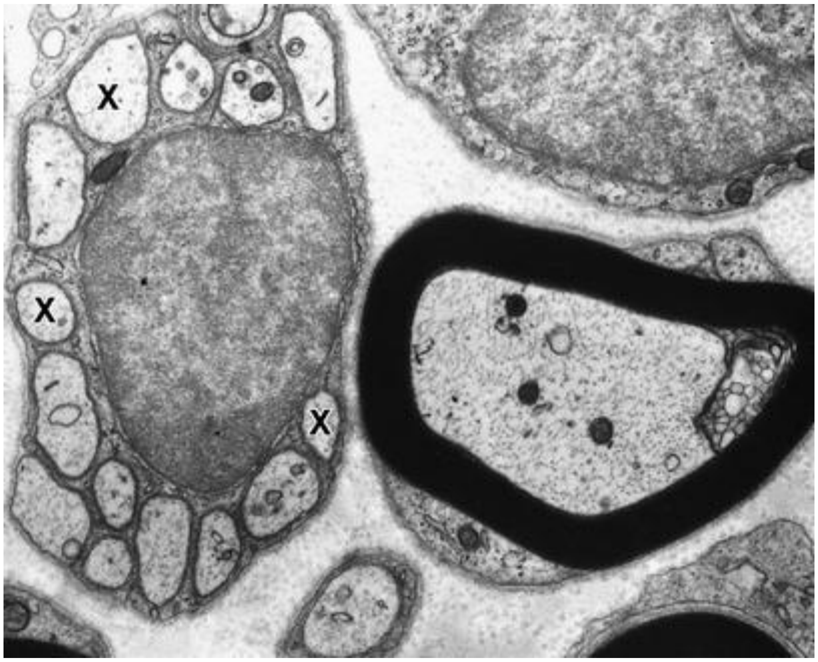

44. A 56-year-old man comes to the office because of a 1-month history of pain and tingling of his hands and a 6-month history of paresthesia of his feet. As part of the workup, a nerve biopsy specimen is obtained and analyzed at the electron microscopic level. The biopsy specimen shows marked loss of structures labeled by the X's in the photomicrograph of a normal biopsy specimen shown. The loss of these structures is most likely to cause which of the following neurologic findings in this patient?

(A) Decreased sensitivity of the deep tendon reflex
(B) Impaired stereognosis
(C) Loss of pain and temperature sensation
(D) Muscle atrophy
(E) Vasoconstriction

---

45. A 5-month-old boy is brought to the clinic by his mother because of a 10-day history of “coughing spells” that occur several times daily and last 1 to 2 minutes; he often vomits afterwards. He was delivered at term to a 16-year-old patient, gravida 1, para 1, following an uncomplicated pregnancy and spontaneous vaginal delivery in Mexico. His parents immigrated to the USA shortly after his birth. He has no history of major medical illness and receives no medications. He has never been to a physician for a well-child examination and has not received any vaccinations. He appears well. Vital signs, including oxygen saturation, are within normal limits. During the physical examination, he coughs uncontrollably for 2 minutes, after which there is a gasping sound and subsequent vomiting. Afterwards, he appears exhausted. Physical examination shows no nasal flaring or intercostal or subcostal retractions. The lungs are clear; no wheezes or crackles are heard. A drug from which of the following classes is most appropriate for this patient?

(A) Cephalosporin
(B) Fluoroquinolone
(C) Macrolide
(D) Penicillin
(E) Sulfonamide

46. A 40-year-old woman comes to the physician because of a runny nose, sneezing, and itching eyes on exposure to cats since childhood. Antiallergy drugs have not provided relief. Her symptoms improve with a program of desensitization involving administration of increasing doses of the allergen. Which of the following is the most likely mechanism of the beneficial effect of this treatment?

(A) Formation of dimers that stimulate clearance by macrophage phagocytosis
(B) Increased production of IgG antibodies that block allergen binding to mast cells
(C) Overwhelming IgE binding sites to result in inactivation of mast cells
(D) Production of an anti-idiotypic antibody to IgE that results in its clearance
(E) Stimulation of macrophages to produce anti-inflammatory cytokines

47. A 3-week-old girl delivered at term with no complications is brought to the physician by her mother because of a 1-week history of yellow eyes and skin, tan-colored stools, and dark brown urine. The newborn has been breast-feeding without difficulty. She is alert and appears to be in no distress. She is at the 50th percentile for length and weight. Physical examination shows scleral icterus and jaundice. There is mild hepatomegaly; the spleen is not palpable. Laboratory studies show:

Hemoglobin	14.4 g/dL
Hematocrit	43%
Leukocyte count	$8000/mm^3$
Serum	
Albumin	3.5 g/dL
Bilirubin, total	14 mg/dL
Direct	12.5 mg/dL
AST	50 U/L
ALT	45 U/L

Which of the following is the most likely diagnosis?

(A) Biliary atresia
(B) Crigler-Najjar syndrome, type I
(C) Gilbert syndrome
(D) Hemolytic disease of the newborn
(E) Physiologic jaundice

---

48. A 65-year-old woman comes to the physician for a follow-up examination after blood pressure measurements were 175/105 mm Hg and 185/110 mm Hg 1 and 3 weeks ago, respectively. She has well-controlled type 2 diabetes mellitus. Her blood pressure now is 175/110 mm Hg. Physical examination shows no other abnormalities. Antihypertensive therapy is started, but her blood pressure remains elevated at her next visit 3 weeks later. Laboratory studies show increased plasma renin activity; the erythrocyte sedimentation rate and serum electrolytes are within the reference ranges. Angiography shows a high-grade stenosis of the proximal right renal artery; the left renal artery appears normal. Which of the following is the most likely diagnosis?

(A) Atherosclerosis
(B) Congenital renal artery hypoplasia
(C) Fibromuscular dysplasia
(D) Takayasu arteritis
(E) Temporal arteritis

49. A 46-year-old woman comes to the physician because of a 1-month history of fatigue, weakness, and palpitations. She has advanced kidney disease as a result of continued use of combination analgesics for low back pain. She has hypertension well controlled with a loop diuretic, a β-adrenergic blocker, and a dihydropyridine calcium channel blocker. She has a history of gastritis and hemorrhoids associated with occult blood in the stools. She is in no acute distress. Her temperature is 36.8°C (98.2°F), pulse is 74/min, respirations are 14/min, and blood pressure is 150/86 mm Hg. Physical examination shows mild midepigastric tenderness to palpation and 2+ edema of the lower extremities. Cardiac examination shows an S_4 gallop. Test of the stool for occult blood is positive. Laboratory studies show:

Hemoglobin	8.8 g/dL
Hematocrit	26.8%
Mean corpuscular volume	82 μm^3
Serum	
Ferritin	262 ng/mL
Folate	284 ng/mL (N=150–450)
Total iron	60 µg/dL
Transferrin saturation	22% (N=20%–50%)
Lactate dehydrogenase	62 U/L

Which of the following is the most likely cause of this patient's anemia?

(A) Bone marrow suppression
(B) Decreased erythropoietin production
(C) Intravascular hemolysis
(D) Iron deficiency anemia
(E) Splenic sequestration

---

50. A 46-year-old woman comes to the physician because of a 3-day history of intermittent pain with urination and increased urinary frequency. She says that she had one similar episode during the past 6 months. She also has had irregular menses, and her last menstrual period occurred 2 months ago. She has not had fever, nausea, vomiting, or blood in her urine. She is sexually active with one male partner. Physical examination shows no abnormalities. Urinalysis shows:

RBC	3–5/hpf
WBC	10–20/hpf
Nitrites	positive
Leukocyte esterase	positive
Bacteria	positive

Which of the following is the strongest predisposing risk factor for the development of this patient's condition?

(A) Leiomyomata uteri
(B) Perimenopause
(C) Pregnancy
(D) Sexual intercourse

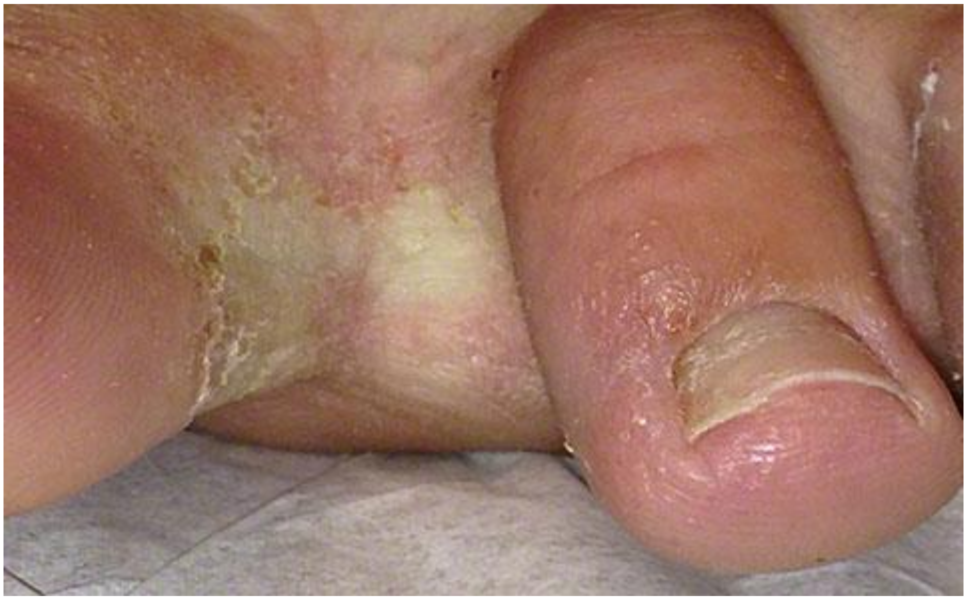

51. A 37-year-old man comes to the office because of a 3-week history of itchy patches of skin and a burning sensation in his feet. He works as a dishwasher and says he frequently works in wet shoes and socks. Vital signs are within normal limits. Physical examination shows the findings in the photograph; these findings are also present on the soles of both feet. The most appropriate pharmacotherapy for this patient will inhibit the activity of which of the following enzymes?

(A) Chitin synthase
(B) 1,3-β-D-Glucan synthase complex
(C) Phospholipase D
(D) Squalene monooxygenase
(E) Thymidylate synthase

---

52. A 10-year-old boy is brought to the emergency department in the middle of summer because of fever, headache, and photophobia. Several of his camp mates have had a similar illness. Physical examination shows mild nuchal rigidity. A lumbar puncture is performed. Laboratory studies show:

Serum glucose	90 mg/dL
Cerebrospinal fluid	
Pressure, opening	50 mm H_2O
Glucose	65 mg/dL
Total protein	70 mg/dL
Leukocyte count	43/mm^3 (95% lymphocytes)

Which of the following infectious agents is the most likely cause of these findings?

(A) Adenovirus
(B) Enterovirus
(C) Herpes simplex virus
(D) *Neisseria meningitidis*
(E) *Streptococcus pneumoniae*

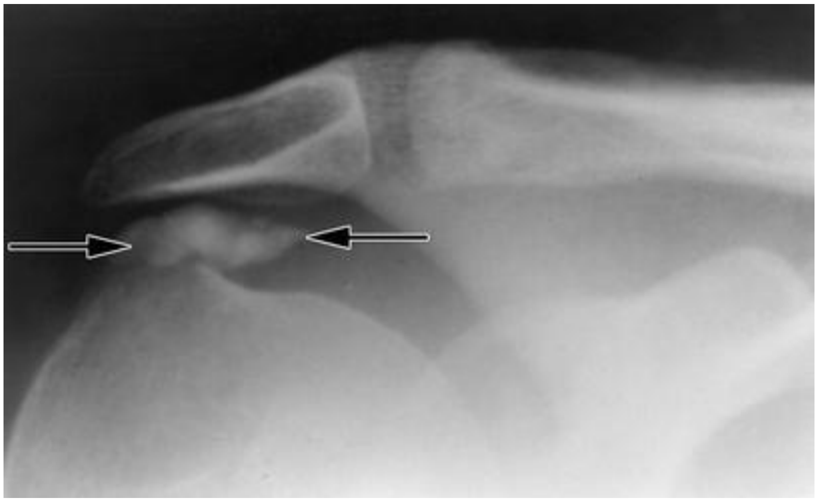

53. A 55-year-old woman comes to the physician because of a 3-week history of right shoulder pain that occurs when she is lying on her right shoulder in bed. There is tenderness to palpation over the greater tubercle. An x-ray of the right shoulder is shown; the arrows indicate a calcium deposit. Further examination of this patient will most likely show impairment of which of the following active shoulder movements?

(A) Abduction
(B) Adduction
(C) External rotation
(D) Flexion
(E) Internal rotation

---

54. A 31-year-old woman with a 5-year history of fatigue comes to the physician for an initial visit. She has seen four other physicians for the same condition within the past 6 months, but no abnormalities were found. She gives the physician a large folder that contains her medical records. She says, "I can barely get out of bed most mornings, but nobody can figure out why because all of my tests turn out normal. I really think I have chronic fatigue syndrome. What do you know about this condition?" The physician has not treated any patient with chronic fatigue syndrome before. Which of the following initial statements by the physician is most appropriate to establish rapport with this patient?

(A) "From the size of the folder you brought, it looks like you've had very thorough examinations in the past."
(B) "I don't have much experience with chronic fatigue syndrome, but I'm committed to learning as much as I can about it."
(C) "I'm not familiar with chronic fatigue syndrome, except that many physicians don't think it's a real disease."
(D) "Let's start over from scratch. We'll need to repeat complete testing."
(E) "When nothing abnormal is found during thorough examinations and laboratory studies, there is often an underlying psychological cause of the symptoms."

55. A 28-year-old man comes to the physician because of a 2-month history of a rash on his wrists and hands. He is a first-year mortuary science student. He also works on his grandfather's farm each weekend. His hobbies include raising homing pigeons and repairing vintage motorcycles. He recently changed to a different type of laundry detergent to save money. Physical examination shows a diffuse erythematous rash involving both hands up to the wrist creases. The rash does not extend over any other parts of the body. Which of the following is the most likely cause of this patient's rash?

(A) Change in laundry detergent
(B) Exposure to chemicals during motorcycle repair
(C) Handling pigeons
(D) Pesticide exposure
(E) Use of latex gloves

56. A 22-year-old woman at 32 weeks' gestation comes to the office for a prenatal visit. Vital signs are within normal limits. Fundal height and fetal heart tones are also normal. At the end of the examination, the patient tells the physician that her friend is a certified lay midwife and has recently convinced her to have a home birth. The patient asks the physician if he would be the backup for the midwife in case of an emergency. Which of the following is the most appropriate initial response by the physician?

(A) "I'm sorry, but I would no longer be able to be your doctor if you pursue a home birth."
(B) "I would like to meet with your friend before I decide."
(C) "If there's a problem, I could still act as your doctor if you arrive at the hospital in labor."
(D) "Let's set up an appointment next week to discuss your birth plan in detail."
(E) "Perhaps your midwife could act as your birth coach instead."

57. A 36-year-old woman with hypertension comes to the office because she thinks she may be "going through early menopause." She has not had a menstrual period since her most recent office visit 6 months ago. During this time, she also has been "gaining weight around the middle" despite increased exercise; she has had a 6.3-kg (14-lb) weight gain. She has no other history of major medical illness. Her only medication is lisinopril. She does not smoke, drink alcohol, or use illicit drugs. She is 168 cm (5 ft 6 in) tall and weighs 107 kg (236 lb); BMI is 38 kg/m^2. Vital signs are within normal limits. Examination shows a uterus consistent in size with a 24-week gestation. Pelvic ultrasonography shows oligohydramnios and a fetus with a misshapen cranium, pericardial effusion, small bladder, and echogenic bowel. The most likely cause of the fetal abnormalities in this patient's pregnancy is interference with which of the following?

(A) Fetal lung/epithelial differentiation
(B) Fetal lung/surfactant development
(C) Fetal renal hemodynamics
(D) Maternal placental perfusion
(E) Maternal prostaglandin synthesis

58. A 67-year-old man comes to the office because he is concerned about memory loss. He says he sometimes forgets the names of acquaintances he sees while he is out shopping. He also has occasional word-finding difficulty and forgets to buy some items when he goes shopping unless he makes a list. He lives alone and is able to manage his finances, cook, and shop without help. He works part-time as an accountant. He has gastroesophageal reflux disease and hypertension. Current medications are hydrochlorothiazide and omeprazole. Vital signs are within normal limits. Physical and neurologic examinations show no abnormalities. On mental status examination, he is fully oriented. His speech is normal, and thoughts are organized. His mood is euthymic, and he has a full range of affect. His concentration is intact, and he is able to perform calculations quickly and accurately. He can name objects accurately and follow written and verbal commands. He recalls three of four objects after 5 minutes. Which of the following is the most appropriate physician response to this patient's concern?

(A) "I am concerned about your memory loss. Let's discuss how to further evaluate your memory."
(B) "There's no need to worry right now, but let's meet again in 6 months."
(C) "Unfortunately, your memory loss will likely increase significantly during the next 5 years; let's discuss some ways to plan for the future."
(D) "Your episodes of forgetfulness are likely just 'Senior Moments,' but we should obtain in-depth laboratory test results and an MRI to be certain."
(E) "Your examination findings indicate that your memory loss is likely consistent with the normal aging process."

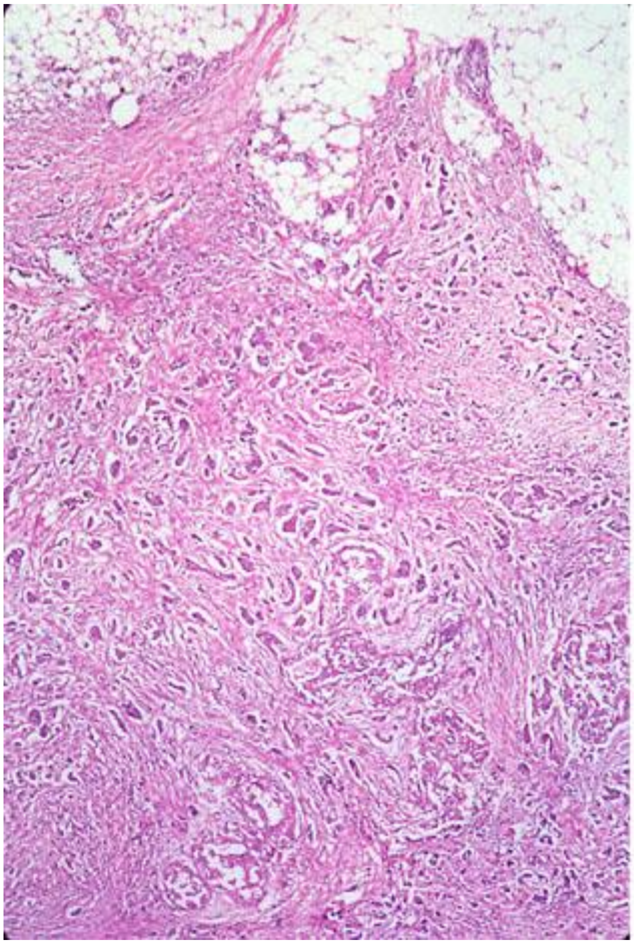
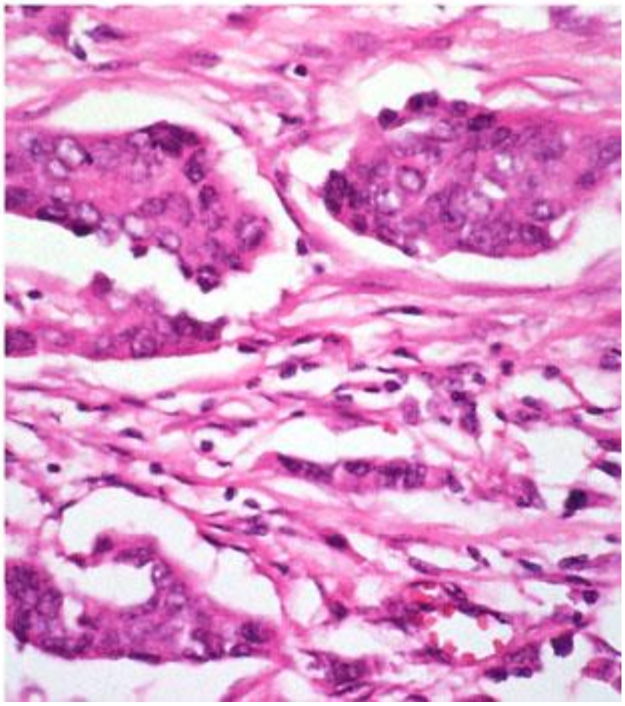

59. A 42-year-old woman comes to the office because of a 1-year history of a left breast mass. She had been living in another country for the past 2 years; a physician in that country told her that the lesion was a cyst. Since returning to the USA 12 weeks ago, she has noticed that the mass has increased in size and is now so tender that any physical contact is painful. She has not had any other breast masses, skin changes, or nipple changes. She has a history of cysts in both breasts, and has undergone aspirations of 2 prior cysts; pathologic examination both times showed no abnormalities. She has a 1-year history of yellow nipple discharge from both breasts and premenstrual bilateral breast pain. She has no other history of serious illness and takes no medications. There is no family history of breast cancer. Vital signs are within normal limits. Physical examination shows a 3-cm, firm, extremely tender and partially mobile mass in the upper outer quadrant of the left breast. Mammography shows a 3.1-cm, irregular mass in the left breast corresponding to the palpable area of the mass. Photomicrographs of an excisional biopsy specimen are shown. Which of the following pathologic findings most strongly suggests malignancy in this patient?

(A) Compressed ducts
(B) Dense fibrosis
(C) Expanded ducts
(D) Multiple large and small cysts
(E) Pleomorphic irregular glands

---

60. A 30-year-old woman comes to the office because of a 4-day history of an increasingly severe, painful rash over her body and in her mouth. The rash began over her trunk area but spread within a day to her face and extremities. Two days before development of the rash, she had flu-like symptoms with muscle aches and fatigue as well as a nonproductive cough, sore throat, and runny nose. Ten days ago, she began treatment with trimethoprim-sulfamethoxazole for a urinary tract infection; she takes no other medications. Temperature is 39.0°C (102.2°F), pulse is 120/min, respirations are 25/min, and blood pressure is 165/105 mm Hg. Physical examination shows diffuse brownish red macular exanthema with bullous lesions. Epidermis at an uninvolved site can be removed with mild tangential pressure. Examination of a

biopsy specimen of one of the lesions shows necrosis of keratinocytes throughout the epidermis. There is minimal lymphocytic infiltration within the superficial dermis. Which of the following is the most likely diagnosis?

(A) Erythema multiforme
(B) Linear IgA bullous dermatosis
(C) Pemphigus vulgaris
(D) Staphylococcal scalded skin syndrome
(E) Toxic epidermal necrolysis

61. A 78-year-old woman is admitted to the hospital because of a 1-week history of jaundice. CT scan of the abdomen shows a mass suggestive of pancreatic cancer. Three hours later, the on-call physician enters the patient's room to discuss the prognosis and obtain consent for a biopsy scheduled for the next morning. On entering the room, the physician greets the patient and her husband. The physician then learns that the patient speaks only Mandarin. Her husband is fluent in Mandarin and English. The hospital interpreter is not available until tomorrow morning. The patient's husband appears anxious and insists that the physician speaks to him and allows him to serve as an interpreter for his wife. Which of the following is the most appropriate next step in management?

(A) Allow the patient's husband to serve as an interpreter
(B) Consult the hospital ethics committee
(C) Explain to the husband that information cannot be provided until the hospital interpreter arrives in the morning
(D) Use a telephone interpreter service

62. Results of a study that examined the impact of risk factors on cardiovascular health are being evaluated. In the study, serum LDL-cholesterol (LDL) concentration is found to have a correlation of 0.6 with serum high-sensitivity C-reactive protein (hs-CRP) concentration. Which of the following statements best describes the relationship between LDL concentration and hs-CRP concentration based on this finding?

(A) Higher LDL concentrations are associated with higher hs-CRP concentrations
(B) Higher LDL concentrations are associated with lower hs-CRP concentrations
(C) Higher LDL concentrations cause higher hs-CRP concentrations
(D) Higher LDL concentrations cause lower hs-CRP concentrations

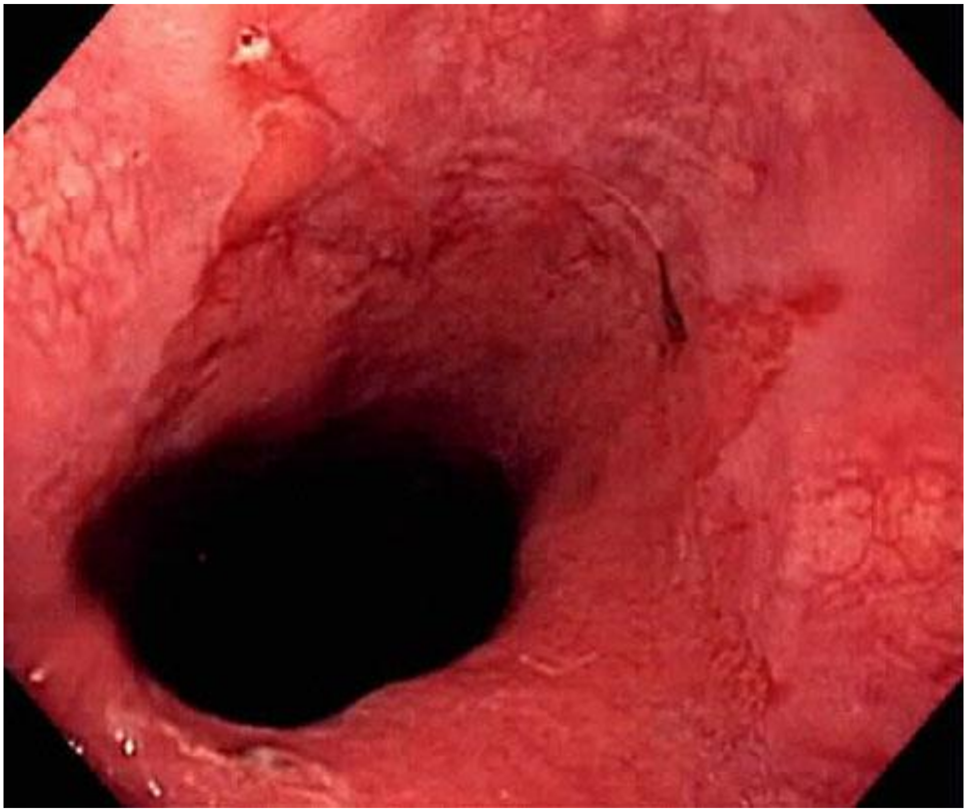

63. A 45-year-old man comes to the physician because of a 10-year history of heartburn that occurs after he eats late at night or consumes spicy food. He has had no weight loss or difficulty swallowing. He takes over-the-counter antacids as needed, but they relieve his discomfort only temporarily. Physical examination shows no abnormalities. An endoscopy is done. The distal esophagus is shown in the photograph. Which of the following is the most likely cause of this patient's symptoms?

(A) Defect in secretin production
(B) Excessive gastrin production
(C) Excessive transient lower esophageal relaxations
(D) Failure of primary esophageal peristalsis
(E) Failure of saliva production

---

64. A 72-year-old man comes to the physician for a health maintenance examination. He has no personal or family history of major medical illnesses. He has never smoked cigarettes. Pulse oximetry on room air shows an oxygen saturation of 98%. Physical examination shows plethoric skin and splenomegaly. Laboratory studies show:

Hemoglobin	21.1 g/dL
Hematocrit	61%
Leukocyte count	15,000/mm^3
Segmented neutrophils	68%
Basophils	4%
Lymphocytes	28%
Platelet count	501,000/mm^3
Leukocyte alkaline phosphatase	increased

A peripheral blood smear shows occasional giant platelets. The primary hematologic defect in this patient most likely occurred in which of the following cells?

(A) Erythroid progenitor
(B) Hematopoietic stem
(C) Lymphatic progenitor
(D) Megakaryocyte progenitor
(E) Pluripotent stem

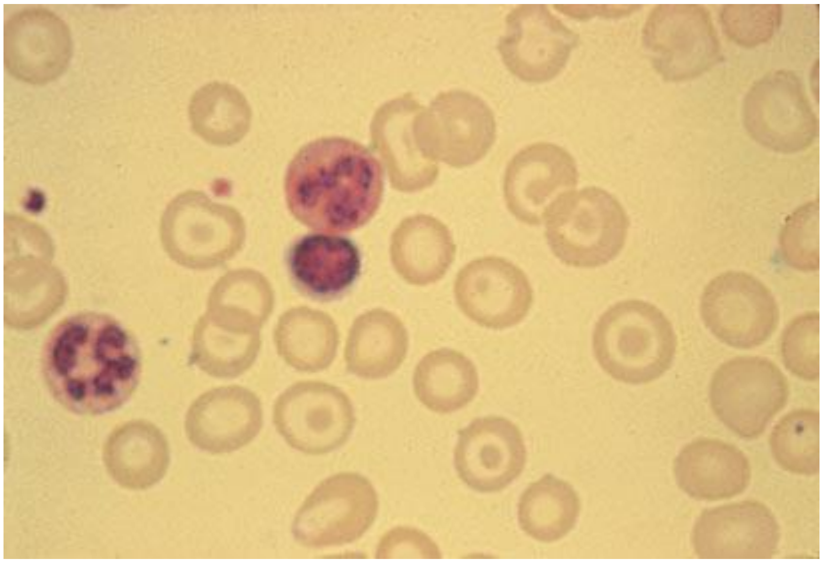

65. A 35-year-old man is brought to the emergency department 30 minutes after he sustained a cut on his hand while loading cargo at his job. He lives alone and takes most of his meals at a local restaurant. He eats mostly snack foods at the bar and fast food. He drinks four to six 12-oz beers daily and double that amount on weekends. He takes no medications. Physical examination shows a 3-cm laceration on the right hand that is bleeding steadily. Laboratory studies show a hemoglobin concentration of 11 g/dL, leukocyte count of $4000/mm^3$, and platelet count of $150,000/mm^3$. A photomicrograph of a peripheral blood smear is shown. A deficiency of which of the following is the most likely cause of this patient's anemia?

(A) Folic acid
(B) Glucose-6-phosphate dehydrogenase
(C) Iron
(D) Vitamin B_1 (thiamine)
(E) Vitamin B_6 (pyridoxine)

---

66. A 3800-g (8-lb 6-oz) newborn is delivered vaginally at 39 weeks' gestation after an uncomplicated pregnancy. Apgar scores are 9 and 9 at 1 and 5 minutes, respectively. The newborn is crying, has pink skin, and appears vigorous. Physical examination shows a vagina and also a structure that appears to be a penis, with the urethra located at the base near the opening of the vagina. Chromosomal analysis is ordered. Which of the following is the most likely cause of the intersex findings in this newborn if the karyotype is found to be 46,XX?

(A) 17α-Hydroxyprogesterone deficiency
(B) Increased concentration of müllerian-inhibiting substance
(C) Maternal androgen exposure
(D) Presence of the sex-determining region Y gene
(E) 5α-Reductase deficiency

67. An 80-year-old woman is brought to the emergency department (ED) 30 minutes after she fell out of her wheelchair at home. This is the second visit to the ED for a fall during the past 3 months. She lives with her daughter and son-in-law, who say they "do the best we can." The patient has dementia, Alzheimer type, coronary artery disease, type 2 diabetes mellitus, and hypertension. Current medications are amlodipine, aspirin, atorvastatin, donepezil, long-acting insulin, and lisinopril. Five years ago, she underwent bilateral below-the-knee amputations because of infected ulcers of the feet. She uses a wheelchair for ambulation. Ten years ago, she underwent three-vessel coronary artery bypass grafting. She has smoked one-half pack of cigarettes daily for 60 years. She drinks one shot of whiskey nightly. She is thin and appears ill and disheveled. Her temperature is 37.2°C (99.0°F), pulse is 80/min, respirations are 20/min, and blood pressure is 120/80 mm Hg. Pulse oximetry on 2 L/min of oxygen by nasal cannula shows an oxygen saturation of 95%. Physical examination shows temporal wasting. There are scattered ecchymoses over the abdomen and all extremities. No other abnormalities are noted. Which of the following is the most appropriate initial history to obtain from this patient?

(A) Abnormal bleeding
(B) Diet
(C) Relationship with her family
(D) Respiratory symptoms
(E) Urinary symptoms

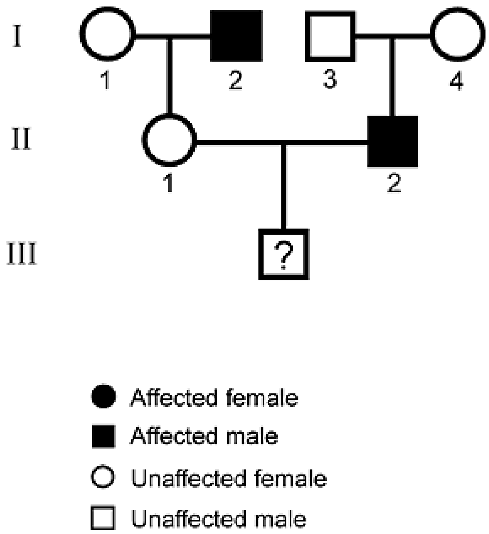

68. A healthy 26-year-old man with red-green color blindness marries a woman whose father also has red-green color blindness. A pedigree is shown. Which of the following best predicts the risk that a son of this couple will be affected?

(A) 0%
(B) 25%
(C) 50%
(D) 75%
(E) 100%

---

69. A 25-year-old woman comes to the physician because of a long history of pain with menses. The pain occurs on the first day of her 5-day menstrual period and lasts all day. She rates the pain as 10 on a 10-point scale. The most appropriate initial pharmacotherapy to relieve this patient's pain has which of the following mechanisms of action?

(A) Inhibition of estrogen synthesis
(B) Inhibition of 11β-hydroxylase activity
(C) Inhibition of prostaglandin synthesis
(D) Stimulation of follicle-stimulating hormone synthesis
(E) Stimulation of luteinizing hormone synthesis

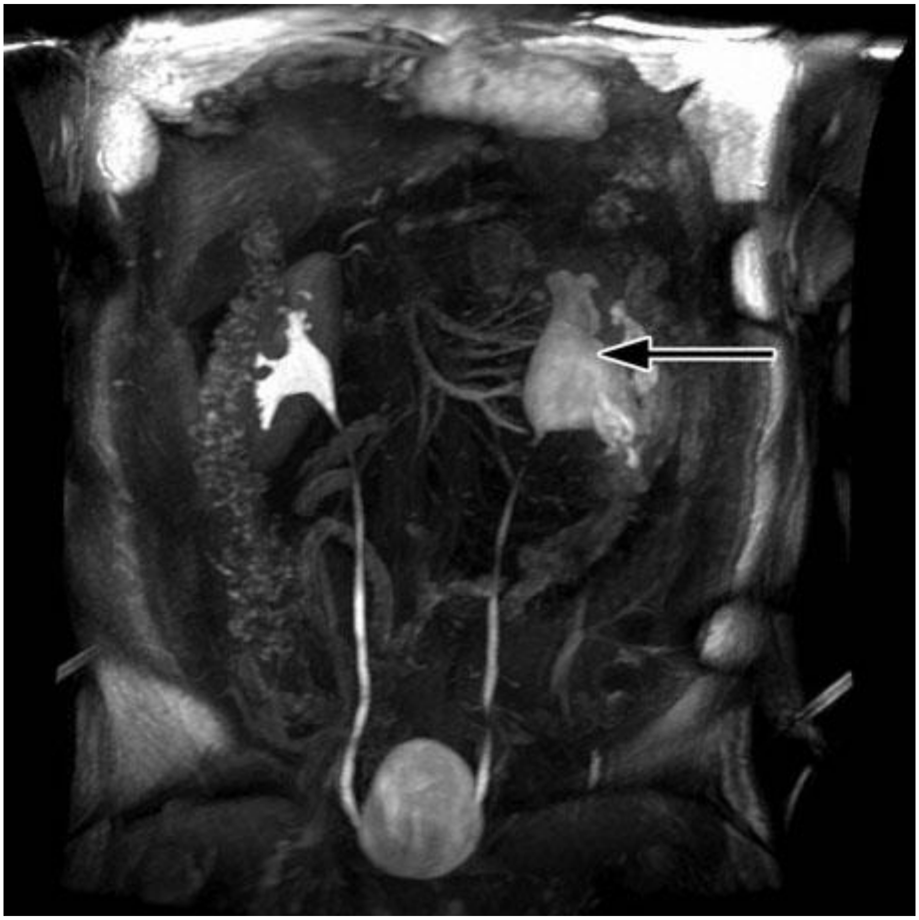

70. A 56-year-old woman comes to the physician because of a 2-year history of recurrent urinary tract infections accompanied by left flank pain. Physical examination shows no abnormalities. Renal ultrasonography shows left-sided hydronephrosis. A T2-weighted coronal MRI of the abdomen is shown; the arrow indicates the hydronephrosis. The left renal collecting system is most likely obstructed at which of the following anatomic locations in this patient?

(A) Bladder neck
(B) Mid ureter
(C) Renal calyx
(D) Ureteropelvic junction
(E) Ureterovesical junction

---

71. A randomized clinical trial is conducted to compare wound healing and cosmetic differences between two surgical procedures for closing skin wounds following cesarean delivery. A total of 1000 women undergoing cesarean delivery during a 6-month period are enrolled in the study, which was 85% of the total number of patients undergoing the procedure. The results show a wound infection rate of 12 cases per 1000 women for Procedure A and 18 cases per 1000 women for Procedure B. Which of the following is the best estimate of the absolute risk reduction for wound infection following Procedure A compared with Procedure B?

(A) (18/1000) – (12/1000)
(B) 0.85 × (12/1000)
(C) 0.85 × (18/1000)
(D) [0.85 × (18/1000–12/1000)]
(E) [(1.2/100) – (1.8/100)]/(1.8/100)

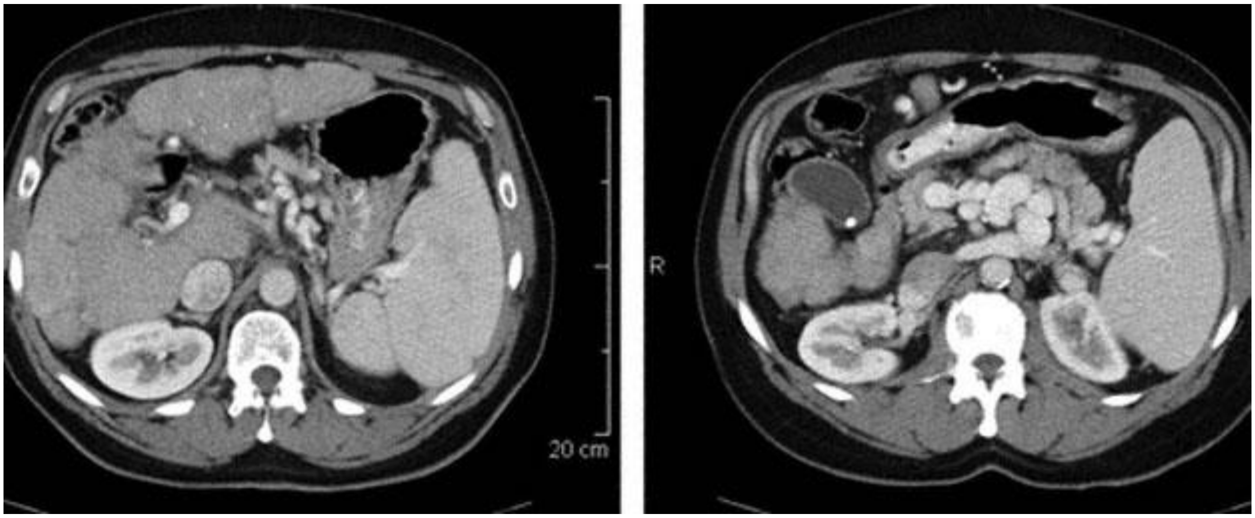

72. A 52-year-old man is admitted to the hospital because of a 2-hour history of vomiting bright red blood. His pulse is 125/min, and blood pressure is 90/60 mm Hg. Physical examination shows jaundice and visible blood vessels surrounding the umbilicus. CT scans of the abdomen are shown. To decrease portal venous pressure in this patient, it is most appropriate to place a shunt between the portal vein and which of the following additional vessels?

(A) Inferior mesenteric vein
(B) Inferior vena cava
(C) Left gastric vein
(D) Splenic vein
(E) Superior mesenteric vein

---

73. A 58-year-old man with chronic obstructive pulmonary disease comes to the clinic with his wife for a follow-up examination. He has smoked one pack of cigarettes daily for 35 years. He has tried to quit smoking twice but was unsuccessful both times. At today's visit, when the physician asks the patient about smoking cessation, he says he is not ready to do so. The patient's wife states her husband's smoking makes her cough and gives her chest tightness. Which of the following is the most appropriate physician statement?

(A) "Are there any reasons why you might want to quit smoking?"
(B) "Are you aware that your lung condition is chronic at this point?"
(C) "I'm sure you don't want your wife to suffer as a result of your smoking."
(D) "The majority of your health issues would improve if you quit smoking."
(E) "Why haven't you been able to stay off cigarettes?"

74. Seven days after admission to the psychiatric unit for treatment of eating disorders, a hospitalized 20-year-old woman has a 2-day history of moderate mouth pain on the inside of both cheeks. She also reports feeling "worthless and fat." Fluoxetine was started on admission. She appears distressed. She is 170 cm (5 ft 7 in) tall and weighs 77 kg (170 lb); BMI is 27 kg/m^2. Vital signs are within normal limits. Physical examination shows bilateral swelling of the parotid and submandibular glands, discoloration of several teeth, and scarring on the dorsum of the right hand. Mental status examination shows no suicidal ideation or intent. Results of laboratory studies are within the reference ranges. A medication with which of the following mechanisms of action is most appropriate to treat this patient's current symptoms?

(A) Binding to muscarinic acetylcholine receptors
(B) Binding to nicotinic acetylcholine receptors
(C) Inhibition of protein synthesis via binding to 50S ribosomal subunits and preventing peptide bond formation
(D) Inhibition of protein synthesis via loss of DNA helical structure
(E) Interference with bacterial wall synthesis

75. A 35-year-old woman, gravida 2, para 1, at 15 weeks' gestation is brought to the hospital in active labor. The physician care team confers and determines that the birth cannot be prevented and that the fetus will not survive. After explaining this fact to the patient, the patient says, "I know it is a long shot, but I still want my baby resuscitated and everything done to save him." After empathizing with the patient, which of the following is the most appropriate initial response by the physician?

(A) "I will ask for a second opinion."
(B) "I will call the father of the baby to get his opinion as well."
(C) "I will call for a medical ethics consultation."
(D) "I will do everything I can to save the baby."
(E) "I'm very sorry, but the baby will not survive."

76. A 48-year-old man who is a contractor interpreter working for the military at a US outpost in Afghanistan comes to the medical clinic because of a 20-day history of nonhealing, painless ulcers on his neck and arms. The lesions enlarged over time and began to express clear fluid, eventually forming shallow ulcers. When the symptoms began, he was sleeping on a mattress on the floor in an old building without air conditioning where the ambient temperature ranged from 21.1°C (70.0°F) to 43.3°C (110.0°F). He originally attributed the lesions to bug bites. Vital signs are within normal limits. Physical examination shows six 2-cm, papular lesions scattered over the neck and upper extremities; each lesion has a 0.6-cm ulcer in the center. There is no pus or exudate. Which of the following vectors is the most likely source of the lesions in this patient?

(A) Flea
(B) Mosquito
(C) Sand fly
(D) Spider
(E) Tick

77. A 17-year-old boy is brought to the clinic for a follow-up examination. He has been evaluated for three episodes of full-body weakness at the ages of 13, 16, and 17 years. Each episode occurred when he lay down after playing in a football scrimmage. The weakness improved spontaneously during the next 6 hours; he was asymptomatic by the time he was evaluated by medical personnel. The patient attributes the episodes to eating "a lot of pasta and salty foods" prior to playing football. Results of a complete blood count and comprehensive metabolic profile following each episode have been within the reference ranges. He has no history of serious illness and takes no medications. Vital signs are within normal limits. Physical and neurologic examinations disclose no abnormalities. Which of the following serum concentrations is most likely to be abnormal if measured during one of this patient's episodes?

(A) Calcium
(B) Chloride
(C) Magnesium
(D) Potassium
(E) Sodium

78. A 3-day-old female newborn is brought to the hospital because of a yellowish, milky fluid leaking from both nipples. She was delivered at term to a 20-year-old woman. Pregnancy and delivery were uncomplicated. Examination of the newborn is otherwise unremarkable. Which of the following hormones is the most likely cause of this finding?

(A) Maternal estrogen
(B) Maternal progesterone
(C) Maternal prolactin
(D) Newborn estrogen
(E) Newborn progesterone
(F) Newborn prolactin

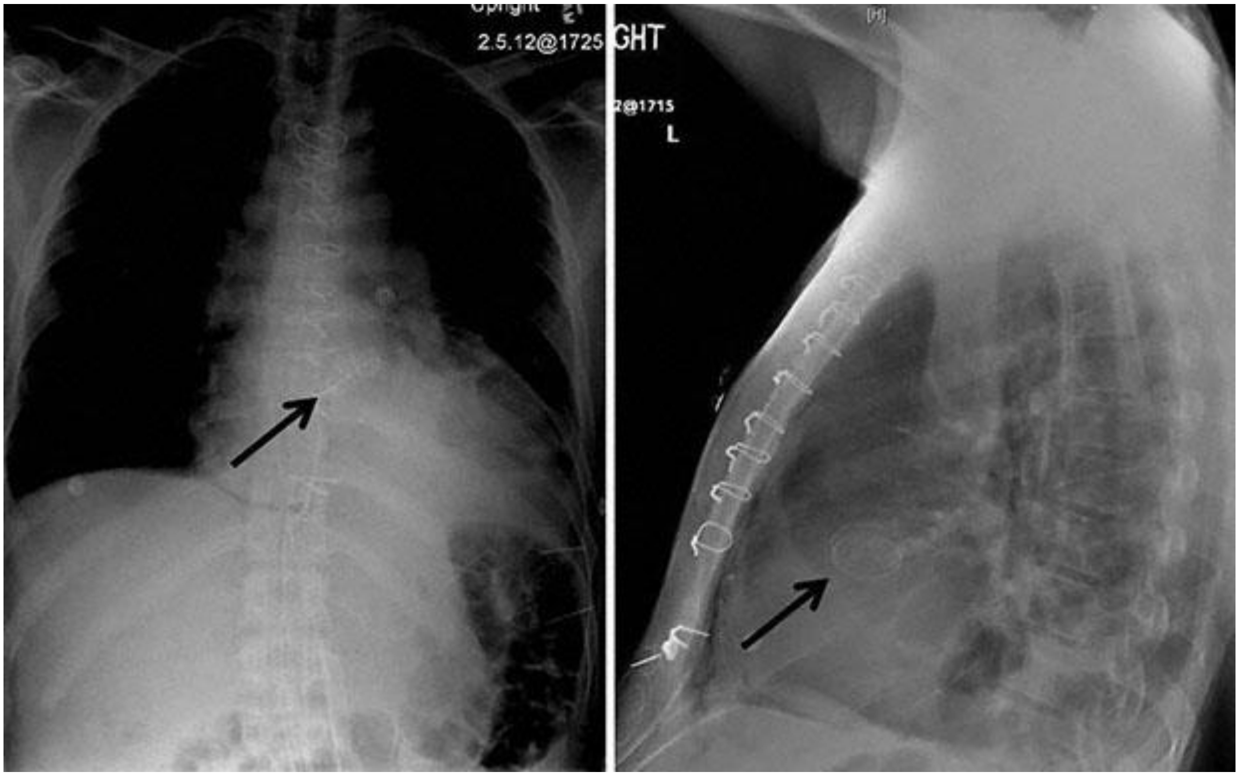

79. A 74-year-old man comes to the office for a follow-up examination 1 month after he was discharged from the hospital following a cardiac valve replacement operation. Physical examination shows a healing median sternotomy wound. A prosthetic click is heard. PA and lateral chest x-rays are shown; the arrows indicate the replaced cardiac valve. Based on these findings, which of the following cardiac valves was most likely replaced in this patient?

(A) Aortic
(B) Mitral
(C) Pulmonic
(D) Tricuspid

---

80. A 37-year-old man who is a carpenter is brought to the emergency department 45 minutes after the sudden onset of fever, shortness of breath, and palpitations. Four days ago, he sustained a puncture wound to his left hand; he treated the wound with antibacterial cream and a bandage. His temperature is 39°C (102.2°F), pulse is 120/min, respirations are 28/min, and blood pressure is 100/60 mm Hg. Examination of the left hand shows diffuse swelling, erythema, and a 2-cm, necrotic puncture wound. His leukocyte count is 14,000/mm^3. Arterial blood gas analysis on room air shows a P_{CO_2} of less than 32 mm Hg. Which of the following is the most likely infectious agent in this patient?

(A) *Clostridium tetani*
(B) *Mycobacterium abscessus*
(C) *Pasteurella multocida*
(D) *Pseudomonas aeruginosa*
(E) *Staphylococcus aureus*

USMLE STEP 1 SAMPLE TEST QUESTIONS

BLOCK 3, ITEMS 81-119

81. A 45-year-old woman comes to the office for a follow-up examination 2 weeks after she sustained a vertebral fracture at L1. The fracture occurred spontaneously and there is no history of trauma to the area or other fractures. She gained 27 kg (60 lb) during the 6 months before the fracture occurred. Her only medication is hydromorphone as needed for pain. She is 163 cm (5 ft 4 in) tall and now weighs 100 kg (220 lb); BMI is 38 kg/m^2. Temperature is 37.0°C (98.6°F), pulse is 86/min, respirations are 12/min, and blood pressure is 145/98 mm Hg. Physical examination shows central obesity and purple striae over the abdomen bilaterally. The lower extremities appear thin. Results of laboratory studies are shown:

Plasma	
Renin activity	5.0 ng/mL/h (N=0.6–4.0)
Metanephrine	0.3 nmol/L (N<0.4)
Serum	
Cortisol, random	43 µg/dL
Adrenocorticotropic hormone	120 pg/mL (N<120)
Aldosterone	8 ng/dL (N=2–9)
Urine 24-hour free cortisol excretion	340 µg/24 h (N=3.5–45)

The most likely cause of the fracture in this patient is an increase in which of the following processes?

(A) Calcium absorption
(B) Calcium excretion
(C) Osteoblast proliferation
(D) Osteoclast proliferation
(E) Phosphorus absorption
(F) Phosphorus excretion

---

82. A 25-year-old woman comes to the emergency department because of a 3-hour history of fever, severe headache, light-headedness, dizziness, shaking chills, and muscle aches. Five hours ago, she was diagnosed with Lyme disease and began doxycycline therapy. She has no other history of serious illness and takes no other medications. Menses occur at regular 28-day intervals. She is currently menstruating and using a tampon. She appears anxious. Temperature is 37.0°C (98.6°F), pulse is 120/min, respirations are 30/min, and blood pressure is 90/60 mm Hg. Pulse oximetry on room air shows an oxygen saturation of 94%. Physical examination shows flushing and diaphoresis. Cardiopulmonary examination shows no other abnormalities. Which of the following is the most likely mechanism of this patient’s current condition?

(A) Exacerbation of infection by *Borrelia burgdorferi*
(B) Infection-mediated sepsis
(C) IgE-mediated allergic reaction to doxycycline
(D) Release of bacterial products producing acute inflammation
(E) Secretion of bacterial endotoxins

83. A newborn delivered at 36 weeks' gestation to a 22-year-old woman, gravida 1, para 1, has difficulty feeding and listlessness. The mother received no prenatal care. Spontaneous vaginal delivery was uncomplicated. The mother's only medication was a prenatal vitamin. The newborn's length is 49 cm (19 in; 39th percentile), and weight is 3100 g (6 lb 13 oz; 30th percentile); head circumference is 33 cm (13 in; 12th percentile). Temperature is 37.0°C (98.6°F), pulse is 134/min, respirations are 38/min, and blood pressure is 73/50 mm Hg. Physical examination shows ambiguous genitalia.

Results of serum studies are shown:

Na^+	133 mEq/L
K^+	5.0 mEq/L (N=3.2–5.5)
Cl^-	103 mEq/L
HCO_3^-	17 mEq/L
Glucose	42 mg/dL (N=30–60)

The most appropriate pharmacotherapy for this patient targets which of the following receptors?

(A) Adrenocorticotropic hormone
(B) Aldosterone
(C) Androgen
(D) Gonadotropin-releasing hormone
(E) Growth hormone

---

84. A 60-year-old woman is brought to the emergency department because of a 4-day history of fever, joint aches, and rash. Three weeks ago, she was admitted to the hospital for treatment of *Staphylococcal aureus* endocarditis. She has received 21 days out of a prescribed 42-day course of intravenous oxacillin. Currently, she appears to be in mild distress. Temperature is 38.0°C (100.4°F), pulse is 115/min, respirations are 24/min, and blood pressure is 120/70 mm Hg. Pulse oximetry on room air shows an oxygen saturation of 97%. Physical examination shows a diffuse maculopapular rash over the trunk and upper and lower extremities. There is no pus or erythema at the skin insertion site of the peripherally inserted central catheter line initially placed on the day of hospital discharge. Results of laboratory studies are shown:

Hemoglobin	11.1 g/dL
Hematocrit	33%
Leukocyte count	$12{,}100/mm^3$
Segmented neutrophils	78%
Eosinophils	9%
Lymphocytes	7%
Monocytes	6%
Platelet count	$341{,}000/mm^3$
Serum	
Na^+	133 mEq/L
K^+	6.5 mEq/L
Cl^-	100 mEq/L
HCO_3^-	15 mEq/L
Urea nitrogen	65 mg/dL
Glucose	96 mg/dL
Creatinine	5.7 mg/dL

Urine microscopy shows eosinophils and WBC casts. Which of the following is the most likely underlying cause of this patient's condition?

(A) Collapsing focal segmental glomerulosclerosis
(B) Glomerular hypertrophy with hemorrhage and necrosis
(C) Interstitial inflammatory infiltrate
(D) Mesangial expansion with glomerular basement membrane thickening
(E) Proximal tubular dilation with loss of brush border

---

85. A 48-year-old man comes to the physician requesting treatment for alcohol withdrawal. He reports a 30-year history of consuming 6 to 10 beers daily. He has had two citations for driving while intoxicated. He has previously experienced alcohol-associated seizures and withdrawal symptoms. His vital signs are within normal limits. Physical examination shows palmar erythema. The most appropriate pharmacotherapy in this patient most likely has which of the following mechanisms?

(A) Blockade of dopamine receptors
(B) Decreased activity of dopamine transporters
(C) Enhancement of the effect of postsynaptic γ-aminobutyric acid (GABA)
(D) Increased GABA transaminase activity
(E) Inhibition of glutamate release
(F) Inhibition of serotonin reuptake
(G) Opening of glutamate channels
(H) Stimulation of 5-hydroxytryptophan receptors

86. A 6-year-old girl is brought to the office because of two episodes of vaginal bleeding during the past 2 months. She has no history of serious illness and receives no medications. She does not appear to be in distress. She is at the 60th percentile for height, 40th percentile for weight, and 35th percentile for BMI. Vital signs are within normal limits. Physical examination discloses palpable breast buds and minimal coarse, pigmented hair on the labia. The remainder of the examination shows no abnormalities. Results of serum studies are shown:

Thyroid-stimulating hormone	2.1 mU/mL (N=0.5–5.0)
Testosterone	680 ng/dL (N=0.17–0.7)
Adrenocorticotropic hormone	18 pg/mL (N=9–52)
Estradiol	185 pg/mL (N<20)
Follicle-stimulating hormone	15 mIU/mL (N<6.7)
Luteinizing hormone	3.0 mIU/mL (N<0.2)

Which of the following is the most likely cause of this patient's condition?

(A) Central activation of neurons
(B) Ectopic prolactin secretion
(C) Exogenous sex steroid secretion
(D) Germline *GNAS* activating mutation
(E) 21-Hydroxylase deficiency

---

87. A previously healthy 64-year-old man is brought to the emergency department 3 hours after the sudden onset of severe flank pain. He has no history of similar pain or serious illness. He takes no medications. He appears to be in distress. His pulse is 100/min, and blood pressure is 168/92 mm Hg. Physical examination shows a soft abdomen with left-sided tenderness. Urinalysis shows microscopic hematuria. A CT scan of the abdomen shows a small ureteric calculus. Analgesic therapy is initiated and the pain resolves 1 hour later. The urine is then strained and a uric acid calculus is found. Which of the following processes is most likely impaired in this patient?

(A) Bile salt metabolism
(B) Cholesterol metabolism
(C) Cytochrome P450 activity
(D) Purine metabolism
(E) Urea cycle

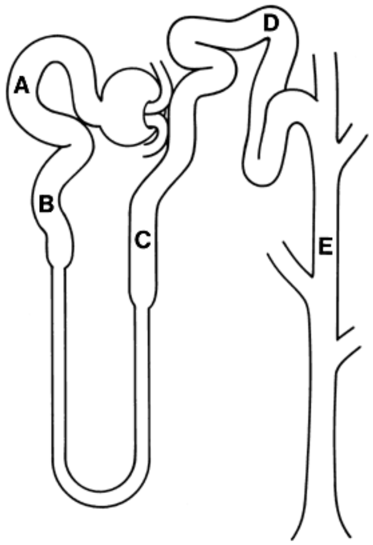

88. A 65-year-old woman has congestive heart failure for which she is treated with an appropriate drug regimen including an ACE inhibitor. Because of insufficient improvement in her symptoms of dyspnea on exertion and edema, the physician prescribes a second agent that results in hyperkalemia. This adverse effect most likely resulted from a drug that targets which of the following labeled locations in the diagram of a nephron?

(A) A
(B) B
(C) C
(D) D
(E) E

---

89. **Patient Information**
Age: 34 years
Gender: M, self-identified
Race/Ethnicity: unspecified
Site of Care: office

The patient presents because of a 2-year history of recurrent abdominal pain and diarrhea; he also has had an 11-kg (25-lb) weight loss during this period. He describes his stools as copious, loose, oily and foul-smelling. His bowel movements occur six to eight times daily, often immediately after meals, and are associated with bloating and cramping. Since the age of 21 years, he has had several episodes of acute pancreatitis. He has not had fevers or bloody stools. He is 178 cm (5 ft 10 in) tall and weighs 57 kg (125 lb); BMI is 18 kg/m^2. Physical examination shows a flat abdomen with hyperactive bowel sounds and diffuse mild tenderness. Digital rectal examination shows no abnormalities. Test of the stool for occult blood is negative. Treatment with which of the following enzymes is most likely to be beneficial in this patient?

(A) Amylase
(B) Lactase
(C) Lipase
(D) Protease
(E) Tryptase

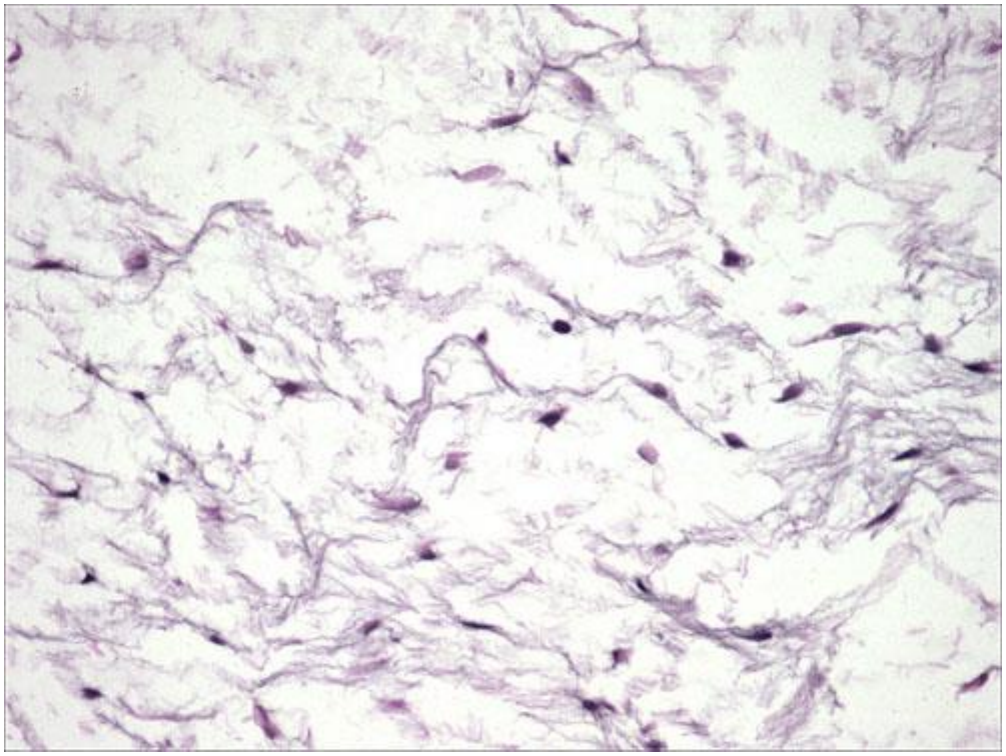

90. A previously healthy 65-year-old woman comes to the physician because of several episodes of fainting during the past 2 months. Each episode lasted several minutes. Her pulse is 82/min, respirations are 20/min, and blood pressure is 135/87 mm Hg. Cardiac examination shows S_3 and S_4. Echocardiography shows a pedunculated intracardiac mass. The lesion is resected. A photomicrograph of the resected lesion is shown. This lesion was most likely obtained from which of the following locations?

(A) Interventricular septum
(B) Left atrium
(C) Left ventricle
(D) Right atrium
(E) Right ventricle

---

91. A 4-year-old girl with type 1 diabetes mellitus is brought to the emergency department by her father because of a 4-hour episode of restlessness, sweating, and confusion that occurred during the night. Yesterday, he allowed her to eat cupcakes and cotton candy at a county fair. At her bedtime that evening, he increased her dose of subcutaneous intermediate-acting and long-acting insulin. Her symptoms began 6 hours later, then resolved spontaneously. After being informed this morning of this nighttime episode, the mother insisted the father bring the patient to the hospital. On arrival, the patient is alert. Her vital signs are within normal limits. Examination shows no abnormalities. Her fingerstick blood glucose concentration is 72 mg/dL. Urinalysis is negative for glucose and ketones. Which of the following is the most likely explanation for this patient's nighttime symptoms?

(A) Hyperglycemia caused by increased glucose consumption
(B) Hyperglycemia caused by increased glycogen metabolism
(C) Hyperglycemia caused by insufficient exogenous insulin
(D) Hypoglycemia caused by excess exogenous insulin
(E) Hypoglycemia caused by excessive renal glucose loss
(F) Hypoglycemia caused by increased glucagon secretion
(G) Nightmare disorder
(H) Sleep terror disorder

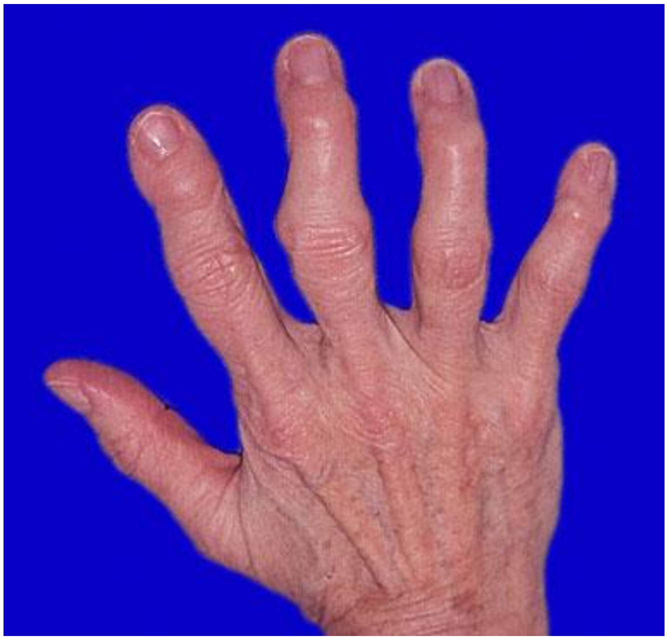

92. A 75-year-old woman comes to the physician because of a 3-year history of pain in her wrists and hands. She says that the pain has become more severe during the past 3 months. She has difficulty buttoning her coat because of the pain. Physical examination shows the findings in the photograph. Which of the following additional hand findings is most likely in this patient?

(A) Cortical thinning
(B) Degenerative changes of the cartilage
(C) Inflammation of digital tendons
(D) Neutrophilic infiltration of the synovium
(E) Thickening of the synovium with pannus

---

93. A 66-year-old man who was recently diagnosed with Parkinson disease comes to the physician for a follow-up examination. Carbidopa-levodopa therapy was initiated at the time of diagnosis. The patient tells the physician that he still has episodes during which he "freezes." He has a clumsy gait, and there is rigidity of his upper extremities and hands. An adjunct therapy designed to inhibit which of the following enzymes is most likely indicated in this patient?

(A) Aromatic L-amino acid decarboxylase
(B) Dopamine β-hydroxylase
(C) Monoamine oxidase B
(D) Phenylethanolamine *N*-methyltransferase
(E) Tyrosine hydroxylase

94. A 2-week-old male newborn is brought to the office for a well-child examination. He was delivered following an uncomplicated, spontaneous vaginal delivery at 41 weeks' gestation. The mother has no history of serious illness and did not receive prenatal care. Her only medication is a prenatal vitamin. She has consumed large amounts of vodka nightly for 10 years. Which of the following examination findings is most likely to be present in this patient?

(A) Hypospadias
(B) Limb hypoplasia
(C) Neck webbing
(D) Short palpebral fissures
(E) Spasticity

95. **Patient Information**

Age: 16 years
Gender: M, self-identified
Race/Ethnicity: Hispanic, self-identified
Site of Care: clinic

History

Reason for Visit/Chief Concern: "I haven't started puberty yet."

History of Present Illness:
• absence of pubic and facial hair
• small penis and testicle size

Past Medical History:
• no serious illness

Medications:
• none

Allergies:
• no known drug allergies

Psychosocial History:
• does not smoke cigarettes, drink alcoholic beverages, or use other substances

Physical Examination

Temp	Pulse	Resp	BP	O_2 Sat	Ht	Wt	BMI
36.0°C (96.8°F)	76/min	13/min	108/61 mm Hg	98% on RA	184 cm (6 ft) 90th %ile	90 kg (198 lb) 97th %ile	27 kg/m^2 94th %ile

• Appearance: long extremities
• Skin: no facial or axillary hair
• HEENT: PERRLA
• Chest: bilateral gynecomastia
• Pulmonary: clear to auscultation
• Cardiac: normal S_1 and S_2
• Abdominal: normoactive bowel sounds; no tenderness to palpation; no hepatosplenomegaly
• Genitourinary: small testicular and penile size; minimal pubic hair; SMR 2
• Neurologic: muscle strength 5/5 throughout

Diagnostic Studies:

Serum	
Follicle-stimulating hormone	45 mIU/mL
Luteinizing hormone	34 mIU/mL
Testosterone	45 ng/dL (N=270–950)
TSH	2.2 µU/L

Question: Which of the following is the most likely underlying cause of this patient's condition?

(A) Constitutional delay in puberty
(B) Hypoplasia of hypothalamic gonadotropin-releasing hormone neurons
(C) Inactivating mutation of the follicle-stimulating hormone-receptor
(D) Mutation in the 21-hydroxylase gene
(E) Nondisjunction of X chromosome during meiosis

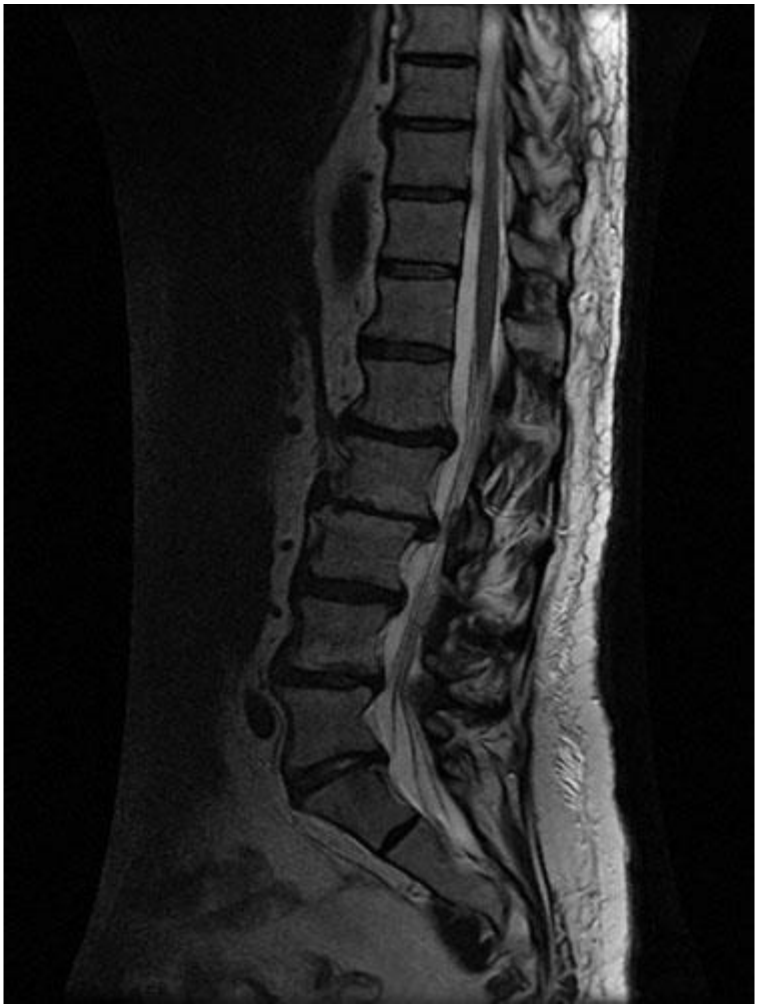

96. A 57-year-old man with chronic low back pain comes to the office for a routine health maintenance examination. The patient's last visit to the office was 2 years ago, and today he says he is "doing about the same," except for an unintentional 10-kg (22-lb) weight gain. He attributes the weight gain to inability to exercise because of his back pain, and he is now considering applying for disability benefits. He was evaluated by a back pain specialist 3 months ago and underwent an MRI of the lumbar spine at that time; however, he says he did not understand the specialist's explanation regarding the MRI results and requests further explanation. Medical history also is remarkable for hyperlipidemia, seasonal allergies, and opioid use disorder; he has not used opioids of any kind since he stopped prescription oxycodone use 5 years ago. Routine medications are atorvastatin, diclofenac, and loratadine. He develops skin flushing when taking niacin, but he has no known drug allergies. He has smoked two packs of cigarettes daily for 25 years. He previously drank two beers daily, but he has not consumed any alcoholic beverages or used any recreational drugs for the past 5 years. He is 178 cm (5 ft 10 in) tall and weighs 104 kg (230 lb); BMI is 33 kg/m^2. Vital signs are within normal limits; blood pressure is 128/70 mm Hg. While standing, the patient leans forward slightly. Range of motion on lumbar extension and flexion is decreased. The remainder of the examination discloses no abnormalities. Results of fasting serum studies are shown:

Na^+	140 mEq/L
K^+	4.7 mEq/L
Cl^-	100 mEq/L
HCO_3^-	24 mEq/L
Urea nitrogen	15 mg/dL
Creatinine	0.7 mg/dL

Cholesterol	
Total	230 mg/dL
HDL	60 mg/dL
LDL	154 mg/dL
Triglycerides	80 mg/dL

MRI of the lumbar spine is shown. Which of the following factors in this patient's history most strongly contributed to the MRI findings?

(A) Alcohol consumption
(B) Cigarette smoking
(C) Diclofenac use
(D) Loratadine use
(E) Opioid use

---

97. A 15-year-old boy is brought to the office by his parents to discuss results of a biopsy done 3 days ago for a rapidly enlarging neck mass. He first noted the mass 1 month ago. Two weeks ago, he was evaluated for an episode of prolonged epistaxis and was found to have a right ear effusion, which was treated with amoxicillin-clavulanic acid. He has no other history of serious illness and takes no medications. Height is at the 10th percentile, weight is at the 50th percentile, and BMI is at the 75th percentile. Vital signs are within normal limits. Physical examination shows a right ear effusion and a 4-cm, firm mass in the right posterior triangle of the neck. Results of a biopsy specimen show squamous epithelium with indistinct cell margins, enlarged atypical nuclei, and absent keratin formation. Which of the following infectious agents is the most likely underlying cause of this patient's current condition?

(A) Cytomegalovirus
(B) Epstein-Barr virus
(C) HIV
(D) Human herpesvirus 8

98. A 48-year-old woman with type 2 diabetes mellitus comes to the physician for a follow-up examination. Current medications are metformin and once-daily insulin. She travels frequently and works long hours. She says that her meals are usually fast food. She leads a sedentary lifestyle. She often forgets to measure her blood glucose concentration. Her last hemoglobin A_{1c} was measured as 8.4%. Which of the following is the most appropriate action by the physician to help this patient improve her diabetic control?

(A) Create an exercise regimen for the patient
(B) Explore barriers to diet adherence
(C) Increase the patient's insulin dosage
(D) Increase the patient's metformin dosage
(E) Measure the patient's blood glucose concentration
(F) Order measurement of the patient's microalbumin concentration
(G) Refer the patient to a nutritionist

99. A 74-year-old woman with a history of coronary artery disease is brought to the emergency department 30 minutes after the sudden onset of crushing chest pain. Her pulse is 120/min, and systolic blood pressure is 70 mm Hg. An ECG shows sinus rhythm with ST elevation in leads V_1 through V_3. The physician plans to administer a drug to increase the patient's blood pressure without increasing her pulse. Which of the following drugs is most appropriate for this patient?

(A) Dopamine
(B) Epinephrine
(C) Isoproterenol
(D) Norepinephrine
(E) Phenylephrine

100. A 2-month-old girl is admitted to the hospital because of severe pneumonia. She has a history of mucocutaneous candidiasis and chronic diarrhea. Her pulse is 160/min, and respirations are 40/min. Crackles are heard over both lung fields. Her leukocyte count is 5400/mm^3 (N=5000–19,500), with 86% segmented neutrophils, 6% lymphocytes, and 8% monocytes. Immunologic testing of a specimen obtained via bronchoscopy is positive for *Pneumocystis jirovecii*. Flow cytometry shows normal concentrations of natural killer cells but no T or B lymphocytes in the peripheral blood. Results of a polymerase chain reaction test of the immunoglobulin V(D)J regions from a bone marrow specimen show an absence of characteristic rearrangements. The most likely cause of these findings is an alteration of which of the following?

(A) Bruton tyrosine kinase
(B) CD40 ligand
(C) Mevalonate kinase
(D) NADPH oxidase
(E) Recombination-activating gene 1 (RAG1) and RAG2

101. A 25-year-old woman, gravida 1, para 1, comes to the office because of a 2-week history of palpitations and heat intolerance. She delivered her child 3 months ago following an uncomplicated pregnancy and delivery. She is breast-feeding. She has no history of serious illness and takes no medications. She is 163 cm (5 ft 4 in) tall and weighs 54 kg (120 lb); BMI is 21 kg/m^2. Temperature is 37°C (98.6°F), pulse is 106/min, respirations are 20/min, and blood pressure is 124/68 mm Hg. Examination shows moist palms and bilateral lid lag. No exophthalmos is noted. The thyroid gland is enlarged and nontender. No murmurs are heard on cardiac examination. Deep tendon reflexes are 3+. Serum studies show an undetectable TSH concentration, thyroxine (T_4) concentration of 20 μg/dL, and triiodothyronine (T_3) concentration of 275 ng/dL. Which of the following is the most likely mechanism of this patient's symptoms?

(A) Activation of mutations of TSH receptors
(B) Increased serum thyroglobulin concentration
(C) Ischemic injury to the hypothalamus
(D) Lymphocytic infiltration of the thyroid
(E) Presence of TSH receptor autoantibodies

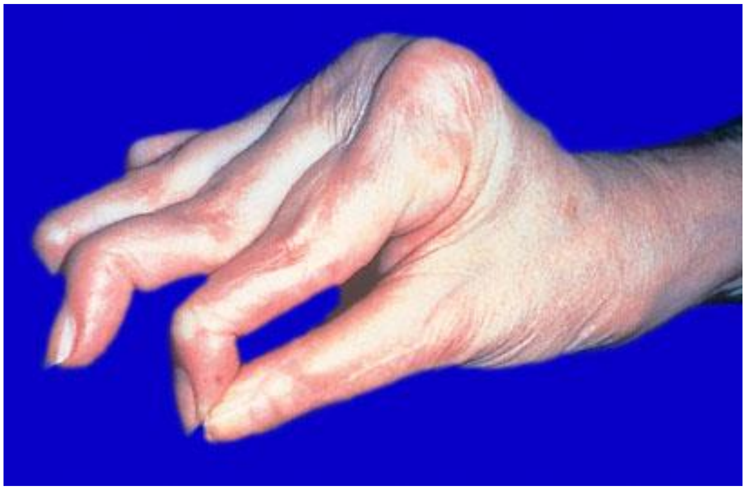

102. A 53-year-old woman comes to the office because of a 6-year history of stiffness and pain of her hands. She has difficulty buttoning her clothes because of the symptoms. She takes no medications. Physical examination shows the findings in the photograph. An abnormality of which of the following is most likely to confirm the diagnosis in this patient?

(A) Anti-citrullinated peptide antibody
(B) Antimitochondrial antibody assay
(C) Human leukocyte antigen-DQ2 antibody assay
(D) Precursor of the erythroid cell line
(E) Precursor of the thrombopoietic line

103. A 73-year-old woman comes to the emergency department because of a 6-hour history of severe abdominal pain. She has chronic atrial fibrillation and underwent placement of a prosthetic mitral valve 4 years ago. Her pulse is 120/min and irregularly irregular, and blood pressure is 80/60 mm Hg. Arteriography shows an embolus in the superior mesenteric artery lodged just beyond the patent middle colic artery. A laparotomy is done. During this procedure, which of the following segments of the abdominal viscera is most likely to appear normal?

(A) Ascending colon
(B) Distal ileum
(C) Distal jejunum
(D) Proximal ileum
(E) Proximal jejunum

104. A 2-year-old boy is brought to the physician because of failure to thrive. He also has had loose, fatty, foul-smelling stools and a cough during the past 2 weeks. He is at the 30th percentile for height and 10th percentile for weight. Physical examination shows no other abnormalities. Laboratory studies show steatorrhea and a sweat chloride concentration of 80 mmol/L. A chest x-ray shows hyperinflation. Sputum culture grows *Haemophilus influenzae* and *Staphylococcus aureus*. Secretion of which of the following substances is most likely to be decreased in this patient?

(A) Bicarbonate
(B) Gastric acid
(C) Glucagon
(D) Insulin
(E) Intrinsic factor

105. A 73-year-old man is brought to the emergency department by his family 1 hour after he walked into the left side of a door frame when leaving his bedroom and then tripped over a chair that was on his left side. Visual field testing shows left lower quadrantanopia. When tactile stimuli are presented on both the left and right sides simultaneously, the patient correctly identifies only the ones on the right. Further examination shows no motor or language deficits. Proprioception is intact. This patient most likely has a brain lesion in which of the following lobes?

(A) Left frontal
(B) Left parietal
(C) Left temporal
(D) Right frontal
(E) Right parietal
(F) Right temporal

106. A 10-year-old boy is brought to the physician because of a 3-week history of nosebleeds and easy bruisability. His older brother has had similar episodes. He is at the 30th percentile for height and weight. Physical examination shows nasal and gingival bleeding and several ecchymoses over the trunk and upper and lower extremities in various stages of healing. Laboratory studies show a platelet count of 300,000/mm^3 (N=150,000–400,000). Platelet adhesion testing shows a normal response to ristocetin, but aggregation does not occur in response to thrombin; platelet morphology is normal. Prothrombin time and activated partial thromboplastin time are within the reference ranges. A defect in which of the following is the most likely cause of the findings in this patient?

(A) Factor VII (proconvertin)
(B) Fibrinogen
(C) Glycoprotein IIb-IIIa
(D) Granule storage pool
(E) von Willebrand factor

107. A previously healthy 45-year-old woman who works as a park ranger comes to the physician because of a 1-week history of shortness of breath, even at rest. She has lived in the mountains at 10,000 feet above sea level for 2 years; the physician's office is located at sea level. Her pulse is 85/min, respirations are 18/min, and blood pressure is 125/90 mm Hg. Physical examination while sitting upright shows jugular venous distention and 2+ pedal edema. During the past 2 years, which of the following has most likely decreased in this patient?

(A) Height of P waves in lead I of the patient's ECG
(B) Height of R waves in lead V_1 of the patient's ECG
(C) Hematocrit
(D) Pulmonary vascular resistance
(E) Right ventricular diastolic compliance
(F) Right ventricular wall thickness

108. A 5-year-old girl is brought to the office by her mother because of a 6-hour history of bloody diarrhea. She is interactive and in no acute distress. Her blood pressure is 90/55 mm Hg. Abdominal examination shows normoactive bowel sounds. Stool cultures are obtained, and the patient's mother is advised to give the girl plenty of fluids. Five days later, the patient develops decreased urine output and is brought back to the office. Her blood pressure is now 135/88 mm Hg. Physical examination shows pallor. Laboratory studies show:

Hemoglobin	8.5 g/dL (N=11–15)
Hematocrit	26% (N=28%–45%)
Platelet count	45,000/mm^3 (N=150,000–400,000)
Serum creatinine	3.3 mg/dL (N=0.3–0.7)

Which of the following infectious agents is the most likely cause of these findings?

(A) *Campylobacter jejuni*
(B) *Escherichia coli*
(C) Rotavirus
(D) *Salmonella enterica* serovar *enteritidis*
(E) *Yersinia pestis*

---

109. A 78-year-old woman is admitted to the intensive care unit because of diverticulitis complicated by *Escherichia coli* sepsis. Treatment with ciprofloxacin is started. Three days later, her serum creatinine concentration has increased from 0.7 mg/dL on admission to 1.3 mg/dL. Urinalysis shows muddy brown casts. The most likely cause of the findings in this patient is ischemia of which of the following structures?

(A) Bowman capsule
(B) Glomerulus
(C) Interstitium
(D) Proximal tubule
(E) Renal vein

110. A 19-year-old man who is a college freshman comes to the office because of a 4-day history of tender, swollen glands. He also has a 6-day history of fever, malaise, and decreased appetite. His temperature is 38.7°C (101.7°F). Physical examination shows swelling of the parotid glands. Which of the following infectious agents is the most likely cause of these findings?

(A) Epstein-Barr virus
(B) Hepatitis B virus
(C) Measles virus
(D) Mumps virus
(E) Rubella virus

111. A 34-year-old woman with myasthenia gravis comes to the emergency department because of a 2-day history of increasing weakness, shortness of breath, and abdominal cramping. Current medications are prednisone and pyridostigmine. Her temperature is 37°C (98.6°F), pulse is 45/min, and respirations are 25/min and shallow. Her voice is soft and hypernasal, and she coughs weakly when swallowing water. Breath and heart sounds are normal. Pulmonary testing shows inability to generate a normal negative inspiratory force during forced inspiration. The abdomen is soft and nontender, with increased bowel sounds. Muscle strength is 4/5 diffusely, with severe, continuous, and diffuse fasciculations. Deep tendon reflexes are sluggish, but symmetric. Which of the following is the most likely cause of this patient's weakness?

(A) Aspiration pneumonia
(B) Guillain-Barré syndrome
(C) Insufficient dose of prednisone
(D) Motor neuron disease
(E) Pyridostigmine overdose

112. A 35-year-old woman comes to the office because she has had three first-trimester spontaneous abortions during the past 3 years. Physical examination shows no abnormalities. Laboratory studies show no endocrine abnormalities. Chromosomal analysis shows a paracentric inversion of the long arm of chromosome 1. Which of the following best describes this patient's risk for early spontaneous abortions and a liveborn child with aneuploidy?

	Risk for Early Spontaneous Abortions	Risk for Liveborn Child With Aneuploidy
(A)	High	high
(B)	High	low
(C)	Low	high
(D)	Low	low

---

113. A 25-year-old man comes to the office because of a 4-hour history of irritability, restlessness, tremor, and palpitations. He is a known user of amphetamines. His pulse is 120/min, respirations are 25/min, and blood pressure is 150/100 mm Hg. Physical examination shows no abnormalities. The most likely cause of this patient's symptoms is sympathomimetic activity arising from which of the following?

(A) Decreased intracellular metabolism of biogenic amines
(B) Decreased monoamine oxidase activity
(C) Decreased presynaptic receptor activation
(D) Increased intracellular metabolism of biogenic amines
(E) Increased presynaptic receptor activation
(F) Increased release of biogenic amines

114. A 14-year-old boy is brought to the office by his mother because of a 1-week history of seizures and difficulty walking. Physical examination shows decreased sensation over the hands and feet and generalized weakness. He walks with an ataxic gait. A muscle biopsy specimen shows coarsely granular fibers that stain red with Gomori trichrome stain. This patient most likely has a genetic defect that most directly affects the synthesis of which of the following?

(A) ATP
(B) Creatine phosphate
(C) Glycogen
(D) NADH
(E) Pyruvate

115. A 72-year-old man is brought to the office because of a 6-month history of increasing fatigue and a 1-month history of numbness of his feet. Physical examination shows marked pallor. Proprioception and sensation to vibration are decreased in the lower extremities. Laboratory studies show:

Hemoglobin	7.6 g/dL
Mean corpuscular volume	117 μm^3
Reticulocyte count	0%
Serum vitamin B_{12} (cobalamin)	23.6 pg/mL (N=200–900)

Histopathology of a gastric mucosal biopsy specimen shows atrophic gastritis with extensive lymphocyte infiltration. Serologic studies will most likely show autoantibodies to which of the following labeled cell types in the photomicrograph of normal mucosa or to one of its secretory products?

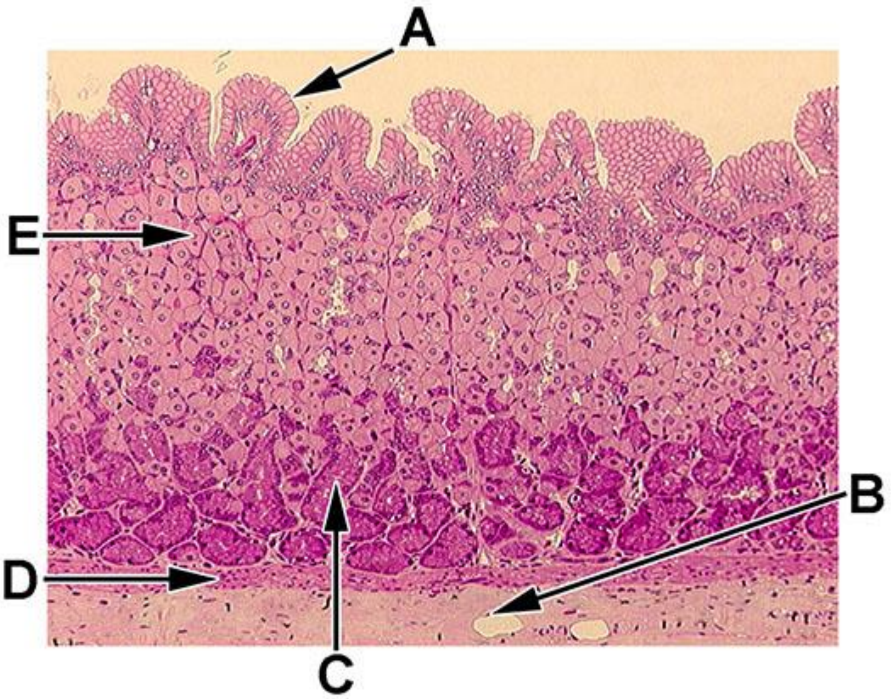

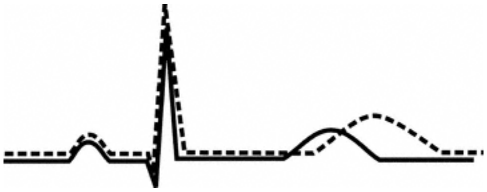

116. A 66-year-old woman comes to the office for a follow-up examination 1 month after being diagnosed with paroxysmal atrial fibrillation. Appropriate pharmacotherapy was initiated at that time, and normal sinus rhythm was restored. Her pulse is 76/min, and blood pressure is 132/86 mm Hg. Physical examination shows no abnormalities. The ECGs shown represent before (solid line) and after (dashed line) administration of the antiarrhythmic drug. Which of the following drugs was most likely prescribed?

(A) Amiodarone
(B) Digoxin
(C) Mexiletine
(D) Phenytoin
(E) Verapamil

117. A 4-year-old boy develops fever 3 weeks after being admitted to the hospital for induction chemotherapy for treatment of acute lymphoblastic leukemia. Chemotherapy medications are L-asparaginase, dexamethasone, doxorubicin, and vincristine. His temperature is 38.2°C (100.8°F), pulse is 122/min, and respirations are 24/min. Physical examination shows pallor, alopecia, and ulcerations over the gums. A central venous catheter with entry site in the right upper chest is present but has no surrounding erythema. A blood culture grows gram-negative rods after 36 hours. Which of the following underlying mechanisms is the most likely cause of this patient's susceptibility to infection?

(A) Deficiency of terminal complement
(B) Hypogammaglobulinemia
(C) Impaired T-lymphocyte function
(D) Inhibition of tumor necrosis factor α function
(E) Neutropenia

118. A 6-year-old boy with acute lymphoblastic leukemia is brought to the office for a follow-up examination. He is receiving high-dose methotrexate therapy. A drug is added to the patient's medication regimen to decrease the toxicity of this therapy to normal cells. The beneficial effect of this new drug on normal cells is most likely achieved by bypassing the cellular requirement for which of the following enzymes?

(A) Dihydrofolate reductase
(B) Methionine synthase
(C) Pyruvate decarboxylase
(D) Thiamine pyrophosphate
(E) Thymidylate synthase

119. A screening test for breast cancer is administered to 1000 women with biopsy-proven breast cancer and to 1000 women without breast cancer. The test results are positive for 250 of the subjects with breast cancer and 100 of the subjects without breast cancer. The screening test is now to be used on a population of 100,000 women with a known prevalence rate of breast cancer of 80 per 100,000. Which of the following is the expected number of false-positives?

(A) 20
(B) 80
(C) 8993
(D) 9992
(E) 10,012

ANSWER FORM FOR USMLE STEP 1 SAMPLE TEST QUESTIONS

Block 1 (Questions 1–40)

1.	___	11.	___	21.	___	31.	___
2.	___	12.	___	22.	___	32.	___
3.	___	13.	___	23.	___	33.	___
4.	___	14.	___	24.	___	34.	___
5.	___	15.	___	25.	___	35.	___
6.	___	16.	___	26.	___	36.	___
7.	___	17.	___	27.	___	37.	___
8.	___	18.	___	28.	___	38.	___
9.	___	19.	___	29.	___	39.	___
10.	___	20.	___	30.	___	40.	___

Block 2 (Questions 41–80)

41.	___	51.	___	61.	___	71.	___
42.	___	52.	___	62.	___	72.	___
43.	___	53.	___	63.	___	73.	___
44.	___	54.	___	64.	___	74.	___
45.	___	55.	___	65.	___	75.	___
46.	___	56.	___	66.	___	76.	___
47.	___	57.	___	67.	___	77.	___
48.	___	58.	___	68.	___	78.	___
49.	___	59.	___	69.	___	79.	___
50.	___	60.	___	70.	___	80.	___

Block 3 (Questions 81–119)

81.	___	91.	___	101.	___	111.	___
82.	___	92.	___	102.	___	112.	___
83.	___	93.	___	103.	___	113.	___
84.	___	94.	___	104.	___	114.	___
85.	___	95.	___	105.	___	115.	___
86.	___	96.	___	106.	___	116.	___
87.	___	97.	___	107.	___	117.	___
88.	___	98.	___	108.	___	118.	___
89.	___	99.	___	109.	___	119.	___
90.	___	100.	___	110.	___		

ANSWER KEY FOR USMLE STEP 1 SAMPLE TEST QUESTIONS

Block 1 (Questions 1–40)

1. A	11. C	21. C	31. D
2. D	12. B	22. A	32. E
3. B	13. F	23. D	33. D
4. A	14. C	24. B	34. A
5. D	15. B	25. B	35. A
6. E	16. B	26. D	36. C
7. C	17. E	27. C	37. D
8. A	18. D	28. E	38. E
9. D	19. B	29. B	39. B
10. A	20. D	30. D	40. D

Block 2 (Questions 41–80)

41. B	51. D	61. D	71. A
42. A	52. B	62. A	72. B
43. C	53. A	63. C	73. A
44. C	54. B	64. B	74. A
45. C	55. E	65. A	75. E
46. B	56. D	66. C	76. C
47. A	57. C	67. C	77. D
48. A	58. E	68. C	78. C
49. B	59. E	69. C	79. A
50. D	60. E	70. D	80. E

Block 3 (Questions 81–119)

81. D	91. D	101. D	111. E
82. D	92. B	102. A	112. B
83. B	93. C	103. E	113. F
84. C	94. D	104. A	114. A
85. C	95. E	105. E	115. E
86. A	96. B	106. C	116. A
87. D	97. B	107. E	117. E
88. E	98. B	108. B	118. A
89. C	99. E	109. D	119. D
90. B	100. E	110. D	
