# Supplementary material for: Benchmarking large language models on the United States medical licensing examination for clinical reasoning and medical licensing scenarios
Source: Sci Rep. 2025 Dec 3;16:1387. doi: 10.1038/s41598-025-31010-4 (PMC12796295; doi:10.1038/s41598-025-31010-4)
Supplement: Supplementary file 2 — Supplementary Information 2. [file 41598_2025_31010_MOESM2_ESM.pdf]

USMLE
United States
Medical
Licensing
Examination®

SAMPLE TEST QUESTIONS

Step 2 Clinical Knowledge (CK)

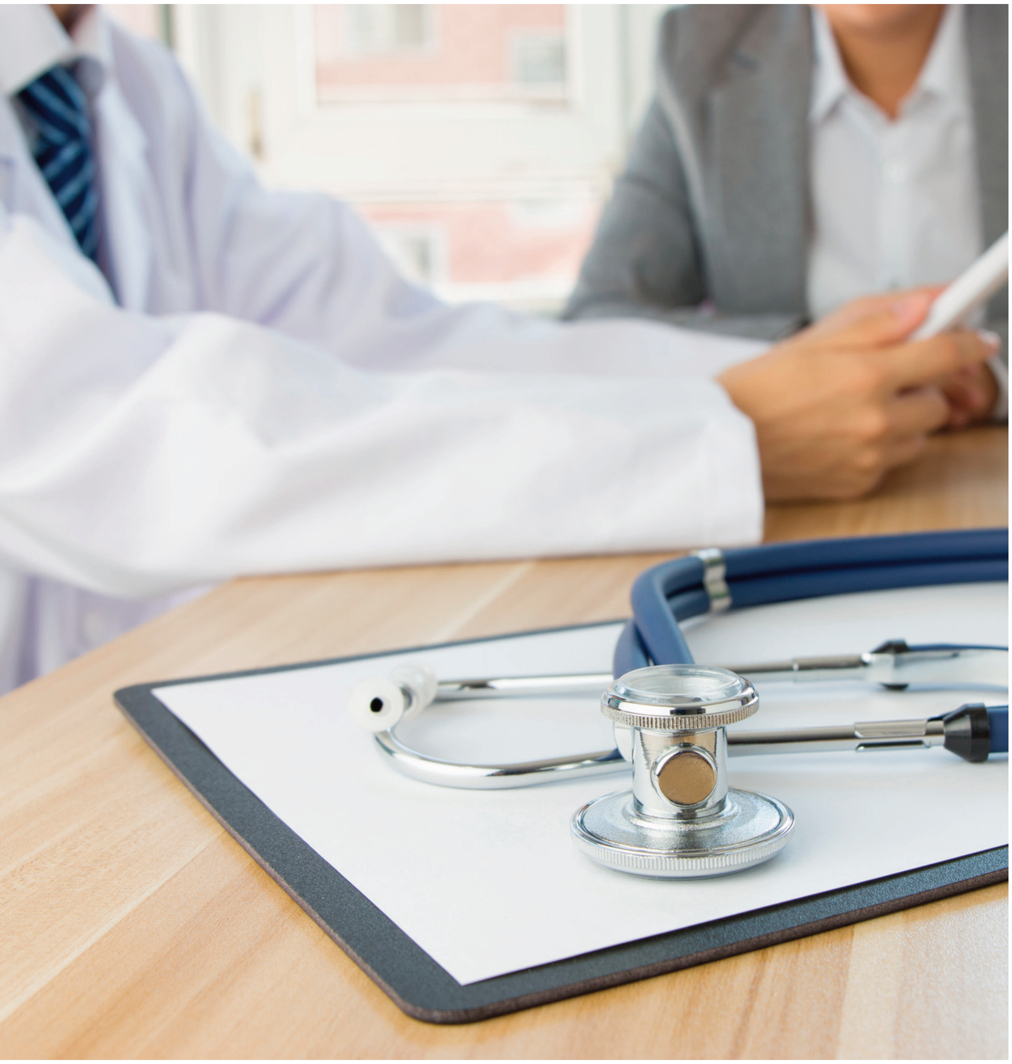

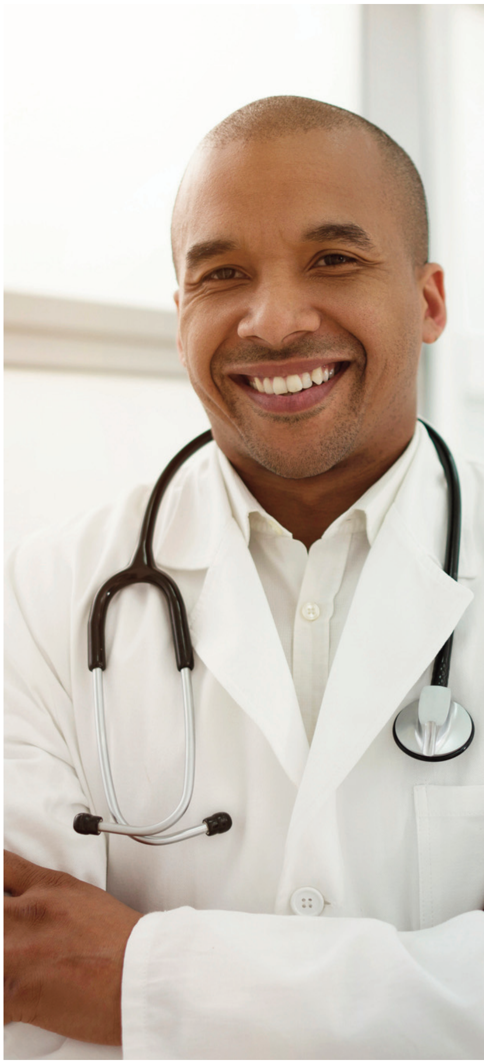

A Joint Program of the Federation of State Medical Boards of the
United States, Inc., and National Board of Medical Examiners

This booklet was updated July 2023.
For Public Release

Copyright © 2023 by the Federation of State Medical Boards of the United States, Inc. (FSMB), and the National Board of Medical Examiners® (NBME®). All rights reserved. Printed in the United States of America. The United States Medical Licensing Examination® (USMLE®) is a joint program of the FSMB and NBME.

CONTENTS

USMLE Step 2 CK Test Question Formats 3

Introduction to USMLE Step 2 CK Sample Test Questions 9

USMLE Laboratory Values 10

USMLE Step 2 CK Sample Test Questions... 13

- Block 1, Items 1–40 13
- Block 2, Items 41–80 29
- Block 3, Items 81–120 47

Answer Form for USMLE Step 2 CK Sample Test Questions....................................... 63

Answer Key for USMLE Step 2 CK Sample Test Questions.. 64

The following are strategies for answering one-best-answer items:

- Read each question carefully. It is important to understand what is being asked.
- Try to generate an answer and then look for it in the response option list.
- Alternatively, read each response option carefully, eliminating those that are clearly incorrect.
- Of the remaining response options, select the one that is most correct.
- If unsure about an answer, it is better to guess since unanswered questions are automatically counted as wrong answers.

Patient Scenario Formats

Patient scenarios for any Single Item or Sequential Item Set may be provided in either Vignette (paragraph) format, or in Chart/Tabular format. Test items using the chart/tabular format are designed to resemble patient charts but are not intended to be an exact representation of a patient chart. Questions written in Chart/Tabular format will contain relevant patient information in list form, organized in clearly marked sections for ease of review. Familiar medical abbreviations may be used within Chart/Tabular format questions.

Single-Item Questions

A single patient-centered vignette is associated with one question followed by four or more response options. The response options are lettered (ie, A, B, C, D, E). A portion of the questions involves interpretation of graphic or pictorial materials. You are required to select the best answer to the question. Other options may be partially correct, but there is only ONE BEST answer. This is the traditional, most frequently used multiple-choice question format on the examination.

Example Question 1

A 32-year-old woman with type 1 diabetes mellitus has had progressive renal failure over the past 2 years. She has not yet started dialysis. Examination shows no abnormalities. Her hemoglobin concentration is 9 g/dL, hematocrit is 28%, and mean corpuscular volume is 94 μm^3. A blood smear shows normochromic, normocytic cells. Which of the following is the most likely cause?

(A) Acute blood loss
(B) Chronic lymphocytic leukemia
(C) Erythrocyte enzyme deficiency
(D) Erythropoietin deficiency
(E) Immunohemolysis
(F) Microangiopathic hemolysis
(G) Polycythemia vera
(H) Sickle cell disease
(I) Sideroblastic anemia
(J) β-Thalassemia trait

(Answer: D)

Example Question 2

Patient Information
Age: 18 years
Gender: F, self-identified
Ethnicity: unspecified
Site of Care: emergency department

History
Chief Complaint: "My roommate took a lot of pills and said she wanted to die."
History of Present Illness:
- patient brought by ambulance 2 hours after a suspected acetaminophen overdose
- roommate had returned to their dorm and found the patient distraught; patient was holding an empty bottle of acetaminophen and said, "I just want to die."
- roommate estimates patient consumed 20 to 30 tablets of 500-mg acetaminophen

Past Medical History:
- generalized anxiety disorder
- major depressive disorder

Medications:
- citalopram

Allergies:
- no known drug allergies

Family History:
- noncontributory

Psychosocial History:
- college freshman; parents live 8 hours away

Physical Examination

Temp	Pulse	Resp	BP	O_2 Sat	Ht	Wt	BMI
36.8°C (98.2°F)	89/min	16/min	108/59 mm Hg	99% on RA	170 cm (5 ft 7 in)	63 kg (140 lb)	22 kg/m^2

- Appearance: awake and alert but distraught and fearful; patient says, "This was totally stupid."
- Pulmonary: clear lung fields
- Cardiac: regular rhythm; S_1 and S_2, with an early systolic murmur
- Abdominal: soft and nontender; liver and spleen cannot be palpated
- Neurologic: no abnormalities; fully oriented

Question: In addition to obtaining serum acetaminophen concentration, which of the following is the most appropriate next step in management?

(A) Administer N-acetylcysteine
(B) Arrange hemodialysis
(C) Gastric lavage
(D) Initiate sodium bicarbonate infusion

(Answer: A)

Sequential Item Sets

A single patient-centered vignette may be associated with two or three consecutive questions about the information presented. Each question is associated with the initial patient vignette but is testing a different point. You are required to select the ONE BEST answer to each question. These questions are designed to be answered in sequential order. You must click “Proceed to Next Item” to view the next item in the set; once you click on this button, the next question will be displayed, and you will not be able to change the answer to the previous question.

Example Sequential Question

A 35-year-old woman is brought to the emergency department because of worsening pain and swelling of her right knee for the past 2 days. She has been taking acetaminophen for the knee pain during the past 2 days, but the pain is worse today. She has not had any trauma to the knee or any previous problems with her joints. She is otherwise healthy and she currently takes an oral contraceptive. She is sexually active and has a 10-year-old son who lives with her. She is a receptionist at a local hotel and she tells you she must stand often while working. She is 160 cm (5 ft 3 in) tall and weighs 52 kg (115 lb); BMI is 20 kg/m^2. Temperature is 37.9°C (98.9°F). The right knee is erythematous, swollen, and tender; there is pain on movement. No other joints are affected. X-ray of the knee shows an effusion but no structural abnormalities of the joint.

Which of the following is the most appropriate next step in diagnosis?

(A) Arthrocentesis of the knee
(B) Blood cultures
(C) Complete blood count
(D) MRI of the knee
(E) Urine cultures

(Answer: A)

Arthrocentesis is done. The synovial fluid is cloudy. Gram stain is negative. Analysis of the synovial fluid shows a leukocyte count of 120,000/mm^3 and 90% neutrophils. Which of the following is the most appropriate additional test on the synovial fluid?

(A) Culture for bacteria
(B) Glucose measurement
(C) Polarized light microscopy
(D) Protein level

(Answer: A)

Abstract Set Format

The abstract item format includes a summary of an experiment or clinical investigation presented in a manner commonly encountered by a physician, eg, as an abstract that accompanies a research report in a medical journal. Examinees must interpret the abstract in order to answer questions on various topics, including

- Decisions about care of an individual patient
- Biostatistics/epidemiology
- Pharmacology/therapeutics
- Use of diagnostic studies

Example Abstract Set

Question

In children with type 1 diabetes mellitus, what factors are associated with increased risk for microalbuminuria and macroalbuminuria?

Methods

Design: Inception cohort followed for a mean of 9.8 years.
Setting: St. Bartholomew's Oxford Diabetes Register, Oxford, England, UK.
Patients: 527 children < 16 years of age (mean age 9 years) who were diagnosed with type 1 diabetes mellitus and included in the Diabetes Register from 1986 to 1997 (90% follow-up).
Prognostic factors: Mean glycated hemoglobin concentration (HbA_{1c}), female sex, mean blood pressure, history of smoking, and age at diagnosis.
Outcomes: Microalbuminuria (albumin-to-creatinine ratio 3.5 to 35 mg/mmol in boys and 4.0 to 47 mg/mmol in girls, in two annual, consecutive, early morning urine samples), and macroalbuminuria (albumin-to-creatinine ratio > 35 mg/mmol in boys and > 47 mg/mmol in girls).

Main results

135 patients (26%) developed microalbuminuria, with a cumulative prevalence of 25.7% (95% CI 21 to 30) after 10 years and 51% (CI 41 to 61) after 19 years. Of the 135 patients with microalbuminuria, there was a cumulative prevalence of regression to the normoalbuminuric range of 52% (CI 42 to 62) after 4.9 years from the onset of microalbuminuria. 18 patients (3%) developed macroalbuminuria, with a cumulative prevalence of 14% (CI 13 to 15) after 3.2 years from onset of microalbuminuria. HbA_{1c} and female sex were associated with increased risk for microalbuminuria (Table). HbA_{1c} and persistent and intermittent microalbuminuria were associated with increased risk for macroalbuminuria (Table).

Conclusions

In children with type 1 diabetes mellitus, poor glycemic control and female sex were associated with development of microalbuminuria. About half of patients with microalbuminuria regressed to normoalbuminuria. Poor glycemic control and persistent or intermittent microalbuminuria were associated with development of macroalbuminuria.

Sources of funding: Diabetes UK; Juvenile Diabetes Research Foundation; Wellcome Trust; NIHR Cambridge Biomedical Research Centre.

Structured abstract based on: Amin R, Widmer B, Prevost AT, et al. **Risk of microalbuminuria and progression to macroalbuminuria in a cohort with childhood onset type 1 diabetes: prospective observational study.** BMJ. 2008;336:697-701. 18349042

Abstract continues on next page.

Factors associated with renal outcomes in children with type 1 diabetes mellitus*

Outcomes	Prognostic factors	At a mean 9.8 years of follow-up
		**Hazard ratio (95% CI)**
Microalbuminuria	HbA_{1c} (per % increase)	1.4 (1.3 to 1.5)
	Female sex	1.4 (1.02 to 2.1)
	Diastolic blood pressure	1.0 (1.0 to 1.0)
	Systolic blood pressure	1.0 (1.0 to 1.0)
	History of smoking	1.3 (0.9 to 2.0)
	Younger age at diagnosis	1.0 (1.0 to 1.1)
Macroalbuminuria	HbA_{1c} (per % increase)	1.4 (1.2 to 1.8)
	Persistent microalbuminuria†	28 (8.0 to 96)
	Intermittent microalbuminuria‡	8.8 (2.4 to 31)
	Female sex	1.3 (0.5 to 3.3)
	Diastolic blood pressure	1.1 (0.9 to 1.1)
	Systolic blood pressure	1.1 (0.9 to 1.1)
	History of smoking	1.3 (0.4 to 4.1)
	Younger age at diagnosis	1.0 (1.0 to 1.1)

*HbA_{1c} = glycated haemoglobin.
†Presence of microalbuminuria at every annual visit after first detection.
‡Positive microalbuminuria followed by regression to normoalbuminuria, then recurrence to microalbuminuria at a later date.

A 12-year-old girl is brought to the office for a follow-up examination 6 months after being diagnosed with type 1 diabetes mellitus. The patient feels well. She says she smokes cigarettes occasionally when with friends. She is not sexually active. She is at the 60th percentile for BMI. Her temperature is 37.0°C (98.6°F), pulse is 80/min, respirations are 16/min, and blood pressure is 108/72 mm Hg. Physical examination shows no other abnormalities. Results of urinalysis are within the reference ranges. Her father, who has type 1 diabetes mellitus and chronic renal disease, asks what markers will be followed to determine his daughter's risk for developing chronic renal disease. Which of the following patient characteristics most increases her risk for developing microalbuminuria over the next 10 years?

(A) Age at diagnosis
(B) BMI
(C) Diastolic blood pressure
(D) Gender
(E) Smoking history

Answer: D

Which of the following current or potential future findings is most likely to increase the patient’s risk for developing macroalbuminuria?

(A) Age at diagnosis
(B) Cigarette use
(C) Elevated systolic blood pressure
(D) Gender
(E) Poor glycemic control

Answer: E

Abstract set continues on next page.

One year later, the patient's urinalysis shows microalbuminuria for the first time. In a population of children of a similar age who have type 1 diabetes mellitus and had microalbuminuria detected for the first time, which of the following outcomes is most likely?

(A) Hemoglobin A_{1c} will normalize within 10 years
(B) Hypertension will be diagnosed within 2 years
(C) Macroalbuminuria will be detected within 1 year
(D) Microalbuminuria will resolve within 5 years

Answer: D

NOTE: When additional question formats are added to the examination, notice will be provided on the USMLE website (www.usmle.org). You must monitor the website to stay informed about the types of questions that occur in the examination, and you must practice with the downloadable sample test questions available on the USMLE website in order to be fully prepared for the examination.

INTRODUCTION TO USMLE STEP 2 CK SAMPLE TEST QUESTIONS

The following pages include 120 sample test questions. Most of these practice questions are the same as those on the USMLE website.

ONLINE:

Please note that reviewing the sample questions as they appear on pages 13–62 is **not a substitute for practicing on the website**.

1. You should run the tutorial and items in the interactive testing experience on the USMLE website well before your test date.
2. The items in the interactive testing experience include additional questions and formats that do not appear in this booklet, such as items with associated audio.

CONTENT:

You should become familiar with all test question formats that will be used in the actual examination.

Although the sample questions exemplify content on the examination, they may not reflect the content coverage on individual examinations.

In the actual examination, questions will be presented in random order. The questions will be presented one at a time in a format designed for easy on-screen reading, including a Normal Laboratory Values button (Table included here on pages 10–12). Photographs, charts, and x-rays in this booklet are not of the same quality as the pictorials used in the actual examination. In addition, you will be able to adjust the brightness and contrast of the computer screen.

TIMING:

To take the following sample test questions as they would be timed in the actual examination, you should allow a maximum of one hour for each block, for a total of three hours. Please be aware that most examinees perceive the time pressure to be greater during an actual examination.

An answer form for recording answers for this practice is provided on page 63. An answer key is provided on page 64. In the actual examination, answers will be selected on the screen; no answer form will be provided.

	Reference Range	SI Reference Intervals
**SERUM**		
General Chemistry:		
Electrolytes		
Sodium (Na^+)	136–146 mEq/L	136–146 mmol/L
Potassium (K^+)	3.5–5.0 mEq/L	3.5–5.0 mmol/L
Chloride (Cl^-)	95–105 mEq/L	95–105 mmol/L
Bicarbonate (HCO_3^-)	22–28 mEq/L	22–28 mmol/L
Urea nitrogen	7–18 mg/dL	2.5–6.4 mmol/L
Creatinine	0.6–1.2 mg/dL	53–106 µmol/L
Glucose	Fasting: 70–100 mg/dL	3.8–5.6 mmol/L
	Random, non-fasting: <140 mg/dL	<7.77 mmol/L
Calcium	8.4–10.2 mg/dL	2.1–2.6 mmol/L
Magnesium (Mg^{2+})	1.5–2.0 mg/dL	0.75–1.0 mmol/L
Phosphorus (inorganic)	3.0–4.5 mg/dL	1.0–1.5 mmol/L
Hepatic:		
Alanine aminotransferase (ALT)	10–40 U/L	10–40 U/L
Aspartate aminotransferase (AST)	12–38 U/L	12–38 U/L
Alkaline phosphatase	25–100 U/L	25–100 U/L
Amylase	25–125 U/L	25–125 U/L
Bilirubin, total // direct	0.1–1.0 mg/dL // 0.0–0.3 mg/dL	2–17 µmol/L // 0–5 µmol/L
Proteins, total	6.0–7.8 g/dL	60–78 g/L
Albumin	3.5–5.5 g/dL	35–55 g/L
Globulin	2.3–3.5 g/dL	23–35 g/L
Lipids:		
Cholesterol		
Total	Normal: <200 mg/dL	<5.2 mmol/L
	High: >240 mg/dL	>6.2 mmol/L
HDL	40–60 mg/dL	1.0–1.6 mmol/L
LDL	<160 mg/dL	<4.2 mmol/L
Triglycerides	Normal: <150 mg/dL	<1.70 mmol/L
	Borderline: 151–199 mg/dL	1.71–2.25 mmol/L
Iron Studies:		
Ferritin	Male: 20–250 ng/mL	20–250 µg/L
	Female: 10–120 ng/mL	10–120 µg/L
Iron	Male: 65–175 µg/dL	11.6–31.3 µmol/L
	Female: 50–170 µg/dL	9.0–30.4 µmol/L
Total iron-binding capacity	250–400 µg/dL	44.8–71.6 µmol/L
Transferrin	200–360 mg/dL	2.0–3.6 g/L

Continued on Next Page

	Reference Range	SI Reference Intervals
Endocrine:		
Follicle-stimulating hormone	Male: 4–25 mIU/mL	4–25 IU/L
	Female: premenopause 4–30 mIU/mL	4–30 IU/L
	midcycle peak 10–90 mIU/mL	10–90 IU/L
	postmenopause 40–250 mIU/mL	40–250 IU/L
Luteinizing hormone	Male: 6–23 mIU/mL	6–23 IU/L
	Female: follicular phase 5–30 mIU/mL	5–30 IU/L
	midcycle 75–150 mIU/mL	75–150 IU/L
	postmenopause 30–200 mIU/mL	30–200 IU/L
Growth hormone - arginine stimulation	Fasting: <5 ng/mL	<5 µg/L
	Provocative stimuli: >7 ng/mL	>7 µg/L
Prolactin (hPRL)	Male: <17 ng/mL	<17 µg/L
	Female: <25 ng/mL	<25 µg/L
Cortisol	0800 h: 5–23 µg/dL	138–635 nmol/L
	1600 h: 3–15 µg/dL	82–413 nmol/L
	2000 h: <50% of 0800 h	Fraction of 0800 h: <0.50
TSH	0.4–4.0 µU/mL	0.4–4.0 mIU/L
Triiodothyronine (T_3) (RIA)	100–200 ng/dL	1.5–3.1 nmol/L
Triiodothyronine (T_3) resin uptake	25%–35%	0.25–0.35
Thyroxine (T_4)	5–12 µg/dL	64–155 nmol/L
Free T_4	0.9–1.7 ng/dL	12.0–21.9 pmol/L
Thyroidal iodine (^{123}I) uptake	8%–30% of administered dose/24 h	0.08–0.30/24 h
Intact PTH	10–60 pg/mL	10–60 ng/L
17-Hydroxycorticosteroids	Male: 3.0–10.0 mg/24 h	8.2–27.6 µmol/24 h
	Female: 2.0–8.0 mg/24 h	5.5–22.0 µmol/24 h
17-Ketosteroids, total	Male: 8–20 mg/24 h	28–70 µmol/24 h
	Female: 6–15 mg/24 h	21–52 µmol/24 h
Immunoglobulins:		
IgA	76–390 mg/dL	0.76–3.90 g/L
IgE	0–380 IU/mL	0–380 kIU/L
IgG	650–1500 mg/dL	6.5–15.0 g/L
IgM	50–300 mg/dL	0.5–3.0 g/L
Other, serum:		
Creatinine clearance	Male: 97–137 mL/min	97–137 mL/min
	Female: 88–128 mL/min	88–128 mL/min
Creatine kinase	Male: 25–90 U/L	25–90 U/L
	Female: 10–70 U/L	10–70 U/L
Lactate dehydrogenase	45–200 U/L	45–200 U/L
Osmolality	275–295 mOsmol/kg H_2O	275–295 mOsmol/kg H_2O
Uric acid	3.0–8.2 mg/dL	0.18–0.48 mmol/L
**GASES, ARTERIAL BLOOD (ROOM AIR)**		
P_{O_2}	75–105 mm Hg	10.0–14.0 kPa
P_{CO_2}	33–45 mm Hg	4.4–5.9 kPa
pH	7.35–7.45	$[H^+]$ 36–44 nmol/L
**CEREBROSPINAL FLUID**		
Cell count	0–5/mm^3	0–5 × 10^6/L
Chloride	118–132 mEq/L	118–132 mmol/L
Gamma globulin	3%–12% total proteins	0.03–0.12
Glucose	40–70 mg/dL	2.2–3.9 mmol/L
Pressure	70–180 mm H_2O	70–180 mm H_2O
Proteins, total	<40 mg/dL	<0.40 g/L

Continued on Next Page

	Reference Range	SI Reference Intervals
**HEMATOLOGIC**		
Complete Blood Count:		
Hematocrit	Male: 41%–53%	0.41–0.53
	Female: 36%–46%	0.36–0.46
Hemoglobin, blood	Male: 13.5–17.5 g/dL	135–175 g/L
	Female: 12.0–16.0 g/dL	120–160 g/L
Mean corpuscular hemoglobin (MCH)	25–35 pg/cell	0.39–0.54 fmol/cell
Mean corpuscular hemoglobin conc. (MCHC)	31%–36% Hb/cell	4.8–5.6 mmol Hb/L
Mean corpuscular volume (MCV)	80–100 μm^3	80–100 fL
Volume		
Plasma	Male: 25–43 mL/kg	0.025–0.043 L/kg
	Female: 28–45 mL/kg	0.028–0.045 L/kg
Red cell	Male: 20–36 mL/kg	0.020–0.036 L/kg
	Female: 19–31 mL/kg	0.019–0.031 L/kg
Leukocyte count (WBC)	4500–11,000/mm^3	4.5–11.0 × 10^9/L
Neutrophils, segmented	54%–62%	0.54–0.62
Neutrophils, bands	3%–5%	0.03–0.05
Lymphocytes	25%–33%	0.25–0.33
Monocytes	3%–7%	0.03–0.07
Eosinophils	1%–3%	0.01–0.03
Basophils	0%–0.75%	0.00–0.0075
Platelet count	150,000–400,000/mm^3	150–400 × 10^9/L
Coagulation:		
Partial thromboplastin time (PTT) (activated)	25–40 seconds	25–40 seconds
Prothrombin time (PT)	11–15 seconds	11–15 seconds
D-Dimer	≤250 ng/mL	≤1.4 nmol/L
Other, Hematologic:		
Reticulocyte count	0.5%–1.5%	0.005–0.015
Erythrocyte count (RBC)	Male: 4.3–5.9 million/mm^3	4.3–5.9 × 10^{12}/L
	Female: 3.5–5.5 million/mm^3	3.5–5.5 × 10^{12}/L
Erythrocyte sedimentation rate (Westergren)	Male: 0–15 mm/h	0–15 mm/h
	Female: 0–20 mm/h	0–20 mm/h
CD4+ T-lymphocyte count	≥500/mm^3	≥0.5 × 10^9/L
Troponin I	≤0.04 ng/mL	≤0.04 µg/L
Endocrine:		
Hemoglobin A_{1c}	≤6%	≤42 mmol/mol
**URINE**		
Calcium	100–300 mg/24 h	2.5–7.5 mmol/24 h
Osmolality	50–1200 mOsmol/kg H_2O	50–1200 mOsmol/kg H_2O
Oxalate	8–40 µg/mL	90–445 µmol/L
Proteins, total	<150 mg/24 h	<0.15 g/24 h
**BODY MASS INDEX (BMI)**	Adult: 19–25 kg/m^2	

BLOCK 1, ITEMS 1–40

1. A 21-year-old man comes to student health services because of a 6-month history of increasingly frequent episodes of moderate chest pain. The first episode occurred while he was sitting in traffic and feeling stressed because he was late for a college class. At that time, he had the sudden onset of moderate chest pain, a rapid heartbeat, sweating, and nausea. He says he felt as though he were going to die. The episode lasted approximately 10 minutes. He had a similar episode 1 month later while on a date; the symptoms were so severe that he abruptly ended the date. During the past 3 weeks, he has experienced two to three episodes weekly. He says he fears having an episode while in public or on a date, so he has decreased his participation in social activities and the amount of time he spends outside of his apartment. He has no history of serious illness and takes no medications. He does not drink alcohol or use other substances. Vital signs are within normal limits. Physical examination discloses no abnormalities. On mental status examination, he has an anxious mood and full range of affect. Which of the following is the most likely diagnosis?

   (A) Agoraphobia
   (B) Generalized anxiety disorder
   (C) Illness anxiety disorder (hypochondriasis)
   (D) Social anxiety disorder (social phobia)
   (E) Somatic symptom disorder

2. A 35-year-old man is brought to the emergency department by a friend 30 minutes after the sudden onset of right-sided weakness and difficulty speaking. The symptoms began while he was lifting weights at the gym. He has not had headache or changes in vision. He has no history of serious illness and takes no medications. He does not smoke cigarettes or drink alcohol. He exercises regularly. His speech is incoherent, but he can understand what others are saying. Vital signs are within normal limits. No bruits are heard over the carotid arteries. Cardiopulmonary examination shows no abnormalities. There is swelling and mild tenderness of the right lower extremity. No cords are palpated. Neurologic examination shows a right facial droop and right upper extremity weakness. An MRI of the brain shows an acute stroke in the left middle cerebral artery territory. Carotid ultrasonography shows no abnormalities. Which of the following is the most appropriate next step in diagnosis?

   (A) Adenosine stress test
   (B) Cardiac catheterization
   (C) Cardiac MRI with gadolinium
   (D) CT angiography
   (E) Echocardiography with bubble study

3. A 65-year-old man comes to the office because of a 2-year history of progressive shortness of breath on exertion and a 6-month history of nonproductive cough. He now has shortness of breath when walking to his mailbox. He has not had fever, weight loss, or chest pain. He has not had recent sick contacts, has had no occupational exposures, and does not own any pets. He has difficult-to-control atrial fibrillation, hypertension, chronic obstructive pulmonary disease, and migraines. Medications are amiodarone, warfarin, lisinopril, tiotropium, and propranolol. Temperature is 37.0°C (98.6°F), pulse is 80/min and irregular, respirations are 16/min, and blood pressure is 110/70 mm Hg. There is no jugular venous distention. Auscultation of the lungs discloses fine crackles bilaterally, both anteriorly and posteriorly, but no egophony. The remainder of the physical examination discloses no abnormalities. An adverse effect of which of the following medications is the most likely cause of these findings?

   (A) Amiodarone
   (B) Lisinopril
   (C) Propranolol
   (D) Tiotropium
   (E) Warfarin

4. An 18-month-old boy is brought to the emergency department by his mother 30 minutes after he fell from his bed onto the floor. Two months ago, he sustained a fracture of the right humerus when he fell while playing in the park. The fracture healed quickly with immobilization and casting. His mother sustained several bone fractures in early childhood, but she currently does not have any medical concerns. On arrival, the boy is crying. Pulse is 162/min, respirations are 48/min, and blood pressure is 122/80 mm Hg. Examination discloses bluish sclera and ecchymoses and tenderness over the right tibia. X-ray of the right lower extremity confirms a fracture of the tibia. The right lower extremity is immobilized with a cast. Limitation of physical activity is recommended to prevent future fractures. Given this patient's condition, it is most appropriate for which of the following types of screening to be done regularly?

(A) Audiography
(B) DEXA scan
(C) Echocardiography
(D) Retinal examination
(E) Serum calcium and vitamin D concentrations

5. A 70-year-old woman is admitted to the hospital because of a 1-hour history of shortness of breath. She has lung cancer and dementia, Alzheimer type, and has had a decline in mental status during the past month. She lives with her boyfriend of 20 years; he has cared for her and has taken care of the home since she was diagnosed with dementia 2 years ago. She has one adult son who lives out of state, but she speaks to him on the phone daily. The patient's neighbor, who is a nurse and a long-term friend, takes her to all medical appointments and ensures she takes her medications appropriately. The patient is unable to understand the poor prognosis of her condition. She has not designated a health care power of attorney. Which of the following is the most appropriate person to make medical decisions for this patient?

(A) Boyfriend
(B) Neighbor
(C) Patient
(D) Physician
(E) Son

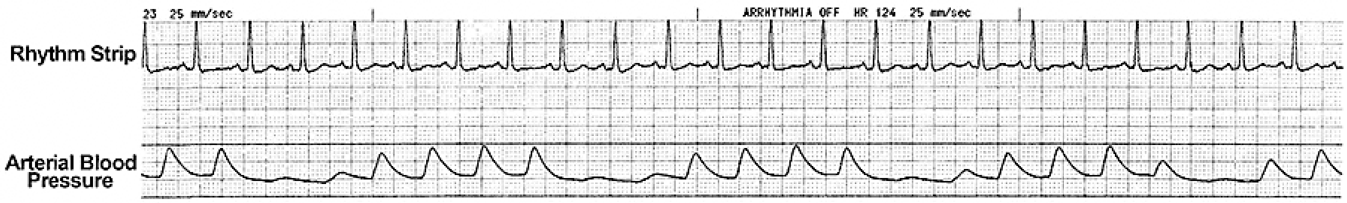

6. A 4-month-old female infant is recovering in the hospital 5 days after uncomplicated surgical repair of a ventricular septal defect via median sternotomy utilizing cardiopulmonary bypass. She was extubated on postoperative day 1 and by postoperative day 4 she was no longer receiving any inotropic infusions or intravenous drugs. She is breast-feeding well; plans are made for discharge from the hospital the next morning. However, on the next morning she develops a temperature of 38.8°C (101.8°F), cool and clammy skin, cold distal extremities, delayed capillary refill, and minimal urine output. Cardiac rhythm strip and tracing of arterial blood pressure are shown. Which of the following is the most appropriate next step in evaluation?

(A) Cardiac catheterization
(B) CT angiography
(C) Echocardiography
(D) Electrophysiology study
(E) MRI of the heart

7. **Patient Information**

Age: 6 years
Sex Assigned at Birth: F
Race/Ethnicity: unspecified
Site of Care: emergency department

History

Reason for Visit/Chief Concern: "My daughter's eyes and legs are swollen."
History of Present Illness:
- 2-week history of cough, congestion, and runny nose that seems to be improving
- parents report the patient's eyes seemed swollen 4 days ago, which they initially attributed to the patient's cold
- 2-day history of swelling over the tops of her feet
- this morning she was unable to put on her shoes because her feet were too swollen

Past Medical History:
- unremarkable

Medications:
- none

Vaccinations:
- up-to-date

Allergies:
- no known drug allergies

Family History:
- mother age 30 years, father age 32 years, and twin sisters age 10 years: alive with no chronic conditions

Physical Examination

Temp	Pulse	Resp	BP	O_2 Sat	Ht	Wt	BMI
37.0°C	140/min	20/min	120/70 mm Hg	99%	115 cm (3 ft 9 in)	25 kg (55 lb)	19 kg/m^2
(98.6°F)				on RA	50th %ile	90th %ile	95th %ile

- Appearance: mildly uncomfortable
- Skin: 2+ pitting edema of both extremities up to the knees
- HEENT: periorbital edema, no erythema; PERRLA; ocular movements are intact
- Pulmonary: clear to auscultation
- Cardiac: regular rhythm; no murmurs
- Abdominal: normoactive bowel sounds; soft, mildly distended, mildly tender to palpation in all quadrants

Diagnostic Studies

Urine	
Specific gravity	1.020 (N=1.003–1.029)
Protein	Large
Ketones	Negative
Blood	Negative
Leukocyte esterase	Negative
WBCs	Negative
RBCs	5–10/hpf

Question: Which of the following additional laboratory findings are most likely to be decreased in this patient?

(A) Hemoglobin
(B) Serum albumin concentration
(C) Serum C3 and C4 concentrations
(D) Serum triglycerides concentration
(E) Serum urea nitrogen concentration

8. A 9-year-old girl is brought to the clinic for a routine examination. She has a 4-year history of asthma. Her only medication is inhaled albuterol with a spacer as needed. She uses it only occasionally and has not used it at all during the past 4 weeks. Her father reports that his daughter has an episode of coughing that awakens her at night once weekly. He also says that his daughter has a sedentary lifestyle because "she coughs if she runs too much." No one in the family smokes cigarettes, and there are no pets. Gas heaters are used in the home. The patient is at the 25th percentile for height and 90th percentile for weight and BMI. Respirations are 16/min. Lungs are clear to auscultation. There is no clubbing of the digits. The remainder of the examination shows no abnormalities. Which of the following is the most appropriate next step in management?

(A) Add oral theophylline to the regimen
(B) Add fluticasone by metered-dose inhaler with a spacer to the regimen
(C) Add salmeterol by metered-dose inhaler with a spacer to the regimen
(D) Begin a 5-day course of oral prednisone
(E) No change in management is necessary

9. A 30-year-old man comes to the office to establish primary care. Medical history is unremarkable and he takes no medications. His father had a myocardial infarction at age 48 years. The patient has smoked one-half pack of cigarettes daily for 10 years. He does not drink alcoholic beverages. He is 168 cm (5 ft 6 in) tall and weighs 82 kg (180 lb); BMI is 29 kg/m^2. Vital signs are within normal limits. The patient is not in distress. Physical examination discloses no abnormalities. Smoking cessation is recommended. Which of the following is the most appropriate screening study for this patient at this time?

(A) ECG
(B) Fasting serum lipid studies
(C) Serum chemistry profile
(D) No screening studies are indicated

10. An 82-year-old woman comes to the office because of a 1-month history of increasing numbness of her feet. She has no history of serious illness. She has taken over-the-counter calcium carbonate tablets for intermittent abdominal pain during the past 40 years; she takes no other medications. She has drunk two glasses of wine with dinner nightly for 50 years. On examination, gastrocnemius deep tendon reflexes are absent. Babinski sign is absent bilaterally. Light touch to the distal lower extremities and feet produces a tingling sensation. Sensation to vibration over the great toes is severely decreased. There is a mild to moderate decrease in proprioception of the great toes. Which of the following is most likely to prevent progression of these neurologic findings?

(A) Alcohol cessation
(B) Calcium carbonate cessation
(C) Folic acid supplementation
(D) Niacin supplementation
(E) Vitamin B_1 (thiamine) supplementation
(F) Vitamin B_{12} (cyanocobalamin) supplementation

11. A 50-year-old man comes to the office for a health maintenance examination. He says he has felt well and reports no symptoms. Medical history is unremarkable. He takes no medications. He uses up to three cans of chewing tobacco weekly and occasionally sleeps with tobacco in his mouth. He says he would like to quit but cannot seem to do it on his own. Vital signs are within normal limits. Examination shows a whitish discoloration that measures approximately 1 cm in diameter on the buccal mucosa. The tongue has a brownish discoloration. Palpation of the neck discloses no lymphadenopathy. In addition to encouraging the patient to quit using chewing tobacco, which of the following is the most appropriate next step in management?

(A) Prescribing oral nystatin suspension
(B) Surgical biopsy of the oral lesion
(C) Swabbing of the white area and sending for cytology
(D) Observation only

12. A 48-year-old man is brought to the emergency department by ambulance 45 minutes after he collapsed at home. He did not have loss of consciousness. He has a 6-week history of shortness of breath and severe fatigue when he walks 20 to 30 feet. During the past 6 weeks, he also has had a 5-kg (11-lb) weight gain despite a decreased appetite. He has no history of serious illness. He cannot remember his last visit to a physician. His only medication is ibuprofen for intermittent mild headaches. His father died of "heart problems" at the age of 78 years. The patient has smoked one pack of cigarettes daily for 22 years. He has consumed 24 cans of beer weekly for 20 years. He works as a painting and drywall contractor but was unable to work last week because of fatigue. Temperature is 36.8°C (98.2°F), pulse is 100/min and regular, respirations are 16/min, and blood pressure is 90/55 mm Hg while supine. Pulse oximetry on 4 L/min of oxygen by nasal cannula shows an oxygen saturation of 91%. On examination, crackles are heard halfway up the lung fields bilaterally. An S_3 is heard; no murmurs are heard. The liver span is 5 cm. There is moderate pitting edema of the calves and ankle swelling bilaterally. Echocardiography is most likely to show which of the following findings?

(A) A large pericardial effusion
(B) Dilated cardiomyopathy
(C) Left ventricular hypertrophy
(D) Paradoxical septal motion
(E) Regional wall motion abnormality

13. A 68-year-old man comes to the clinic because of a 6-month history of frequent falls. He sustained a fracture of the left wrist 4 weeks ago during a fall. He has not had loss of consciousness before or during the falls. He says that turning and pivoting often cause him to fall, and he has a tendency to fall backward when walking. He has Parkinson disease treated with carbidopa-levodopa and entacapone. Vital signs are within normal limits. Examination shows masked facies and a resting tremor of the right upper extremity. Romberg sign is absent. When the patient stands still and is pulled backward, he is unable to maintain his posture. Which of the following is the most appropriate intervention to decrease this patient's risk for future falls?

(A) Biofeedback
(B) Physical therapy
(C) Pramipexole therapy
(D) Ropinirole therapy
(E) Rotigotine therapy

14. A 6-year-old boy is brought to the office by his parents as a new patient. Three months ago, he and his 3-year-old sister and 4-year-old brother were adopted from a Russian orphanage, where they had lived for 2 years. Their biological parents died in a motor vehicle collision; they have no other relatives. The adoptive parents say they were told that the children had stable caregivers, age-appropriate stimulation, and schooling at the orphanage. The children were shy initially on meeting them but now seem comfortable. They play well with each other; the patient is protective of his siblings. His medical and developmental history prior to 2 years ago is unknown. He has been learning English slowly; his parents hope he will learn enough by the end of the summer to begin kindergarten, a grade below that of his peers. Although he can perform basic mathematics, he is unable to read, even in Russian. Results of receptive language testing are consistent with an average IQ. His daily living skills are appropriate for age. He is at the 10th percentile for height and weight. During the examination, the patient's adoptive mother, who is fluent in Russian, translates for him. Physical examination shows no abnormalities. On mental status examination, he is shy and maintains intermittent eye contact. He slowly answers questions with brief statements. He smiles and says he is excited and pleased about being adopted. Results of laboratory studies are within the reference ranges. Which of the following is the most likely diagnosis?

(A) Autism spectrum disorder
(B) Fetal alcohol syndrome
(C) Intellectual developmental disorder
(D) Learning disorder
(E) Post-traumatic stress disorder
(F) Reactive attachment disorder

15. A 17-year-old boy is brought to the emergency department because of a 1-hour history of progressive difficulty breathing and a 2-hour history of an itchy, red rash over his trunk, arms, and legs. He has not used new soaps, detergents, or lotions during the past week. Thirty-six hours ago, he began a 1-week course of amoxicillin for an ear infection; he has taken four doses since that time. He has no other history of serious illness and no known allergies. He takes no other medications. Pulse is 100/min, respirations are 28/min, and blood pressure is 90/60 mm Hg. Pulse oximetry on room air shows an oxygen saturation of 95%. He appears anxious and is in moderate respiratory distress. Examination shows an erythematous, raised, demarcated rash over the trunk and all extremities. Which of the following is the most appropriate next step in management?

(A) Administration of albuterol
(B) Administration of diphenhydramine
(C) Administration of epinephrine
(D) Complete blood count
(E) Observation only

16. Two weeks after undergoing open cholecystectomy for gangrenous cholecystitis, a 47-year-old woman comes to the clinic for removal of surgical staples. She was treated with oral vancomycin for *Clostridioides difficile* colitis during hospitalization. She has type 2 diabetes mellitus. Current medications are metformin and pantoprazole. She reports loose stools four times daily during the past 2 weeks. In addition to wearing a clean isolation gown, which of the following is the most appropriate precaution for the physician to take?

(A) Washing hands thoroughly with alcohol gel
(B) Washing hands thoroughly with soap and water
(C) Wearing a surgical mask
(D) Wearing sterile gloves and preparing the staple skin line with chlorhexidine
(E) No additional precautions are necessary

The abstract displayed on this page is for use with items #17–19 on the following page.

Question

In patients with opioid use disorder, what is the relative efficacy of buprenorphine and methadone maintenance therapy?

Review scope

Included studies compared buprenorphine maintenance therapy (BMT), >1 mg/day, with methadone maintenance therapy (MMT), ≥20 mg/day, or placebo (including 1 mg/day of buprenorphine) in patients who were dependent on heroin or other opioids. Studies of pregnant women and those assessing buprenorphine or methadone for detoxification but with no maintenance phase were excluded. Outcomes included treatment retention and urinalysis-confirmed use of morphine, cocaine, and benzodiazepines.

Review methods

MEDLINE and EMBASE/Excerpta Medica (2003 to Jan 2013), Cochrane Central and Cochrane Library (2013, Issue 1), PsycLIT, Current Contents, several other databases, ClinicalTrials.gov and other electronic sources of ongoing trials, conference proceedings, Library of Congress databases, national focal points for drug research, and reference lists were searched for randomized controlled trials (RCTs). Authors were consulted. 31 RCTs (n=5430), ranging in size from 40 to 736 patients, met the selection criteria. Duration of study interventions ranged from 2 weeks to 52 weeks. 20 trials compared BMT with MMT, and 11 compared BMT with placebo.

Main results

As shown in the table below, low-, medium-, and high-dose BMT increased treatment retention more than placebo; BMT did not differ from equivalent doses of MMT for retention or had lower retention.

High-dose (standard mean difference −1.17, 95% CI −1.85 to −0.49), but not medium- or low-dose, BMT decreased morphine use more than placebo. Medium- and flexible-dose BMT and MMT did not differ for morphine use or cocaine use. BMT vs MMT and high-dose BMT vs placebo did not differ for benzodiazepine use.

Buprenorphine maintenance therapy (BMT) vs methadone maintenance therapy (MMT) or placebo for treatment retention in patients with opioid use disorder

Comparisons*	Number of Trials (n)	Weighted Retention Rates	RBI (95% CI)	NNT (95% CI)
High-dose BMT vs placebo	5 (1001)	72% vs 40%	82% (15 to 190)	4 (3 to 7)
Medium-dose BMT vs placebo	4 (887)	66% vs 38%	74% (6 to 187)	4 (2 to 34)
Low-dose BMT vs placebo	5 (1131)	60% vs 40%	50% (19 to 88)	5 (4 to 10)
			**RBR (95% CI)**	**NNH (95% CI)**
High-dose BMT vs high-dose MMT	1 (134)	5.2% vs 6.6%	21% (−216 to 20)	Not significant
Medium-dose BMT vs medium-dose MMT	7 (780)	43% vs 48%	13% (−10 to 31)	Not significant
Flexible-dose BMT vs flexible-dose MMT	11 (1391)	50% vs 63%	17% (5 to 27)	8 (5 to 25)
Low-dose BMT vs low-dose MMT	3 (253)	38% vs 56%	33% (13 to 48)	6 (4 to 17)

CI = confidence interval; NNH = number needed to harm; NNT = number needed to treat; RBI = relative benefit increase; RBR = relative benefit reduction.
*Flexible dose = dose is titrated within a broad dose range according to patient preference; low-dose BMT = 2 to 6 mg; medium-dose BMT = 7 to 15 mg; high-dose BMT = 16 mg; medium-dose MMT = 40 to 85 mg.

Conclusions

In patients with opioid use disorder, buprenorphine is more efficacious than placebo, but less efficacious than methadone therapy, for treatment retention.

Source of funding: Australian Government Department of Health.

Structured abstract based on: Mattick RP, Breen, C, Kimber J, Davoli M. **Buprenorphine maintenance versus placebo or methadone maintenance for opioid dependence**. Cochrane Database Syst Rev. 2014;(2):CD002207. 24500948

Items #17–19 refer to the abstract displayed on the previous page.

17. A 38-year-old woman comes to the clinic for follow-up examination. She has opioid use disorder and currently takes methadone 40 mg daily as maintenance therapy. She says the methadone is causing mild sedation that is interfering with her job. She would like to discontinue methadone maintenance therapy but is concerned about heroin relapse. She says she also takes a friend's valium when she is feeling "stressed out." The physician discusses alternative maintenance therapy strategies with the patient. The physician discusses the pros and cons of medium- or high-dose buprenorphine maintenance therapy compared with medium- or high-dose methadone therapy with the patient. Based on this abstract, which of the following is the most appropriate information for the physician to convey to the patient?

(A) Buprenorphine is 13% and 21% less effective than methadone at medium and high doses, respectively
(B) Buprenorphine is a better maintenance strategy if the patient is also regularly using benzodiazepines
(C) Methadone is statistically more likely to result in treatment retention
(D) Neither drug is more effective than placebo
(E) There is an unclear difference in efficacy between the two drugs at these doses

18. Which of the following aspects of the study is most likely to limit confidence in the authors' conclusions?

(A) Exclusion of patients using methadone for acute detoxification
(B) Exclusion of pregnant patients
(C) Inclusion of randomized controlled trials only
(D) Inclusion of trials that compared active drug with placebo
(E) Inclusion of trials with an intervention duration of 2 weeks
(F) Use of urinalysis to determine treatment retention

19. Which of the following is most likely to bias the results of this study?

(A) Earlier diagnosis of relapse in patients receiving methadone maintenance therapy
(B) Exclusion criteria of studies used for the review
(C) Likelihood that more trials with positive results will be published
(D) Source of funding
(E) Variety of sample sizes of the included trials

END OF SET

---

20. A 57-year-old man is brought to the emergency department by ambulance 30 minutes after he had loss of consciousness. His wife says he was making dinner when he fell on the kitchen floor. He has hypertension and his only medication is lisinopril. He has no history of operative procedures. En route, his temperature was 37.2°C (99.0°F), pulse was 90/min, respirations were 10/min and labored, and blood pressure was 180/100 mm Hg. Pulse oximetry on 40% oxygen by face mask showed an oxygen saturation of 99%. On arrival, he is unresponsive. His Glasgow Coma Scale score is 3. He is intubated and mechanically ventilated. Temperature is 37.2°C (99.0°F), pulse is 90/min, ventilatory rate is 10/min, and blood pressure is 180/100 mm Hg. Pulse oximetry on an F_{IO_2} of 0.4 shows an oxygen saturation of 98%. On examination, Doll's eye (oculocephalic) maneuver shows absent eye movements, and the corneal reflex is absent. On ice-water caloric testing, there is no nystagmus or deviation of the eyes toward the ear being irrigated. CT scan of the head shows a ruptured cerebral aneurysm. When the patient's wife is notified of the patient's condition, she says he wanted to be an organ donor but does not have an advance directive. Which of the following is the most appropriate next step in management?

(A) Consult with the hospital ethics committee
(B) Contact the organ bank for potential donation
(C) Determine if there are any patients awaiting organ donation in the hospital
(D) Explain that the patient is not a candidate for organ donation because he does not have an advance directive
(E) Explain that the patient is not a candidate for organ donation because of his history of hypertension

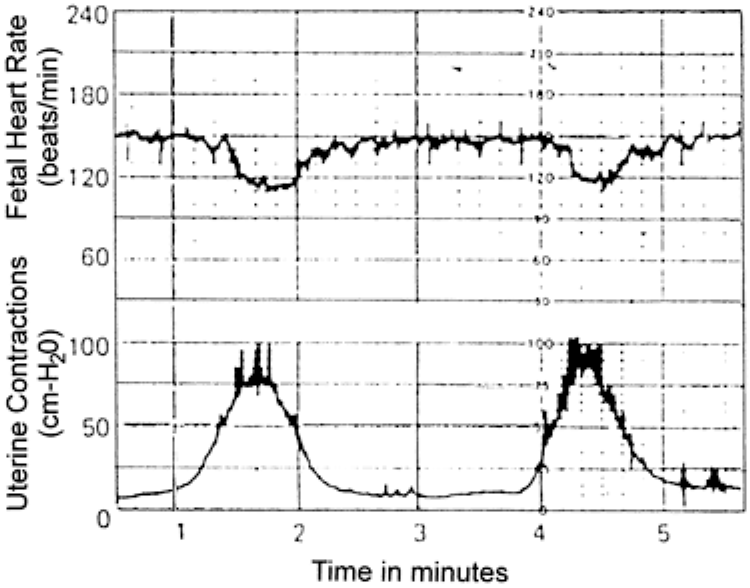

21. A 36-year-old woman, gravida 2, para 1, at 39 weeks' gestation is admitted to the hospital in labor. She reports painful contractions that occur every 2 to 3 minutes and last 60 seconds. She has not had vaginal bleeding. She has no history of serious illness, and pregnancy has been uncomplicated. Temperature is 37.0°C (98.6°F), pulse is 84/min and regular, and blood pressure is 100/70 mm Hg. The abdomen is nontender and consistent in size with a 39-week gestation. External fetal monitoring shows a heart rate of 150/min with moderate variability, several spontaneous accelerations, and no decelerations. An external tocometer shows regular uterine contractions every 2 to 3 minutes. The cervix is 6 cm dilated and 100% effaced; the vertex is at 0 station. Ultrasonography shows the fetus in a cephalic presentation. The membranes are artificially ruptured, yielding copious clear fluid. Thirty minutes later, the cervix is 9 cm dilated and 100% effaced; the vertex is at +1 station. A fetal heart tracing is shown. Which of the following is the most appropriate next step in management?

(A) Advising the patient to begin pushing
(B) Amnioinfusion
(C) Expectant management
(D) Forceps-assisted vaginal delivery
(E) Immediate cesarean delivery

---

22. A 27-year-old woman comes to the office because of a 3-week history of nasal congestion, cough productive of sputum, intermittent moderate headache, and intermittent moderate pain over her cheeks. She has had similar symptoms three to four times yearly during the past 5 years. The symptoms last 2 to 8 weeks and generally resolve spontaneously. She has had two episodes of bacterial pneumonia during the past 5 years; both episodes resolved with antibiotic therapy. Her only current medication is acetaminophen as needed for headache. Vital signs are within normal limits. Examination shows no abnormalities. Serum study results show decreased IgA, IgG, and IgM concentrations. Antibiotic therapy is begun, and the patient's condition improves. Which of the following is most likely to decrease the likelihood of recurrent infection in this patient?

(A) Daily inhaled tobramycin therapy
(B) Daily intranasal glucocorticoid therapy
(C) Daily trimethoprim-sulfamethoxazole therapy
(D) Intranasal influenza virus vaccine administration
(E) Monthly immune globulin replacement therapy

23. A 37-year-old man comes to the office because of a 2-year history of mild to moderate fatigue. The fatigue waxes and wanes without relation to activity. He has no history of serious illness and takes no medications. He drinks three glasses of whiskey weekly. He has had two lifetime female sexual partners; he uses condoms consistently. He immigrated to the United States from China 15 years ago. Vital signs are within normal limits. Examination discloses no abnormalities. Results of laboratory studies are shown:

Serum	
Creatinine	1.2 mg/dL
ALT	85 U/L
AST	60 U/L
HBsAg	positive
Anti-HCV	negative
Anti-HBs	negative
IgG anti-HBc	positive
Blood	
Hematocrit	35%
Hemoglobin	12 g/dL
MCV	82 μm^3

Without treatment of his current condition, this patient is most likely to develop which of the following?

(A) Amyloidosis
(B) Essential mixed cryoglobulinemia
(C) Hepatocellular carcinoma
(D) Membranoproliferative glomerulonephritis
(E) Polyarteritis nodosa
(F) Sjögren syndrome

24. A 10-year-old boy is brought to the office because of moderate right knee pain. He is in no distress. Medical history is remarkable for hemophilia A. Examination of the right knee shows warmth and swelling; there is pain with flexion. Range of motion is limited by pain and swelling. Which of the following is the most appropriate therapy for this patient?

(A) ADH (vasopressin)
(B) Factor VIII concentrate
(C) Factor IX concentrate
(D) Ferrous sulfate
(E) Fresh frozen plasma

25. A 72-year-old man comes to the clinic because of a 3-week history of visual changes in his right eye. During this period, he has noticed that the lines of his daily crossword puzzle look curved, and the blinds in his apartment appear wavy. He wears magnifying lenses for reading. He has not had pain in his eye or photophobia. He has no history of trauma to the area. On examination, visual acuity is 20/200 in the right eye and 20/100 in the left eye. Both pupils are round and reactive to light. Palpation of both globes through closed eyelids shows no abnormalities. There is normal red reflex bilaterally. The lenses appear clear. Which of the following is the most likely diagnosis?

(A) Cataracts
(B) Central retinal artery occlusion
(C) Closed-angle glaucoma
(D) Macular degeneration
(E) Temporal arteritis

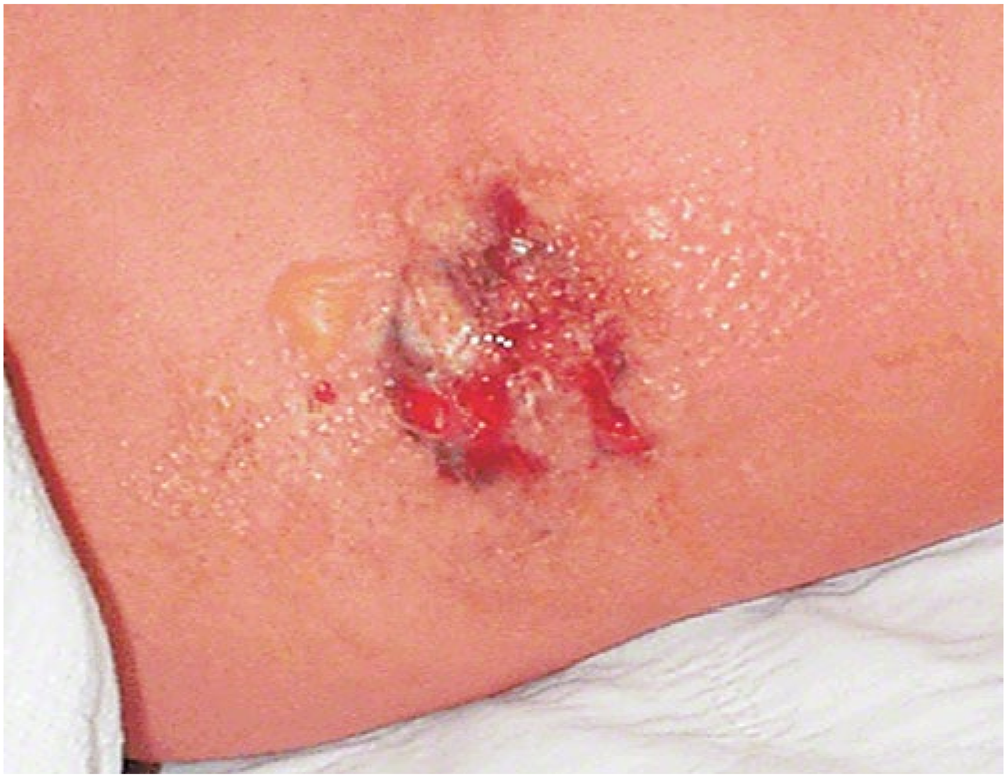

26. A 28-year-old man comes to the clinic because of a 2-day history of a red, severely painful lesion on his left forearm. He says that he felt stinging pain as he put his shirt on in the dark 2 days ago; the pain was so severe he immediately threw down the shirt and saw a spider running across the floor. He returned yesterday from a 1-week trip to the mountains in the southeastern United States. He has type 1 diabetes mellitus treated with lisinopril and insulin. He does not drink alcoholic beverages. He is sexually active with two male partners and uses condoms inconsistently. Vital signs are within normal limits. A photograph of the lesion is shown. No other abnormalities are noted. Which of the following physical findings is most likely to appear during the next 24 hours?

(A) Areas of eschar at the center of the wound site
(B) Gangrene of the distal aspect of the left extremity
(C) Generalized upper extremity edema
(D) Palpable purpuric lesions across the trunk and upper extremities
(E) Rapidly spreading erythema around the wound site
(F) Tender, erythematous streaks up to the epitrochlear nodes

---

27. A 37-year-old man comes to the clinic for a health maintenance examination prior to employment. He feels well. He has no history of serious illness and takes no medications. One year ago, his blood pressure was 136/85 mm Hg. He is 173 cm (5 ft 8 in) tall and weighs 100 kg (220 lb); BMI is 33 kg/m^2. Today, his blood pressure is 138/87 mm Hg. Examination shows no abnormalities. In addition to recommending weight loss, which of the following is the most appropriate next step to prevent cardiovascular morbidity in this patient?

(A) ACE inhibitor therapy
(B) Calcium supplementation
(C) DASH diet
(D) Fish oil supplementation
(E) Thiazide diuretic therapy

28. A 39-year-old man comes to the office because of a 6-week history of mild fatigue and increased thirst and urination. During this time, he also has had a 6.8-kg (15-lb) weight loss despite no change in appetite. He is otherwise asymptomatic. He has no history of serious illness and takes no medications. He is 180 cm (5 ft 11 in) tall and weighs 61 kg (135 lb); BMI is 19 kg/m^2. Temperature is 37.0°C (98.6°F), pulse is 80/min, respirations are 18/min, and blood pressure is 118/70 mm Hg. Examination shows no abnormalities. Results of laboratory studies are shown:

Serum	
Na^+	135 mEq/L
K^+	4.2 mEq/L
Cl^-	100 mEq/L
Urea nitrogen	25 mg/dL
Glucose	578 mg/dL
Blood	
Hemoglobin A_{1c}	10.7%

In addition to recommending dietary modification, which of the following is the most appropriate next step in management?

(A) Glyburide therapy
(B) Insulin therapy
(C) Metformin therapy
(D) Pioglitazone therapy
(E) Sitagliptin therapy

29. Two days after admission to the hospital for treatment of a fractured femur and closed head injury sustained in a motor vehicle collision, an 8-year-old girl has a brief, generalized tonic-clonic seizure. CT scan of the head on admission disclosed no abnormalities. She has no history of serious illness. Oral acetaminophen was initiated for pain, and the patient underwent open reduction and internal fixation of the fracture. She receives no routine medications. Today, she appears drowsy but responds to questions appropriately. She is at the 55th percentile for height, weight, and BMI. Temperature is 36.8°C (98.2°F), pulse is 92/min, respirations are 24/min, and blood pressure is 106/64 mm Hg. Pulse oximetry on room air shows an oxygen saturation of 95%. Pupils are equal and reactive to light and accommodation. Ocular movements are full. Muscle strength is 5/5, and deep tendon reflexes are normal. Results of serum studies are shown:

Na^+	122 mEq/L
K^+	3.8 mEq/L
Cl^-	94 mEq/L
HCO_3^-	24 mEq/L
Glucose	80 mg/dL
Calcium	8.8 mg/dL

Which of the following is the most appropriate next step in management?

(A) Administer a bolus of intravenous 3% saline
(B) Administer subcutaneous vasopressin
(C) Initiate fosphenytoin therapy
(D) Initiate therapy with 0.9% saline at 1.5 times the maintenance requirements
(E) Order EEG
(F) Order MRI of the brain

30. A 43-year-old man comes to the office because of a 2-week history of fatigue and mild pain in his right armpit. He has not had fever or weight loss. Medical history is remarkable for type 2 diabetes mellitus and hypercholesterolemia. Medications are metformin, simvastatin, and aspirin. He is a social worker and frequently visits clients in homeless shelters. He lives alone with two dogs and a cat and has one male sexual partner. Vital signs are within normal limits. He appears well. Examination of the right upper extremity shows an erythematous axilla with a 4-cm, mobile, tender, nonfluctuant axillary lymph node and a tender, 1-cm epitrochlear lymph node. Abdominal examination shows no hepatosplenomegaly. Which of the following is the most likely diagnosis?

(A) Castleman disease
(B) Cat-scratch disease
(C) Hidradenitis suppurativa
(D) T-cell lymphoma
(E) Tuberculosis

31. A 2-year-old girl is brought to the office by her parents for a well-child examination. She was adopted 2 months ago. She has no history of serious illness and receives no medications. Vital signs are within normal limits. Examination shows no abnormalities. After the examination, the parents ask when they should tell their daughter about the adoption. Which of the following is the most appropriate advice regarding the best time to tell this patient?

(A) After she turns 18 years of age
(B) As early as possible, even if she cannot process the whole experience
(C) Once the seal on the adoption records has been lifted
(D) Right before she is enrolled in kindergarten
(E) When she learns that she is not biologically related to her parents

32. A surgical intensive care unit observes that the number of days that patients have central venous catheters in place and the number of bloodstream infections have been increasing. A multidisciplinary team from the unit would like to decrease the number of days that central venous catheters are in place in order to improve performance in this area. Which of the following is the most appropriate next step?

(A) Ask clinical providers in the unit to identify what they believe to be the most common factors leading to nonremoval of central venous catheters
(B) Change all central venous catheters every 5 days
(C) Place stickers on all intensive care unit doors reminding staff to reassess the need for a central line
(D) Plan a randomized controlled trial to test the effects of a reminder to reassess the need for a central line on a daily goals sheet implemented during rounds

33. A 17-year-old girl comes to the office because of a 3-month history of daily moderate epigastric pain that is more severe after meals. Three weeks ago, she had watery diarrhea for 2 days, which resolved spontaneously. She has not had vomiting. She has no history of serious illness and takes no medications. Vital signs are within normal limits. Examination, including abdominal examination, shows no abnormalities. Which of the following is most likely to confirm the diagnosis?

(A) CT scan of the abdomen
(B) Endoscopy
(C) Examination of the stool for ova and parasites
(D) Stool culture
(E) Upper gastrointestinal series

34. A 23-year-old man comes to the emergency department because of a 1-hour history of intermittent coughing spasms that began suddenly while he was attempting to clean mold from his garage with a mixture of acetic acid and cleaning agents. The cough is not productive. He reports shortness of breath only during the coughing spasms. Medical history is unremarkable and he takes no medications. He does not smoke cigarettes, drink alcoholic beverages, or use other substances. He is 180 cm (5 ft 11 in) tall and weighs 75 kg (165 lb); BMI is 23 kg/m^2. Vital signs are within normal limits. Pulse oximetry on room air shows an oxygen saturation of 94%. The patient has periodic paroxysmal coughing during the examination. Oropharynx is clear. Auscultation of the lungs discloses bilateral wheezes throughout all lung fields. There is no use of accessory muscles of respiration. The remainder of the physical examination discloses no abnormalities. Chest x-ray shows mild hyperexpansion of the lungs bilaterally. Results of arterial blood gas analysis on room air are shown:

PO_2	72 mm Hg
PCO_2	38 mm Hg
pH	7.41
HCO_3^-	23 mEq/L

Which of the following is the most appropriate initial step in management?

(A) Albuterol therapy
(B) Bronchoscopy
(C) CT scan of the chest
(D) Intubation
(E) Methylprednisolone therapy

35. An 87-year-old man is admitted to the hospital because of a 4-day history of moderate abdominal cramps, nausea, and vomiting. He has not had a bowel movement or passed gas in the past 24 hours. He has colon cancer metastatic to the lungs, liver, and peritoneum. Eighteen months ago, he underwent a low anterior resection for T3N2 rectal cancer. Six months ago, exploratory laparotomy showed diffuse carcinomatosis and bowel obstruction, and an ileotransverse bypass was performed. The patient and his family were informed of the findings and advised that he would not be a candidate for a laparotomy in the future because there would be no surgical options to treat another obstruction. The patient has no other history of serious illness. Medications are 5-fluorouracil and bevacizumab. Temperature is 37.2°C (99.0°F), pulse is 100/min, respirations are 20/min, and blood pressure is 130/80 mm Hg. The abdomen is distended and mildly tender to palpation. There are no peritoneal signs. CT scan of the abdomen shows a mid-jejunal small-bowel obstruction, multiple intraperitoneal tumor deposits, and ascites. The physician recommends insertion of nasogastric and percutaneous endoscopic gastrostomy tubes. The patient and his family request that the patient undergo a laparotomy and intestinal bypass. Which of the following is the most appropriate next step in management?

(A) Abide by the wishes of the patient and his family
(B) Alter the chemotherapy regimen
(C) Explain the futility of the operation to the patient and his family
(D) Offer to transfer the patient to another facility
(E) Suggest external beam radiation therapy

36. A 62-year-old woman comes to the office because of a 3-month history of anxiety, insomnia, and frequent bowel movements. She has had a 9-kg (20-lb) weight loss during this period. Pulse is 90/min and irregularly irregular, respirations are 22/min, and blood pressure is 150/65 mm Hg. She is restless. The thyroid gland is diffusely enlarged. A bruit is heard over the thyroid. Examination shows palmar erythema; some of her fingernails are separated from the nail beds. There is widening of the palpebral fissures and thickening of the skin over the dorsum of the feet with a peau d'orange appearance. There is a fine tremor. Which of the following is the most likely diagnosis?

(A) Addison disease
(B) Carcinoid syndrome
(C) Cushing syndrome
(D) Pheochromocytoma
(E) Thyrotoxicosis

37. **Patient Information**
Age: 28 years
Gender: M, self-identified
Race/Ethnicity: unspecified
Site of Care: clinic

History
Reason for Visit/Chief Concern: follow-up 1 week after undergoing colonoscopy because of a 3-month history of hematochezia and iron deficiency anemia
History of Present Illness:
• 3-month history of three loose bowel movements daily
• no fever or abdominal pain
• since colonoscopy, no bright red blood per rectum
• reports no new symptoms today
Past Medical History:
• generalized anxiety disorder

Medications:
• citalopram
Allergies:
• no known drug allergies
Family History:
• mother and father alive with no chronic conditions
Psychosocial History:
• has smoked two packs of cigarettes daily for 10 years
• does not drink alcoholic beverages

Physical Examination

Temp	Pulse	Resp	BP	O_2 Sat	Ht	Wt	BMI
36.1°C (97.0°F)	76/min	14/min	100/62 mm Hg	–	170 cm (5 ft 7 in)	59 kg (130 lb)	20 kg/m^2

• Abdominal: scaphoid; normoactive bowel sounds; liver span is 9 cm by percussion; mild tenderness to palpation over the right lower quadrant without rebound or guarding; no hepatosplenomegaly

Diagnostic Studies
• colonoscopy: areas of coalesced ulcers with normal appearing mucosa between, involving the ileum

Question: Which of the following is the most appropriate recommendation for this patient considering his condition?

(A) Annual screening for lymphoproliferative disorders
(B) Discontinuation of citalopram therapy
(C) Prophylactic colectomy
(D) Smoking cessation

38. A 19-year-old man is brought to the emergency department (ED) by ambulance 15 minutes after he was stabbed in the chest during a fight. The stab wound is located medially to the left nipple. At the scene, pulse was 105/min, respirations were 18/min, and blood pressure was 120/80 mm Hg. Pulse oximetry on room air showed an oxygen saturation of 96%. Two large-bore intravenous catheters were placed en route to the ED, and lactated Ringer solution was initiated. On arrival in the trauma bay, the patient develops a pulse of 130/min and blood pressure of 80/40 mm Hg. There is jugular venous distention. Results of focused assessment with sonography for trauma (FAST) are most likely to lead to which of the following next steps for this patient?

(A) CT scan of the chest and abdomen with contrast
(B) Exploratory laparotomy
(C) Needle decompression and chest tube placement
(D) Pericardiocentesis
(E) Peritoneal lavage

39. A 47-year-old woman is scheduled to undergo right knee arthroscopy and partial medial meniscectomy. Medical history is otherwise unremarkable. At her preoperative examination 2 weeks ago, her surgeon discussed the risks and benefits of the procedure, and both parties signed the operative consent form. On the patient's arrival at the hospital, the paperwork is reviewed. She is taken into the operating room and sedated, and the anesthesiology team starts a spinal anesthetic. After the patient's right lower extremity is prepped for the procedure, a surgical pause is taken to review the patient's name and consent form and to confirm the site of surgery. The operating room team is unable to locate the consent form for review. No markings are present on the patient's extremities to indicate the site of the procedure. The surgeon says that he remembers reviewing the consent form with the patient and seeing her sign it. The patient's husband is in the waiting room. Which of the following is the most appropriate course of action?

(A) Ask the patient if she recalls signing the consent form and, if so, proceed with the operation
(B) Ask the patient to sign a new consent form and proceed with the operation
(C) Do not proceed, and remove the patient from the operating room
(D) Obtain verbal consent from the patient's husband before proceeding with the operation
(E) Proceed with the operative procedure as planned

40. A 30-year-old man comes to the physician because he and his wife have been unable to conceive during the past 2 years. Previous evaluation of his wife showed no abnormalities. He has not been exposed to pesticides, heavy metals, radiation, or testicular overheating. Physical examination shows no abnormalities. Serum studies show a testosterone concentration within the lower reference range. A diagnosis of oligospermia is made. Which of the following is the most appropriate initial action by the physician?

(A) Ask the patient about his alcohol intake, smoking, and stress level
(B) Educate the patient about declining sperm counts with increased age
(C) Prescribe a phosphodiesterase inhibitor
(D) Provide a topical testosterone gel
(E) Recommend in vitro fertilization

BLOCK 2, ITEMS 41–80

41. A 40-year-old man comes to the office during the summer because of a 3-day history of fever, diffuse muscle aches, fatigue, and sore throat. He has had no sick contacts. Medical history is unremarkable and he takes no medications. He received the influenza virus vaccine this year. Ten days ago, he returned from a trip to Malaysia, where he says he enjoyed the local food and nightlife. Temperature is 38.8°C (101.8°F), pulse is 90/min, respirations are 16/min, and blood pressure is 126/70 mm Hg. The remainder of the examination shows no abnormalities. Results of laboratory studies are shown:

Blood	
Hemoglobin	10 g/dL
MCV	90 μm^3
WBC	2400/mm^3
Neutrophils, segmented	60%
Neutrophils, bands	5%
Lymphocytes	5%
Monocytes	20%
Eosinophils	4%
Basophils	6%
Platelet count	350,000/mm^3
Red cell distribution width	14%

Monospot test result is negative. Which of the following is the most appropriate next step in diagnosis?

(A) Cytomegalovirus serologic testing
(B) Epstein-Barr virus serologic testing
(C) Hemagglutination inhibition assay
(D) HIV RNA polymerase chain reaction testing
(E) Serum hepatitis B surface antigen testing

42. A 22-year-old man is brought to the emergency department by his girlfriend 30 minutes after she found him unconscious in his home. She last saw him yesterday and he appeared well. He has a history of cocaine and heroin use, and also use of prescription narcotic analgesics that he obtains from friends. On arrival, he is lethargic. Temperature is 35.0°C (95.0°F), pulse is 68/min, respirations are 8/min, and blood pressure is 96/50 mm Hg. Pulse oximetry on room air shows an oxygen saturation of 96%. The left lower extremity is cyanotic and cold to the touch. The remainder of the examination shows no abnormalities. Results of serum studies are shown:

K^+	5.5 mEq/L
HCO_3^-	18 mEq/L
Creatinine	1 mg/dL
Creatine kinase	50,000 U/L

This patient is at greatest risk for which of the following conditions?

(A) Acute kidney injury
(B) Acute liver failure
(C) Cardiac arrhythmia
(D) Hypocalcemia
(E) Hypophosphatemia

43. A 62-year-old woman is brought to the emergency department (ED) by her husband because of a 3-day history of increasing confusion. She has a 20-year history of bipolar disorder well controlled with medication for the past 5 years. Her husband forgot to bring her medication bottles and cannot remember which medication she takes. He reports that she has recently had mild headaches and has taken acetaminophen as needed. On questioning, he says that there has been no particular change in their routine, but during the past 2 days they have been outside playing tennis more than usual because of the onset of sunny, warm weather. While in the ED, the patient has a generalized tonic-clonic seizure lasting 2 minutes. She is 163 cm (5 ft 4 in) tall and weighs 72 kg (160 lb); BMI is 27 kg/m^2. Pulse is 104/min, respirations are 18/min, and blood pressure is 140/90 mm Hg. Examination shows dry mucous membranes. ECG shows a second-degree atrioventricular block. Which of the following is most likely responsible for this patient's confusion?

(A) Acetaminophen
(B) Bupropion
(C) Lithium
(D) Risperidone
(E) Topiramate

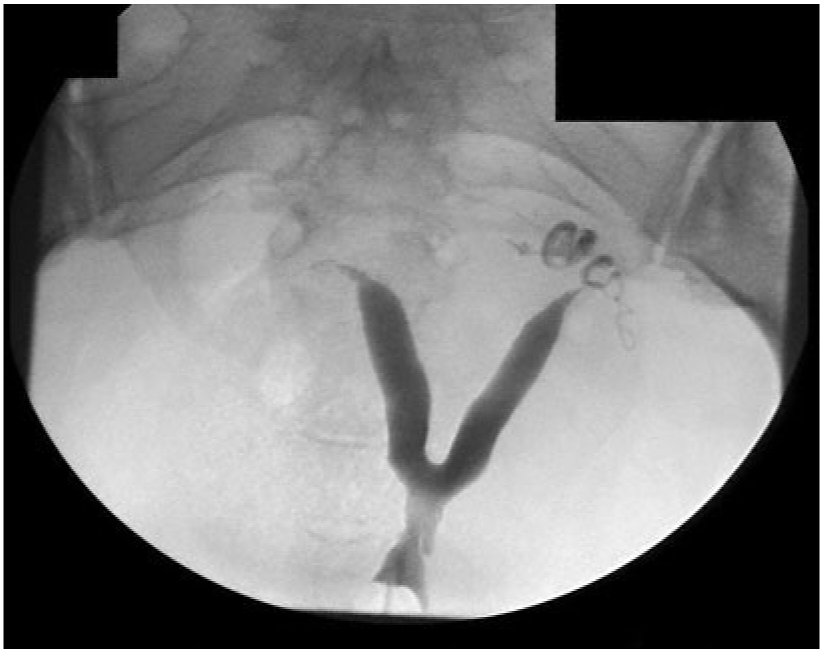

44. A 32-year-old nulligravid woman comes to the office because she has been unable to conceive during the past 12 months. She and her husband have unprotected sexual intercourse three times weekly. Two years ago, she was involved in a motor vehicle collision; CT scan at that time showed absence of the left kidney. She has no other history of serious illness, and her only medication is a prenatal vitamin. She has no known allergies. Vital signs are within normal limits. Pelvic examination shows no abnormalities. Hysterosalpingography is shown. When this patient conceives, she is at greatest risk for which of the following pregnancy complications?

(A) Fetal macrosomia
(B) Multiple gestation
(C) Oligohydramnios
(D) Preeclampsia
(E) Preterm labor

45. **Patient Information**

Age: 14 years
Gender: F, self-identified
Ethnicity: unspecified
Site of Care: office

History

Reason for Visit/Chief Concern: "I feel light-headed."
History of Present Illness:
• 1-week history of episodes of light-headedness
• almost lost consciousness during three episodes
• experienced tunnel vision and almost fell when getting out of bed this morning
• evaluated 10 days ago for follow-up of attention-deficit/hyperactivity disorder; clonidine therapy initiated at that time
• sister is currently hospitalized for meningitis; patient has been receiving prophylactic rifampin
Past Medical History:
• moderate persistent asthma that is well controlled
• last asthma exacerbation was 1 week ago; 5-day course of prednisone was initiated at that time, which she completed
• attention-deficit/hyperactivity disorder diagnosed at age 8 years; has been on numerous different medications

Medications:
• albuterol
• fluticasone
• methylphenidate
• clonidine
• rifampin
Vaccinations:
• up-to-date
Allergies:
• no known drug allergies
Family History:
• mother and father: alive with no chronic conditions
• sister: currently being treated for meningitis
Psychosocial History:
• lives with parents, 18-year-old sister, and 2-year-old sister
• has difficulty in school because of attention-deficit/hyperactivity disorder
• does not smoke cigarettes, drink alcoholic beverages, or use nonprescribed medications or other substances
• not sexually active

Physical Examination

Temp	Pulse	Resp	BP	O_2 Sat	Ht	Wt	BMI
37.0°C (98.6°F)	90/min	20/min	115/70 mm Hg (lying down) 95/55 mm Hg (after standing for 5 min)	99% on RA	155 cm (5 ft 1 in) 25th %ile	42 kg (93 lb) 10th %ile	17.5 kg/m^2 25th %ile

• Appearance: sitting in a chair; comfortable, not in acute distress
• Skin: no rashes
• Pulmonary: clear to auscultation
• Cardiac: regular rhythm; no murmurs; capillary refill time of <2 seconds
• Abdominal: nondistended; normoactive bowel sounds; soft, nontender
• Neurologic: alert; cranial nerves intact

Question: An adverse effect of which of the following medications is the most likely cause of this patient's current condition?

(A) Albuterol
(B) Clonidine
(C) Methylphenidate
(D) Prednisone
(E) Rifampin

46. A 51-year-old woman comes to the office for a health maintenance examination. She feels well. She has no history of serious illness and takes no medications. She smoked one pack of cigarettes daily for 28 years but quit 6 years ago. Pulse is 80/min, respirations are 12/min, and blood pressure is 122/78 mm Hg. On examination, pedal pulses are absent bilaterally. A femoral bruit is heard in the left lower extremity. Which of the following is the most appropriate next step in management?

(A) Pentoxifylline therapy
(B) Peripheral artery catheterization
(C) Serum lipid studies
(D) Warfarin therapy
(E) No further management is indicated

47. A 47-year-old woman comes to the office for a follow-up examination. Three days ago, she was brought to the emergency department because of cellulitis of her right lower extremity, and trimethoprim-sulfamethoxazole therapy was begun. She has had no adverse reaction to the medication. She has kept her leg elevated, and her cellulitis has improved moderately. She also has hypertension, gout, and congenital heart disease. Her other medications are lisinopril, allopurinol, and warfarin. Five years ago, she underwent mechanical mitral valve replacement. Temperature today is 36.7°C (98.0°F), pulse is 72/min, and blood pressure is 128/83 mm Hg. Examination shows a 4 × 5-cm, erythematous, warm rash over the right lower extremity. There is no red streaking, vesicles, or purulence. There is no rash elsewhere. Pulses in the lower extremities are intact. Lungs are clear to auscultation. On cardiac examination, a mechanical click is heard during S_1. A grade 2/6 holosystolic murmur is heard best at the apex. The remainder of the examination shows no abnormalities. Results of laboratory studies are shown:

Blood	
Hematocrit	35%
WBC	13,000/mm^3
Platelet count	357,000/mm^3
PTT	38 sec
PT	57 sec (INR=5.2)

Which of the following actions is most likely to have prevented the near miss in this patient?

(A) Review for potential drug-drug interaction
(B) Dietary counseling
(C) Echocardiography
(D) Venous duplex ultrasonography of the right lower extremity
(E) Discontinuation of allopurinol

48. A 58-year-old woman comes to the office for a routine examination. She has type 2 diabetes mellitus. During the past 3 months, her fingerstick blood glucose concentrations have ranged from 80 mg/dL to 230 mg/dL; previously, they ranged from 80 mg/dL to 140 mg/dL. Medications are metformin and aspirin. During the past 3 months, she has exercised three times weekly; previously, she exercised once weekly. She follows a balanced diet. Despite exercise and diet, she has not had weight loss. She is 157 cm (5 ft 2 in) tall and weighs 72 kg (160 lb); BMI is 29 kg/m^2. Examination shows a 1 × 2-cm, erythematous, soft lesion over the posterior plantar aspect of the left foot. Sensation to pinprick and touch is decreased over the ankles and feet. Hemoglobin A_{1c} is 7%. Which of the following findings are most likely to be present in this patient?

	**Glucose**	**Insulin**	**Insulin Receptor Responsiveness**
(A)	Increased	increased	decreased
(B)	Increased	increased	normal
(C)	Increased	normal	decreased
(D)	Increased	normal	normal
(E)	Normal	normal	normal

49. A 57-year-old woman comes to the emergency department because of a 2-day history of fever, chills, and sore throat. She is on day 6 of a 7-day course of trimethoprim-sulfamethoxazole for treatment of cystitis. She has been taking lisinopril nightly for 2 years for treatment of hypertension. She has no other history of serious illness and takes no other medications. Temperature is 38.6°C (101.4°F), pulse is 84/min, respirations are 16/min, and blood pressure is 118/76 mm Hg. Examination shows erythema of the pharynx but no exudates. No other abnormalities are noted. Results of laboratory studies are shown:

Blood	
Hematocrit	38%
WBC	2200/mm^3
Neutrophils, segmented	16%
Neutrophils, bands	4%
Lymphocytes	64%
Monocytes	16%
Platelet count	225,000/mm^3

Which of the following is the most likely explanation for these findings?

(A) Acute mononucleosis
(B) Acute myelogenous leukemia
(C) Adverse effect of trimethoprim-sulfamethoxazole
(D) Allergic reaction to lisinopril
(E) Myelofibrosis
(F) Sepsis syndrome

50. A 39-year-old man is brought to the emergency department because of a 3-hour history of moderate pain and swelling of his right knee and ankle. His symptoms began after he scaled a wall during an obstacle course. He landed awkwardly on his right leg and rolled his right ankle; his knee buckled. He limped to the finish line. He underwent reconstruction of a torn right anterior cruciate ligament 15 years ago. He and his boyfriend are engaged to be married. He feels safe at home. He does not appear to be in distress. Examination of the right knee shows mild tenderness to palpation of the midsagittal joint line. Adduction and abduction stress testing is stable at 0 and 30 degrees of flexion. Range of motion is full. Lachman test is negative. There is moderate tenderness to palpation of the distal aspect of the fibula. There are ecchymoses over the anterolateral aspect of the right ankle. The ankle is swollen. There is no tenderness to palpation of the medial aspect of the ankle. Range of motion of the ankle is limited to 5 degrees of dorsiflexion and 30 degrees of plantar flexion because of pain. Which of the following is the most appropriate next step in diagnosis?

	**Knee**	**Ankle**
(A)	X-ray	x-ray
(B)	X-ray	no diagnostic testing indicated
(C)	No diagnostic testing indicated	x-ray
(D)	No diagnostic testing indicated	no diagnostic testing indicated

51. A 13-year-old girl is brought to the office for evaluation of short stature. Since birth, she has been below the 3rd percentile for height and at the 3rd percentile for weight. She has never had a menstrual period. There is no family history of short stature, but her maternal uncle did not begin puberty until the age of 16 years. Her current height is consistent with that of a 9½-year-old girl. Pulse is 82/min and blood pressure is 110/70 mm Hg. Sexual maturity rating is stage 1 for breast development and stage 2 for pubic hair development. Which of the following is the most appropriate next step in diagnosis?

(A) Complete blood count and serum chemistry panel
(B) Measurement of urine free cortisol concentration
(C) X-rays of the left hand and wrist
(D) X-rays of the long bones and spine
(E) X-ray of the skull

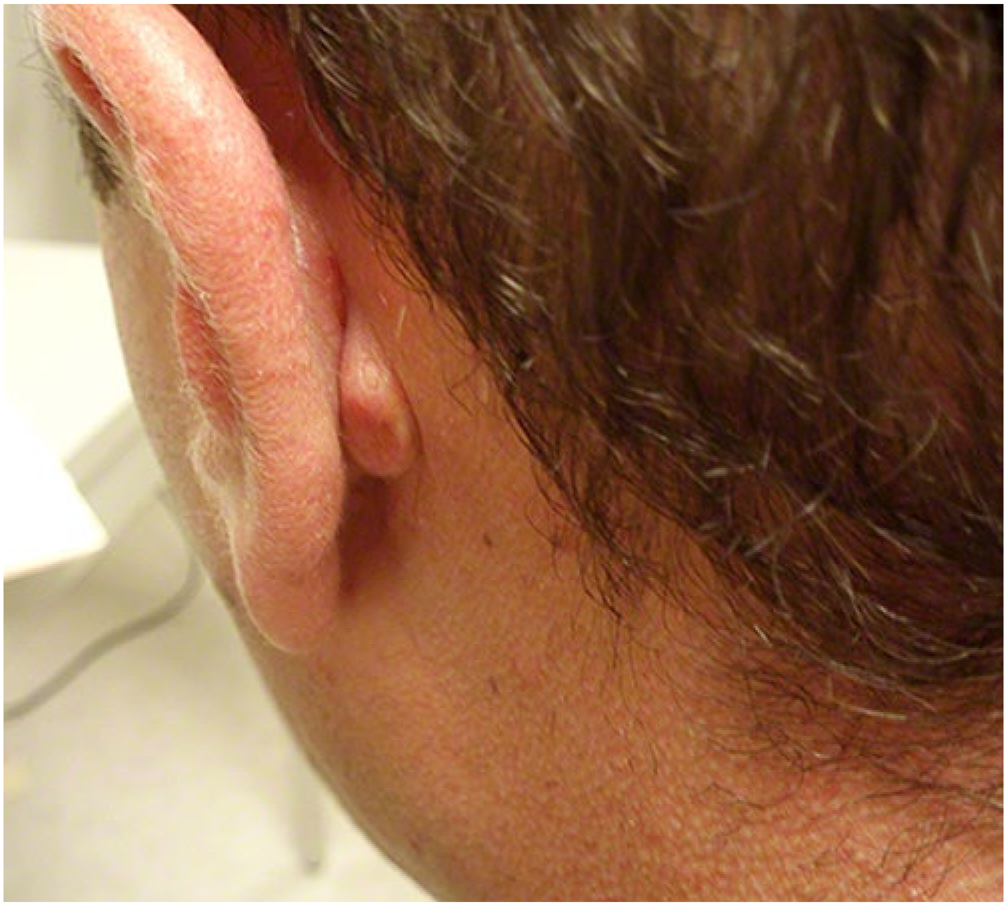

52. A 20-year-old woman comes to the office because of a 1-year history of a lump on her left ear that appeared 2 months after she had the ear pierced. Physical examination shows the findings in the photograph. Which of the following is the most appropriate initial treatment for this patient?

(A) Corticosteroid injection
(B) Cryotherapy
(C) Laser ablation
(D) Needle aspiration
(E) Wide excision

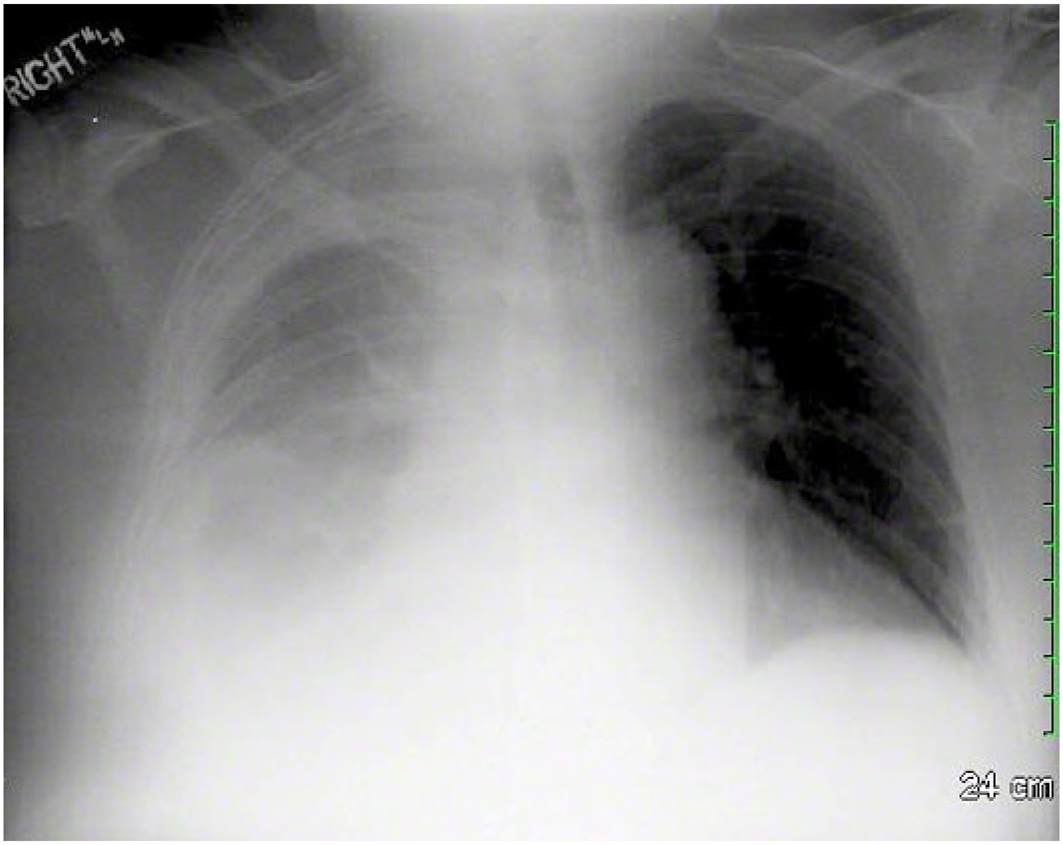

53. A 42-year-old woman is brought to the emergency department by ambulance 20 minutes after a head-on motor vehicle collision in which she was the restrained driver. On arrival, she is alert and reports chest and abdominal pain. Medical history is unremarkable and she takes no medications. Temperature is 36.7°C (98.0°F), pulse is 120/min, respirations are 28/min, and blood pressure is 130/80 mm Hg. Pulse oximetry on 4 L/min of oxygen via nasal cannula shows an oxygen saturation of 90%. Breath sounds are decreased on the right. Trachea is in the midline position. Cardiac examination discloses no abnormalities. An abrasion consistent in shape with a seat belt is present over the right upper abdominal quadrant; the area is tender to palpation. Radial and dorsalis pedis pulses are normal bilaterally. Results of laboratory studies are shown:

Arterial blood gas analysis on oxygen at 4 L/min	
P_{O_2}	65 mm Hg
P_{CO_2}	32 mm Hg
pH	7.38
Blood	
Hemoglobin	10.0 g/dL

Chest x-ray is shown. In addition to intravenous analgesic therapy, which of the following is the most appropriate next step in management?

(A) Bronchoscopy
(B) Intubation and mechanical ventilation
(C) Placement of a thoracic epidural
(D) Thoracentesis
(E) Thoracotomy
(F) Tube thoracostomy
(G) Observation only

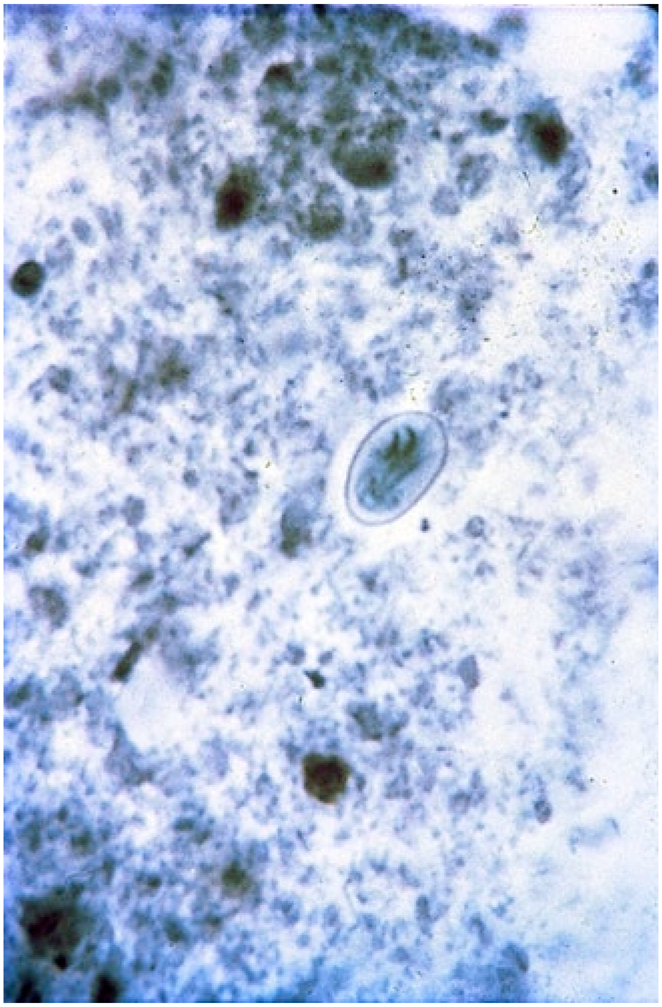

54. A 31-year-old woman comes to the office because of a 3-month history of intermittent nonbloody diarrhea. The first episode began 5 days after a camping trip in rural Virginia with her family. During the trip, she drank from a spring-fed pond that she says was “clean.” During the past 3 weeks, she also has had a 2.3-kg (5-lb) weight loss despite no change in appetite. She has not had fever or other gastrointestinal symptoms. She has no history of serious illness and takes no medications. She is sexually active with one female partner. The patient is 163 cm (5 ft 4 in) tall and weighs 54 kg (119 lb); BMI is 20 kg/m^2. Temperature is 37.9°C (100.2°F), pulse is 85/min and regular, respirations are 12/min, and blood pressure is 115/70 mm Hg. Abdominal examination shows mild, diffuse tenderness to deep palpation; bowel sounds are increased. Test of the stool for occult blood is negative. Results of a stool smear are shown. Which of the following is the most likely infectious agent?

(A) *Campylobacter jejuni*
(B) *Clostridioides difficile*
(C) *Entamoeba histolytica*
(D) *Escherichia coli*
(E) *Giardia lamblia*
(F) *Salmonella enteritidis*
(G) *Shigella dysenteriae*

55. A randomized placebo-controlled clinical trial is conducted to assess the effectiveness of a selective estrogen receptor modulator (SERM) for chemoprophylaxis of breast cancer. A subset of the results shows:

	SERM	Placebo
Number of participants	2400	1200
Woman-years of follow-up	7200	3600
Invasive breast cancer	7	13
Breast cancer rate (per 1000 woman-years)	1	3.6
Estrogen receptor status (invasive cancers)		
Positive	2	10
Negative	4	2
Unknown	1	1

Which of the following represents the absolute risk reduction for invasive breast cancer per 1000 woman-years in those treated with a SERM?

(A) 0.28
(B) 2.6
(C) 3.6
(D) 6
(E) 7

Items #56–57 are part of a sequential item set. In the actual examination environment, you will not be able to view the second item until you click "Proceed to Next Item." After navigating to the second item, you will not be able to add or change an answer to the first item.

A 57-year-old woman comes to the emergency department 1 hour after vomiting bright red blood. She thinks it was approximately 1 cup of blood. She reports that during the past month, she has felt full sooner than usual while eating. She has not had other episodes of vomiting and has not had weight loss. She has no history of operative procedures. She has hypertension and type 2 diabetes mellitus. Medications are hydrochlorothiazide and metformin. She has not taken aspirin or nonsteroidal anti-inflammatory drugs during the past month. She does not smoke cigarettes, drink alcohol, or use other substances. Temperature is 37.0°C (98.6°F), pulse is 116/min, respirations are 16/min, and blood pressure is 110/78 mm Hg. Examination shows mild tenderness to palpation in the midepigastric area; there is no rebound tenderness. Test of the stool for occult blood is positive. Hematocrit is 31%, platelet count is 240,000/mm^3, and prothrombin time is 13 seconds (INR=1.2).

56. In addition to intravenous administration of fluids and nasogastric tube lavage, which of the following is the most appropriate next step in management?

(A) Abdominal CT scan
(B) Octreotide scan
(C) Technetium 99m scan
(D) Colonoscopy
(E) Esophagogastroduodenoscopy
(F) Mesenteric angiography

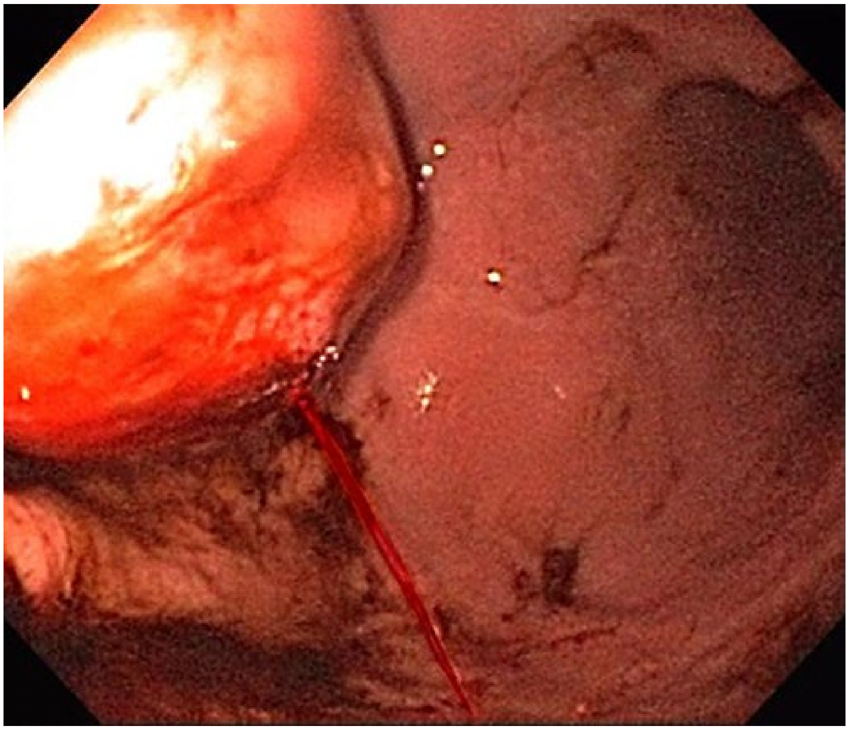

57. After intravenous administration of fluids and nasogastric tube lavage, the patient undergoes esophagogastroduodenoscopy. A photograph of the endoscopic findings is shown. Which of the following is the most appropriate next step in management?

(A) Octreotide therapy
(B) Omeprazole therapy
(C) Tyrosine kinase inhibitor therapy
(D) Endoscopic hemostatic therapy
(E) Endoscopic biopsy
(F) Endoscopic resection

END OF SET

---

58. A 77-year-old woman who resides in a skilled nursing care facility is examined by the physician 1 day after staff noticed some blood on her bedsheets. She sustained a cerebral infarction 8 years ago and has been confined to bed since then because of residual left hemiparesis. She is currently taking warfarin. She is 160 cm (5 ft 3 in) tall and weighs 50 kg (110 lb); BMI is 20 kg/m^2. Temperature is 36.0°C (96.8°F), pulse is 70/min, respirations are 18/min, and blood pressure is 180/90 mm Hg. There is no lymphadenopathy. Examination shows a small, clean, decubitus ulcer on the sacrum. Pelvic examination shows a 3-cm, raised, fleshy lesion on the right labium majus. Vaginal examination cannot be performed because of the patient's discomfort. Which of the following is the most appropriate next step in management?

(A) Cytologic evaluation of the vulva
(B) CT scan of the abdomen and pelvis
(C) Application of an antifungal cream
(D) Application of a corticosteroid cream
(E) Colonoscopy
(F) Biopsy of the vulva

59. A 68-year-old man comes to the office because of a 3-month history of moderate low back pain that extends into both legs. He first noticed the pain while standing in the ticket line at a theater. At that time, the pain was accompanied by tingling in both legs. The symptoms recurred a few days later as he was standing in the grocery checkout line. The pain has not responded to ibuprofen or naproxen but improves with sitting or lying down. Prior to onset of the pain, the patient typically walked for 30 minutes four times weekly. He discontinued his walking regimen 2 months ago because it seemed to trigger his back pain. He has hypertension and hyperlipidemia. Medications are hydrochlorothiazide-triamterene, simvastatin, and aspirin. The patient has smoked one pack of cigarettes daily for 40 years. Pulse is 74/min and blood pressure is 140/90 mm Hg. Lungs are clear to auscultation; expiration phase is delayed. On cardiac examination, a grade 2/6 systolic crescendo-decrescendo murmur is heard at the upper right sternal border. There is trace ankle edema bilaterally; ankle brachial index is 1.0. Musculoskeletal and neurologic examinations show no abnormalities. Which of the following is the most likely diagnosis?

(A) Abdominal aortic aneurysm
(B) Osteoporosis
(C) Peripheral neuropathy
(D) Spinal stenosis
(E) Vascular claudication

60. A 50-year-old man comes to the office because of a 3-day history of mild headache and intermittent confusion. He has a 30-year history of schizophrenia and a 5-year history of emphysema. Medications are clozapine, sertraline, and inhaled beclomethasone and salmeterol. He also receives home oxygen therapy. He smoked two packs of cigarettes daily for 33 years but quit 5 years ago. Temperature is 36.8°C (98.2°F), respirations are 20/min, pulse is 90/min, and blood pressure is 115/70 mm Hg. Examination shows moist mucous membranes. Lungs are clear to auscultation. There is no peripheral edema. Neurologic examination shows no focal findings. Results of a complete blood count are within the reference ranges. Results of other laboratory studies are shown:

Serum	
Na^+	116 mEq/L
K^+	4 mEq/L
Cl^-	90 mEq/L
Urea nitrogen	10 mg/dL
Creatinine	1 mg/dL
Glucose	100 mg/dL

Which of the following is the most appropriate next step in management?

(A) CT scan of the chest
(B) Measurement of serum cortisol and TSH concentrations
(C) Measurement of serum uric acid concentration
(D) Measurement of urine and plasma osmolarity
(E) MRI of the brain

61. A 67-year-old man is evaluated in the intensive care unit. He has end-stage pancreatic cancer and was hospitalized 3 days ago for treatment of pneumonia. Respirations are 6/min. Pulse oximetry on 100% oxygen by face mask shows an oxygen saturation of 78%. Examination shows feeble respiratory efforts; he is using accessory muscles of respiration. On mental status examination, the patient is oriented to person but not to place or time. If the patient is not endotracheally intubated and mechanically ventilated, he will die within hours. His wife says the patient recently told her that he would never want mechanical ventilation, but they never completed paperwork regarding his wishes. His daughter insists that he be mechanically ventilated. Which of the following is the most appropriate action for the physician to take?

(A) Perform endotracheal intubation and begin mechanical ventilation
(B) Perform endotracheal intubation and then consult the hospital ethics committee regarding mechanical ventilation
(C) Perform endotracheal intubation only
(D) Provide palliative therapy only
(E) Seek a court order to assign a legal guardian

62. A 5-day-old boy is brought to the office for an initial well-child examination. He was born at 40 weeks' gestation and discharged at 60 hours of life. On newborn screening, hemoglobin electrophoresis showed an FS pattern. He is at the 50th percentile for length and weight. Temperature is 37.0°C (98.6°F), pulse is 136/min, and respirations are 34/min. He appears well. Examination shows no abnormalities. Which of the following is the most appropriate next step in management?

(A) Deferoxamine therapy
(B) Hydroxyurea therapy
(C) Iron supplementation
(D) Monthly blood transfusions
(E) Penicillin prophylaxis
(F) Vitamin B_{12} (cyanocobalamin) supplementation

63. A 1-week-old female newborn is brought to the office for a follow-up examination after newborn screening showed a serum TSH concentration of 40 µU/mL (N=1–20). She has been breast-feeding well. She passes five to six stools and has multiple wet diapers daily. She has returned to her birth weight. Vital signs are within normal limits. Examination discloses no abnormalities. Serum studies today show a TSH concentration of 14 µU/mL (N=0.5–6.5) and free thyroxine (FT_4) concentration of 0.4 ng/dL (N=0.9–2.2). Which of the following is the most appropriate next step in management?

(A) Hydrocortisone therapy
(B) Levothyroxine therapy
(C) Monthly serial measurement of serum thyroglobulin concentration
(D) Monthly serial measurement of serum TSH and free thyroxine (FT_4) concentrations
(E) Radioactive iodine uptake scan
(F) Ultrasonography of the thyroid gland

64. An 18-year-old patient comes to the emergency department because of a 2-day history of severe abdominal pain. The pain began in the middle of the abdomen after a large meal but now is localized to the right lower quadrant. The patient also has a 2-month history of intermittent diarrhea. Three weeks ago, the patient had an upper respiratory tract infection that resolved spontaneously. The patient currently takes no medications. The patient identifies as nonbinary and uses they/they pronouns; their assigned sex at birth was male. They do not smoke cigarettes or use other substances. Temperature is 37.8°C (100.0°F), pulse is 80/min, respirations are 20/min, and blood pressure is 110/70 mm Hg. Abdominal examination shows fullness and tenderness to palpation of the right lower quadrant. Leukocyte count is 11,000/mm^3. Which of the following is the most appropriate next step in diagnosis?

(A) Abdominal ultrasonography of the right lower quadrant
(B) Air-contrast barium enema
(C) CT scan of the abdomen
(D) Technetium 99m scan of the bowel
(E) Upper gastrointestinal series with small bowel follow-through

65. A 25-year-old woman, gravida 3, para 2, at 36 weeks' gestation comes to the emergency department because of heavy vaginal bleeding following sexual intercourse 3 hours ago. She has received no prenatal care. She has no history of serious illness or operative procedures. Vital signs are within normal limits. Continuous external fetal heart monitoring shows a baseline of 155/min with no accelerations or decelerations. The uterus is consistent in size with a 36-week gestation. Tocodynamometer monitoring shows contractions every 8 minutes. Which of the following is the most appropriate next step in management?

(A) Cesarean delivery now
(B) Digital examination of the cervix
(C) Intravenous administration of magnesium sulfate
(D) Intravenous administration of oxytocin
(E) Transabdominal ultrasonography

66. A 75-year-old man is admitted to the hospital because of a 3-day history of fever. He has type 2 diabetes mellitus, hypertension, and chronic obstructive pulmonary disease. One year ago, he had a stroke with residual right lower extremity hemiparesis. Medications are amlodipine, tiotropium, metformin, glimepiride, lisinopril, and aspirin. He does not drink alcoholic beverages and has smoked one pack of cigarettes daily for 50 years. The patient is widowed, lives alone, and has three adult children. He is not in acute distress. He is 178 cm (5 ft 10 in) tall and weighs 82 kg (180 lb); BMI is 26 kg/m^2. Temperature is 39.2°C (102.6°F), pulse is 110/min, respirations are 22/min, and blood pressure is 150/90 mm Hg. Pulse oximetry on room air shows an oxygen saturation of 96%. Scattered end-expiratory wheezes are heard. There is dense hemiparesis of the right lower extremity. Appropriate therapy is initiated, and 3 days later, the patient's condition has improved. He is awake, alert, and conversing with one of his sons about current events. Temperature is 37.2°C (99.0°F), pulse is 72/min, and blood pressure is 130/82 mm Hg. Discharge plans are discussed with the patient, his son, the physician, and a physical therapist. The physical therapist says the patient would be best cared for in a nursing care facility. The patient acknowledges that a nursing facility would provide better care for him but says he would prefer to remain at home with a visiting nurse. Once outside the patient's room, his son says, "I have his power of attorney. Don't listen to him. He needs to be in a nursing home." Which of the following is the most appropriate next step?

(A) Inform the son that the decision is the patient's to make, regardless of the medical team's recommendation
(B) Reassure the son that the patient will be admitted to a nursing care facility in accordance with the physical therapist's recommendation
(C) Refrain from making any decisions until a family meeting with the son and siblings is arranged to discuss the matter
(D) Request psychiatric evaluation of the patient's mental competency
(E) Request to review the legal documentation designating the son as having power of attorney

67. A 59-year-old man is brought to the emergency department because of a 1-hour history of weakness of his left arm and leg and mild headache. He says that, when he was gardening with his wife, he suddenly became unable to hold his gardening tool, lift his left arm, or walk. Two weeks ago, he had a nonproductive cough and nasal congestion that resolved spontaneously after 1 week. He has hypertension treated with lisinopril. On arrival, he is lethargic. He is 183 cm (6 ft) tall and weighs 97 kg (215 lb); BMI is 29 kg/m^2. Temperature is 36.6°C (97.9°F), pulse is 94/min, respirations are 16/min, and blood pressure is 174/109 mm Hg. The pupils measure 3 mm and are reactive to light. Muscle movement is decreased over the left lower aspect of the face. Muscle strength is 0/5 in the left extremities. Deep tendon reflexes are absent in the left extremities and 2+ in the right extremities. CT scan of the head shows a large area of increased attenuation in the right putamen region. Which of the following is the most likely cause of this patient's condition?

(A) Amyloid angiopathy
(B) Embolism
(C) Small vessel disease
(D) Vasculitis
(E) Venous thrombosis

68. A 24-year-old primigravid woman at 10 weeks' gestation comes to the office for a scheduled dilatation and suction curettage. Ultrasonography 2 days ago showed a fetus consistent in size with a 9-week gestation with absent cardiac activity. Two weeks ago, the patient had an upper respiratory tract infection that resolved spontaneously. One year ago, she was diagnosed with herpes simplex virus (HSV) 2; her last outbreak was 6 months ago. She has no other history of serious illness. She is 163 cm (5 ft 4 in) tall. She weighed 82 kg (180 lb) prior to her pregnancy; BMI was 31 kg/m^2. She has had a 3.6-kg (8-lb) weight gain during her pregnancy. Vital signs are within normal limits. The vulvar area is shaven; no lesions are noted. Examination shows a normal-appearing cervix. The uterus is nontender and consistent in size with an 8-week gestation. There is homogenous white-gray vaginal discharge; the pH of the discharge is 5.5. She undergoes dilatation and suction curettage with sterile instruments and povidone-iodine vaginal cleansing. Which of the following is the strongest predisposing risk factor for postoperative infection in this patient?

(A) History of upper respiratory tract infection
(B) HSV 2
(C) Obesity
(D) Type of vaginal discharge
(E) Vulvar shaving

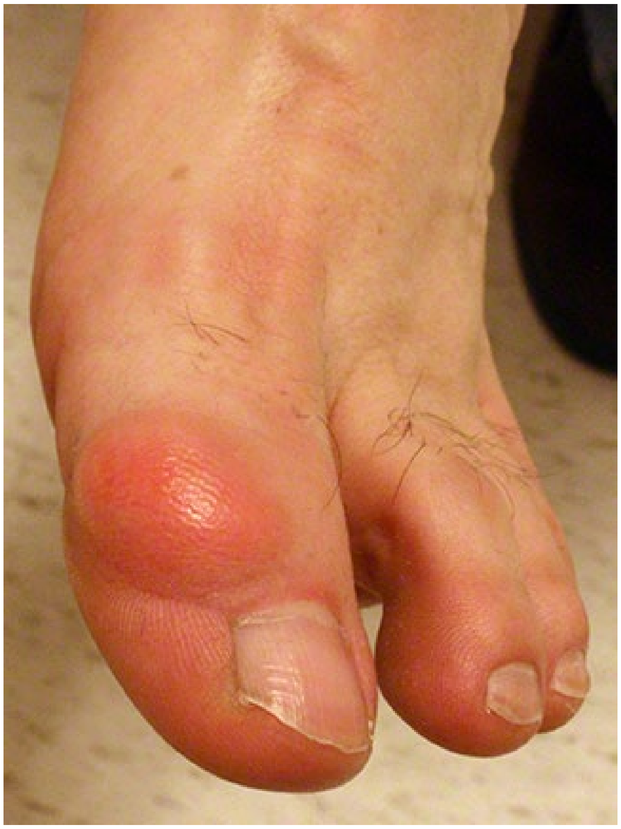

69. A 74-year-old woman comes to the clinic 2 days after the sudden onset of severe left foot pain. She has had difficulty walking during this time and has been using a cane. She has not sustained trauma to the foot. She has type 2 diabetes mellitus and hypertension. Medications are metformin, insulin glargine, hydrochlorothiazide, lisinopril, and atorvastatin. She typically wears support stockings because of lower leg swelling, but the foot pain has made it difficult for her to wear them recently. She is a traveling pastor and spends more than 4 hours daily in her car. She has been monogamous with her wife for the past 30 years. Blood pressure is 146/88 mm Hg; other vital signs are within normal limits. A photograph of the left great toe is shown. No other abnormalities are noted. Hemoglobin A_{1c} is 7.4%, serum creatinine concentration is 1.4 mg/dL, and serum uric acid concentration is 7.8 mg/dL. Results of serum electrolyte concentrations are within the reference ranges. Which of the following is the most likely diagnosis?

(A) Cellulitis
(B) Gout
(C) Infected mucoid cyst
(D) Osteoarthritis
(E) Pseudogout
(F) Rheumatoid arthritis

---

70. A 47-year-old man comes to the office for a routine examination before beginning an exercise program. He feels well. He has a 25-year history of type 1 diabetes mellitus. A grade 1/6, early peaking, systolic ejection murmur is heard best at the left third intercostal space. Sensation to light touch is mildly decreased over the toes. The remainder of the examination shows no abnormalities. An exercise stress test shows a 3-mm ST-segment depression early in the test. The test does not produce any pain. Which of the following is the most likely diagnosis?

(A) Cardiomyopathy
(B) Congenital heart disease
(C) Coronary artery disease
(D) Valvular heart disease
(E) Normal cardiac findings

71. A 22-year-old man comes to the office for a health maintenance examination. He feels well and has not noticed any health issues. He is in the US Army. Two months ago, he returned from a 10-month deployment to Southwest Asia, during which he started smoking one pack of cigarettes daily. The patient says he is happy to be home and has been celebrating with his friends and family. He has been consuming an average of six beers daily since returning to the United States. He has no history of major medical illness. He does not use any medications or other substances. His mother has type 2 diabetes mellitus. The patient is 180 cm (5 ft 11 in) tall and weighs 72 kg (160 lb); BMI is 22 kg/m^2. His first blood pressure reading is 156/95 mm Hg; repeat reading is 160/92 mm Hg. He says he remembers his blood pressure being normal when he had it checked in the past. Physical examination discloses no other abnormalities. Which of the following is the most likely cause of this patient's increased blood pressure?

(A) Cigarette smoking
(B) Essential hypertension
(C) Excessive alcohol use
(D) Pheochromocytoma
(E) Renal artery stenosis

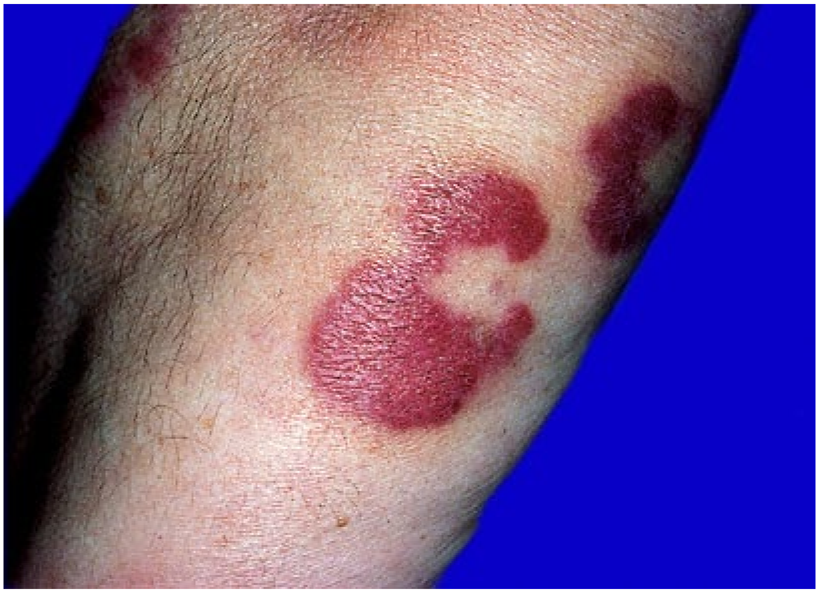

72. A 32-year-old man comes to the office because of a 3-month history of a painless rash over his arms. During the past month, he also has had a 3.2-kg (7-lb) weight loss despite no change in appetite. He has no history of serious illness and takes no medications. He is 175 cm (5 ft 9 in) tall and weighs 72 kg (158 lb); BMI is 23 kg/m^2. Pulse is 98/min; other vital signs are within normal limits. Lungs are clear to auscultation. Cardiac examination shows no abnormalities. A photograph of the left upper extremity is shown. Similar findings are noted over the right upper extremity. Which of the following is the most likely cause of this patient's dermatologic findings?

(A) Actinic keratosis
(B) Dermatitis herpetiformis
(C) Kaposi sarcoma
(D) Lichen planus
(E) Seborrheic dermatitis

73. A 42-year-old nulligravid woman comes to the office because of a 6-month history of hot flashes and intermittent palpitations. Her last menstrual period was 6 months ago. Menses previously occurred at regular 29-day intervals but gradually had occurred less frequently during the past 2 years. She underwent ovarian cystectomy 13 years ago for a small benign teratoma. She used an oral contraceptive for 12 years until tubal ligation 6 years ago. Her mother and sister had menopause at the ages of 47 and 49 years, respectively. The patient has smoked one pack of cigarettes daily for 26 years. She is sexually active and monogamous with one male partner. She is 170 cm (5 ft 7 in) tall and weighs 86 kg (191 lb); BMI is 30 kg/m^2. Pulse is 82/min and blood pressure is 130/85 mm Hg. Physical examination, including pelvic examination, discloses no abnormalities. Which of the following is the most appropriate next step in diagnosis?

(A) Measurement of serum follicle-stimulating hormone concentration
(B) Measurement of serum luteinizing hormone concentration
(C) Measurement of serum testosterone concentration
(D) ECG
(E) Pelvic ultrasonography

74. A colleague of a 47-year-old physician who is conducting patient evaluations during afternoon walk-in clinic hours notices that the physician appears groggy, has slurred speech, and smells of alcohol. Earlier in the day, the physician had attended a celebratory lunch for the graduation of one of the clinic's employees from nursing school. The colleague has not witnessed similar findings in the physician in the past. The physician is scheduled to work at the clinic for the next 4 hours. A physician assistant is present at the clinic and typically aids the physician with evaluating patients. Which of the following is the most appropriate action for the colleague to take at this time?

(A) Allow the physician to finish his time at the clinic, but raise concern about the episode at the next physicians' staff meeting
(B) Ask the office administrator to cancel the remainder of the clinic hours
(C) Ask the physician assistant to examine the physician's patients and discharge them before the physician has a chance to examine them
(D) Confront the physician and ask him to leave the clinic
(E) Report the physician to the clinic administrator and conduct the remainder of the clinic hours without him
(F) Report the physician to the state medical board

75. A 68-year-old woman returns to the office for a follow-up examination. One month ago, urinalysis showed protein during work-up of a suspected urinary tract infection. At that time, the patient was prescribed a 3-day course of oral cephalexin. Today, she says her previous pain on urination and urinary frequency have resolved. She has major depressive disorder and osteoarthritis. Current medications are venlafaxine and acetaminophen. Temperature is 37.0°C (98.6°F), pulse is 66/min, respirations are 18/min, and blood pressure is 122/66 mm Hg. The patient appears physically fit. Examination discloses no abnormalities. Repeat urinalysis is shown:

Specific gravity	1.015 (N=1.003–1.029)
pH	6.0 (N=4.5–7.8)
Protein	negative
Blood	negative
Leukocyte esterase	negative
Nitrite	negative
WBCs	3/hpf
RBCs	1/hpf

Which of the following is the most appropriate additional diagnostic study at this time?

(A) Determination of urine albumin:creatinine ratio
(B) 24-Hour urine collection for measurement of protein concentration
(C) Ultrasonography of the kidneys
(D) Urine sulfosalicylic acid method
(E) No additional testing is indicated

76. A 22-year-old man is brought to the emergency department because of severe right lower leg pain since he fell in the woods during a camping trip 2 days ago. The pain has worsened during the past 12 hours. He sustained a small scratch below his knee as a result of the fall. He has no history of serious illness and uses no medications or other substances. He does not drink alcohol. Pulse is 110/min, respirations are 14/min, and blood pressure is 105/60 mm Hg. Examination of the right lower extremity shows edema, diffuse cellulitis, and hemorrhagic bullae from the knee to the ankle. No abnormalities of the left lower extremity are noted. Peripheral intravenous antibiotic therapy and infusion of 2 L of crystalloid fluid are begun. Which of the following is the most appropriate next step in management?

(A) Fine-needle aspiration of the bullae
(B) Hyperbaric oxygen therapy
(C) MRI of the right lower extremity
(D) Surgical debridement
(E) Hospital admission for observation only

77. A 27-year-old woman, gravida 3, para 2, at 12 weeks' gestation comes to the office for her first prenatal visit. Her first child was delivered at 41 weeks' gestation and weighed 4167 g (9 lb 3 oz). Her second child was delivered at 37 weeks' gestation and weighed 4309 g (9 lb 8 oz). She has no history of serious illness and her only medication is a prenatal vitamin. Her sister has a history of delivering a stillborn infant. Her mother and father both have type 2 diabetes mellitus. Eight months ago, the patient immigrated to the United States from Mexico. The patient is 157 cm (5 ft 2 in) tall. She weighed 95 kg (210 lb) prior to her pregnancy; BMI was 38 kg/m^2. She has had a 3-kg (6.6-lb) weight gain during her pregnancy. Pulse is 80/min, respirations are 14/min, and blood pressure is 110/60 mm Hg. Bimanual examination shows a uterus consistent in size with a 12-week gestation. Ultrasonography confirms a 12-week gestation. Which of the following is the most appropriate next step in management?

(A) Antiphospholipid antibody screening
(B) Chest x-ray
(C) Fasting glucose tolerance test
(D) Measurement of serum α-fetoprotein concentration
(E) Measurement of serum free thyroxine concentration

78. An 84-year-old woman comes to the office because of a 1-month history of malaise. She has not had fever, weight loss, or abdominal pain. She has type 2 diabetes mellitus well controlled with long-acting insulin and metformin. She has missed multiple appointments during the past 6 months with no reasons given. Her hemoglobin A_{1c} 6 months ago was 6.7%. She says she has been "feeling fine." Her son moved back into her home 9 months ago because he had lost his job. The patient's husband died 5 years ago, and she has no other children. She appears unkempt. She is alert and fully oriented. She is 163 cm (5 ft 4 in) tall and weighs 77 kg (170 lb); BMI is 29 kg/m^2. Her pulse is 80/min and regular, respirations are 18/min, and blood pressure is 160/94 mm Hg. Mucous membranes are dry; there is no thrush. The skin is dry and intact. The remainder of the physical examination shows no abnormalities. Mental status examination shows no memory deficit. She has a normal mood and flat affect. Her fasting serum glucose concentration is 380 mg/dL. Results of urinalysis are shown:

Glucose	4+
Ketones	none
Nitrites	none
Leukocyte esterase	none

Which of the following is the most likely diagnosis?

(A) Dietary indiscretion
(B) Elder neglect
(C) Major depressive disorder
(D) Pancreatic malignancy
(E) Urinary tract infection

79. A 15-year-old boy is brought to the office 2 hours after he awoke with weakness of the right side of his face. He has a 2-week history of intermittent headache, sensitivity to light, and mild to moderate neck pain. He has no history of serious illness. His only medication is acetaminophen as needed. He lives in New Jersey and has not traveled outside of the state during the past 6 months. Temperature is 37.4°C (99.3°F), pulse is 78/min and regular, and blood pressure is 108/70 mm Hg. Flexion of the neck elicits moderate pain and resistance. The right eyelids do not close completely. Ocular movements are normal. There is no evidence of ptosis. The right side of the forehead does not wrinkle when the patient looks up. There is severe weakness of the right corner of the mouth. There are no abnormalities of the left side of the face. Muscle strength in the jaw, tongue, and palate is normal and symmetric. There is no dysarthria. Muscle strength is 5/5 in all extremities. Coordination and sensation are intact. Gait is normal. A lumbar puncture is performed, and opening pressure is 180 mm H_2O. Cerebrospinal fluid analysis results are shown:

Glucose	60 mg/dL
Total protein	52 mg/dL
WBC	85/mm^3
Segmented neutrophils	30%
Lymphocytes	70%
RBC	1/mm^3

Which of the following is the most appropriate next step in diagnosis?

(A) Measurement of serum angiotensin-converting enzyme activity
(B) Measurement of serum Lyme (*Borrelia burgdorferi*) antibody concentration
(C) Polymerase chain reaction test for cytomegalovirus
(D) Serum antinuclear antibody assay
(E) Serum protein electrophoresis

80. A 2-year-old girl is brought to the emergency department because of loud breathing, harsh cough, and a hoarse cry since she awoke 1 hour ago. Her parents state that yesterday she had a runny nose. Medical history is unremarkable and she receives no medications. Temperature is 38.5°C (101.3°F), pulse is 120/min, respirations are 32/min, and blood pressure is 105/65 mm Hg. There are moderate suprasternal and subcostal retractions. Inspiratory stridor is heard at rest. Which of the following is the most appropriate initial pharmacotherapy?

(A) Intravenous dexamethasone
(B) Nebulized albuterol
(C) Nebulized budesonide
(D) Nebulized epinephrine
(E) Oral albuterol
(F) Oral prednisone
(G) Subcutaneous epinephrine

BLOCK 3, ITEMS 81–120

81. A 28-year-old man is admitted to the intensive care unit 3 hours after undergoing resection and anastomosis of a small-bowel injury and nephrectomy because of a right renal laceration sustained in a motor vehicle collision. He also has bilateral, minimally displaced pubic rami fractures. He is intubated and mechanically ventilated. He is receiving morphine and propofol. He responds only to painful stimuli. Pulse is 110/min, ventilatory rate is 12/min, and blood pressure is 133/68 mm Hg. The abdomen is distended and soft, and the incision is clean, dry, and intact. Since the operation, urine output had been 40 mL/h and bloody. During the past hour, urine output has been 5 mL and bloody. In addition to intravenous administration of 0.9% saline, which of the following is the most appropriate next step in management?

(A) CT scan of the abdomen and pelvis
(B) Repeat surgical exploration of the abdomen
(C) Transfusion of packed red blood cells
(D) Ultrasonography of the bladder

82. A 15-year-old girl is brought to the office because of a 3-day history of diffuse abdominal pain, fever, and vomiting. She has cerebral palsy, situs inversus, and severe scoliosis. She uses a wheelchair for ambulation. During previous visits, transferring this patient from her wheelchair to the examination table has been time-consuming and has caused the patient discomfort. Today, the patient appears uncomfortable. The physician decides to conduct the examination while the patient is sitting in her wheelchair. Temperature is 38.3°C (101.0°F), pulse is 110/min, respirations are 20/min, and blood pressure is 110/50 mm Hg. Pulse oximetry on room air shows an oxygen saturation of 100%. Examination shows mildly dry mucous membranes. The physician evaluated four previous patients today who had vomiting and fever; he diagnosed gastroenteritis in all four patients. The physician attributes this patient's increased pulse to mild dehydration and fever, and he diagnoses this patient with gastroenteritis. The physician prescribes ondansetron and tells the patient to return if she has intractable vomiting or if her abdominal pain worsens. Five hours later, the patient is taken to the emergency department after her mother finds her listless and barely responsive. Temperature is 38.9°C (102.0°F), pulse is 180/min, respirations are 35/min, and blood pressure is 70/50 mm Hg. Pulse oximetry on room air shows an oxygen saturation of 96%. CT scan of the abdomen shows volvulus, and emergency resection of necrotic bowel is done. Which of the following best describes the error that occurred?

(A) Latent error
(B) Near miss
(C) Non-preventable error
(D) Premature closure
(E) Systems failure

83. A 27-year-old man comes to the office because of a 1-month history of depressed mood and fatigue. He reports that it takes him at least 1 hour to fall asleep at night. He has had decreased concentration at work and decreased interest in socializing with his friends. He no longer exercises. He has epilepsy well controlled with levetiracetam. Physical examination discloses no abnormalities. On mental status examination, he has a sad mood and is briefly tearful. He has no suicidal ideation, hallucinations, or delusions. Which of the following is the most appropriate pharmacotherapy?

(A) Alprazolam
(B) Aripiprazole
(C) Bupropion
(D) Buspirone
(E) Mirtazapine

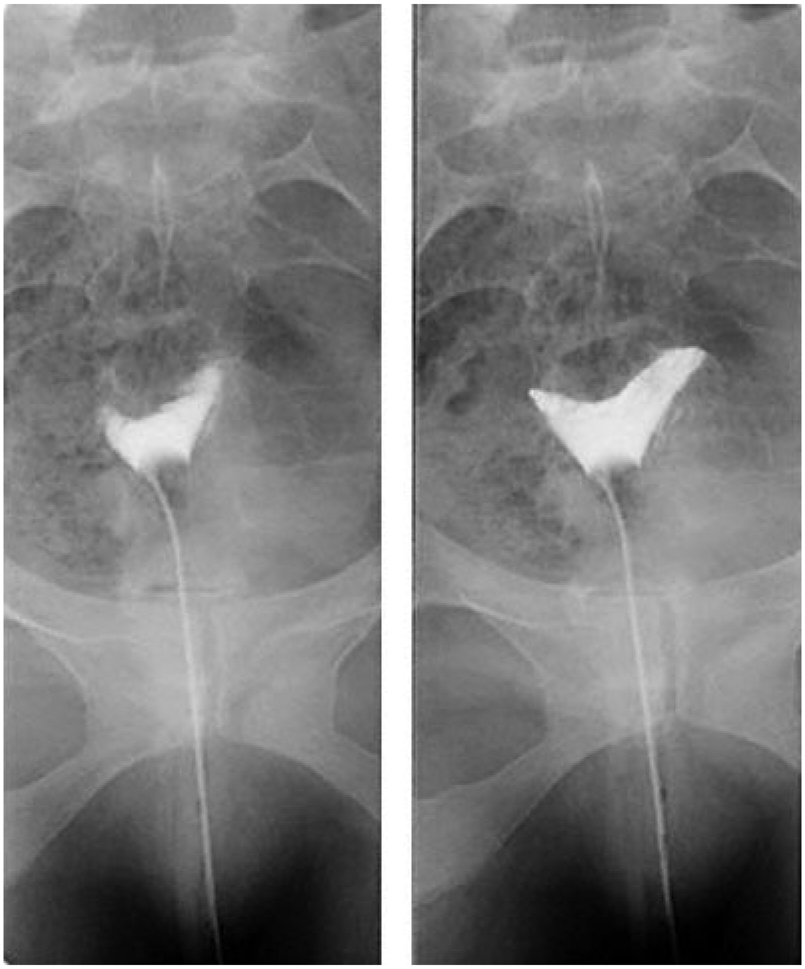

84. A 23-year-old nulligravid woman comes to the office because she has been unable to conceive during the past year. She and her husband have had regular unprotected intercourse during this time. The husband has no children. Prior to attempting to conceive, the patient regularly used depot medroxyprogesterone. Menses occur at regular 28-day intervals with occasional mid-cycle abdominal pain previously diagnosed as mittelschmerz. She had a ruptured appendix at age 17 years and pelvic inflammatory disease at age 19 years. At age 21 years, cervical cytology showed a low-grade squamous intraepithelial lesion; repeat cervical cytology 1 year later showed no abnormalities. Current medications are a prenatal vitamin and occasional ibuprofen for mid-cycle abdominal pain. She is 168 cm (5 ft 6 in) tall and weighs 59 kg (130 lb); BMI is 21 kg/m^2. Vital signs are within normal limits. Physical examination discloses no abnormalities. Hysterosalpingography is done, and x-rays obtained during the procedure are shown. Without treatment, which of the following is the most likely clinical course for this patient?

(A) Female factor infertility
(B) Hydatidiform mole
(C) Recurrent spontaneous abortions
(D) Successful pregnancy within the next year

---

85. A 50-year-old woman comes to the office for a health maintenance examination. She feels well. Medical history is unremarkable and she takes no medications. There is no family history of serious illness. Physical examination, including breast examination, discloses no abnormalities. Mammography shows a cluster of 20 pleomorphic microcalcifications in a 1-cm area at the 2-o'clock position of the right breast. Which of the following is the most likely diagnosis?

(A) Ductal carcinoma in situ
(B) Fat necrosis
(C) Fibroadenoma
(D) Mastitis
(E) Sclerosing adenosis

86. A community has created standard-of-care guidelines for ambulatory patients with several common diagnoses. The community's standard of care for patients with asthma is annual pulmonary function testing. Recent analysis of billing records from one clinic in this community shows that only 34% of patients with asthma at the clinic undergo pulmonary function testing. Enacting which of the following procedures is most likely to improve this clinic's adherence to the community's standard of care for patients with asthma?

(A) Annual chart reviews of patients with asthma and feedback to the physicians
(B) Diagnosis-driven reminders in patient charts
(C) Placement of flyers in the clinic waiting room that remind patients with asthma to undergo annual pulmonary function testing
(D) Pulmonary function testing of all patients examined for respiratory symptoms
(E) Pulmonary function testing of patients with asthma at each examination preceded by an exacerbation

87. A 27-year-old woman, gravida 1, para 1, comes to the emergency department because of a 1-day history of moderate pain in her right breast. Three weeks ago, she underwent cesarean delivery of a healthy newborn at term because of breech presentation. She is breast-feeding, but the newborn is not latching properly on the right. She has no history of serious illness. Medications are acetaminophen for postoperative pain and a prenatal vitamin. Temperature is 38.9°C (102.0°F). Examination of the right breast shows a 5 × 2-cm wedge-shaped area of erythema and moderate tenderness. The left breast is normal. Abdominal examination shows a well-healing surgical incision. In addition to beginning antipyretic therapy, which of the following is the most appropriate next step in management?

(A) Application of a breast binder on the right
(B) Dicloxacillin therapy
(C) Fluconazole therapy
(D) Fine-needle aspiration of the erythematous area
(E) No further management is indicated

88. A 12-year-old girl is brought to the office for a well-child examination. She feels well. She has no history of serious illness and receives no medications. Menarche has not yet occurred. She is at the 30th percentile for height and 60th percentile for weight. Examination of the breasts shows no glandular tissue; the areolae follow the skin contours of the chest. There is no pubic hair. The remainder of the examination shows no abnormalities. Which of the following is the most appropriate next step in diagnosis?

(A) Measurement of serum follicle-stimulating hormone and luteinizing hormone concentrations
(B) Measurement of serum growth hormone and thyroxine concentrations
(C) MRI of the brain
(D) X-ray of the left hand and wrist to determine bone age
(E) No additional diagnostic steps are indicated

89. A 42-year-old woman comes to the office because of a 2-month history of episodes of light-headedness and loss of consciousness for 10 seconds. The episodes occur two to three times weekly at work. She says her light-headedness resolves after she eats snacks. During this time, she also has had a 9-kg (20-lb) weight gain. She has no history of serious illness and takes no medications. She is 160 cm (5 ft 3 in) tall and weighs 72 kg (160 lb); BMI is 28 kg/m^2. Vital signs are within normal limits. Physical examination, including neurologic examination, shows no abnormalities. Serum glucose concentration is 41 mg/dL, serum C peptide concentration is 0.5 ng/mL (N=0.5–2.5), and serum insulin concentration is 80 μIU/mL (N=5–20). CT scan of the abdomen is most likely to show which of the following findings?

(A) Duodenal mass with multiple liver metastases
(B) Fluid-filled mass between the posterior aspect of the stomach and pancreas
(C) Low-density mass in the head of the pancreas obstructing the main duct
(D) Vascular mass in the neck of the pancreas
(E) No abnormalities

90. A hospital with a large intensive care unit (ICU) would like to improve communication among team members who care for patients with complex conditions. Research has shown that team communication is most effective when one clinician is designated as the team leader and assumes responsibility for directing the care. The team leader ensures that all team members share a common understanding of the patient and openly discuss their views about the case. The common understanding includes the patient's diagnosis, prognosis, and care plan. This allows the team to quickly recognize when new data deviate from the expected and then reassess the team's approach to the patient. Which of the following is the most appropriate method for maintaining a common mental model among all team members?

(A) Conduct weekly structured team briefings and daily huddles
(B) Educate all team members on protocols for common illnesses
(C) Have the team leader discuss the case with each team member individually on a regular basis
(D) Maintain a consistent team of intensivists in the ICU
(E) Maintain a disease-specific ICU

91. A 12-month-old girl is brought to the office by her father because of a 3-day history of fever and mildly decreased appetite and activity. She has not had cough, vomiting, or diarrhea. She receives no medications. Vaccinations are up-to-date. She is at the 35th percentile for height, 50th percentile for weight, and 60th percentile for head circumference. Temperature is 39.2°C (102.6°F); other vital signs are within normal limits. Examination shows no other abnormalities. A catheter urine sample is obtained, and urinalysis shows 20–30 WBC/hpf and is positive for nitrites. Oral trimethoprim-sulfamethoxazole therapy is begun. The next day, urine culture grows greater than 100,000 colonies/mL of *Escherichia coli* that is susceptible to trimethoprim-sulfamethoxazole. Which of the following is the most appropriate next step in management?

(A) Ciprofloxacin prophylaxis
(B) Clindamycin prophylaxis
(C) CT urography
(D) Ultrasonography of the kidneys and bladder
(E) Voiding cystourethrography

92. A 32-year-old man comes to the office because of a 2-week history of persistent nonproductive cough occurring in short bursts. During the past week, he has had bilateral chest pain with severe coughing. He has vomited several times after coughing vigorously. He has not had shortness of breath or fever. Three weeks ago, he had a runny nose, sneezing, and mild malaise that resolved spontaneously after 1 week. Toward the end of that period, he noticed the onset of cough, which has since worsened. He has no history of serious illness, and his only medication is over-the-counter cough drops. He drinks 24 to 36 oz of beer weekly. He does not smoke cigarettes or use other substances. He is sexually active with one male partner; they use condoms consistently. He is 173 cm (5 ft 8 in) tall and weighs 86 kg (190 lb); BMI is 29 kg/m^2. Temperature is 37.0°C (98.6°F), pulse is 70/min, respirations are 12/min, and blood pressure is 120/80 mm Hg. Pulse oximetry on room air shows an oxygen saturation of 98%. During the examination, the patient coughs several times. There is no sinus tenderness to palpation and no cervical lymphadenopathy. The oropharynx appears normal. Lungs are clear to auscultation. Cardiac examination discloses no abnormalities. Which of the following is the most appropriate pharmacotherapy?

(A) Albuterol
(B) Azithromycin
(C) Levofloxacin
(D) Omeprazole
(E) Prednisone

93. **Patient Information**

Age: 18 years
Gender: F, self-identified
Race/Ethnicity: unspecified
Site of Care: emergency department

History

Reason for Visit/Chief Concern: "I'm coughing up green and yellow mucus."
History of Present Illness:
- cystic fibrosis with two to three exacerbations yearly for past 5 years
- 3-day history of cough productive of copious amounts of yellow and green sputum
- associated with subjective fever, shaking chills, and progressive shortness of breath
- symptoms occurred first only with exertion but now occur at rest
- also reports sharp left-sided chest pain that she attributes to extended paroxysms of coughing
- symptoms are similar to previous exacerbations

Past Medical History:
- cystic fibrosis complicated by bronchiectasis and pancreatic insufficiency

Medications:
- pancrelipase
- dornase alfa
- ivacaftor
- albuterol prn for pulmonary symptoms
- 3.0% inhaled saline prn for pulmonary symptoms

Allergies:
- no known drug allergies

Family History:
- brother: age 13 years with cystic fibrosis

Psychosocial History:
- does not smoke cigarettes, drink alcoholic beverages, or use other substances
- high school student

Physical Examination

Temp	Pulse	Resp	BP	O_2 Sat	Ht	Wt	BMI
37.9°C (100.2°F)	112/min and regular	22/min	138/74 mm Hg	92% on RA	157 cm (5 ft 2 in)	54 kg (118 lb)	22 kg/m^2

- Appearance: in mild respiratory distress; using accessory muscles of respiration; frequent coughing productive of yellow and green sputum
- Pulmonary: bilateral diffuse crackles with moderate diffuse expiratory wheezes more prominent in the apices

Diagnostic Studies

Blood	
Hematocrit	42%
Hemoglobin	13.1 g/dL
WBC	16,400/mm^3
Platelet count	334,000/mm^3

- portable chest x-ray: bilateral basilar consolidations with increased interstitial markings; no effusions or pneumothorax

Question: In addition to initiating broad-spectrum antibiotic therapy, which of the following is the most appropriate next step in evaluation?

(A) CT angiography of the chest
(B) Serum immunoglobulin concentrations
(C) Spirometry with lung volume measurement
(D) Sputum culture
(E) Transbronchial lung biopsy

94. A 16-year-old boy comes to the emergency department (ED) because of moderate left knee pain and abrasions sustained 2 hours ago in a skateboarding collision. He has a history of occasional injuries sustained while skateboarding, including an injury to the same knee 6 months ago. He says his knee had felt fine until today's injury, and he has been fit and active. The patient has an *EXT1* mutation, which is also present in his father. The patient takes no medications. Vital signs are within normal limits. Examination shows superficial abrasions over the anterolateral aspect of the left knee. No effusion or instability is noted in the knee, but there is tenderness to palpation. The patient walks with a limp but is able to bear weight on the leg. X-ray of the left knee shows two 1 × 2-cm bone polyps extending, one medially and one laterally, from the proximal tibia at the level of the closing physis; this is unchanged from an x-ray of the same knee obtained during his previous ED visit 6 months ago. Which of the following is the most appropriate recommendation for activity and additional workup?

(A) Biopsy of the polyps and recommendation to bear weight as tolerated
(B) Biopsy of the polyps and recommendation for no weight bearing
(C) MRI of the knee and recommendation to bear weight as tolerated
(D) MRI of the knee and recommendation for no weight bearing
(E) Recommendation to bear weight as tolerated; no further workup needed
(F) Recommendation for no weight bearing; no further workup needed

95. A 70-year-old woman comes to the office because of three episodes of rectal bleeding during the past 4 days. During each episode, she had the sudden urge to have a bowel movement and then passed a bright red, bloody stool. She has not had chest, abdominal, or rectal pain, palpitations, dizziness, light-headedness, or black stools. Her most recent colonoscopy 8 years ago showed no abnormalities. She has osteoarthritis treated with daily ibuprofen. She takes no other medications. She is 168 cm (5 ft 6 in) tall and weighs 69 kg (152 lb); BMI is 25 kg/m^2. Temperature is 37.0°C (98.6°F), and respirations are 16/min. While sitting, her pulse is 80/min and blood pressure is 130/76 mm Hg. While standing, her pulse is 88/min and blood pressure is 126/74 mm Hg. On examination, mucous membranes are moist. The abdomen is soft, nontender, and nondistended. There is no rebound tenderness or guarding. Bowel sounds are normal. Rectal examination shows no stool in the vault; there are no masses. Which of the following is the most likely diagnosis?

(A) Anal fissure
(B) Colonic polyp
(C) Diverticulosis
(D) Duodenal ulcer
(E) Gastritis
(F) Ulcerative colitis

96. A 14-year-old boy is brought to the office because of a 6-month history of changes in behavior. Two days ago, the patient and his friends were detained by police for trespassing on abandoned property and entering an unoccupied house. No alcohol or other substances were involved. The police officer who brought the patient home reported that there was evidence indicating this had not been the first time the boys entered the property. The mother says her son was polite and close with his parents until 6 months ago when he began socializing with a different group of boys in his class. She says the patient is constantly exchanging text messages with his new friends. The group has inside jokes and nicknames for one another. Every weekend, the patient goes out with his friends and argues with his parents about his curfew. At home, he is irritable and shares few details about his outside activities with his parents. He has no history of serious illness and receives no medications. Examination shows no abnormalities. Urine toxicology screening is negative. Which of the following is the most likely explanation for this patient's behavior?

(A) Adjustment disorder
(B) Attention-deficit/hyperactivity disorder
(C) Conduct disorder
(D) Oppositional defiant disorder
(E) Normal development

97. A 6-year-old boy is brought to the emergency department by his mother because of the acute onset 1 hour ago of severe right-sided lumbar pain and three episodes of emesis. The pain is sharp and causes him to double over and cry. Medical and family histories are unremarkable. The patient takes no medications. Temperature is 36.7°C (98.0°F), pulse is 150/min, respirations are 30/min, and blood pressure is 110/70 mm Hg. He appears to be in severe pain. Abdominal examination discloses right upper quadrant tenderness to palpation. The remainder of the examination discloses no abnormalities. Intravenous morphine is administered and results in improvement in the patient's pain. Results of dipstick urinalysis are shown:

Specific gravity	1.035 (N=1.003–1.029)
pH	5.5
Protein	30 mg/dL
Glucose	Negative
Bilirubin	Negative
Blood	4+
Leukocyte esterase	1+
Nitrite	Negative
Urobilinogen	Negative
WBCs	5–10/hpf
RBCs	50–100/hpf

Renal ultrasonography shows severe right-sided hydronephrosis with moderate hydroureter to the bladder. Which of the following is the most appropriate next step in evaluation?

(A) Captopril renography
(B) CT scan of the abdomen and pelvis
(C) MAG-3 renal scan with furosemide
(D) Radionuclide cystography
(E) Retrograde pyelography
(F) Ultrasonography of the spinal column
(G) Voiding cystourethrography

98. A 20-year-old man comes to the office at the end of the summer because of a 2-month history of generalized malaise, fatigue, intermittent cough productive of whitish sputum, and decreased appetite. During this time, he has lost approximately 4 kg (10 lb). The patient previously was on the college track team but now feels winded when he tries to go running. He has a history of childhood asthma. He currently takes no medications. He does not smoke cigarettes, drink alcoholic beverages, or use other substances. He identifies as African American. He has been working at a lumber mill for the summer, feeding logs into a saw. He is 178 cm (5 ft 10 in) tall and weighs 77 kg (170 lb); BMI is 24 kg/m^2. Temperature is 37.7°C (99.9°F), pulse is 78/min, respirations are 20/min, and blood pressure is 114/70 mm Hg. Diffuse crackles are heard bilaterally. The remainder of the examination shows no abnormalities. Chest x-ray shows a reticulonodular pattern that is more prominent in the upper lung fields. Results of pulmonary function tests show a mixed obstructive and restrictive pattern. Which of the following is the most likely diagnosis?

(A) Aspergillosis
(B) Asthma recurrence
(C) Hypersensitivity pneumonitis
(D) Sarcoidosis
(E) Silicosis

99. A 32-year-old woman is admitted to the hospital because of a 2-year history of intractable seizures. Her boyfriend, who has accompanied her, describes the seizures as episodes of bilateral limb shaking and moving her head from side to side, during which the patient closes her eyes and cries. The episodes last 15 to 20 minutes, and she is intermittently responsive during this time. Trials of phenytoin, carbamazepine, gabapentin, oxcarbazepine, and divalproex have not provided relief of her symptoms. Her current medications are zonisamide and pregabalin. The patient has a history of childhood sexual and physical abuse. Vital signs are within normal limits. Neurologic examination shows no focal findings. The patient undergoes long-term video EEG monitoring, which shows that her seizures are not associated with any EEG changes. Which of the following is the most appropriate next step in management?

(A) Cognitive behavioral therapy
(B) Hypnotic therapy
(C) Increasing the dosage of pregabalin
(D) Psychoanalytic therapy
(E) Surgical resection of the epileptogenic focus

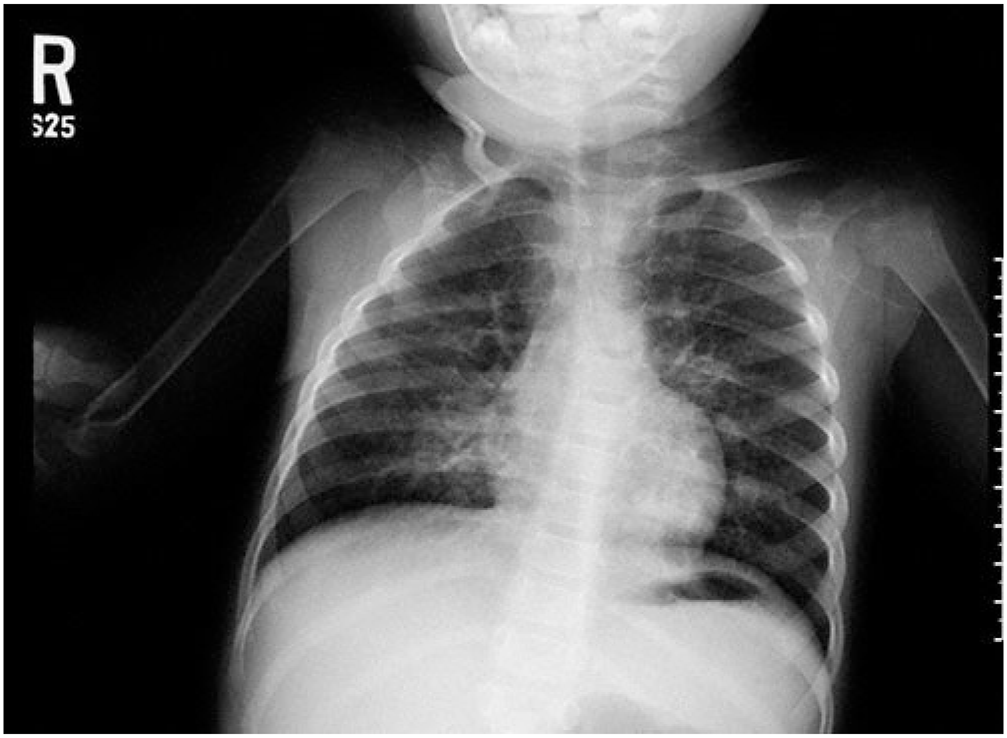

100. A 2-year-old girl is brought to the clinic by her parents for an initial examination. Medical records show that she has a cleft palate and ventriculoseptal defects that have not been repaired. During the past 18 months, she has had two episodes of pneumonia and at least 10 episodes of otitis media treated with antibiotic therapy. The patient's parents recently adopted her from China. She is at the 10th percentile for length and 5th percentile for weight. Vital signs are within normal limits. Examination shows middle ear effusions bilaterally and a cleft palate. Lungs are clear to auscultation. A grade 3/6 holosystolic murmur is heard on cardiac examination. There is no hepatosplenomegaly. Chest x-ray is shown. A complete blood count is most likely to show which of the following leukocyte findings in this patient?

(A) Eosinophilia
(B) Lymphocytosis
(C) Lymphopenia
(D) Neutropenia
(E) Neutrophilia

101. A 25-year-old primigravid woman at 28 weeks' gestation comes to the office for a routine prenatal visit. At 18 weeks' gestation, she had vaginal bleeding that resolved spontaneously. At that time, her blood group was determined to be O, Rh-negative and she received Rh_o(D) immune globulin. She reports good fetal movement. Blood pressure is 120/70 mm Hg. Fetal heart rate is 160/min. Fundal height is 30 cm. The fetus is in a vertex presentation. Hemoglobin concentration is 11 g/dL and hematocrit is 34%. Serum antibody assay is positive; the anti-D titer is too weak to measure. Results of serologic testing for syphilis and HIV antibody testing are negative. Which of the following is the most appropriate next step in management?

(A) Repeat serum anti-D antibody titer
(B) Ultrasonography of the pelvis
(C) Administration of Rh_o(D) immune globulin
(D) Amniocentesis
(E) Induction of labor

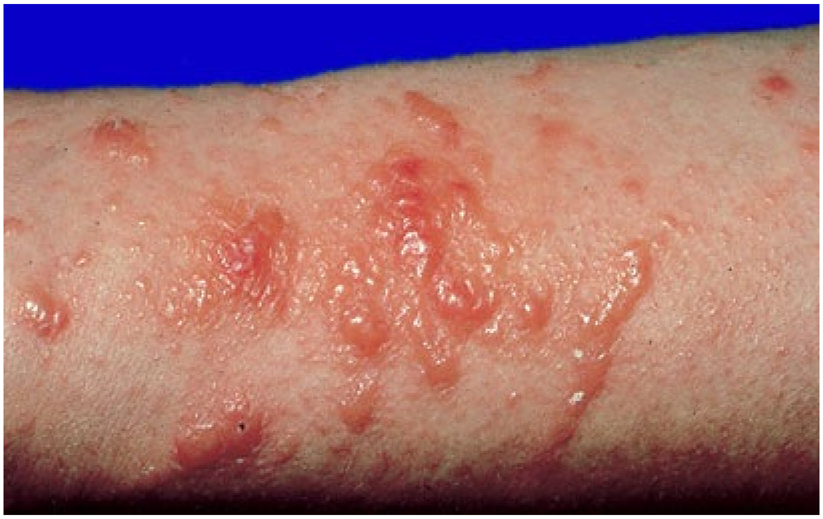

102. A 5-year-old boy is brought to the office by his parents in July because of a 2-day history of an itchy rash. The rash began as small red bumps on the right forearm and spread to the left cheek and right ankle within the same day. He has not had fever, vomiting, or diarrhea. No other family members have similar symptoms. Medical history is unremarkable and he receives no medications. The family switched to a new brand of bath soap last week. They have three pet cats and one dog. Three days ago, the family attended a picnic at a nearby park. Temperature is 37.0°C (98.6°F), pulse is 90/min, and respirations are 20/min. The patient is alert and scratching his right forearm. Examination shows mild edema of the left cheek. A photograph of the right forearm is shown. Similar lesions are noted over the left cheek and right ankle. The remainder of the examination shows no abnormalities. Avoidance of which of the following is most likely to have prevented this patient's condition?

(A) Dog
(B) Flora
(C) Peanut butter
(D) Perfumed bath soap
(E) Sun

103. **Patient Information**

Age: 56 years
Gender: F, self-identified
Race/Ethnicity: mixed race (White and Honduran)
Site of Care: clinic

History

Reason for Visit/Chief Concern: "I'm tired and I don't feel well."
History of Present Illness:
- fatigue and malaise for 3 months; tires quickly with physical activity
- early satiety

Past Medical History:
- 5-year history of hypertension
- 5-year history of hyperlipidemia

Medications:
- chlorthalidone
- enalapril
- atorvastatin

Allergies:
- no known drug allergies

Physical Examination

Temp	Pulse	Resp	BP	O_2 Sat	Ht	Wt	BMI
37.5°C (99.5°F)	75/min	14/min	139/78 mm Hg	–	162 cm (5 ft 4 in)	94 kg (207 lb)	36 kg/m^2

- HEENT: normal conjunctivae and oropharynx; thyroid gland is normal in size with no mass or tenderness
- Pulmonary: clear to auscultation
- Cardiac: S_1 and S_2; no murmur or gallop
- Abdominal: nontender; liver edge palpated at the right costal margin with inspiration; dullness to percussion over the fifth to eighth intercostal spaces at the left anterior axillary line

Diagnostic Studies

Blood		Urine	
RBC	4.5 million/mm^3	Protein	3+
Hematocrit	39%		
Hemoglobin	13.0 g/dL		
MCV	86.7 μm^3		
WBC	67,000/mm^3		
Neutrophils, segmented	83%		
Lymphocytes	2%		
Eosinophils	5%		
Basophils	4%		
Metamyelocytes	3%		
Myelocytes	3%		

Question: Which of the following is the most likely diagnosis?
(A) Acute lymphocytic leukemia
(B) Acute myeloid leukemia
(C) Chronic lymphocytic leukemia
(D) Chronic myeloid leukemia
(E) Monoclonal gammopathy of undetermined significance

104. A 65-year-old woman comes to the office because of a 6-month history of progressive shortness of breath and a 1-year history of nonproductive cough. Initially, the shortness of breath occurred only with exertion but now occurs at rest. It does not worsen when she lies down but is still present. Her symptoms have limited her ability to travel internationally with the Peace Corps. She has not had chest pain, fever, chills, or blood-tinged sputum. She has hypertension, type 2 diabetes mellitus, and major depressive disorder. Medications are amlodipine, lisinopril, insulin glargine, and sertraline. She has smoked one pack of cigarettes daily for 50 years. She drinks one glass of wine on special occasions. Temperature is 37.2°C (99.0°F), pulse is 77/min, respirations are 16/min, and blood pressure is 132/82 mm Hg. Pulse oximetry on room air shows an oxygen saturation of 94%. There is no jugular venous distention. On pulmonary examination, end-expiratory wheezes are heard; there is a prolonged expiratory phase. Heart sounds are distant. Which of the following is the most appropriate next step in diagnosis?

(A) CT scan of the chest
(B) Echocardiography
(C) Exercise stress testing
(D) PPD skin testing and chest x-ray
(E) Pulmonary function testing

105. A 45-year-old man comes to the office because of a 6-month history of difficulty sleeping, nervousness, and fatigue. He goes to bed at 11 PM every night, falls asleep within 15 minutes, then sleeps "fitfully." After he awakens, he has mild neck pain and feels tired, edgy, and tense during the rest of the day. The company where he worked for 27 years closed 6 months ago, and he has been unable to find another job. He says he worries about his finances and job prospects. Medical history is remarkable for alcohol use disorder. He has not drunk alcohol for 1 year. He says he is committed to being sober but recently has "wanted a drink." He currently takes no medications. Vital signs are within normal limits. Physical examination discloses no abnormalities. Mental status examination shows a constricted, anxious affect. Which of the following is the most appropriate pharmacotherapy?

(A) Buspirone
(B) Clonazepam
(C) Diphenhydramine
(D) Imipramine
(E) Quetiapine
(F) Temazepam

106. A 56-year-old man comes to the clinic with his wife because of a 6-month history of restlessness while sleeping. The wife says he motions as if he were throwing or kicking a ball and sometimes punches the pillow during the night; he has hit her twice. Recently, he began talking in his sleep and yelling expletives. Occasionally, he seems to be "acting out" a dream; twice, he has awakened and could tell his wife what the dream was about. Otherwise, he is unaware of these episodes and feels well rested in the mornings. He has a history of constipation treated with polyethylene glycol. He takes no other medications. Vital signs are within normal limits. Examination shows no abnormalities. This patient is at greatest risk for developing which of the following?

(A) Alzheimer disease
(B) Amyotrophic lateral sclerosis
(C) Cerebral infarction
(D) Narcolepsy
(E) Parkinson disease

107. A 77-year-old woman is brought to the emergency department by her son because of a 3-day history of pain in her right wrist. The patient has advanced dementia, Alzheimer type, and is unable to provide her medical history. She is completely dependent on her son, with whom she lives and who is her only caregiver. The son says he is unaware of any trauma to the patient's wrist. The son says the patient has no other history of serious illness. Her only medication is memantine. She is nonverbal and has a flat affect. She appears unkempt and to have poor hygiene. Temperature is 36.7°C (98.1°F), pulse is 82/min and regular, respirations are 18/min, and blood pressure is 150/90 mm Hg. The patient moans when the physician touches her right wrist. Examination shows multiple bruises over both forearms and wrists. The remainder of the examination shows no abnormalities. X-ray of the right wrist shows a distal radial fracture; the fracture is treated with cast immobilization. Which of the following is the most appropriate next step in management?

(A) Admit the patient to the hospital for further evaluation
(B) Arrange for placement of the patient in a nursing care facility
(C) Contact adult protective services
(D) Provide phone numbers of nearby nursing care facilities
(E) Recommend further evaluation at a clinic specializing in geriatric patients

108. A 27-year-old man comes to the emergency department because of a 4-week history of progressive shortness of breath and swelling of the lower extremities. During this time, he also has had shortness of breath during the night while lying down. He also has a 6-month history of intermittent, moderate substernal chest pain and pressure while exercising. He has not had syncope. He has no history of serious illness and takes no medications. Temperature is 37.0°C (98.6°F), pulse is 110/min, respirations are 24/min, and blood pressure is 145/92 mm Hg. Examination shows severe, diffuse gingivitis. Crackles are heard halfway up the posterior lung bases bilaterally. On cardiac examination, a prominent S_3 is heard. There is 3+ pitting edema of the lower extremities. ECG shows sinus tachycardia. Which of the following is the most likely substance used?

(A) Cocaine
(B) Heroin
(C) Methadone
(D) Methamphetamine
(E) Toluene

109. A 39-year-old man comes to the office because of a 3-month history of episodes of headaches, palpitations, and sweating. The episodes occur two to three times monthly. He has hypertension treated with a calcium-channel blocking agent and an ACE inhibitor. Blood pressure is 166/112 mm Hg. Examination shows no other abnormalities. CT scan of the abdomen shows a 4-cm left adrenal mass. Which of the following is the most appropriate next step in management?

(A) 24-Hour urine collection for measurement of vanillylmandelic acid and 5-hydroxyindoleacetic acid
(B) Adrenal venous sampling
(C) Laparoscopic left adrenalectomy and right adrenal biopsy
(D) Measurement of plasma and urine catecholamine and metabolite concentrations
(E) Transsphenoidal hypophysectomy

110. A 41-year-old woman is being prepared for discharge from the hospital after treatment for acute pancreatitis. She has tolerated oral fluids well for the past 12 hours. She has had three prior admissions for pancreatitis. Medical history also is remarkable for alcohol use disorder. The patient does not have secure housing. Which of the following recommendations is most likely to reduce this patient's risk for future hospital admissions?

(A) Abstinence from alcoholic beverages
(B) Enrollment in a health insurance plan
(C) Nutritional counseling
(D) Placement in a women's shelter

111. An 8-year-old boy is brought to the urgent care center by his mother while on a family vacation in Florida because of a 3-day history of an itchy, burning rash. The ambient temperature during the past 3 days has been 37.8°C (100.0°F). The mother does not recall any recent exposures for her son, and none of her other children have a rash. The patient's medical history is unremarkable. Examination of the patient's back shows superficial, clear, 1- to 2-mm vesicles that resemble water droplets. The vesicles rupture easily on palpation. There is no surrounding erythema. It is most appropriate for the physician to recommend which of the following for this patient?

(A) Antifungal cream
(B) Heat avoidance
(C) Oral antibiotic therapy
(D) Oral antiviral therapy
(E) Oral retinoid therapy
(F) Ultraviolet therapy

112. A 55-year-old woman comes to the office for a follow-up examination 3 months after sustaining an uncomplicated myocardial infarction in the distribution of the left anterior descending artery. She has participated in an outpatient rehabilitation program since discharge from the hospital 6 weeks ago, but her attendance in the program has become irregular during the past 4 weeks. When questioned about this, she reports a 4-week history of depressed mood, inability to enjoy activities she previously found pleasurable, loss of appetite, and insomnia. She also has hyperlipidemia and hypertension. Medications are aspirin, atorvastatin, and metoprolol. She is 163 cm (5 ft 4 in) tall and weighs 68 kg (150 lb); BMI is 26 kg/m^2. Vital signs are within normal limits. Lungs are clear to auscultation. Cardiac examination discloses a normal rhythm. There is no peripheral edema. Results of serum studies are within the reference ranges. Which of the following is the most likely effect of this patient's depression on her overall health?

(A) It is likely to decrease her perceived quality of life but have no impact on her mortality
(B) It is likely to double her risk for death during the next 3 months
(C) It is likely to decrease her risk for ischemia because she is likely to rest and avoid socializing
(D) It likely will have no significant effect on her physical health

113. A 78-year-old man is evaluated in the hospital following admission 8 hours ago for treatment of aspiration pneumonia. He has a 5-year history of intermittent dysphagia resulting in spontaneous regurgitation of undigested food and liquids. Over-the-counter antacids had provided no improvement in his dysphagia. Until the development of his recent fever, chest pain, and shortness of breath, the patient had not had any other symptoms and had not sought medical care. He has no other history of serious illness. In the hospital, he has been receiving intravenous piperacillin-tazobactam. He does not smoke cigarettes or drink alcoholic beverages. Temperature is 38.1°C (100.6°F), pulse is 90/min, respirations are 24/min, and blood pressure is 100/65 mm Hg. He appears comfortable and is sitting upright in bed. Decreased breath sounds are heard in the right lower lung field. Abdomen is soft and nontender. Which of the following is the most likely underlying cause of this patient's dysphagia?

(A) Achalasia
(B) Esophageal cancer
(C) Hiatal hernia
(D) Zenker diverticulum

114. A 27-year-old woman comes to the office because of a 3-day history of constant mild pain in her left lower abdomen. Vigorous movement makes the pain worse. She has not had nausea. She is otherwise healthy and takes no medications. Menses occur at regular 28-day intervals and last 4 to 6 days. Her last menstrual period was 3 weeks ago. She is sexually active with one male partner, and they use condoms inconsistently. Temperature is 36.7°C (98.0°F), pulse is 80/min, respirations are 18/min, and blood pressure is 104/60 mm Hg. The abdomen is flat and soft with voluntary guarding of the left lower quadrant. Bowel sounds are normal. Pelvic examination shows tender fullness of the left adnexa. Urine pregnancy test is negative. Ultrasonography shows a normal uterus with a 3 × 4-cm left adnexal cyst and a small amount of free fluid in the pelvis. Which of the following is the most appropriate next step in management?

(A) Antibiotic therapy
(B) Appendectomy
(C) CT scan-guided aspiration
(D) Laparoscopy
(E) Observation only

115. A 60-year-old woman comes to the office for a health maintenance examination. She says she feels well. She has no history of serious illness and takes no medications. Vital signs are within normal limits. Observation of the tympanic membranes is limited because of cerumen filling both external auditory canals. The external ears appear normal. No other abnormalities are noted. On questioning, she says she hears well. Which of the following is the most appropriate next step in management?

(A) Irrigation of the ear canals by the physician
(B) Manual removal of the cerumen by an otorhinolaryngologist
(C) Recommendation for daily use of cotton swabs
(D) Recommendation for use of a ceruminolytic agent
(E) No further management is indicated at this time

116. A hospitalized 37-year-old woman has a 2-hour history of severe chest pain and blood-streaked sputum 4 days after undergoing hysterectomy and oophorectomy for ovarian cancer. Current medications are oxycodone, prophylactic low-molecular-weight heparin, and a multivitamin. Temperature is 37.0°C (98.6°F), pulse is 90/min, respirations are 22/min, and blood pressure is 110/70 mm Hg. Examination shows a left pleural rub. An x-ray and CT scan of the chest show a triangular pleural-based density in the lower lobe of the left lung. No pulmonary emboli are seen in the major branches. Venous duplex ultrasonography of the lower extremities shows no deep venous thrombosis. Which of the following is the most likely diagnosis?

(A) Air embolism
(B) Empyema
(C) Hemothorax
(D) Pericarditis
(E) Pneumonia
(F) Pulmonary hemorrhage
(G) Pulmonary hypertension
(H) Pulmonary infarction

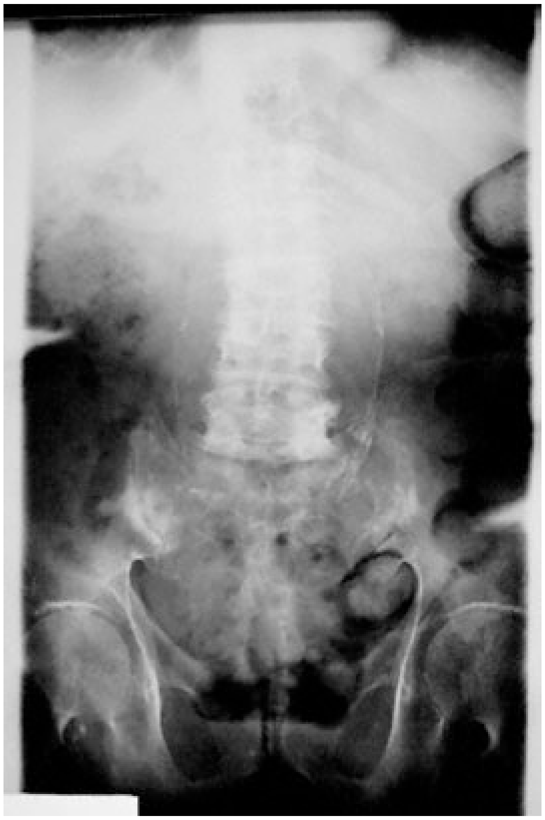

117. A 77-year-old man is brought to the emergency department because of a 1-hour history of severe, constant abdominal pain and dizziness. He has hypertension well controlled with hydrochlorothiazide. He has smoked two packs of cigarettes daily for 60 years. He is 175 cm (5 ft 9 in) tall and weighs 113 kg (250 lb); BMI is 37 kg/m^2. Temperature is 37.0°C (98.6°F), pulse is 120/min, respirations are 25/min, and blood pressure is 80/60 mm Hg. Pulse oximetry on room air shows an oxygen saturation of 93%. He appears uncomfortable. Abdominal examination shows mild distention; bowel sounds are decreased. Groin pulses are 1+. Dorsalis pedis pulses are not palpable. Hematocrit is 37% and leukocyte count is 10,000/mm^3. Serum electrolyte concentrations, liver function tests, and amylase activity are within the reference ranges. An x-ray of the abdomen is shown. Which of the following is the most likely diagnosis?

(A) Acute pancreatitis
(B) Mesenteric ischemia
(C) Perforated duodenal ulcer
(D) Ruptured abdominal aortic aneurysm
(E) Small-bowel obstruction

---

118. A 1-week-old male newborn is evaluated in the hospital nursery 4 days after undergoing operative repair of a myelomeningocele. He was born at 37 weeks' gestation via uncomplicated, spontaneous vaginal delivery to an 18-year-old primigravid woman. The defect was diagnosed prenatally by ultrasonography, and the repair was done 72 hours after delivery. He is at the 5th percentile for length, 10th percentile for weight, and 25th percentile for head circumference. Temperature is 36.9°C (98.4°F), pulse is 138/min, and respirations are 42/min. Pulse oximetry on room air shows an oxygen saturation of 98%. The newborn appears comfortable. Physical examination shows a soft, flat, anterior fontanel. Red reflexes are normal. Moro reflex is symmetric in the upper extremities; there is no movement in the lower extremities. Ultrasonography of the brain shows mildly enlarged ventricles. Which of the following is the most likely associated finding in this patient?

(A) Congenital heart malformation
(B) Cryptorchidism
(C) Impaired folate metabolism
(D) Neurogenic bladder
(E) Severe cognitive impairment

119. A randomized controlled trial is conducted to assess the effectiveness of a new antiarrhythmic drug in patients with recurrent ventricular tachycardia. A total of 100 participants with ventricular tachycardia are randomly assigned to receive either the new drug or a placebo. Results show that the new drug group has fewer episodes of recurrent ventricular tachycardia compared with the placebo group. The difference was found to be statistically significant ($p<0.05$). The investigators conclude that the new drug can be used to decrease mortality from cardiac arrhythmia. Which of the following factors most likely invalidates the author's conclusions?

(A) Extrapolation of findings beyond data
(B) Insufficient power
(C) No information regarding confidence interval
(D) Selection bias

120. A 42-year-old woman comes to the office because of a 2-week history of sore throat and fullness in her neck. She has no history of serious illness and takes no medications. She appears anxious. Temperature is 37.8°C (100.0°F), pulse is 102/min, respirations are 22/min, and blood pressure is 146/82 mm Hg. The oropharynx is clear, and there are no exudates. There is palpable fullness and tenderness of the anterior aspect of the neck. There is no lymphadenopathy. Lungs are clear to auscultation. Results of a complete blood count are within the reference ranges. Chest x-ray shows no abnormalities. Which of the following is the most likely diagnosis?

(A) Acute mononucleosis
(B) Gastroesophageal reflux disease
(C) Laryngitis
(D) Subacute thyroiditis
(E) Tracheitis

ANSWER FORM FOR USMLE STEP 2 CK SAMPLE TEST QUESTIONS

Block 1 (Questions 1–40)

1.____	9.____	17.____	25.____	33.____
2.____	10.____	18.____	26.____	34.____
3.____	11.____	19.____	27.____	35.____
4.____	12.____	20.____	28.____	36.____
5.____	13.____	21.____	29.____	37.____
6.____	14.____	22.____	30.____	38.____
7.____	15.____	23.____	31.____	39.____
8.____	16.____	24.____	32.____	40.____

Block 2 (Questions 41–80)

41.____	49.____	57.____	65.____	73.____
42.____	50.____	58.____	66.____	74.____
43.____	51.____	59.____	67.____	75.____
44.____	52.____	60.____	68.____	76.____
45.____	53.____	61.____	69.____	77.____
46.____	54.____	62.____	70.____	78.____
47.____	55.____	63.____	71.____	79.____
48.____	56.____	64.____	72.____	80.____

Block 3 (Questions 81–120)

81.____	89.____	97.____	105.____	113.____
82.____	90.____	98.____	106.____	114.____
83.____	91.____	99.____	107.____	115.____
84.____	92.____	100.____	108.____	116.____
85.____	93.____	101.____	109.____	117.____
86.____	94.____	102.____	110.____	118.____
87.____	95.____	103.____	111.____	119.____
88.____	96.____	104.____	112.____	120.____

Answer Key for USMLE Step 2 CK Sample Test Questions

Block 1 (Questions 1–40)

1.	A	9.	B	17.	E	25.	D	33.	B
2.	E	10.	F	18.	E	26.	A	34.	A
3.	A	11.	B	19.	C	27.	C	35.	C
4.	A	12.	B	20.	B	28.	B	36.	E
5.	E	13.	B	21.	C	29.	A	37.	D
6.	C	14.	D	22.	E	30.	B	38.	D
7.	B	15.	C	23.	C	31.	B	39.	C
8.	B	16.	B	24.	B	32.	A	40.	A

Block 2 (Questions 41–80)

41.	D	49.	C	57.	D	65.	E	73.	A
42.	A	50.	C	58.	F	66.	A	74.	E
43.	C	51.	C	59.	D	67.	C	75.	E
44.	E	52.	A	60.	D	68.	D	76.	D
45.	B	53.	F	61.	D	69.	B	77.	C
46.	C	54.	E	62.	E	70.	C	78.	B
47.	A	55.	B	63.	B	71.	C	79.	B
48.	A	56.	E	64.	C	72.	C	80.	D

Block 3 (Questions 81–120)

81.	D	89.	E	97.	B	105.	A	113.	D
82.	D	90.	A	98.	C	106.	E	114.	E
83.	E	91.	D	99.	A	107.	C	115.	E
84.	A	92.	B	100.	C	108.	D	116.	H
85.	A	93.	D	101.	C	109.	D	117.	D
86.	B	94.	E	102.	B	110.	A	118.	D
87.	B	95.	C	103.	D	111.	B	119.	A
88.	E	96.	E	104.	E	112.	B	120.	D
