# Supplementary material for: Benchmarking large language models on the United States medical licensing examination for clinical reasoning and medical licensing scenarios
Source: Sci Rep. 2025 Dec 3;16:1387. doi: 10.1038/s41598-025-31010-4 (PMC12796295; doi:10.1038/s41598-025-31010-4)
Supplement: Supplementary file 3 — Supplementary Information 3. [file 41598_2025_31010_MOESM3_ESM.pdf]

Sample Test Questions

Step 3

A Joint Program of the Federation of State Medical Boards of the United States, Inc. (FSMB), and National Board of Medical Examiners® (NBME®)

This booklet was updated December 2023.

Copyright © 2023 by the Federation of State Medical Boards of the United States, Inc. (FSMB), and National Board of Medical Examiners® (NBME®). All rights reserved. Printed in the United States of America. The United States Medical Licensing Examination® (USMLE®) is a joint program of the FSMB and NBME.

CONTENTS

USMLE Step 3 Multiple Choice Test Question Formats 2
Introduction to USMLE Step 3 Sample Test Questions.. 5
USMLE Step 3 Normal Laboratory Values... 6
Answer Form for USMLE Step 3 Sample Questions... 9
USMLE Step 3 Sample Test Questions 10
Answer Key for USMLE Step 3 Sample Questions 95

USMLE STEP 3 MULTIPLE-CHOICE TEST QUESTION FORMATS

The following are strategies for answering one-best-answer questions (eg, Single Items, Multiple Item Sets, and Sequential Item Sets):

- Read each patient vignette and question carefully. It is important to understand what is being asked.
- Try to generate an answer and then look for it in the option list.
- Alternatively, read each option carefully, eliminating those that are clearly incorrect. Of the remaining options, select the one that is most correct.
- If unsure about an answer, it is better to guess since unanswered questions are automatically counted as wrong answers.

Patient Scenario Formats

Patient scenarios for any Single Item or Sequential Item Set may be provided in either Vignette (paragraph) format, or in Chart/Tabular format. Questions written in Chart/Tabular format will contain relevant patient information in list form, organized in clearly marked sections for ease of review. Familiar medical abbreviations may be used within Chart/Tabular format questions.

Single-Item Questions

A single patient-centered scenario is associated with one question followed by four or more response options. The response options for all questions are lettered (ie, A, B, C, D, E). You are required to select the best answer to the question. Other options may be partially correct, but there is only ONE BEST answer. This is the traditional, most frequently used multiple-choice question format on the examination.

Example Question 1

1. A 30-year-old man comes to the emergency department because of an acute episode of renal colic. Medical history is remarkable for episodes of painful urination and passing of what he calls "gravel in my urine." Urinalysis demonstrates microscopic hematuria with some crystalluria and no casts. Supine x-ray of the abdomen shows no abnormalities. A 4-mm renal calculus is detected in the distal right ureter on ultrasonography. There is no evidence of dilation of the collecting system. The patient's pain is responsive to narcotic medication. In addition to administering intravenous fluids, which of the following is the most appropriate next step?

   A. Acidification of urine by drinking cranberry juice
   B. Cystoscopic removal of the calculus
   C. Cystoscopic ureteral lavage
   D. Shock wave lithotripsy
   E. Straining of the urine
   (Answer: E)

Sequential Item Sets

A single patient-centered scenario may be associated with two or three consecutive questions about the information presented. Each question is associated with the initial patient scenario but is testing a different point. You are required to select the ONE BEST answer to each question. Questions are designed to be answered in sequential order. You must click "Proceed to Next Item" to view the next question in the set; once you click on this button, the next question will be displayed, and you will not be able to add or change an answer to the previous question.

Example Questions 4 to 5

A 2-year-old girl is brought to the office by her mother for evaluation of fever. You have been the girl's physician since birth. While in the office, the girl stiffens and then has bilateral, symmetrical shaking of her upper and lower extremities; she becomes mildly cyanotic. The episode lasts for approximately 45 seconds, after which she becomes relaxed and appears to fall asleep. Vital signs at this time are temperature 40.0°C (104.0°F), pulse 120/min, and respirations 40/min. On physical examination she has a generally pink complexion and flushed cheeks. She is limp and somnolent and responds with a cry to noxious stimulus. Tympanic membranes are inflamed bilaterally, nose has a scant, clear discharge, and throat is mildly erythematous. Lungs are clear to auscultation except for transmitted upper airway sounds. Heart has rapid rate with a grade 1/6 systolic murmur at the left sternal border. Complete blood count, blood culture, lumbar puncture, and catheterized urine specimen are obtained and sent for stat analysis. Acetaminophen is administered by rectal suppository. Thirty minutes later the patient awakens and is smiling. She is afebrile. Additional history discloses that she was born at term, she had an uneventful neonatal course, she has normal growth and development, and vaccinations are up-to-date. She has never had an episode similar to this. Initial laboratory results are shown:

Blood	
WBC	10,400/mm^3
Neutrophils, segmented	25%
Neutrophils, bands	5%
Lymphocytes	65%
Monocytes	5%
Cerebrospinal fluid	0 RBC/mm^3
Urinalysis	Normal

Other laboratory studies are pending.

4. In addition to ampicillin for otitis media and acetaminophen, this child also should receive which of the following?

   A. Oral ethosuximide
   B. Oral phenobarbital
   C. Oral phenytoin
   D. Rectal diazepam
   E. No additional medications
   (Answer E)

5. Two weeks later the patient is brought to the office for a follow-up visit. Her mother says that she is doing well and she has had no recurrence of her symptoms. Examination of the ears shows resolution of the otitis media. Which of the following is the most important diagnostic step at this time?

   A. Audiology testing
   B. Cognitive testing
   C. CT scan of the head
   D. EEG
   E. No additional testing
   (Answer E)

INTRODUCTION TO USMLE STEP 3 SAMPLE TEST QUESTIONS

The following pages include 137 sample test questions. Please note that reviewing the sample questions is not a substitute for acquainting yourself with the test software. You should run the Step 3 tutorial and practice test questions that are provided on the USMLE website well before your test date. The sample materials on the USMLE website include additional item formats that do not appear in this booklet (eg, items with associated audio findings). Note that the function of items such as pharmaceutical ads, abstracts, and sequential item sets is unique in an examination interface. You should become familiar with all test question/item formats as they will be used in the actual examination.

In addition, the computer-based case simulation (CCS) format you will see on an actual Step 3 examination is not represented in this booklet. You must become familiar with the CCS format by reading information available in the *USMLE Content Description and General Information* booklet and by practicing with sample CCS cases before you take the Step 3 examination; the information and the practice materials are available on the USMLE Web site (www.usmle.org).

These sample questions are illustrative of the types of questions used in the Step 3 examination. Although the questions exemplify content on the examination, they may not reflect the content coverage on individual examinations. Questions are grouped together by the content appropriate for each examination day in the same manner as in the actual computer-administered test blocks. In the actual examination, the questions will be presented one at a time in a format designed for easy on-screen reading, including use of a panel for the table of normal laboratory values (included here on pages 6–8) and some pictorials. Photographs, charts, and x-rays referred to in this booklet are not of the same quality as the pictorials used in the actual examination. In addition, you will have the capability to adjust the brightness and contrast of pictorials on the computer screen.

To take the following sample test questions as they would be timed in the actual examination, you should allow a maximum of 1 hour for each of the Foundations of Independent Practice (FIP) blocks, and a maximum of 45 minutes for each of the Advanced Clinical Medicine (ACM) blocks, for a total of 3 hours 30 minutes. Please be aware that most examinees perceive the time pressure to be greater during an actual examination. An answer sheet for recording answers for this practice is provided on page 9. An answer key is provided on page 95. In the actual examination, answers will be selected on the screen; **no answer form will be provided**.

USMLE LABORATORY VALUES

	Reference Range	SI Reference Intervals
**SERUM**		
General Chemistry:		
Electrolytes		
Sodium (Na^+)	136–146 mEq/L	136–146 mmol/L
Potassium (K^+)	3.5–5.0 mEq/L	3.5–5.0 mmol/L
Chloride (Cl^-)	95–105 mEq/L	95–105 mmol/L
Bicarbonate (HCO_3^-)	22–28 mEq/L	22–28 mmol/L
Urea nitrogen	7–18 mg/dL	2.5–6.4 mmol/L
Creatinine	0.6–1.2 mg/dL	53–106 µmol/L
Glucose	Fasting: 70–100 mg/dL	3.8–5.6 mmol/L
	Random, non-fasting: <140 mg/dL	<7.77 mmol/L
Calcium	8.4–10.2 mg/dL	2.1–2.6 mmol/L
Magnesium (Mg^{2+})	1.5–2.0 mg/dL	0.75–1.0 mmol/L
Phosphorus (inorganic)	3.0–4.5 mg/dL	1.0–1.5 mmol/L
Hepatic:		
Alanine aminotransferase (ALT)	10–40 U/L	10–40 U/L
Aspartate aminotransferase (AST)	12–38 U/L	12–38 U/L
Alkaline phosphatase	25–100 U/L	25–100 U/L
Amylase	25–125 U/L	25–125 U/L
Bilirubin, Total // Direct	0.1–1.0 mg/dL // 0.0–0.3 mg/dL	2–17 µmol/L // 0–5 µmol/L
Proteins, total	6.0–7.8 g/dL	60–78 g/L
Albumin	3.5–5.5 g/dL	35–55 g/L
Globulin	2.3–3.5 g/dL	23–35 g/L
Lipids:		
Cholesterol		
Total	Normal: <200 mg/dL	<5.2 mmol/L
	High: >240 mg/dL	>6.2 mmol/L
HDL	40–60 mg/dL	1.0–1.6 mmol/L
LDL	<160 mg/dL	<4.2 mmol/L
Triglycerides	Normal: <150 mg/dL	<1.70 mmol/L
	Borderline: 151–199 mg/dL	1.71–2.25 mmol/L
Iron Studies:		
Ferritin	Male: 20–250 ng/mL	20–250 µg/L
	Female: 10–120 ng/mL	10–120 µg/L
Iron	Male: 65–175 µg/dL	11.6–31.3 µmol/L
	Female: 50–170 µg/dL	9.0–30.4 µmol/L
Total iron-binding capacity	250–400 µg/dL	44.8–71.6 µmol/L
Transferrin	200–360 mg/dL	2.0–3.6 g/L

Continued on Next Page

USMLE LABORATORY VALUES (continued)

	Reference Range	SI Reference Intervals
Endocrine:		
Follicle-stimulating hormone	Male: 4–25 mIU/mL	4–25 IU/L
	Female: premenopause 4–30 mIU/mL	4–30 IU/L
	midcycle peak 10–90 mIU/mL	10–90 IU/L
	postmenopause 40–250 mIU/mL	40–250 IU/L
Luteinizing hormone	Male: 6–23 mIU/mL	6–23 IU/L
	Female: follicular phase 5–30 mIU/mL	5–30 IU/L
	midcycle 75–150 mIU/mL	75–150 IU/L
	postmenopause 30–200 mIU/mL	30–200 IU/L
Growth hormone - arginine stimulation	Fasting: <5 ng/mL	<5 µg/L
	Provocative stimuli: >7 ng/mL	>7 µg/L
Prolactin (hPRL)	Male: <17 ng/mL	<17 µg/L
	Female: <25 ng/mL	<25 µg/L
Cortisol	0800 h: 5–23 µg/dL	138–635 nmol/L
	1600 h: 3–15 µg/dL	82–413 nmol/L
	2000 h: <50% of 0800 h	Fraction of 0800 h: <0.50
TSH	0.4–4.0 µU/mL	0.4–4.0 mIU/L
Triiodothyronine (T_3) (RIA)	100–200 ng/dL	1.5–3.1 nmol/L
Triiodothyronine (T_3) resin uptake	25%–35%	0.25–0.35
Thyroxine (T_4)	5–12 µg/dL	64–155 nmol/L
Free T_4	0.9–1.7 ng/dL	12.0–21.9 pmol/L
Thyroidal iodine (^{123}I) uptake	8%–30% of administered dose/24 h	0.08–0.30/24 h
Intact PTH	10–60 pg/mL	10–60 ng/L
17-Hydroxycorticosteroids	Male: 3.0–10.0 mg/24 h	8.2–27.6 µmol/24 h
	Female: 2.0–8.0 mg/24 h	5.5–22.0 µmol/24 h
17-Ketosteroids, total	Male: 8–20 mg/24 h	28–70 µmol/24 h
	Female: 6–15 mg/24 h	21–52 µmol/24 h
Immunoglobulins:		
IgA	76–390 mg/dL	0.76–3.90 g/L
IgE	0–380 IU/mL	0–380 kIU/L
IgG	650–1500 mg/dL	6.5–15.0 g/L
IgM	50–300 mg/dL	0.5–3.0 g/L
Other, serum:		
Creatinine clearance	Male: 97–137 mL/min	97–137 mL/min
	Female: 88–128 mL/min	88–128 mL/min
Creatine kinase	Male: 25–90 U/L	25–90 U/L
	Female: 10–70 U/L	10–70 U/L
Lactate dehydrogenase	45–200 U/L	45–200 U/L
Osmolality	275–295 mOsmol/kg H_2O	275–295 mOsmol/kg H_2O
Uric acid	3.0–8.2 mg/dL	0.18–0.48 mmol/L
**GASES, ARTERIAL BLOOD (ROOM AIR)**		
P_{O_2}	75–105 mm Hg	10.0–14.0 kPa
P_{CO_2}	33–45 mm Hg	4.4–5.9 kPa
pH	7.35–7.45	[H^+] 36–44 nmol/L
**CEREBROSPINAL FLUID**		
Cell count	0–5/mm^3	0–5 × 10^6/L
Chloride	118–132 mEq/L	118–132 mmol/L
Gamma globulin	3%–12% total proteins	0.03–0.12
Glucose	40–70 mg/dL	2.2–3.9 mmol/L
Pressure	70–180 mm H_2O	70–180 mm H_2O
Proteins, total	<40 mg/dL	<0.40 g/L

Continued on Next Page

USMLE LABORATORY VALUES (continued)

	Reference Range	SI Reference Intervals
**HEMATOLOGIC**		
Complete Blood Count:		
Hematocrit	Male: 41%–53%	0.41–0.53
	Female: 36%–46%	0.36–0.46
Hemoglobin, blood	Male: 13.5–17.5 g/dL	135–175 g/L
	Female: 12.0–16.0 g/dL	120–160 g/L
Mean corpuscular hemoglobin (MCH)	25–35 pg/cell	0.39–0.54 fmol/cell
Mean corpuscular hemoglobin conc. (MCHC)	31%–36% Hb/cell	4.8–5.6 mmol Hb/L
Mean corpuscular volume (MCV)	80–100 μm^3	80–100 fL
Volume		
Plasma	Male: 25–43 mL/kg	0.025–0.043 L/kg
	Female: 28–45 mL/kg	0.028–0.045 L/kg
Red cell	Male: 20–36 mL/kg	0.020–0.036 L/kg
	Female: 19–31 mL/kg	0.019–0.031 L/kg
Leukocyte count (WBC)	4500–11,000/mm^3	4.5–11.0 × 10^9/L
Neutrophils, segmented	54%–62%	0.54–0.62
Neutrophils, bands	3%–5%	0.03–0.05
Lymphocytes	25%–33%	0.25–0.33
Monocytes	3%–7%	0.03–0.07
Eosinophils	1%–3%	0.01–0.03
Basophils	0%–0.75%	0.00–0.0075
Platelet count	150,000–400,000/mm^3	150–400 × 10^9/L
Coagulation:		
Partial thromboplastin time (PTT) (activated)	25–40 seconds	25–40 seconds
Prothrombin time (PT)	11–15 seconds	11–15 seconds
D-Dimer	≤250 ng/mL	≤1.4 nmol/L
Other, Hematologic:		
Reticulocyte count	0.5%–1.5%	0.005–0.015
Erythrocyte count (RBC)	Male: 4.3–5.9 million/mm^3	4.3–5.9 × 10^{12}/L
	Female: 3.5–5.5 million/mm^3	3.5–5.5 × 10^{12}/L
Erythrocyte sedimentation rate (Westergren)	Male: 0–15 mm/h	0–15 mm/h
	Female: 0–20 mm/h	0–20 mm/h
CD4+ T-lymphocyte count	≥500/mm^3	≥0.5 × 10^9/L
Troponin I	≤0.04 ng/mL	≤0.04 µg/L
Endocrine:		
Hemoglobin A_{1c}	≤6%	≤42 mmol/mol
**URINE**		
Calcium	100–300 mg/24 h	2.5–7.5 mmol/24 h
Osmolality	50–1200 mOsmol/kg H_2O	50–1200 mOsmol/kg H_2O
Oxalate	8–40 µg/mL	90–445 µmol/L
Proteins, total	<150 mg/24 h	<0.15 g/24 h
**BODY MASS INDEX (BMI)**	Adult: 19–25 kg/m^2	

ANSWER FORM FOR USMLE STEP 3 SAMPLE QUESTIONS

Block 1 (Questions 1–38): FIP

1. ______	9. ______	17. ______	25. ______	33. ______
2. ______	10. ______	18. ______	26. ______	34. ______
3. ______	11. ______	19. ______	27. ______	35. ______
4. ______	12. ______	20. ______	28. ______	36. ______
5. ______	13. ______	21. ______	29. ______	37. ______
6. ______	14. ______	22. ______	30. ______	38. ______
7. ______	15. ______	23. ______	31. ______	
8. ______	16. ______	24. ______	32. ______	

Block 2 (Questions 39–77): FIP

39. ______	47. ______	55. ______	63. ______	71. ______
40. ______	48. ______	56. ______	64. ______	72. ______
41. ______	49. ______	57. ______	65. ______	73. ______
42. ______	50. ______	58. ______	66. ______	74. ______
43. ______	51. ______	59. ______	67. ______	75. ______
44. ______	52. ______	60. ______	68. ______	76. ______
45. ______	53. ______	61. ______	69. ______	77. ______
46. ______	54. ______	62. ______	70. ______	

Block 3 (Questions 78–107): ACM

78. ______	84. ______	90. ______	96. ______	102. ______
79. ______	85. ______	91. ______	97. ______	103. ______
80. ______	86. ______	92. ______	98. ______	104. ______
81. ______	87. ______	93. ______	99. ______	105. ______
82. ______	88. ______	94. ______	100. ______	106. ______
83. ______	89. ______	95. ______	101. ______	107. ______

Block 4 (Questions 108–137): ACM

108. ______	114. ______	120. ______	126. ______	132. ______
109. ______	115. ______	121. ______	127. ______	133. ______
110. ______	116. ______	122. ______	128. ______	134. ______
111. ______	117. ______	123. ______	129. ______	135. ______
112. ______	118. ______	124. ______	130. ______	136. ______
113. ______	119. ______	125. ______	131. ______	137. ______

USMLE STEP 3 SAMPLE TEST QUESTIONS

GENERAL INSTRUCTIONS: Read each question carefully and in the order in which it is presented. Then select the one best response option of the choices offered. More than one option may be partially correct. You must select the **ONE BEST** answer and fill in the corresponding blank line on the answer sheet.

Some items are grouped together around a clinical vignette as a set or case; be particularly careful to read and answer these cases or sets of items in the order they are presented.

The items in this exam are divided into blocks according to the day they will appear on the actual Step 3 examination. The first day of the Step 3 examination is referred to as Foundations of Independent Practice (FIP), and the second day is referred to as Advanced Clinical Medicine (ACM).

Block 1:	Foundations of Independent Practice (FIP)	Items 1–38
Block 2:	Foundations of Independent Practice (FIP)	Items 39–77
Block 3:	Advanced Clinical Medicine (ACM)	Items 78–107
Block 4:	Advanced Clinical Medicine (ACM)	Items 108–137

Block 1: FIP

Items 1–38; Time - 1 hour

ALL ITEMS REQUIRE SELECTION OF ONE BEST CHOICE.

1. A 75-year-old man is brought to the emergency department by his son 2 hours after the sudden onset of fever, chills, pleuritic chest pain, and cough productive of rust-colored sputum. He rates his chest pain as an 8 on a 10-point scale. Temperature is 38.9°C (102°F), pulse is 106/min, respirations are 22/min, and blood pressure is 130/80 mm Hg. Oxygen saturation is 94% on room air. The patient appears to be in moderate respiratory distress. Physical examination shows splinting on the left side. There is dullness to percussion and egophony over the left lower lobe. Abdominal examination shows no abnormalities. Results of laboratory studies are shown:

Serum	
Calcium	8.4 mg/dL
Urea nitrogen	18 mg/dL
Creatinine	1.4 mg/dL
Na^+	131 mEq/L
K^+	4.1 mEq/L
Cl^-	108 mEq/L
HCO_3^-	25 mEq/L
Blood	
Hematocrit	37%
Hemoglobin	12.4 g/dL
WBC	21,000/mm^3
Neutrophils, segmented	79%
Neutrophils, bands	10%
Lymphocytes	11%
Platelet count	250,000/mm^3
Arterial blood gas analysis on room air:	
pH	7.45
P_{CO_2}	45 mm Hg
P_{O_2}	62 mm Hg
HCO_3^-	24 mEq/L
O_2 saturation	95%

Urinalysis shows no abnormalities. An ECG shows sinus tachycardia. A chest x-ray shows consolidation in the left lower lobe and no cardiomegaly. Intravenous antibiotics are administered, and the patient receives oxygen via nasal cannula. Three hours later, he is lying on his left side and has increased dyspnea; he is rolled to his right side and his symptoms improve within minutes. Which of the following best explains this improvement?

(A) Positionally apparent pulmonary emboli
(B) Positionally decreased alveolar-arterial gradient
(C) Positionally impeded filling of the left ventricle
(D) Positionally impeded movement of the diaphragm
(E) Positionally increased left pleural effusion

2. A 55-year-old woman, gravida 4, para 4, comes to the office because of vaginal spotting that has occurred once monthly for the past 2 months. Menopause occurred 1 year ago, and this is the patient's first postmenopausal examination. Medical history is significant for elevated blood pressure readings during the past 2 years and diabetes mellitus for which she follows an 1800-calorie diet. She takes no medications. Her most recent cervical cytology and mammography obtained 1 year ago disclosed no abnormalities. Family history is significant for hypertension in her mother who died at age 58 years from an unknown cancer. The patient has smoked one pack of cigarettes daily for the past 30 years and drinks beer occasionally. BMI is 42 kg/m^2. Temperature is 36.7°C (98.0°F), pulse is 90/min, respirations are 16/min, and blood pressure is 136/84 mm Hg. Bimanual examination discloses a firm, smooth, mobile, nontender uterus that is consistent in size with a 6-week gestation. The remainder of the physical examination discloses no abnormalities. Results of dipstick urinalysis are within the reference ranges; fingerstick hematocrit is 35% and 2-hour postprandial serum glucose concentration is 188 mg/dL. Which of the following is the most appropriate diagnostic study at this time?

(A) Complete blood count
(B) CT scan of the pelvis
(C) Endometrial biopsy
(D) Hysterosalpingography
(E) Serum CA 125 concentration

3. A 28-year-old woman comes to the health center for an initial visit. The patient appears somewhat anxious. After introductions she says, "Doctor, something has been happening to me for the past 6 weeks or so that has me a bit scared. About once or twice a week, and out of nowhere and for no reason, I have this sudden feeling of anxiety. It really gets severe and I get short of breath. I just don't know what's going on." She has recently divorced her husband of 7 years. Given the evidence at this time, which of the following is the most likely diagnosis?

(A) Adjustment disorder with anxiety
(B) Agoraphobia
(C) Dysthymic disorder
(D) Generalized anxiety disorder
(E) Posttraumatic stress disorder

4. A 71-year-old woman with a 15-year history of severe chronic obstructive pulmonary disease (COPD) is admitted to the intensive care unit for treatment of an acute COPD exacerbation. The patient has been receiving home oxygen therapy for the past 3 years; $FEV_1$1 year ago was 27%. She has a do-not-resuscitate order stating that she does not want cardiopulmonary resuscitation, with no specific comment on intubation. Her daughter, who is not present, has power of attorney for the patient's health care decisions, although the patient has made all decisions in the past. Medical history also is significant for hypertension. Medications are lisinopril and hydrochlorothiazide, as well as inhaled tiotropium, albuterol, and fluticasone. The patient is intubated and mechanical ventilation is begun. She is treated with antibiotics, corticosteroids, and nebulized bronchodilators. Her condition gradually improves and she is extubated 4 days later. Within 2 hours of extubation, the patient becomes short of breath and has difficulty clearing her secretions. She is not using accessory muscles of respiration. Temperature is 37.0°C (98.6°F), pulse is 98/min, respirations are 25/min, and blood pressure is 168/98 mm Hg. Oxygen saturation is 89% on oxygen at 6 L/min. The patient is alert, fully oriented, and conversant. Results of arterial blood gas analysis are shown:

PO_2	61 mm Hg
PCO_2	55 mm Hg
pH	7.32

An attempt is made to manage the patient with noninvasive positive pressure ventilation, but the patient reports discomfort caused by the mask and requests that it be removed. When initially asked, she declines to be reintubated. Which of the following is the most appropriate next step in management?

(A) Clearly state to the patient that if she again declines intubation when offered to her one more time, she will receive comfort care only
(B) Contact the patient's daughter to request additional information regarding the patient's previous feelings regarding intubation
(C) Explain to the patient that because her advance directive is confusing, discussing her options would be helpful
(D) Proceed with reintubating the patient
(E) Request psychiatric evaluation of the patient's capacity to make health care decisions

5. A phase 3 trial is planned to investigate the use of a new medication for the prevention of type 2 diabetes mellitus. Which of the following inclusion criteria should be selected to design the most efficient study?

(A) Patients aged 18 years and older who have a BMI of less than 23 kg/m^2 and are smokers
(B) Patients aged 18 years and older with no significant medical history
(C) Patients aged 45 years and older with hyperlipidemia and central obesity
(D) Patients aged 65 years and older with a hemoglobin A_{1c} of 6.5% or greater

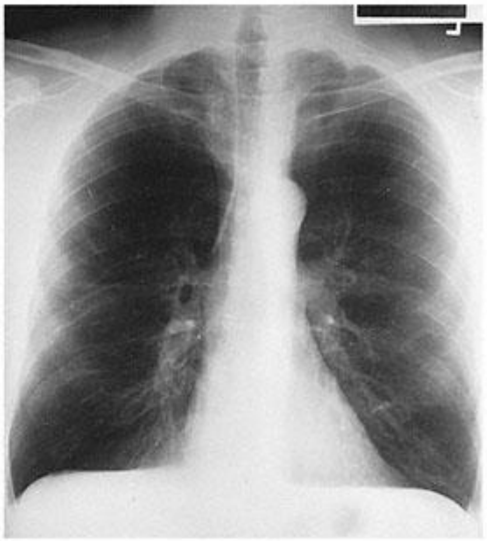

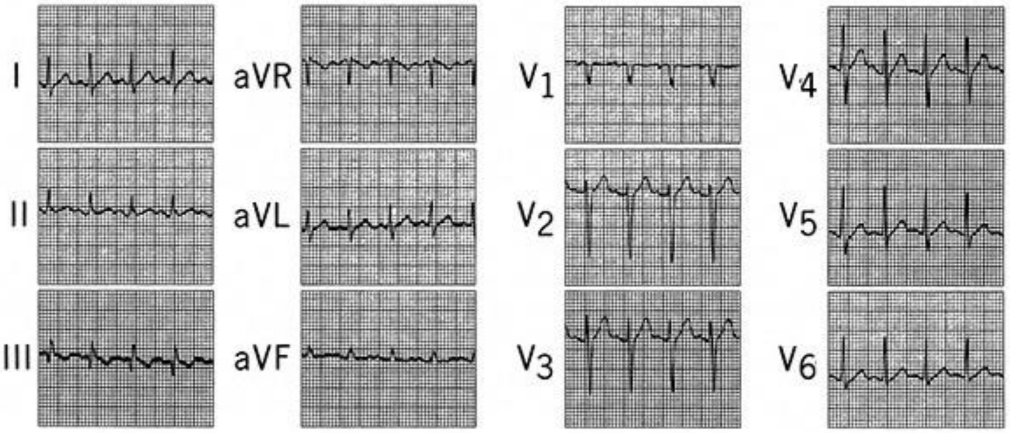

6. A 60-year-old man is admitted to the hospital 4 hours after he awoke early this morning with shortness of breath, dizziness, and pleuritic chest pain. Medical history is significant for coronary artery disease, hyperlipidemia, and chronic obstructive pulmonary disease. Medications are atorvastatin, enteric-coated 81-mg aspirin, atenolol, albuterol, ipratropium, and home oxygen at 1 L/min via nasal cannula. He has not had any recent illness or exposure to anyone known to be ill. He has smoked one pack of cigarettes daily for the past 40 years and he drinks three to four alcoholic beverages monthly. BMI is 21 kg/m^2. Temperature is 36.9°C (98.4°F), pulse is 150/min, respirations are 36/min, and blood pressure is 105/72 mm Hg. Oxygen saturation is 84% on oxygen at 1 L/min via nasal cannula. The patient appears uncomfortable and is speaking in three- to four-word sentences. Jugular venous pressure is 10 cm H_2O at 45 degrees. Auscultation of the lungs discloses poor air movement and diffuse scattered inspiratory wheezes bilaterally. Cardiac examination discloses a palpable parasternal lift. The abdomen is protuberant with a visible fluid wave and a palpable epigastric pulsation. The liver edge is palpated 3 cm below the right costal margin and the area is tender. Examination of the lower extremities shows 2+ bilateral pitting edema. Chest x-ray and ECG are shown. Which of the following is the most likely underlying explanation for this patient's condition?

(A) External compression of the superior vena cava
(B) *Helicobacter pylori* infection with transmural ulceration and peritoneal leaking
(C) Lactic acidosis caused by systemic hypoperfusion
(D) Pulmonary vasculature compromise and cor pulmonale
(E) Widespread hepatocellular necrosis and parenchymal edema

7. A 67-year-old male US military veteran is admitted to the rehabilitation facility 3 days after undergoing uncomplicated revision of his left transtibial amputation to a transfemoral amputation, which was required because of dehiscence of the previous incision. Today, the patient reports pain at the site of incision that he rates as a 3 on a 10-point scale. He reports phantom sensation but no phantom pain. He also has atrial fibrillation, type 2 diabetes mellitus, mild diabetic retinopathy, hypertension, and severe peripheral vascular disease that required aortic to bilateral iliac arterial bypass 2 years ago. Medications are dabigatran, lisinopril, metformin, and metoprolol. The patient is 172 cm (5 ft 8 in) tall and weighs 86 kg (190 lb); BMI is 29 kg/m^2. Temperature is 37.0°C (98.6°F), pulse is 68/min, respirations are 18/min, and blood pressure is 138/88 mm Hg. The patient does not appear to be in distress. Examination shows a well-healing incision. Fingerstick blood glucose concentration is 120 mg/dL. Which of the following findings on further physical examination is most likely to inhibit this patient's long-term ability to ambulate with a prosthesis?

(A) A 3-cm-diameter blackened eschar on the right heel
(B) A 5-degree left hip flexion contracture
(C) An irregular pulse
(D) Loss of proprioception in the right great toe

8. Researchers at a large health sciences center would like to evaluate the relationship between dental x-rays and risk for thyroid cancer. They plan to enroll 300 participants with thyroid cancer and 400 participants without thyroid cancer. All participants will be asked to report past exposure to dental x-rays. The study will be federally funded. Since many of the participants may have received dental x-rays at the institution, a member of the institutional review board (IRB) is concerned about increased liability arising from the results of the study, especially if a positive association between dental x-rays and thyroid cancer is found. Which of the following is the most appropriate response by the IRB regarding the member’s concern?

(A) Approve the study as submitted
(B) Do not approve the study
(C) Require language in the informed consent document advising participants of the right to sue should a positive association between dental x-rays and thyroid cancer be found
(D) Require language in the informed consent document releasing the institution from liability

9. A 39-year-old man comes to the emergency department because of a 1-day history of progressively worsening generalized itchiness of his skin. He also has had pain with urination and blood in his urine during the past 5 days. He has not had fever, chills, nausea, vomiting, flank pain, or diarrhea. Two days ago he was seen in the emergency department and diagnosed with renal calculi. Medical history otherwise is remarkable for psoriasis. His current medications are oxycodone-ibuprofen, tamsulosin, and topical clobetasol. Vital signs are temperature 36.7°C (98.0°F), pulse 76/min, respirations 12/min, and blood pressure 118/74 mm Hg. Examination of the skin shows multiple excoriations over the back and abdomen; no lesions are noted. The remainder of the physical examination discloses no abnormalities. Results of urinalysis are shown:

Specific gravity	1.010 (N=1.003–1.029)
Bilirubin	Trace positive
Blood	3+
Leukocyte esterase	Negative
RBCs	Positive

Which of the following is the most likely cause of this patient's pruritus?

(A) Exacerbation of psoriasis
(B) Obstruction of the common bile duct
(C) Oxycodone therapy
(D) Tamsulosin therapy
(E) Ureteral obstruction

10. A 72-year-old man is admitted to the hospital through the emergency department because of a 12-hour history of fever, chills, increasingly severe abdominal pain, nausea, and vomiting. Medical history includes hypertension, hypercholesterolemia, and atherosclerotic heart disease with placement of a coronary artery stent 3 years ago. Current medications are atorvastatin and lisinopril. He appears anxious and pale. BMI is 30 kg/m^2. Temperature is 40.0°C (104.0°F), pulse is 116/min, respirations are 20/min, and blood pressure is 100/65 mm Hg. Oxygen saturation is 94% on room air. A bruit is heard over the right carotid artery. Cardiac examination discloses no murmurs; an S_4 is present. Lungs are clear to auscultation. Abdominal examination discloses decreased bowel sounds and diffuse tenderness with guarding that is most severe in the left upper quadrant. Both lower extremities are cool to touch with weak pedal pulses. Abdominal x-ray shows dilation of the proximal colon. Results of serum laboratory studies are shown:

Amylase	230 U/L
Urea nitrogen	20 mg/dL
Creatinine	1.0 mg/dL
Na^+	138 mEq/L
K^+	3.5 mEq/L
Cl^-	101 mEq/L
HCO_3^-	12 mEq/L
Glucose	180 mg/dL

Surgical resection of the colon is done. Which of the following histopathologic changes will most likely be identified in the resected colon?

(A) Caseating granulomatous inflammation with serosal fibrous adhesions
(B) Coagulative necrosis involving mucosa and submucosa
(C) Neutrophilic infiltrates in the mucosa with venous congestion and edema
(D) Patchy mucopurulent exudate with exploding glandular crypts
(E) Transmural chronic inflammation with ulcerations extending into submucosa

11. A 37-year-old woman comes to the emergency department because of a 4-hour history of severe right-sided abdominal pain. She describes the pain as sharp and says it radiates to the pelvic region; she rates the pain as a 9 on a 10-point scale. She also has had associated nausea but has not vomited. She has not had any recent trauma, fever, chills, changes in bowel habits, or urinary symptoms. She took ibuprofen 3 hours ago but has had no relief of her current symptoms. Medical history is otherwise unremarkable and she takes no other medications. She does not smoke cigarettes or drink alcoholic beverages. She is not sexually active and her last menstrual period ended 4 days ago. The patient is holding her right side and is grimacing in pain. Temperature is 37.2°C (99.0°F), pulse is 80/min, respirations are 22/min, and blood pressure is 136/74 mm Hg. Auscultation of the chest discloses normal breath sounds and normal S_1 and S_2. Abdominal examination discloses normal bowel sounds, tenderness with voluntary guarding on the right side, but no palpable masses. Pain is elicited on percussion over the right costovertebral angle. Results of laboratory studies are shown:

Serum	
Urea nitrogen	16 mg/dL
Creatinine	0.8 mg/dL
Calcium	9.8 mg/dL
Blood	
Hemoglobin	13.9 g/dL
WBC	13,000/mm^3
Neutrophils	68%
Lymphocytes	27%
Monocytes	3%
Eosinophils	2%
Urine	
Specific gravity	1.010 (N=1.003–1.029)
Occult blood	Trace positive
pH	6.0
Glucose	Negative
Protein	Negative

Intravenous fluids and analgesic therapy are initiated. Which of the following is the most appropriate next step in evaluation?

(A) Cystoscopy
(B) Exploratory laparotomy
(C) Helical CT scan of the abdomen
(D) X-ray of the kidney, ureter, and bladder
(E) No further testing is indicated at this time

12. A 75-year-old woman is brought to the emergency department by her husband 60 minutes after she "fainted" while standing at the bathroom sink. The husband witnessed her fainting spell and says she lost consciousness for approximately 15 to 30 seconds. He says she was alert upon awakening but looked pale. The patient did not have any tonic-clonic movements and sustained no traumatic injuries during the fall. She did not have palpitations or other warning signs prior to losing consciousness and has not had slurred speech or abnormalities of movement upon awakening. Medical history is significant for hypertension and gout. Medications include lisinopril, metoprolol, 81-mg aspirin, and allopurinol. She has no drug allergies. She does not smoke cigarettes or drink alcoholic beverages. The patient is in no acute distress. Temperature is 37.0°C (98.6°F), pulse is 68/min, respirations are 15/min, and blood pressure is 162/74 mm Hg. Standing pulse and blood pressure are 70/min and 172/75 mm Hg, respectively. Pulse and blood pressure after standing for 2 minutes are 68/min and 148/58 mm Hg, respectively. Examination of the neck discloses jugular venous distention, 5 cm at 30 degrees; there are no carotid bruits. Lungs are clear to auscultation. Cardiac examination discloses a regular rhythm with a soft systolic ejection murmur heard best at the left upper sternal border. There is no peripheral edema and pulses are 2+ bilaterally. Neurologic examination and ECG disclose no abnormalities. Serum troponin I concentration is 0.1 ng/mL (N<0.35). Results of the remaining laboratory studies are within the reference ranges. CT scan of the head shows no abnormalities. Which of the following is the most appropriate next step in evaluation?

(A) Electroencephalography
(B) Outpatient ambulatory ECG monitoring (24-hour)
(C) Outpatient echocardiography
(D) Telemetry observation
(E) Tilt test

13. A 52-year-old man comes to the emergency department because of fever and nonpruritic lesions that suddenly developed over his legs 2 hours ago. He has not had any gastrointestinal symptoms. Medical history is significant for type 2 diabetes mellitus and cirrhosis secondary to hemochromatosis. Medications include insulin and spironolactone. Vaccinations are up-to-date. The patient returned home 1 day ago from a vacation in a southeastern state. He has had no known contact with anyone who has been ill. On arrival, temperature is 38.6°C (101.5°F), pulse is 118/min, respirations are 22/min, and blood pressure is 84/50 mm Hg. Oxygen saturation is 93% on room air. Lungs are clear to auscultation. Cardiac examination discloses a grade 2/6 systolic murmur heard best along the left sternal border. Spider angiomas are present over the torso. Abdomen is distended and diffusely dull to percussion. Palpation of the abdomen does not disclose tenderness. The liver and spleen are not palpated. The lower extremities are erythematous and warm to the touch; hemorrhagic bullae are present from the dorsum of the feet to the knees bilaterally. Results of laboratory studies are shown:

Serum		Blood	
ALT	68 U/L	Hematocrit	33%
AST	61 U/L	Hemoglobin	11.0 g/dL
Urea nitrogen	31 mg/dL	WBC	16,000/mm^3
Creatinine	2.1 mg/dL	Neutrophils, segmented	85%
Na^+	127 mEq/L	Neutrophils, bands	4%
K^+	5.0 mEq/L	Lymphocytes	7%
Cl^-	95 mEq/L	Monocytes	4%
HCO_3^-	16 mEq/L	Platelet count	83,000/mm^3
Lactate dehydrogenase	166 U/L		
Glucose	354 mg/dL		

Which of the following microorganisms is the most likely causal agent of this patient's condition?

(A) *Enterobacter aerogenes*
(B) *Enterococcus faecalis*
(C) *Mycobacterium marinum*
(D) *Vibrio vulnificus*

14. A 58-year-old man comes to the office for follow-up of an 8-month history of blood pressure readings ranging between 150 and 180 mm Hg systolic, and 85 and 100 mm Hg diastolic. Medical history is otherwise unremarkable. Medications include 20-mg lisinopril, 100-mg atenolol, 25-mg hydrochlorothiazide, and amlodipine. At the patient's last visit 6 weeks ago, the dose of amlodipine was increased from 5 mg to 10 mg. The patient does not smoke cigarettes and drinks alcoholic beverages socially. BMI is 24 kg/m^2. Temperature is 37.0°C (98.6°F), pulse is 86/min, respirations are 14/min, and blood pressure is 176/102 mm Hg. Physical examination discloses no abnormalities. Results of laboratory studies are shown:

Serum	
Urea nitrogen	18 mg/dL
Creatinine	1.0 mg/dL
Na^+	140 mEq/L
K^+	4.2 mEq/L
Cl^-	105 mEq/L
HCO_3^-	25 mEq/L

Specific additional history should be obtained regarding which of the following?

(A) Caffeine use
(B) Exercise history
(C) Frequency of fast food consumption
(D) Refill patterns on medications

15. A 65-year-old man comes to the office because of a 4-month history of increasingly severe joint pain. Medical history is significant for an acute anterior myocardial infarction 5 years ago. Current medications are metoprolol and 81-mg aspirin. Active and passive motion of the elbow and knee joints elicits pain. A diagnosis of osteoarthritis is made. The patient says that a colleague of his recommended celecoxib to treat his pain. The concerns with adding celecoxib to this patient's medication regimen are related to inhibition of which of the following processes?

(A) Both cyclooxygenase-1 (COX-1) and cyclooxygenase-2 (COX-2) decreasing prostanoid production
(B) COX-1 decreasing prostacyclin (PGI_2) production
(C) COX-1 decreasing thromboxane A_2 production
(D) COX-2 decreasing prostacyclin (PGI_2) production
(E) COX-2 decreasing thromboxane A_2 production

16. The quality improvement department of a hospital has received several incident reports indicating that patients admitted to the hospital have received a different amount of oxygen than was originally ordered by the admitting physician. On admission, the current practice for ordering oxygen therapy is that the admitting physician includes oxygen in the written admission orders. An example of admission orders for oxygen is shown:

Oxygen: 2 liters via nasal cannula with goal of oxygen saturation >90%

After patients are admitted to the hospital, the amount of oxygen a patient receives is based upon physician and nurse discussion, which is not usually documented in the medical record as an additional order. In addition to reviewing the hospital's protocol for oxygen delivery, instituting which of the following measures would most likely decrease discrepancies in oxygen delivery without creating additional risks?

(A) Conducting an in-service program on oxygen use and documentation of orders
(B) Creating a standard set of oxygen orders that includes initial dose, titration parameters, and goal dose
(C) Encouraging the nurses to refrain from adjusting the patient's oxygen dose without a written order from the physician
(D) Requiring nurses to enter verbal orders in the electronic medical record and requiring physician signature within 24 hours
(E) Requiring nurses to telephone a physician prior to any deviation from the original admission order for oxygen therapy

17. A 21-year-old ballet dancer comes to the office because she and her husband would like to conceive. She has not had a menstrual period during the past 8 months. She runs 13 km (8 mi) daily. The patient identifies as White. She is 165 cm (5 ft 5 in) tall and weighs 48 kg (105 lb); BMI is 18 kg/m^2. Temperature is 36.1°C (97.0°F), pulse is 52/min, respirations are 20/min, and blood pressure is 90/70 mm Hg. Physical examination discloses a thin woman. The remainder of the physical examination shows no abnormalities. The patient is advised to gain weight and to decrease the amount of strenuous exercise. She returns for follow-up 3 months later. She says she still has not had a period even though she has gained 2 kg (4.4 lb). Which of the following is the most likely cause of this patient's amenorrhea?

(A) Addison disease
(B) Hypothalamic hypogonadism
(C) Partial hypopituitarism
(D) Polycystic ovarian syndrome
(E) Premature ovarian failure

18. A 19-year-old woman who is at 32 weeks' gestation comes to the clinic for a prenatal visit. She says, "I tried to quit smoking as soon as I knew I was pregnant," but she admits that she has only been able to decrease her smoking to 5 to 10 cigarettes daily. Her partner has continued to smoke during the pregnancy. The patient has a history of cocaine use but her urine screening studies have been negative during this pregnancy. The patient is unemployed and dropped out of school in the 11th grade. She has been receiving supplemental nutritional foods during this pregnancy. She says she plans to bottle-feed this baby because her friends have told her, "Breast-feeding hurts you, and besides, formula makes babies grow faster." Which of the following factors in this patient's history places her infant at greatest risk for sudden infant death syndrome (SIDS)?

(A) Employment status
(B) History of cocaine use
(C) Lack of breast-feeding
(D) Maternal smoking

19. **Patient Information**
Age: 66 years
Gender: F, self-identified
Race/Ethnicity: unspecified
Site of Care: office

The patient presents because of a 3-day history of increasingly severe pain when swallowing solids and liquids. Use of over-the-counter antacids has not resulted in relief. There is no associated nausea, vomiting, or loss of appetite. Medical history is remarkable for hypertension, diet-controlled type 2 diabetes mellitus, migraine, and osteoporosis. Current medications are alendronate, chlorthalidone, propranolol, sumatriptan, and calcium citrate with vitamin D supplements. She has smoked one-half pack of cigarettes daily for 10 years and drinks one to two alcoholic beverages monthly. She does not use other substances. BMI is 24 kg/m^2. Temperature is 37.0°C (98.6°F), pulse is 84/min, respirations are 16/min, and blood pressure is 130/90 mm Hg. Oxygen saturation is 98% on room air. Physical examination shows no abnormalities. Endoscopy is most likely to show ulceration of which of the following structures?

(A) Duodenum
(B) Esophagus
(C) Gastric cardia
(D) Gastric fundus
(E) Pylorus

20. An 11-year-old boy is brought to the office by his parents for a well-child examination and routine vaccinations. His medical history is unremarkable and he receives no medications. Vaccinations were up-to-date at his most recent examination 1 year ago. He is not sexually active, and the family is affiliated with a religion that teaches abstinence. The parents express reluctance about administering the human papillomavirus (HPV) vaccine today because of the potential adverse effects associated with the vaccine. They ask if their son really needs this vaccine today, given that he will not become sexually active until he is much older and married. The patient's vital signs are within normal limits. Physical examination shows no abnormalities. Which of the following is the most appropriate response to the parents?

(A) Acknowledge that there are risks associated with vaccines but emphasize that those associated with the HPV vaccine are small because it is an inactivated vaccine
(B) Advise the parents to vaccinate their son in order to contribute to the elimination of HPV from the general population
(C) Explain that even if their son waits until marriage to have sex, he could still be exposed to HPV by his future partner
(D) Provide the parents with literature about the vaccine and advise them that they may defer the decision because the vaccine may be administered until the age of 26 years

The abstract displayed on these following pages is for use with items #21-23 on page 26.

Question

In patients with cirrhosis and acute bleeding esophageal varices, how do endoscopic sclerotherapy and emergency portacaval shunt compare for control of bleeding and survival?

Methods

Design: Randomized controlled trial (San Diego Bleeding Esophageal Varices Study). ClinicalTrials.gov NCT00690027.

Allocation: Concealed.

Blinding: Blinded (gastroenterologist who evaluated patients for portal-systemic encephalopathy).

Follow-up period: Up to 17 years.

Setting: University of California San Diego Medical Center.

Patients: 211 patients (mean age 49 years, 77% men) with acute bleeding esophageal varices resulting from cirrhosis, who required a transfusion of ≥ 2 units of blood and, for patients transferred from other hospitals, observation of upper gastrointestinal bleeding within 48 hours of transfer. Exclusion criterion was > 1 previous session of endoscopic sclerotherapy.

Intervention: Endoscopic sclerotherapy (n = 106) or emergency portacaval shunt (n = 105). Emergency portacaval shunt comprised a direct side-to-side or direct end-to-side portacaval shunt done within 8 hours of initial contact.

Outcomes: Control of bleeding at > 30 days, survival, readmissions for variceal or nonvariceal bleeding requiring transfusion of packed red blood cells, and recurrent portal-systemic encephalopathy.

Patient follow-up: 100% (minimum follow-up until death or 9.4 years).

Main results

15-year survival was lower with endoscopic sclerotherapy than with emergency portacaval shunt (10/106 vs 48/105, relative benefit reduction 79%, 95% CI 62 to 89; number needed to harm 3, CI 2 to 4). Other main results are shown in the Table.

Endoscopic sclerotherapy (EST) vs emergency portacaval shunt (EPCS) in patients with cirrhosis and acute bleeding esophageal varices

**Outcomes**	**Child risk class**	**EST**	**EPCS**	***P* value**
Control of bleeding at > 30 days*		20%	100%	<.001
Median survival (years)	A	4.62	10.43	.003
	B	2.61	6.19	<.001
	C	0.58	5.30	.005
Mean number of readmissions for variceal bleeding requiring packed red blood cell transfusion		6.8	0.4	<.001
Recurrent portal-systemic encephalopathy†		35%	15%	.001

*Excluding indeterminate deaths at 14 days from nonbleeding causes.

†In patients who survived 30 days and left hospital.

Conclusion

In patients with cirrhosis and acute bleeding esophageal varices, emergency portacaval shunt was better than endoscopic sclerotherapy for control of bleeding, recurrent encephalopathy, and survival.

Sources of funding: National Institutes of Health and Surgical Education and Research Foundation.

Structured abstract based on: Orloff MJ, Isenberg JI, Wheeler HO, et al. **Randomized trial of emergency endoscopic sclerotherapy versus emergency portacaval shunt for acutely bleeding esophageal varices in cirrhosis.** J Am Coll Surg. 2009;209:25-40. 19651060

Items #21–23 refer to the abstract displayed on the previous pages.

21. A 52-year-old man with hepatic cirrhosis comes to the emergency department because of a 3-hour history of vomiting blood. Esophagogastroduodenoscopy confirms actively bleeding esophageal varices. Based on the abstract shown, the physician is considering an emergency portacaval shunt (EPCS) procedure rather than endoscopic sclerotherapy (EST). According to the results in the abstract, approximately how many patients must be treated with EPCS rather than EST to prevent one case of recurrent portal-systemic encephalopathy?

(A) 1
(B) 3
(C) 5
(D) 10
(E) 16

22. Which of the following most strongly limits the generalizability of this study's findings?

(A) The allocation was concealed
(B) EPCS is available only at specialty centers
(C) The follow-up period was too short
(D) The patients were not blinded
(E) Unmeasured confounders were not controlled by the study design

23. Which of the following conclusions is most appropriate based on the results presented in the table?

(A) The 95% confidence interval for the difference in survival between EPCS and EST for Child-Pugh class A patients includes 0 years
(B) EPCS is more effective than EST in decreasing hospital readmissions for variceal bleeding requiring transfusion
(C) The median survival after EPCS is statistically significantly less for Child-Pugh class C than for Child-Pugh class B
(D) The randomization procedure was ineffective in decreasing bias in this study

END OF SET

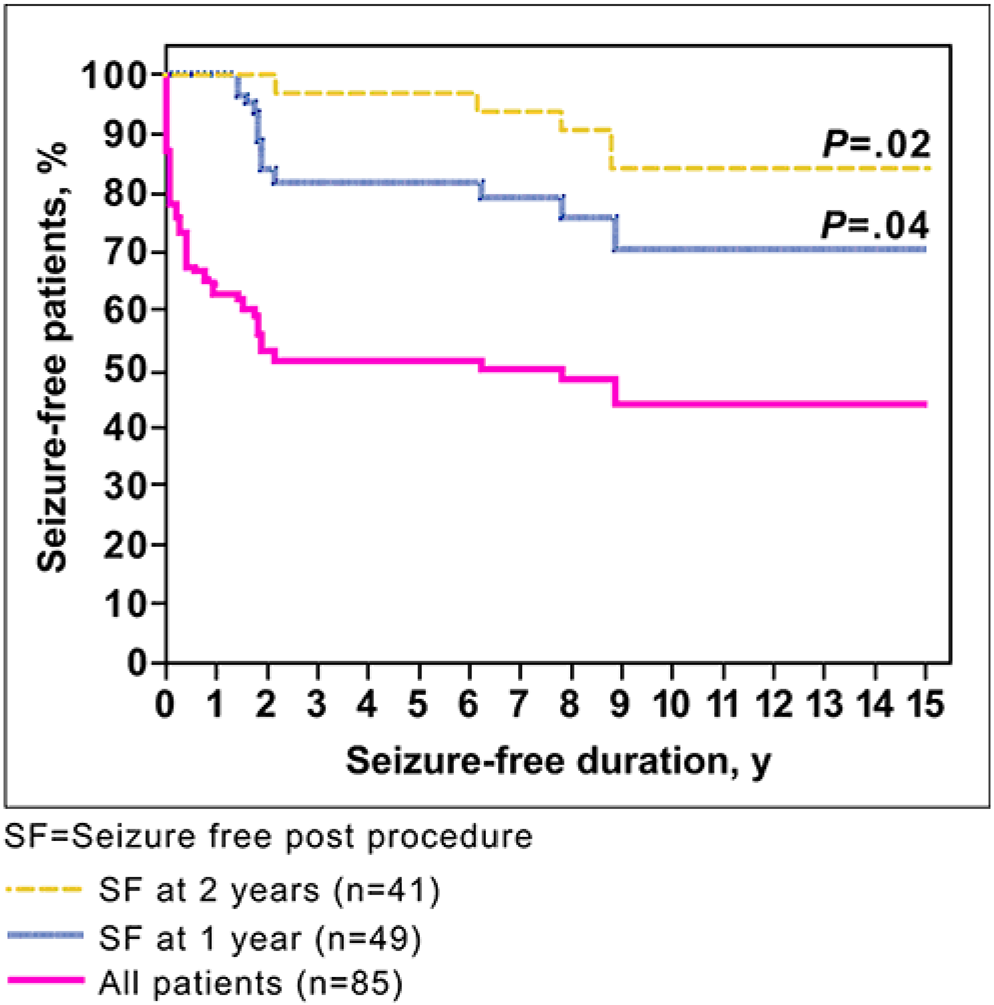

24. An 18-year-old woman with a 5-year history of epilepsy comes to the office with her parents for a follow-up examination. She has sustained three seizures during the past 6 months; all of her seizures originate from the temporal lobe. Anticonvulsant therapy has not decreased the frequency of her seizures. The patient and her parents ask about the potential benefits of partial temporal lobectomy. The physician performs a literature search and finds a published study conducted by a large surgical center evaluating outcomes of the operation. A total of 85 patients who underwent partial temporal lobectomy during a 3-year period were observed for 15 years to determine the number and frequency of seizures sustained postoperatively. Subgroup analyses were conducted of patients who reported no seizures 1 and 2 years postoperatively. A Kaplan-Meier plot of long-term outcomes is shown; the *P*-values provided represent subgroups compared with all other study patients. Which of the following is the most appropriate conclusion to draw from these data regarding seizure recurrence after the procedure?

(A) The absence of seizures 2 years postoperatively is the best predictor of long-term seizure control
(B) The majority of seizure recurrence occurred between 2 and 3 years postoperatively
(C) 30% of patients were seizure free 6 months after the procedure
(D) No conclusion can be determined because the subgroup analysis lacks statistical significance

25. A 2-week-old newborn with trisomy 18 diagnosed at birth is brought to the office by his parents for a follow-up visit. He is unable to suck properly and receives all his nutrition via nasogastric tube. The newborn was born at 37 weeks' gestation to a 29-year-old woman, gravida 2, para 1. Apgar scores were 4 at 1 minute and 8 at 5 minutes. The father is age 30 years. They have a 4-year-old daughter who is healthy. The mother reports that her son has trouble breathing and often stops breathing for up to 20 seconds at a time. He has had no fever, vomiting, or diarrhea. Length is 46 cm (18 in; 5th percentile), weight is 2400 g (5 lb 5 oz; 5th percentile), and head circumference is 32 cm (13 in; 10th percentile). Physical examination shows dysmorphic features with a small jaw and small head, as noted at birth. The patient is listless and his breaths are shallow. During the examination, he has episodes of apnea lasting up to 15 seconds. His tone is increased and he has overlapping fingers and rocker bottom feet. There is a 3/6 systolic murmur heard loudest over the left sternal border. Which of the following is the most appropriate next step?

(A) Admission to the hospital for a sleep apnea study
(B) CT scan of the head
(C) Electroencephalography
(D) Referral to a cardiologist
(E) Referral to home hospice care

26. A 79-year-old retired executive schedules an urgent office visit. When you see him, he says that he had difficulty walking upon getting out of bed this morning because of dizziness, which started last week and worsened last night. He has a 20-year history of hypertension, for which he was treated, but for the past 10 years his blood pressure has been normal without treatment. At his most recent visit 3 weeks ago, his blood pressure was 150/90 mm Hg and he was instructed to adhere to a low-salt diet. He drank a moderate amount of alcoholic beverages in the past and smoked about 16 cigarettes daily from age 20 years to age 50 years. He has not smoked cigarettes during the past 29 years. He is taking no medication at present. All his close relatives have lived to be at least in their 80s. This morning, pulse is 78/min and regular and blood pressure is 160/90 mm Hg. Neurologic examination shows instability when the patient is turning and horizontal nystagmus with change in position. Funduscopic examination shows no abnormalities. Which of the following is the most likely diagnosis?

(A) Cardiac arrhythmia
(B) Cerebellar ataxia due to alcohol abuse
(C) Labyrinthitis
(D) Orthostatic hypotension
(E) Stroke

27. A 78-year-old woman who resides in a nursing care facility has had increasing confusion and drowsiness during the past 10 days. The head nurse says, "The patient is usually alert, talkative, and outgoing, but during the past several days she has become increasingly confused and withdrawn. She drifts off to sleep even during activities that she enjoys." Vital signs have remained normal, and she has been eating well. There has been no change in her medication regimen, which includes pravastatin for hypercholesterolemia, amlodipine for hypertension, a daily 81-mg aspirin, and periodic sublingual nitroglycerin for stable angina pectoris. On interview, the patient says she does not have any symptoms. The patient identifies as White. Temperature is 37.0°C (98.6°F), pulse is 80/min, respirations are 16/min, and blood pressure is 117/84 mm Hg. The patient appears to be drifting in and out of sleep during the physical examination, but she is arousable and not in distress. She is oriented only to person and place. There is a 5-cm ecchymosis without hematoma on the right parietal scalp. Pupils are equal, round, and reactive to light and accommodation. Ocular movements are intact. Optic discs are poorly visualized due to bilateral cataracts. The remainder of the physical examination, including neurologic examination, shows no abnormalities. Results of laboratory studies are shown:

Serum		Blood	
Urea nitrogen	15 mg/dL	Hemoglobin	13.0 g/dL
Creatinine	1.0 mg/dL	WBC	8000/mm^3
Na^+	136 mEq/L	Platelet count	160,000/mm^3
K^+	3.9 mEq/L	Urine	
Cl^-	95 mEq/L	Specific gravity	1.015 (N=1.003–1.029)
HCO_3^-	25 mEq/L	pH	6.5 (N=4.5–7.8)
Glucose	98 mg/dL	Protein	Negative
		Glucose	Negative
		Occult blood	Negative
		Leukocyte esterase	Negative
		Nitrite	Negative

Which of the following is the most likely explanation for the patient's worsening condition?

(A) Drug-drug interaction
(B) Head trauma
(C) Hypothyroidism
(D) Major depressive episode
(E) Viral encephalitis

28. A 12-year-old girl with a seizure disorder is brought to the emergency department 40 minutes after her aunt found her unconscious in her backyard. The aunt reports that the patient is visiting her for the summer. On arrival, the patient is awake but drowsy. She says she is embarrassed because she forgot to bring her anticonvulsant medication from home. She requests that you not call her mother "because she will yell and get angry." Temperature is 37.0°C (98.6°F), pulse is 74/min, respirations are 16/min, and blood pressure is 100/70 mm Hg. Physical examination shows no focal neurologic deficits. The patient requests that she receive one dose of carbamazepine now and that she be given a prescription to fill at the nearby pharmacy. Which of the following is the most appropriate next step?

(A) Administer the dose of carbamazepine and provide a prescription
(B) Administer the dose of carbamazepine but do not provide a prescription
(C) Attempt to contact the mother for permission to treat before proceeding
(D) Do not contact the mother but request that the aunt sign a consent form
(E) Provide a prescription, but do not administer the dose of carbamazepine

29. A 63-year-old man comes to the office because of a 10-minute episode of right eye blindness 2 days ago. The episode resolved spontaneously and there was no associated eye pain. Medical history is remarkable for hypertension and type 2 diabetes mellitus. Medications include hydrochlorothiazide, metoprolol, glipizide, simvastatin, and 81-mg aspirin. He is 183 cm (6 ft) tall and weighs 104 kg (229 lb); BMI is 31 kg/m^2. Vital signs are within normal limits. Physical examination discloses pupils that are equal in size and reactive to light. Visual fields are intact. Visual acuity is 20/20 in both eyes with corrective lenses. Cardiac examination discloses a regular rate and rhythm with no murmurs. Neurologic examination discloses no abnormalities. Which of the following is the most appropriate diagnostic study at this time?

(A) Fluorescein angiography of the right eye
(B) Measurement of intraocular pressures
(C) Transesophageal echocardiography
(D) Ultrasonography of the neck
(E) No further evaluation is indicated

30. A 55-year-old man comes to the office because of a 4- to 6-week history of what he describes as "chest pounding" that worsens when he lies on his back. He also reports occasional episodes of nonexertional chest pain lasting 1 to 2 minutes. He is unable to further characterize the pain. He says that he has had moderate shortness of breath after walking three blocks, but he has not had nocturnal dyspnea. Medical history is remarkable for type 2 diabetes mellitus and gastroesophageal reflux disease. Current medications include simvastatin, metformin, esomeprazole, and 81-mg aspirin. The patient has smoked one pack of cigarettes daily for the past 35 years. Family history is remarkable for myocardial infarction in his father at age 58 years. The patient is 183 cm (6 ft) tall and weighs 91 kg (200 lb); BMI is 27 kg/m^2. Temperature is 36.7°C (98.1°F), pulse is 88/min, respirations are 16/min, and blood pressure is 165/55 mm Hg. There is no jugular venous distention. Carotid pulses are brisk and bounding. Auscultation of the lungs discloses mild, diffuse wheezes. Cardiac examination discloses a point of maximal impulse that is displaced laterally. There is a soft S_1 and S_2; a grade 3/6 blowing murmur is audible at the lower left sternal border that extends through two-thirds of diastole. Systolic and diastolic bruits are heard over both femoral arteries. Results of laboratory studies are shown:

Serum		Blood	
Cholesterol		Hemoglobin	13.0 g/dL
Total	300 mg/dL	WBC	10,500/mm^3
HDL	30 mg/dL		
LDL	180 mg/dL		
Triglycerides	190 mg/dL		
Urea nitrogen	28 mg/dL		
Creatinine	1.4 mg/dL		

ECG shows normal sinus rhythm and left ventricular hypertrophy. Chest x-ray shows an enlarged cardiac silhouette and prominent ascending aorta. Which of the following is the most appropriate additional diagnostic study at this time?

(A) Arterial brachial index of the lower extremity
(B) Coronary angiography
(C) Pulmonary function testing
(D) Renal ultrasonography
(E) Transthoracic echocardiography

31. A 39-year-old woman comes to the office because of a 1-year history of progressively worsening shortness of breath. She had been generally healthy and says that initially the shortness of breath occurred only after strenuous exercise; however, she now becomes fatigued and short of breath after walking two to three blocks. During the past 2 weeks, she has noted a rapid heart rate and light-headedness. She has not had chest pain, lower extremity edema, orthopnea, or any nocturnal symptoms. Medical history is remarkable for Graves disease, which was diagnosed 4 years ago and treated with radioactive iodine. Current medications include levothyroxine, a daily multivitamin, and an oral contraceptive. She does not smoke cigarettes or drink alcoholic beverages. She is 168 cm (5 ft 6 in) tall and weighs 57 kg (125 lb); BMI is 20 kg/m^2. Temperature is 37.0°C (98.6°F), pulse is 120/min and irregular, respirations are 16/min, and blood pressure is 110/85 mm Hg. Lungs are clear to auscultation. Cardiac examination discloses a rapid rate, a palpable parasternal lift, a normal S_1, and a widely split, prominent S_2. There is a grade 2/6 systolic murmur heard best over the second intercostal space. Examination of the lower extremities discloses trace edema of both feet. The remainder of the physical examination discloses no abnormalities. Results of laboratory studies are shown:

Serum		Blood	
Na^+	140 mEq/L	Hemoglobin	13.0 g/dL
K^+	3.4 mEq/L	Platelet count	155,000/mm^3
Cl^-	100 mEq/L		
HCO_3^-	25 mEq/L		
Glucose	130 mg/dL		
TSH	0.58 μU/mL		

ECG shows atrial fibrillation with a ventricular rate of 140/min and right axis deviation. Chest x-ray shows clear lung fields, no effusion, and prominent pulmonary arteries. Which of the following is the most appropriate additional diagnostic study?

(A) CT scan of the chest
(B) Echocardiography
(C) Perfusion lung scan
(D) Pulmonary function testing
(E) Ultrasonography of the thyroid

32. A 53-year-old man is admitted to the hospital for treatment of acute pancreatitis. Temperature is 37.8°C (100.0°F), pulse is 90/min, respirations are 16/min, and blood pressure is 160/90 mm Hg. Oxygen saturation is 94% on room air. There is tenderness and voluntary guarding in the epigastrium with hypoactive bowel sounds. Physical examination otherwise shows no abnormalities. Pneumatic compression devices are in place on both lower extremities. Results of laboratory studies show a leukocyte count of 18,700/mm^3, serum amylase concentration of 540 U/L, and serum lipase concentration of 500 U/L (N<200). Orders are written that the patient is to be given nothing by mouth; intravenous fluids are administered and he is given fentanyl intravenously for pain. On the third day in the hospital he is noted to be diaphoretic with labored breathing. Temperature now is 38.2°C (100.8°F), pulse is 95/min, respirations are 30/min, and blood pressure is 110/70 mm Hg. There are decreased breath sounds at the left lung base. Abdominal examination is unchanged. Which of the following is the most appropriate next step in the evaluation of these new findings?

(A) Chest x-ray
(B) Determination of cardiac enzyme activity
(C) Echocardiography
(D) Ultrasonography of the chest
(E) Ventilation-perfusion lung scans

33. A 24-year-old woman comes to the office because of a 3-month history of intermittent episodes of irritability and insomnia. She says, "I'll be fine for 2 to 3 weeks, but then I get so sensitive to anything my husband says. My mood will last for a few days, then I'm fine again." In addition to insomnia, she has associated headache, fatigue, and overeating during the same period. The patient married 3 months ago, and she says her husband has become frustrated with the unpredictability of her moods. She works as a phlebotomist. Medical history is unremarkable and she takes no prescribed medications. She does not smoke cigarettes or use other substances. She drinks one to two alcoholic beverages weekly. Her last menstrual period was 1 week ago. BMI is 20 kg/m^2. Vital signs are within normal limits and physical examination discloses no abnormalities. At this time, specific additional history should be obtained regarding which of the following?

(A) Carbohydrate intake
(B) Coital frequency
(C) Frequency of physical activity
(D) History of psychological trauma
(E) Stress level
(F) Timing of symptoms

34. A 55-year-old businessman comes to the office for a preemployment examination. The patient's last visit to a physician's office was 10 years ago. Examination at that time disclosed no abnormalities. Medical history is unremarkable, and the patient takes no medications. His father died of coronary artery disease at age 66 years. His 80-year-old mother was once treated for "some type of thyroid disease." The patient has a sedentary lifestyle and says, "My energy level isn't what it used to be." He frequently eats at fast-food restaurants. BMI is 30 kg/m^2. Temperature is 37.0°C (98.6°F), pulse is 82/min, respirations are 14/min, and blood pressure is 142/92 mm Hg. Physical examination discloses obesity but no other abnormalities. Results of fasting laboratory studies are shown:

Serum		Blood	
Cholesterol		Hematocrit	42%
Total	200 mg/dL	WBC	10,500/mm^3
HDL	34 mg/dL		
LDL	160 mg/dL		
Triglycerides	249 mg/dL		
Creatinine	1.1 mg/dL		
Glucose	126 mg/dL		

Results of serum liver function tests are within the reference ranges. Chest x-ray and ECG disclose no abnormalities. Determination of which of the following is the most appropriate next step in evaluation?

(A) Arterial blood gas values
(B) Hemoglobin A_{1c}
(C) 3-Hour glucose tolerance test
(D) Serum cortisol concentration
(E) Serum fructosamine concentration

35. An 8-year-old girl is brought to the emergency department via ambulance 1 hour after the onset of a generalized tonic-clonic seizure. Paramedics noted a pulse of 50/min followed by a 30-minute period of asystole. En route, the patient was intubated and oxygen therapy and an epinephrine inotropic drip were initiated. On arrival, the patient is unconscious. Temperature is 36.7°C (98.0°F), pulse is 40/min, respirations are 16/min, and blood pressure cannot be obtained. Oxygen saturation is 99% on an FIO_2 of 1.0. Physical examination shows mildly dysmorphic facies. Pupils are sluggish in response to light. Lungs are clear to auscultation with ventilated sounds. Cardiac examination discloses a regular rhythm and sinus bradycardia. Abdomen is soft and nontender. Neurologic examination discloses paralysis. Distal pulses are 1+ and weak. Echocardiography shows global hypokinesis with lateral inferior wall and septal akinesis. Left ventricular function is 20%. The patient is admitted to the hospital. Four days later, there has been no improvement in her condition. Life support is withdrawn, and the patient dies. At autopsy, examination of the heart shows evidence of an acute myocardial infarction. Which of the following is the most likely underlying cause of this patient's death?

(A) Cardiac conduction abnormality
(B) Coronary artery plaque rupture
(C) Increased pulmonary vascular resistance
(D) Systolic prolapse of mitral valve
(E) Thickened left ventricular wall and contractile dysfunction

36. A 2-month-old boy is brought to the office because of a 3-day history of yellow-tinged eyes and skin. He was delivered at 41 weeks' gestation. Pregnancy and delivery were uncomplicated. At delivery, length was 54 cm (21.3 in; 90th percentile), weight was 4200 g (9 lb 4 oz; 90th percentile), and head circumference was 37 cm (14.6 in; 90th percentile). Apgar scores were 9 and 10 at 1 and 5 minutes, respectively. He has been formula-fed and feeds every 3 to 4 hours without spitting up or emesis. He has pale bowel movements two to three times daily. He receives no medications. Current length is 60 cm (23.6 in; 75th percentile), weight is 5.7 kg (12 lb 9 oz; 65th percentile), and head circumference is 41 cm (16.1 in; 90th percentile). Temperature is 36.9°C (98.4°F), pulse is 130/min and regular, respirations are 40/min, and blood pressure is 105/65 mm Hg. Physical examination shows diffuse jaundice and conjunctival icterus. Cardiopulmonary examination discloses no abnormalities. The abdomen is soft, and there is mild hepatomegaly. Results of serum studies are shown:

ALT	265 U/L (N=12–45)
AST	280 U/L (N= 22–63)
Bilirubin, total	9.1 mg/dL (N<1.0)
Direct	6.6 mg/dL (N<0.3)
Protein, total	6.6 g/dL (N=4.6–7.9)
Albumin	4.5 g/dL (N=1.9–4.9)

Abdominal ultrasonography shows a hypoplastic gallbladder and small common bile duct. Which of the following changes is most likely occurring in this patient's liver at this time?

(A) Bile ductular proliferation
(B) Centrilobular necrosis
(C) Increased glycogen stores
(D) Intranuclear hepatocyte inclusions
(E) Macrovesicular steatosis

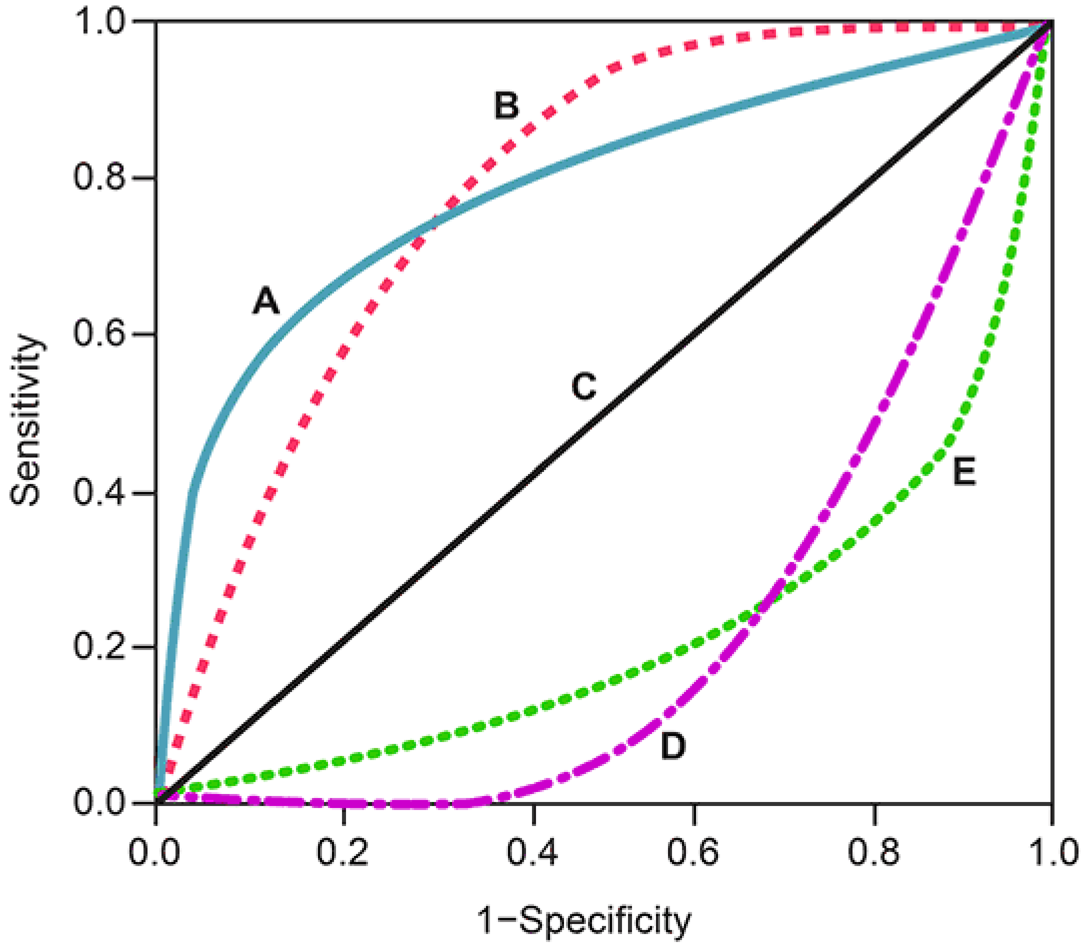

37. A 24-year-old primigravid woman comes to the clinic for her first prenatal visit. Her last menstrual period was 6 weeks ago. Medical history otherwise is unremarkable and her only medication is a prenatal vitamin. Family history is remarkable for gestational diabetes mellitus in her maternal aunt. The patient's vital signs are within normal limits. Physical examination discloses no abnormalities. The patient tells the physician that she would like to be screened for gestational diabetes mellitus and wants to make sure the test will not yield a false-negative result. Several screening tests are available and each test's performance is depicted in the graph shown. Based on these data, which of the following screening tests is the most appropriate choice for this patient?

(A) A
(B) B
(C) C
(D) D
(E) E
(F) Cannot be determined from the data provided

38. A 2-year-old girl is brought to the office by her father because of a 2-day history of a painful mass over the right side of her neck that has been increasing in size. The father says she cries when her neck is touched. She also has a 1-day history of a temperature of 38.1°C (100.6°F). The pain and fever have been well controlled with acetaminophen and ibuprofen. The patient has no history of serious illness and receives no other medications. Her maternal first cousin had non-Hodgkin lymphoma, and her paternal aunt had acute myelogenous leukemia. Examination and appropriate testing are done, and the patient is diagnosed with lymphadenitis. The physician informs the patient's father of the diagnosis and prescribes amoxicillin-clavulanate therapy. The patient's father questions the diagnosis and asks that his daughter be referred to a specialist. In addition to expressing empathy, which of the following is the most appropriate physician response?

(A) "I don't think she needs a specialist. What are you worried about?"
(B) "I know you're worried because of your family history of cancer, but I'm confident in the diagnosis and see no need to refer your daughter to a specialist."
(C) "Tell me what your greatest concerns are."
(D) "There's no need for your daughter to see a specialist. She'll feel better once she completes this course of antibiotics."
(E) "Why do you think that your daughter needs to see a specialist?"

NOTE: THIS IS THE END OF BLOCK 1.
ANY REMAINING TIME MAY BE USED TO CHECK ITEMS IN THIS BLOCK.

Block 2: FIP
Items 39–77; Time - 1 hour

ALL ITEMS REQUIRE SELECTION OF ONE BEST CHOICE.

39. A 68-year-old man is brought to the emergency department by his wife 2 hours after he collapsed into a chair and lost consciousness for 30 seconds. The wife says that during the 10 minutes prior to collapsing, the patient had nausea and two episodes of vomiting. The patient's medical history is significant for hypertension, hyperlipidemia, and coronary artery disease. Medications are metoprolol, 81-mg aspirin, and simvastatin. BMI is 28 kg/m^2. The patient is awake and fully oriented. Temperature is 37.3°C (99.2°F), pulse is 104/min, respirations are 24/min, and blood pressure is 163/94 mm Hg. Oxygen saturation is 95% on room air. Physical examination discloses drooping of the right side of the patient's face. Muscle strength is 3/5 in the left upper extremity and 4/5 in the left lower extremity; strength in the right extremities is normal. Gait is ataxic. The patient has difficulty swallowing when trying to drink a cup of water. This patient's symptoms are most consistent with injury to the brain in the area supplied by which of the following cerebral arteries?

(A) Anterior
(B) Internal carotid
(C) Middle
(D) Posterior
(E) Vertebrobasilar

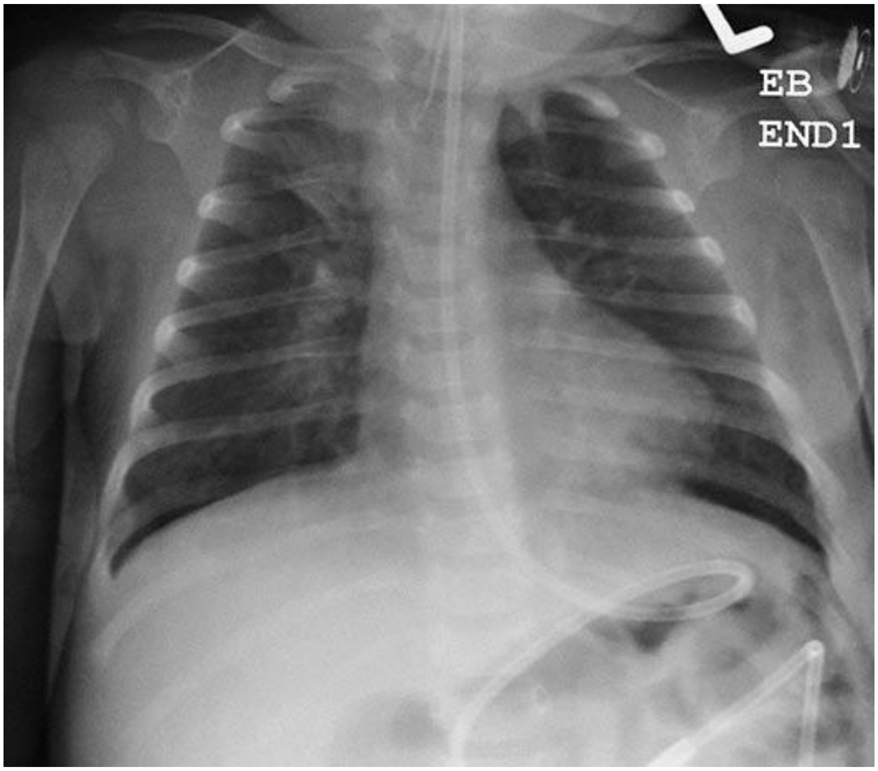

40. A 3-month-old female infant is admitted to the intensive care unit via the emergency department in winter because of respiratory distress. The infant first became ill 3 days ago with fever and congestion, but her symptoms have worsened since that time. This morning she developed pallor, lethargy, cough, copious respiratory secretions, and tachypnea. On arrival at the emergency department today, temperature was 37.1°C (98.8°F), pulse was 124/min, respirations were 80/min, and blood pressure was 80/35 mm Hg. Oxygen saturation was 78% on room air; after administration of supplemental oxygen, oxygen saturation increased to 94%. The infant's pallor has not subsided, and she is in obvious respiratory distress. Physical examination discloses head bobbing, nasal flaring, and subcostal retractions with each breath. Auscultation of the lungs discloses crepitant crackles throughout both lung fields with scattered expiratory wheezing. The remainder of the physical examination discloses no abnormalities. The infant is prepared for intubation based on her clinical status. Results of laboratory studies are shown:

Capillary blood gas analysis on oxygen at 2 L/min	
P_{O_2}	55 mm Hg (N>90)
P_{CO_2}	64 mm Hg (N=26.4–41.2)
pH	7.22 (N=7.35–7.45)
HCO_3^-	26 mEq/L

Blood	
Hematocrit	30%
Hemoglobin	10.1 g/dL
WBC	9800/mm^3
Neutrophils, segmented	18%
Lymphocytes	80%
Platelet count	220,000/mm^3

Chest x-ray is shown. Which of the following is the most likely cause of this infant's illness?

(A) *Haemophilus influenzae*
(B) Herpes virus
(C) *Mycoplasma pneumoniae*
(D) Respiratory syncytial virus
(E) *Streptococcus pneumoniae*

41. A 44-year-old woman, who is a known patient, comes to the office for a Pap smear. She says she has felt fine but has noted a significant increase in the amount of bleeding with her menstrual periods. She says, "My periods are still regular but they now last 10 days instead of 6. It seems the flow is very heavy for at least 5 of the 10 days. With periods like this, I am anxious for menopause." She also reports frequent fatigue and occasional insomnia, but she attributes these symptoms to having three teenaged children. She has not had dysmenorrhea, dyspareunia, or vaginal discharge. Medical history is unremarkable. She does not use any medications and says that she does not like to take pills. Family history is positive for colonic polyps. The patient has been separated from her husband for 5 years. She is occasionally sexually active and always uses condoms. Pelvic examination today discloses no abnormalities except for an enlarged uterus, approximately 8 weeks' gestational size, which is unchanged from her previous examination. Ultrasonography 2 years ago disclosed a myomatous uterus. Cervical cytology and appropriate cultures are obtained, and complete blood count and serum lipid profile are ordered. Which of the following is the most appropriate diagnostic study at this time?

(A) Colposcopy
(B) Determination of serum follicle-stimulating hormone and luteinizing hormone concentrations
(C) Determination of serum prolactin concentration
(D) Endometrial biopsy
(E) Hysteroscopy

42. A 58-year-old man, who is recovering in the hospital 2 days after an uncomplicated elective right total knee arthroplasty, suddenly develops left-sided chest pressure without radiation. The patient was able to ambulate with assistance earlier today. Medical history is significant for hypertension, type 2 diabetes mellitus, dyslipidemia, and osteoarthritis. His routine medications are enalapril, metoprolol, metformin, and rosuvastatin; since admission his routine medications have been continued except for metformin, which has been replaced with sliding-scale insulin. Enoxaparin was added for deep venous thrombosis prophylaxis. The patient is diaphoretic. Temperature is 37.0°C (98.6°F), pulse is 100/min, respirations are 20/min, and blood pressure is 90/60 mm Hg. Oxygen saturation is 92% on room air. Jugular venous pressure is 15 cm H_2O. Lungs are clear to auscultation. Cardiac examination discloses an S_3 and a grade 2/6 murmur heard best at the right second intercostal space with no radiation. ECG shows sinus tachycardia with 2-mm ST-segment elevations in leads II, III, aVF, V_1, and V_2. Intravenous heparin, 325-mg aspirin, and sublingual nitroglycerin are administered, after which the patient's systolic blood pressure immediately decreases to 72 mm Hg. Which of the following is the most likely cause of this patient's symptoms?

(A) Acute mitral regurgitation
(B) Pericardial tamponade
(C) Pulmonary embolism
(D) Right ventricular infarction
(E) Vasovagal reaction

43. A study is conducted to assess physician use of β-adrenergic blocking agents as treatment for heart failure. A random sample of 5000 practicing physicians is selected and surveyed using a 10-item validated questionnaire. Researchers stratify the current prescribing patterns by age of physician, and by location and type of practice. Which of the following most accurately characterizes this study design?

(A) Case-control study
(B) Case series
(C) Clinical trial
(D) Cross-sectional study
(E) Prospective cohort study
(F) Retrospective cohort study

44. A 28-year-old man comes to the office because of a 3-day history of increasing pain on urination. He says, "I'm worried that I might have a sexual disease." He has been married for the past 7 years, and he states that his only sexual relations since then have been with his wife. Medical history is unremarkable and the patient takes no medications. He works at a gardening center. Vital signs are within normal limits. Abdominal examination discloses shotty bilateral inguinal lymphadenopathy. Examination of the penis discloses a minimal amount of watery urethral discharge. The testes appear normal. The remainder of the physical examination shows no abnormalities. Which of the following is the most appropriate diagnostic study at this time?

(A) Gram stain of urethral discharge before and after prostatic massage
(B) Polymerase chain reaction test for *Neisseria gonorrhoeae* and *Chlamydia trachomatis*
(C) Urinalysis
(D) Urine culture and sensitivity
(E) No study, pending reevaluation of sexual history

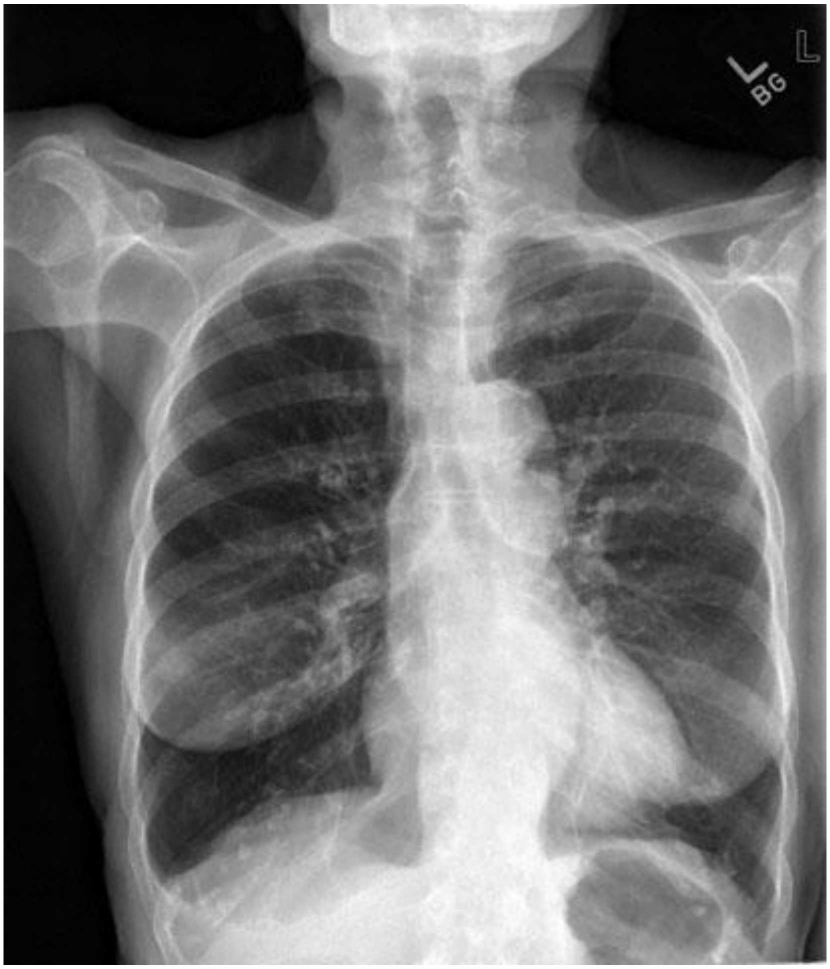

45. A 72-year-old woman comes to the office because of a 1-year history of worsening shortness of breath and occasional chest tightness and nonproductive cough. Her symptoms have varied in intensity but she has had several periods of worsening symptoms during the past 6 months. Medical history is unremarkable and she takes no medications. She has smoked 1½ packs of cigarettes daily for the past 50 years; she does not drink alcoholic beverages. She works as a florist. BMI is 22 kg/m^2. Vital signs are within normal limits. The patient is not in respiratory distress. Auscultation of the lungs discloses a prolonged expiratory phase. Cardiac examination discloses no abnormalities. There are no rashes or lesions. Results of spirometry done in the office are shown:

	**Baseline**	**Post Bronchodilator**
FEV_1	60% of predicted	65% of predicted
FVC	82% of predicted	82% of predicted
FEV_1/FVC ratio	0.55	0.62

Chest x-ray is shown. Which of the following is the most likely underlying cause of this patient's condition?

(A) Bacterial colonization of large- and medium-sized airways
(B) Bronchial constriction in response to environmental allergens
(C) Destruction of alveolar walls leading to enlargement of air spaces
(D) Diffuse patchy interstitial inflammation and fibrosis
(E) Eosinophilic infiltration of the pulmonary interstitium

46. **Patient Information**
Age: 16 years
Gender: M, self-identified
Race/Ethnicity: unspecified
Site of Care: office

The patient is brought for a well-child visit. The patient's mother is concerned because his grades have declined from B's and C's to C's and D's during the past year. She says he has been having difficulty concentrating in school and asks the physician to write a prescription for methylphenidate. Medical history is unremarkable and the patient takes no medications. The mother has a history of major depressive disorder treated with sertraline. The patient's maternal grandfather, uncle, and cousin have alcohol use disorder. The physician asks the patient's mother to leave the examination room. When alone with the physician, the patient says he has smoked cannabis at least three times weekly during the past 6 months. He often smokes with friends in the morning before school starts. He does not drink alcoholic beverages or take prescription drugs or other substances. Physical examination shows no abnormalities. On mental status examination, he reports no depressive symptoms or thoughts of harming himself. Urine toxicology screening is positive for Δ^9-tetrahydrocannabinol. After the examination, the patient becomes distressed and says, "You cannot tell my mother about the marijuana. This is confidential." Which of the following is the most appropriate initial physician response?

(A) "I am worried about your cannabis use, so I recommend that you decrease your cannabis use and see me again in 4 weeks for a repeat urine test."
(B) "I can give you a telephone number for enrollment in a substance abuse treatment program."
(C) "Cannabis is bad for your brain, so let me tell you how 12-step programs can help you stop using cannabis."
(D) "Because you are not actually unsafe, this discussion is between the two of us."
(E) "We need to discuss how cannabis affects your functioning. How can I help you tell your mother?"

47. A 77-year-old man is admitted to the hospital because of a 2-week history of gangrene of the right foot. Three years ago, he underwent a left transmetatarsal amputation. During hospitalization, he required intubation and mechanical ventilation for 2 weeks because of sepsis. He then spent 2 months in an acute rehabilitation facility. Medical history also is remarkable for type 2 diabetes mellitus, peripheral vascular disease, and hypertension. His medications are insulin, irbesartan, hydrochlorothiazide, and aspirin. He is widowed, lives alone, and keeps in contact with his two sons. He has a living will, and his younger son is designated health care proxy. The patient is alert and fully oriented. Temperature is 38.4°C (101.1°F), pulse is 90/min and regular, respirations are 12/min, and blood pressure is 156/90 mm Hg. Oxygen saturation is 96% on room air. There is a 5 × 6-cm ulcer with black margins over the plantar surface of the right foot; malodorous, white-green exudate; and visible bone in the center of the ulcer. Popliteal fossa pulses are decreased and posterior tibial pulses are absent bilaterally. Antibiotic therapy is begun. The patient is informed that he requires amputation of the right leg below the knee. He declines amputation, saying, "I don't ever want to go through what I went through before with my left foot." He is told that the potential risks for not proceeding with amputation are loss of the entire right lower extremity and death. He still refuses the procedure. Which of the following is the most appropriate action for this physician to take?

(A) Abide by the patient's wishes
(B) Consult the hospital bioethics department
(C) Obtain consent for amputation from both sons
(D) Obtain consent for amputation from the courts
(E) Obtain consent for amputation from the younger son
(F) Review any additional instructions in the patient's living will

48. A 63-year-old woman is evaluated in the hospital 2 days after she was admitted for management of left sternal chest pain and a hypertensive emergency with a blood pressure of 195/110 mm Hg. Workup included CT angiography of the chest, which showed no evidence of pulmonary embolism. During the past 6 hours, she has had decreased urine output. A bladder catheter yielded only 50 mL of urine during the past 3 hours. She has not had abdominal pain. Medical history is remarkable for hypertension, osteoarthritis, and stage 3 chronic kidney disease with a baseline serum creatinine concentration of 1.7 mg/dL. Routine medications are lisinopril, amlodipine, and hydrochlorothiazide. The chest pain she had on admission has resolved. Temperature is 37.6°C (99.7°F), pulse is 87/min, respirations are 18/min, and blood pressure is 147/87 mm Hg. Physical examination discloses no abnormalities. Results of laboratory studies obtained on admission and today are shown:

	**On Admission**	**Today**
Serum		
Na^+	138 mEq/L	141 mEq/L
K^+	4.2 mEq/L	5.1 mEq/L
Cl^-	119 mEq/L	118 mEq/L
HCO_3^-	23 mEq/L	21 mEq/L
Urea nitrogen	26 mg/dL	26 mg/dL
Creatinine	1.6 mg/dL	2.4 mg/dL
Glucose	106 mg/dL	108 mg/dL
Urine		
Specific gravity	–	1.010 (N=1.003–1.029)
Protein	–	Negative
Blood	–	Negative
Leukocyte esterase	–	Negative
WBCs	–	0–3/hpf
RBCs	–	0–3/hpf
Casts	–	Muddy brown

Which of the following factors in this patient's history most increased her risk for developing her current complication?

(A) Bladder catheterization
(B) Chronic kidney disease
(C) Uncontrolled hypertension
(D) Use of lisinopril
(E) Volume depletion

49. A randomized placebo-controlled trial is conducted to assess the efficacy of duloxetine in patients with severe rheumatoid arthritis who are currently treated with methotrexate. Patients who have six or more joints that are actively inflamed, swollen, or tender to palpation are eligible for inclusion in the study. The primary outcome measure is the number of joints that are no longer inflamed after 4 months of treatment. One hundred fifty patients are enrolled and randomized to the duloxetine group (n=75) or the placebo group (n=75). The mean number of involved joints is 12. Data show that patients in the intervention group have a mean of seven joints that are no longer inflamed at the end of the trial; patients in the placebo group have a mean of two joints that are no longer inflamed at the end of the trial. Results of a two-tailed *t* test show P=.02. Which of the following is the most appropriate conclusion to draw regarding the clinical and statistical significance of this study?

	**Clinically Significant**	**Statistically Significant**
(A)	No	no
(B)	No	yes
(C)	Yes	no
(D)	Yes	yes

---

50. A physician would like to evaluate the benefit of a new osteoporosis medication in preventing fractures in women aged 40 to 60 years. A total of 300 women aged 40 to 60 years with osteoporosis documented by DEXA scan are enrolled in the study from general gynecology clinics. The patients are equally randomized into two groups. Group A receives the new osteoporosis medication and Group B receives placebo. The patients are followed for 5 years and bone density is measured annually by DEXA scanning. Results show that the bone density of patients who received the study medication is unchanged and the bone density in patients who received placebo decreased by 2% (P=.01). The physician concludes that the new medication decreases the risk for fracture in women aged 40 to 60 years. Which of the following is the most appropriate conclusion to draw from these data?

(A) The impact of the medication on bone density may not correlate with the risk for fracture
(B) The lack of a change in bone density among patients who received the study medication suggests the presence of a confounding variable
(C) The lack of a change in bone density among the treatment group suggests that the medication regimen is too complicated
(D) The lack of women younger than 40 years of age makes it difficult to evaluate the long-term benefit of treatment
(E) Selection bias is likely because the patients were all selected from general gynecology practices

51. A 15-year-old boy is brought to the clinic after his father noticed significant swelling of the boy’s chest when he was getting dressed 6 hours ago. The patient reports a 3-month history of gradually worsening left-sided chest pain and swelling, but he says he was too embarrassed to tell his parents. He has not had fever, chills, night sweats, loss of appetite, weight loss, or any recent trauma to the chest. Medical history is unremarkable and he takes no medications. The patient is a good student. He does not smoke cigarettes or drink alcoholic beverages. Temperature is 36.4°C (97.5°F), pulse is 69/min, respirations are 14/min, and blood pressure is 115/70 mm Hg. Oxygen saturation is 100% on room air. Examination of the head, neck, and skin discloses no abnormalities. There is a fixed palpable mass measuring approximately 14 × 12 cm in the left chest wall that is tender to palpation. The remainder of the physical examination discloses no abnormalities. Biopsy of this mass is most likely to show which of the following?

(A) Acid-fast bacilli
(B) Gram-positive cocci in clusters
(C) Infiltrating nests of squamous cells and increased keratin
(D) Malignant syncytiotrophoblasts and cytotrophoblasts
(E) Poorly differentiated anaplastic spindle cells

52. A 4-year-old boy is brought to the office by his mother because of a 2-kg (5-lb) weight loss, decreased appetite, and daily fatigue during the past 2 months. The mother says the patient's fatigue has limited his physical activity. The patient's medical history is remarkable for asthma and seasonal allergies. He has had three asthma exacerbations during the past 2 months that have required treatment with oral prednisone. Routine medications are cetirizine, albuterol, and budesonide. The patient is 99 cm (3 ft 3 in; 25th percentile) tall and weighs 14 kg (31 lb; 25th percentile); BMI is 14.3 kg/m^2 (10th percentile). Temperature is 36.7°C (98.0°F), pulse is 70/min, respirations are 18/min, and blood pressure is 80/50 mm Hg. The patient appears lethargic. Physical examination discloses no abnormalities. Results of laboratory studies are shown:

Serum		Blood	
Creatinine	0.8 mg/dL (N=0.03–0.59)	Hemoglobin	12.0 g/dL (N=11.5–14.5)
Na^+	125 mEq/L	WBC	10,000/mm^3
K^+	4.5 mEq/L	Platelet count	250,000/mm^3
Cl^-	90 mEq/L	Urine	
HCO_3^-	25 mEq/L	Specific gravity	1.025 (N=1.003–1.029)
Glucose	60 mg/dL (N=60–100)	Protein	Negative
		Blood	Negative
		Leukocyte esterase	Negative
		Nitrite	Negative

Which of the following is the most likely underlying cause of this patient's current condition?

(A) Decreased activity of 21-hydroxylase enzyme
(B) Decreased production of adrenocorticotropic hormone (ACTH)
(C) Decreased production of antidiuretic hormone
(D) Increased aldosterone production
(E) Increased insulin production

53. A 38-year-old woman is brought to the emergency department because of a 4-hour history of confusion and lethargy. Medical history is remarkable for major depressive disorder and a 6-month history of moderate low back pain that began after she lifted heavy boxes during a move. Her medications are fluoxetine daily and acetaminophen with codeine as needed. Family history is remarkable for hypertension and congestive heart failure in her mother. The patient lives with her husband and her mother. The patient drinks two glasses of wine on weekends. On arrival, she is somnolent but arousable to sternal rub. Temperature is 36.4°C (97.5°F), pulse is 32/min, respirations are 16/min, and blood pressure is 74/36 mm Hg. Oxygen saturation is 100% on room air. Pupils measure 6 mm in diameter. Examination shows no other abnormalities. Results of laboratory studies are shown:

Serum		Blood	
ALT	180 U/L	Hemoglobin	12 g/dL
AST	150 U/L	WBC	12,000/mm^3
Creatinine	2.5 mg/dL	Platelet count	400,000/mm^3
Acetaminophen	negative		
Urine toxicology			
Opiates	positive		

An overdose of a drug with which of the following mechanisms of action is the most likely cause of this patient’s condition?

(A) Agonism of μ-opioid receptors
(B) Inhibition of aldosterone
(C) Inhibition of angiotensin-converting enzyme
(D) Inhibition of β_1-adrenergic receptors
(E) Inhibition of central nervous system neuron serotonin reuptake
(F) Inhibition of prostaglandin synthesis
(G) Inhibition of renal sodium and chloride resorption

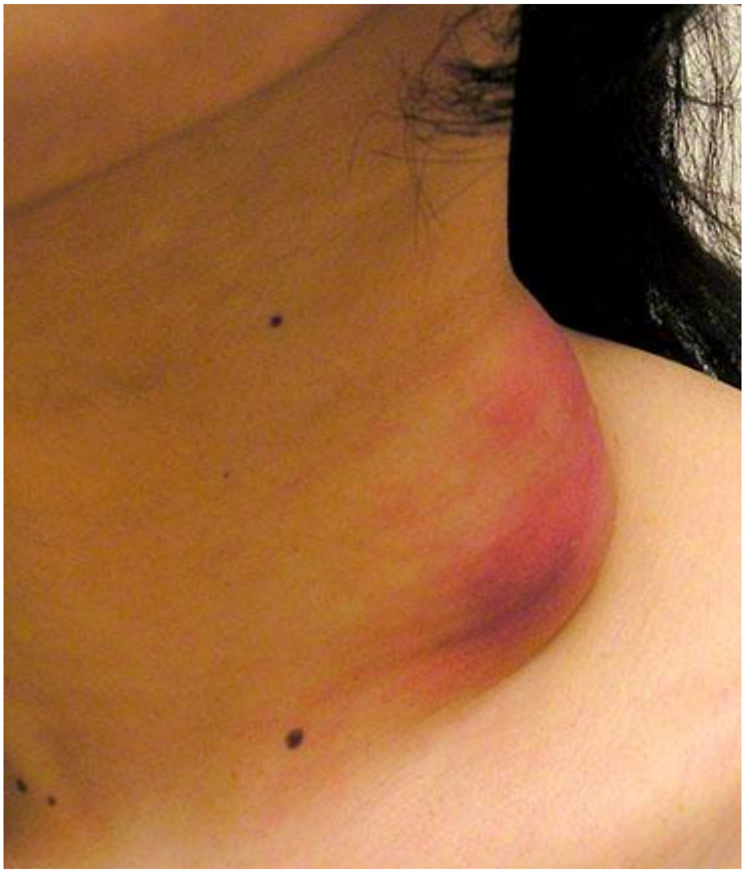

54. A 56-year-old woman comes to the office because of a 1-week history of fever and moderate pain and swelling of the left side of her neck. Medical history is remarkable for a 4-year history of HIV infection. She has declined antiretroviral therapy and takes no medications. BMI is 20 kg/m^2. Temperature is 38.3°C (100.9°F); other vital signs, including oxygen saturation on room air, are within normal limits. Physical examination discloses warmth and moderate tenderness over the swelling shown in the photograph. Lungs are clear to auscultation. There is no rash or hepatosplenomegaly. CD4+ T-lymphocyte count is 160/mm^3, and plasma HIV viral load is 56,000 copies/mL. Pathologic examination of a biopsy specimen of a cervical lymph node shows 2+ acid-fast bacilli, and result of a nucleic acid amplification test is positive for a drug-sensitive mycobacterium. The infection in this patient most likely reached the cervical lymph nodes through which of the following routes?

(A) Direct inoculation via the skin
(B) Direct tissue invasion from the hilar lymph nodes
(C) Hematogenous spread from the lungs
(D) Lymphatic drainage from pharyngeal tissues
(E) Trafficking of infected lymphocytes

55. **Patient Information**
Age: 64 years
Gender: F, self-identified
Ethnicity: unspecified
Site of Care: office

History
Reason for Visit/Chief Concern: "I can't catch my breath even when I'm sitting still."

History of Present Illness:
• shortness of breath at rest for 12 hours
Past Medical History:
• 15-year history of chronic obstructive pulmonary disease (COPD)
• inguinal hernia repair 1 week ago
Medications:
• albuterol
Allergies:
• no known drug allergies
Psychosocial History:
• does not smoke cigarettes or drink alcoholic beverages

Physical Examination

Temp	Pulse	Resp	BP	O_2 Sat	Ht	Wt	BMI
36.7°C (98.0°F)	110/min	24/min	140/80 mm Hg	86% on RA	168 cm (5 ft 6 in)	63 kg (140 lb)	23 kg/m^2

• Appearance: in acute distress; bilateral sternocleidomastoid muscles and intercostal muscles are retracted
• Neck: jugular venous pressure of 4 cm H_2O above the sternal angle
• Pulmonary: clear lung fields bilaterally
• Cardiac: tachycardia; S_1 and S_2, no murmurs, rubs, or gallops

Diagnostic Studies

Plasma	
Brain natriuretic peptide (BNP)	100 pg/mL (N<167)
D-dimer	25 µg/mL (N<0.5)

Blood	
Hematocrit	39%
Hemoglobin	13.0 g/dL
WBC	9000/mm^3
Platelet count	325,000/mm^3

• chest x-ray: no abnormalities

Question: Which of the following is the most likely diagnosis?

(A) COPD exacerbation
(B) Heart failure
(C) Pneumonia
(D) Pneumothorax
(E) Pulmonary embolism

56. A 20-year-old primigravid woman comes to the emergency department at 8 weeks' gestation because of a 2-day history of persistent nausea and vomiting with a 0.9-kg (2-lb) weight loss during this time. She also notes that she has not voided during the past 8 hours. She has not had fever, chills, or contact with anyone known to be ill. Medical history is unremarkable. She has taken prenatal vitamins sporadically during her pregnancy, but they worsen her nausea and vomiting. Temperature is 36.8°C (98.2°F), pulse is 98/min, respirations are 18/min, and blood pressure is 118/60 mm Hg. Oxygen saturation is 99% on room air. Physical examination discloses no abnormalities. Urine specific gravity is greater than 1.030 (N=1.003–1.029). Which of the following laboratory study results is most likely to be abnormal in this patient?

(A) Leukocyte count
(B) Serum ALT and AST activities
(C) Serum calcium concentration
(D) Serum glucose concentration
(E) Serum urea nitrogen concentration
(F) No abnormality is likely

57. A 60-year-old woman, who lives alone, is brought to the emergency department on a Friday evening by her next-door neighbor who says the patient has fallen several times during the past several days. An emergency department record from a visit 1 year ago for a minor laceration indicates that the patient has Parkinson disease with mild dementia, bipolar disorder, and hypertension. Her brother fills her weekly medication organizer, but he is overseas on a business trip for the week. The patient is unable to remember which medications she takes and whether there have been any changes recently to her medication regimen. The neighbor was unable to locate her pill organizer in her home, and the patient thinks her brother keeps her pill bottles at his house to prevent her from inadvertently taking extra doses. She has her brother's address on a card in her wallet, along with the names and phone numbers of her primary care physician, psychiatrist, and pharmacy. The medical record from her visit 1 year ago shows that her medications included clonazepam, clonidine, and lithium carbonate. Doses of each medication are also listed. Attempts to contact the patient's primary care physician and psychiatrist for more detailed medication history have been unsuccessful. Which of the following is the most appropriate initial approach to managing the patient's medications?

(A) Ask the police to enter the brother's house to locate the patient's pill bottles
(B) Contact the patient's pharmacy to obtain information on her current medications
(C) Observe the patient closely while continuing to try to reach her psychiatrist
(D) Order the medications as listed in the emergency department records from her previous visit
(E) Order serum concentrations of the medications listed on the previous emergency department record, then prescribe doses of the medications in accordance with those concentrations

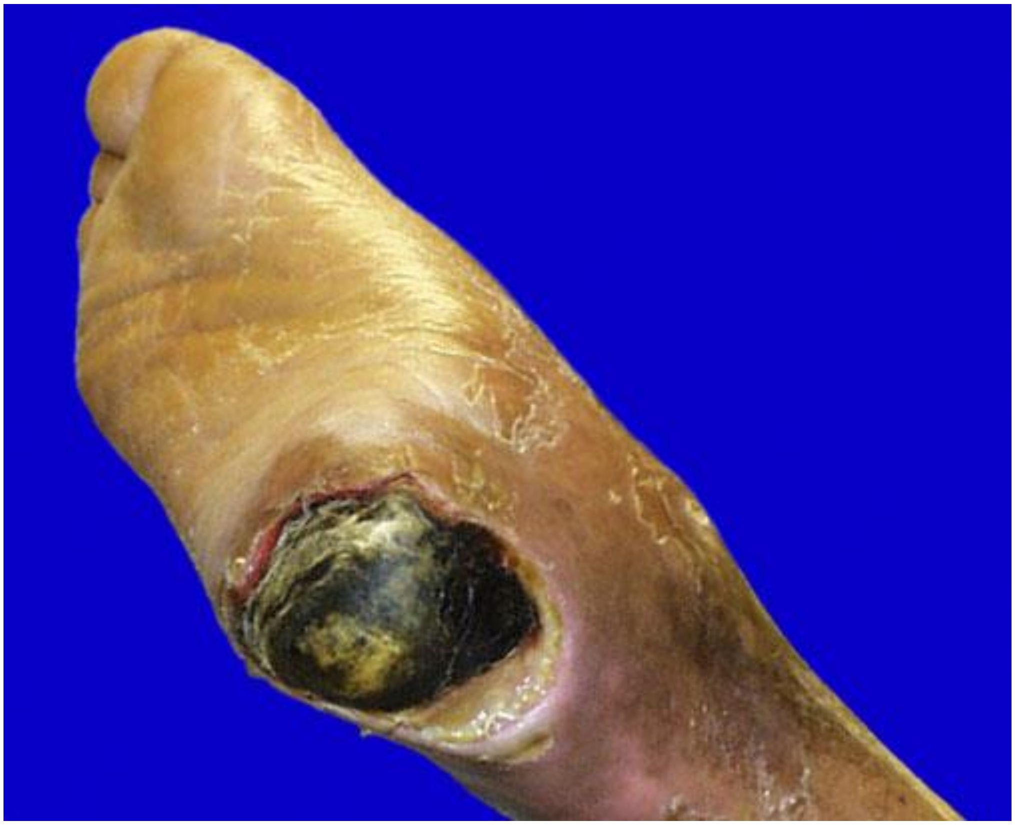

58. A 73-year-old man is admitted to a nursing care facility from the hospital following a prolonged hospitalization for pneumonia, septicemia, and respiratory failure. Medical history is remarkable for advanced dementia, Alzheimer type, hypertension, and type 2 diabetes mellitus. Current medications include lisinopril, enteric-coated aspirin, and insulin glargine. The patient appears frail and is confined to bed. He opens his eyes to voice but is noncommunicative. BMI is 27 kg/m^2. Temperature is 37.2°C (99.0°F), pulse is 66/min, respirations are 15/min, and blood pressure is 142/86 mm Hg. Oxygen saturation is 97% on room air. Physical examination discloses the finding on the patient's right foot as shown in the photograph. The remainder of the examination discloses no abnormalities. Which of the following is the most likely underlying cause of this patient's condition?

(A) Infection with methicillin-resistant *Staphylococcus aureus*
(B) Prolonged immobility
(C) Pyoderma gangrenosum
(D) Systemic atheroemboli
(E) Venous stasis secondary to valvular incompetence

59. A 78-year-old woman, who was admitted to the hospital 3 days ago because of worsening renal failure, has become increasingly lethargic and nonresponsive. Medical history is significant for type 2 diabetes mellitus, hypertension, and stroke 1 year ago with residual right-sided weakness and slurred speech. Medications include insulin, aspirin, and furosemide. On admission, the patient was alert with mildly slurred speech; she was oriented to person, place, and time. Physical examination disclosed decreased motor strength and muscle atrophy in the right upper and lower extremities. Results of laboratory studies showed a serum urea nitrogen concentration of 82 mg/dL and a creatinine concentration of 6.1 mg/dL. Hemodialysis was recommended but the patient declined and expressed an understanding that she would die without the intervention. In addition, she requested a do-not-attempt resuscitation (DNAR) order. This was documented in her medical record and her code status was changed to DNR. Serum urea nitrogen concentration now is 130 mg/dL. She has had no urine output during the past 48 hours. The patient's daughter arrives and states that she is named as the surrogate decision maker by her mother's advance directive. The daughter insists that hemodialysis be initiated and the code status be changed to full code. Which of the following is the most appropriate course of action?

(A) Continue current management; do not initiate hemodialysis
(B) Initiate hemodialysis and change the code status to full code until the patient is more alert
(C) Initiate hemodialysis but keep the code status as DNAR until the patient is more alert
(D) Obtain consent from the patient's daughter to initiate all medically indicated interventions

60. An 88-year-old woman is transferred to the emergency department from the nursing care facility where she resides 4 hours after facility staff noted that the patient had rectal bleeding. Medical history is significant for long-standing dementia secondary to multiple prior strokes, coronary artery disease, hypertension, hypercholesterolemia, and chronic renal disease. Medications include 81-mg aspirin, simvastatin, lisinopril, and hydrochlorothiazide. On arrival in the emergency department, temperature is 37.2°C (98.9°F), pulse is 102/min, respirations are 20/min, and blood pressure is 105/60 mm Hg. Oxygen saturation is 95% on room air. The patient is awake but is not oriented to person, place, or time. She is unable to speak coherently and becomes agitated and combative when a physical examination is attempted. Which of the following is the most appropriate initial intervention?

(A) Administer lorazepam and proceed with physical examination
(B) Contact the patient's health care proxy to discuss how to proceed
(C) Place the patient in soft wrist restraints and proceed with physical examination
(D) Schedule outpatient flexible sigmoidoscopy and discharge the patient

61. A 56-year-old man comes to the office because of a 3-week history of right arm weakness and drooping of his right eyelid. Medical history is significant for hypertension and dyslipidemia, but he takes no medications. He has smoked one-half pack of cigarettes daily for the past 40 years. BMI is 26 kg/m^2. Temperature is 37.2°C (99.0°F), pulse is 82/min, respirations are 20/min, and blood pressure is 138/74 mm Hg. Physical examination shows ptosis on the right side. The left pupil measures 5 mm in diameter and the right pupil measures 4 mm in diameter; both pupils are reactive to light and accommodation. Muscle strength is 3/5 in the right finger flexors and hand grip. Muscle strength and deep tendon reflexes are otherwise normal. Fingerstick blood glucose concentration is 119 mg/dL. Which of the following is the most likely diagnosis?

(A) Cerebellar artery aneurysm
(B) Diabetic oculomotor paresis
(C) Myasthenia gravis
(D) Myasthenic (Lambert-Eaton) syndrome
(E) Pancoast tumor

Items #62–63 are part of a sequential item set. In the actual examination environment, you will **not** be able to view the second item until you click "Proceed to Next Item." After navigating to the second item, you will not be able to add or change an answer to the first item.

A 16-year-old boy comes to the office because of a 2-week history of six to eight daily episodes of loose, watery stools. The episodes are associated with abdominal cramps. His stool has not contained any blood. Medical history is significant for recurrent sinopulmonary infections typically treated with oral antibiotics. His last infection occurred 1 month ago and resolved within 10 days. He currently takes no medications and is not sexually active. He has no history of recent travel. He does not smoke cigarettes, drink alcoholic beverages, or use other substances. BMI is 18 kg/m2. Temperature is 37.7°C (99.9°F), pulse is 100/min, respirations are 18/min, and blood pressure is 110/60 mm Hg. Lungs are clear to auscultation. Cardiac examination discloses no abnormalities. Bowel sounds are normoactive. Abdomen is soft; palpation discloses mild, diffuse tenderness but no masses or hepatosplenomegaly. Digital rectal examination discloses brown stool; test of the stool for occult blood is negative. A culture of the stool is obtained. Acid-fast smear of a stool specimen shows numerous 6-µm ovoid oocysts.

62. Which of the following is the most likely causal agent of this patient's condition?

(A) *Cryptosporidium parvum*
(B) *Entamoeba histolytica*
(C) Norovirus
(D) *Salmonella typhi*
(E) *Shigella flexneri*

63. Appropriate treatment is prescribed for the patient's gastrointestinal infection. It is noted that this is the patient's sixth illness during the past 18 months. In determining whether this patient has an immune deficiency, which of the following is the most appropriate diagnostic study to obtain at this time?

(A) CD4+ T-lymphocyte count
(B) CD8+ T-lymphocyte count
(C) Serum complement concentrations
(D) Serum interferon gamma assay
(E) Serum quantitative immunoglobulin concentrations

END OF SET

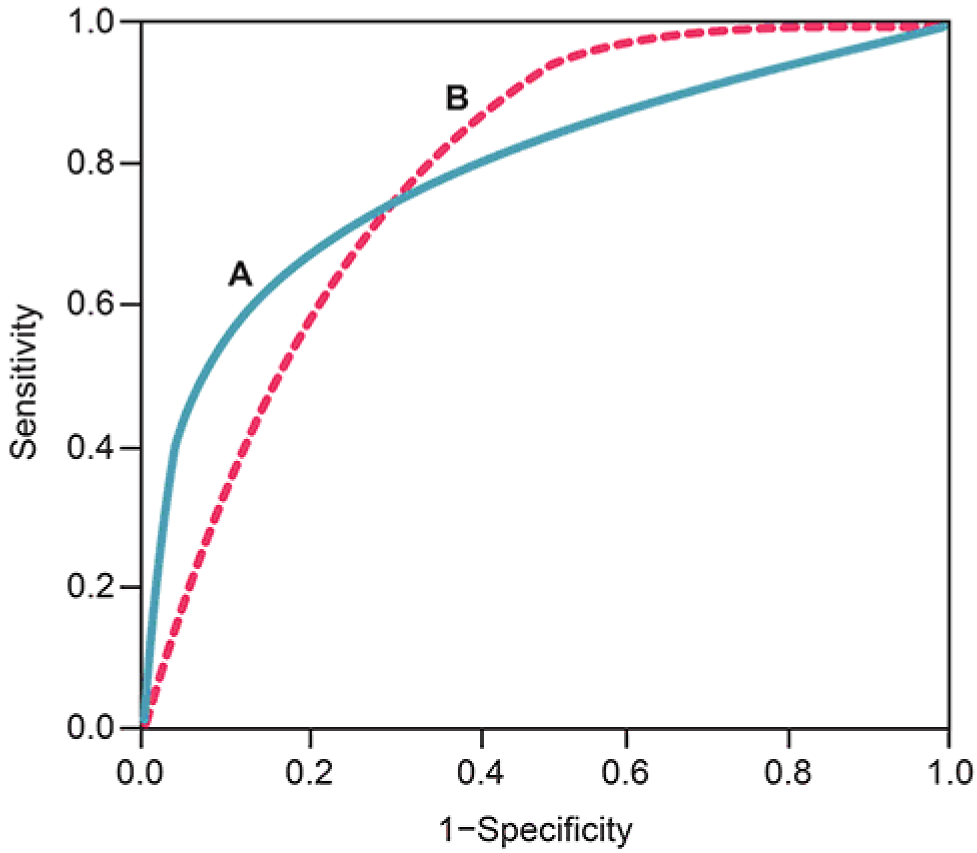

64. Physicians at an ambulatory clinic would like to develop a rapid screening test to diagnose influenza virus infection after the clinic reported 20% more cases of influenza virus infection this year than any other year. Patients at the clinic currently wait almost 90 minutes for results of a nasopharyngeal swab test to determine if they have influenza virus infection. The physicians develop two tests, Test A and Test B, which take only 10 minutes to yield results. The receiver operator characteristic (ROC) curves for the two tests are shown. Based on these data, which of the following is the most appropriate conclusion to draw about Test A compared with Test B?

(A) In the high false positive rate range, Test A is more accurate than Test B
(B) In the high sensitivity range, Test A is more accurate than Test B
(C) In the low false positive rate range, Test A is more accurate than Test B
(D) In the low sensitivity range, Test B is more accurate than Test A

65. A 5-year-old boy is brought to the office by his parents because of a 2-month history of progressive difficulty climbing stairs, playing on playground equipment, and rising from a chair. He has mild cognitive delay for which he is receiving academic assistance, and he has had delayed milestones such as walking. He otherwise is healthy. He has no siblings. Family history is unremarkable; neither of his parents has any siblings. The patient's vital signs are within normal limits. Physical examination shows hip girdle proximal muscle weakness and increased circumference of the calves bilaterally. The patient has to use his arms to stand up. Serum creatine kinase activity is 25,000 U/L (N=20–200). Genetic testing is ordered. Which of the following mutations is most likely to be identified in this patient?

(A) Frameshift insertion in the gene encoding alpha-1 (III) collagen
(B) Large deletion in the gene encoding dystrophin
(C) Missense mutation in the gene encoding fibrillin 1
(D) Splice-site mutation in the gene encoding spectrin
(E) Translocation involving the gene encoding myosin I

66. Researchers are attempting to recruit patients from the primary care clinic of a large urban hospital to take part in a minimal-risk research study. When initiating the informed consent process, it is observed that African American patients tend to exhibit a greater reluctance to take part in the study. During interim review, data confirm low enrollment totals among African Americans in the community. Which of the following is the best course of action to take?

(A) Ask the institutional review board to waive the requirement for informed consent because it is deterring potential participants
(B) Convene a representative focus group of potential participants to ask questions regarding reluctance to enroll
(C) Focus recruitment efforts on White patients who are more likely to enroll and acknowledge this study limitation in the publication
(D) Recognize this as an unavoidable impediment and continue the recruitment practices in the same manner
(E) Require all patients to take part in the study if they wish to receive care in this clinic to ensure a representative sample

67. A 54-year-old woman comes to the office because of a 10-month history of moderate to severe upper abdominal pain, nausea after eating, and a "burning" sensation in her throat and chest. This is her third visit for these symptoms during the past 3 months. She says that the recommended diet modification and weight loss regimen have not relieved her symptoms, but she later acknowledges that her diet changes have been minimal and she has not been exercising regularly. She takes over-the-counter omeprazole daily but says she is unsure how many tablets she takes. She says her symptoms have caused her to use all of her sick days at work and she is "miserable." She says she is convinced that she has Barrett esophagus. BMI is 29 kg/m^2. Vital signs are within normal limits. She appears anxious and preoccupied with her symptoms. Physical examination shows no abnormalities. On mental status examination, she is irritable but is otherwise communicative and fully oriented. Cognition is intact. She uses medical terms with ease. An upper gastrointestinal series 1 month ago showed no abnormalities. The patient requests an endoscopy because she read about them while researching her symptoms on the Internet. Which of the following is the most appropriate next step in management?

(A) Ask the patient if she would like to see a specialist
(B) Ask the patient to describe her goals for and expectations of treatment
(C) Ask the patient to further explain why she thinks she has Barrett esophagus
(D) Discuss why the patient has not made the suggested changes to her diet and exercise
(E) Encourage the patient to describe how she feels about having an unresolved medical condition

68. A 64-year-old woman comes to the office because of a 5-month history of pain in her right calf on exertion. The pain occurs when she walks and resolves within 5 minutes of rest. Initially, the pain developed after the patient walked three blocks, but now it occurs after she walks only one block. She has no other symptoms. Medical history is remarkable for hypertension, hyperlipidemia, and type 2 diabetes mellitus. Medications are metformin, lisinopril, and atorvastatin. She does not smoke cigarettes, drink alcoholic beverages, or use any other substances. BMI is 31 kg/m^2. Temperature is 37.0°C (98.6°F), pulse is 80/min, respirations are 16/min, and blood pressure is 120/80 mm Hg. Oxygen saturation is 95% on room air. Ankle brachial index is 0.6 on the right and 0.8 on the left. Physical examination discloses a left carotid bruit, a right femoral bruit, and decreased pedal pulses bilaterally. Laboratory studies show a hemoglobin A_{1c} of 7.0%. The results of additional laboratory studies, including urinalysis, are within the reference ranges. The most appropriate pharmacotherapy for this patient targets which of the following?

(A) Cyclooxygenase
(B) Factor Xa
(C) Nitric oxide synthase
(D) Phosphodiesterase
(E) Prostacyclin synthase

The pharmaceutical advertisement on the following pages is for use with items #69 and 70 on page 56.

WHEN CONSISTENT FOCUS AND IMPULSE CONTROL ARE NEEDED

Essepro XL tabs

(focophenydate sodium) 10 mg, 25 mg

- **Better** sustained action than other medications in its class
- **Improved** late afternoon performance aids in homework
- **No** adverse impact on sleep

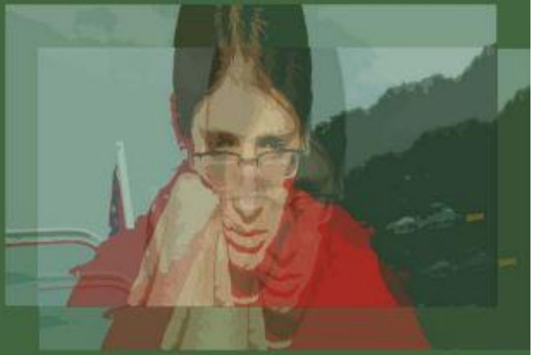

In the Featured Outcomes of Concentration in US children (FOCUS) study, Essepro XL 10 mg and 25 mg were compared with methylphenidate XL and placebo as monotherapy for attention deficit-hyperactivity disorder (ADHD) in children ages 6–17 years in a 12-week, double-blind trial. Outcomes for at least 50 patients in each of the four treatment arms were collected. The criteria for enrollment were based on DSM-V criteria for ADHD and, in some cases, physician assessment outside of DSM criteria. The FOCUS study used the NBMEC test of concentration. Previous studies have demonstrated that a score of 3.5 or greater is consistent with current attention to task. Both Essepro XL 10 mg and 25 mg demonstrated sustained duration scores of >3.5 on the NBMEC test of concentration.[1,2]

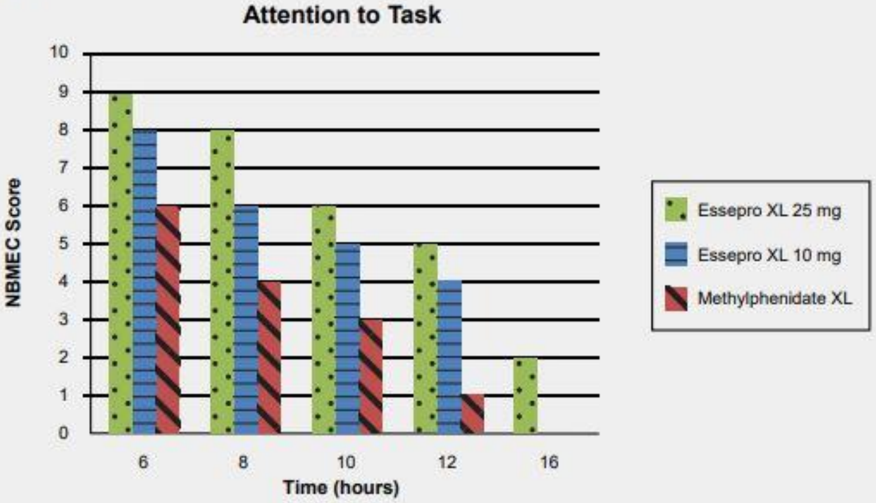

WARNING: Essepro XL (focophenydate sodium) is a schedule II controlled substance that can lead to abuse or dependency.

Essepro XL is approved for children ages 6 to 12 years and adolescents ages 13 to 17 years as once daily monotherapy for the treatment of ADHD.

The safety and efficacy of Essepro XL in adults with ADHD have not been established.

Most Commonly Reported Adverse Events in the FOCUS Trial (a 12-week, active-comparator, placebo-controlled trial[3])

Adverse Events	Essepro XL 25 mg (n=55)	Essepro XL 10 mg (n=50)	Methylphenidate XL (n=52)	Placebo (n=57)
Decreased appetite	11	7	6	7
Insomnia	8	7	6	5
Irritability	8	6	7	11
Tachycardia	7	5	3	1
Headache	7	4	6	2
Emotional lability	7	3	2	0
Dizziness	6	7	8	4
Weight loss >2 kg	5	3	1	0
Nausea	4	3	8	4
Abdominal pain	3	3	4	4
Vomiting	1	1	2	3
New onset tic	1	1	0	0

Important Safety Information

Essepro XL should not be used in patients with known hypersensitivity to stimulant medications, such as methylphenidate.

Use with caution in patients with a history of hypertension or who, on two separate occasions, have had systolic blood pressure measurements greater than the 95th percentile for age and height.

Essepro XL should not be used in patients with a history of hyperthyroidism, symptomatic cardiovascular disease, glaucoma, psychotic symptoms, drug abuse, or monoamine oxidase inhibitor (MAOI) or selective-serotonin reuptake inhibitor (SSRI) use.

Psychosis, mania, growth suppression, visual disturbances, and aggression have been reported in patients taking stimulant medications.

Dosing

Essepro XL is available in 10 mg and 25 mg tablets. It is recommended that Essepro XL be taken in the morning with food.

Tablets should be swallowed whole and not divided or crushed.

1. Jones T, Barone M. Prevalidation of attention to task scale in children and adolescents. *USMLE J Psychometric Measures*. 2011;34:112-117. 2. Smith TA, Mathes B. Kinetics and efficacy of once daily focophenydate sodium for children and adolescents with attention deficit-hyperactivity disorder. *USMLE J Attention Distraction*. 2012;9:35-42. 3. Ellis M, Carr S. Safety of focophenydate sodium in children and adolescents with attention deficit-hyperactivity disorder: results of the 12-week FOCUS study. *USMLE J Ped Psych*. 2013;44:18-22.

NB Pharma, Inc.

Essepro XL tabs
(focophenydate sodium) 10 mg, 25 mg

Items #69–70 refer to the pharmaceutical advertisement displayed on the previous pages.

An 18-year-old woman comes to the university health center requesting medication to improve her concentration. She says she needs to maintain a high grade-point average so that she will be accepted into graduate school. She graduated in the top 10% of her class at a selective preparatory high school. She is taking six classes this semester and says that she is having trouble keeping up with her workload. Several of her friends take medication for attention-deficit/hyperactivity disorder (ADHD) and have told her that these medications help them study and perform better on examinations. The patient says she took some of her roommate's Essepro XL and was able to study for hours without being distracted. She says Essepro XL has the added benefit of suppressing her appetite, which might help her lose weight. Medical history is remarkable for asthma and seasonal allergies since early childhood. Medications are albuterol inhaler as needed, fluticasone inhaler twice daily, and oral 24-hour loratadine 10 mg with pseudoephedrine once daily. BMI is 30 kg/m^2. Blood pressure is 130/84 mm Hg; other vital signs are within normal limits. The lungs are clear to auscultation. Cardiac examination discloses a midsystolic click. The patient has an advertisement for Essepro XL, as shown, and asks the physician if she could be prescribed this medication to improve her concentration and to lose weight. The physician decides not to prescribe Essepro XL for this patient.

69. Based on the advertisement, which of the following is the most appropriate rationale for this decision?

(A) Essepro XL is contraindicated in patients who also are taking stimulant medications
(B) Essepro XL is likely to exacerbate the patient's heart condition
(C) Essepro XL is not indicated for this patient
(D) The patient is likely to share Essepro XL with her friends
(E) There is no evidence that Essepro XL will help the patient lose weight

70. Based on the advertisement, which of the following is the greatest advantage of Essepro XL over methylphenidate XL?

(A) Decreased likelihood of disrupted sleep patterns
(B) Decreased potential for dependency
(C) Decreased rates of nausea and anorexia
(D) Increased academic performance
(E) Increased availability of safety data
(F) Increased duration of action

END OF SET

---

71. A 29-year-old woman comes to the office for counseling. She learned that she is HIV-positive 1 month ago and she believes that she most likely contracted the infection approximately 6 months ago. Laboratory studies ordered in preparation for this visit show a CD4+ T-lymphocyte count of 800/mm^3 and undetectable serum HIV RNA levels. She has no symptoms of AIDS. She has never been pregnant, but she is considering pregnancy and wants to know her risk for transmitting HIV to future children. Which of the following is the most accurate statement?

(A) As a subgroup, newborns have the fastest rate of increase in HIV infection
(B) Intrapartum transmission accounts for less than 50% of neonatal HIV infection
(C) Maternal viral load is the best predictor of perinatal transmission of HIV infection
(D) The risk for perinatal transmission is higher among women who conceive within 1 year of initial HIV infection
(E) Route of delivery does not influence the risk for perinatal transmission of HIV infection

72. A 50-year-old woman who has been your patient for the past 3 years comes to the office because of heartburn and increased belching and bloating after meals for the past month. Antacids provide little relief. The symptoms tend to occur after eating. She is currently asymptomatic. She has never had an operation. The patient identifies as African American. Physical examination shows mild scleral icterus and a 4-cm mass in the epigastric area. Which of the following is the most appropriate initial diagnostic study?

(A) CT scan of the abdomen
(B) Determination of serum amylase and lipase concentrations
(C) Endoscopic retrograde cholangiopancreatography
(D) HIDA scan
(E) Upper gastrointestinal barium study

73. A 50-year-old woman comes to the office because of the gradual onset of pain and swelling of both knees and distal interphalangeal joints of the hands during the past 12 weeks. The pain is exacerbated with movement and is accompanied by morning stiffness that usually resolves within 15 to 20 minutes. Recently the joint pain has occurred at night and has restricted her activity. Ibuprofen has provided moderate relief of her symptoms. Medical history is remarkable for type 2 diabetes mellitus, hypertension, and obesity. Additional medications include glipizide, metformin, and hydrochlorothiazide. She is 168 cm (5 ft 6 in) tall and weighs 105 kg (230 lb); BMI is 37 kg/m^2. Temperature is 37.0°C (98.6°F), pulse is 70/min, respirations are 16/min, and blood pressure is 140/90 mm Hg. Examination of the hands shows bony enlargements of the distal and proximal interphalangeal joints with mild tenderness to the interphalangeal joints. Examination of both knees shows bony enlargement with tenderness at the joint margin, limited range of motion, and mild joint effusions. Results of laboratory studies are shown:

Serum		Blood	
Rheumatoid factor	Nonreactive	WBC	8000/mm^3
Uric acid	6 mg/dL		

X-rays of the knees are most likely to show which of the following?

(A) Bone demineralization and erosions
(B) Soft tissue swelling, sclerotic joint margins, and large cystic erosions
(C) Soft tissue swelling with no evidence of cartilage or bone destruction
(D) Subchondral sclerosis of bone and asymmetric joint space narrowing
(E) No abnormal findings

74. A 17-year-old high school football player is brought to the emergency department by his parents 1 hour after sustaining an injury to his upper back during a game. He says another player struck him in the back with his helmet. Immediately following the incident, the patient noticed weakness of his lower extremities. On arrival, temperature is 36.7°C (98.0°F), pulse is 110/min, respirations are 20/min, and blood pressure is 130/80 mm Hg. Physical examination discloses point tenderness at the midthoracic spine. Muscle strength is 3/5 in the lower extremities. Sensation is diminished to pain and vibration below the lower chest margins. Deep tendon reflexes are absent in the lower extremities. Babinski sign is absent bilaterally. MRI of the spine is most likely to show which of the following?

(A) Anterior spinal artery territory ischemia
(B) Contusion of the thoracic spinal cord
(C) Lateral T7 disc herniation with nerve root compression
(D) Thoracic cord hemisection
(E) Traumatic syrinx

75. **Patient Information**
Age: 19 years
Gender: M, self-identified
Race/Ethnicity: unspecified
Site of Care: hospital

The patient who is a college student is admitted because of fever, chills, intense headache, nonproductive cough, and generalized weakness for the past 3 days. Medical history is unremarkable and he takes no medications. He does not use any substances. He works as a greenskeeper at a local golf course in western North Carolina. On admission, he appears acutely ill. He is somnolent but arousable. Temperature is 39.0°C (102.2°F), pulse is 106/min, respirations are 22/min, and blood pressure is 90/56 mm Hg. Oxygen saturation is 98% on room air. Examination of the skin shows an erythematous, macular rash with superimposed petechiae on the palms, soles, legs, and thighs. Funduscopic examination discloses no abnormalities. Neck is supple with no adenopathy. The remainder of the physical examination, including neurologic examination, discloses no abnormalities. Intravenous access is established. Lumbar puncture for examination of cerebrospinal fluid discloses an opening pressure of 210 mm H_2O; the cerebrospinal fluid is slightly turbid. ECG shows nonspecific ST-segment and T-wave abnormalities. Portable chest x-ray shows no abnormalities. Results of laboratory studies are shown:

Serum		Blood	
Urea nitrogen	23 mg/dL	Hemoglobin	11.5 g/dL
Creatinine	1.3 mg/dL	WBC	11,600/mm^3
Glucose	99 mg/dL	Neutrophils, segmented	75%
Na^+	134 mEq/L	Neutrophils, bands	15%
K^+	4.5 mEq/L	Lymphocytes	10%
Cl^-	104 mEq/L	Platelet count	43,000/mm^3
HCO_3^-	22 mEq/L	INR	1.3
Cerebrospinal fluid			
Cell count	20 cells/mm^3		
Glucose	70 mg/dL		
Lymphocytes	95%		
Proteins	75 mg/dL		
Gram stain	Negative		
India ink preparation	Negative		

Appropriate therapy is initiated and the patient's condition improves during the next week. Cultures of the blood and cerebrospinal fluid are negative. Which of the following is the most likely source of this patient's illness?

(A) Inhaling contaminated droplets
(B) Insecticide from the golf course
(C) Kissing
(D) Mosquito bite
(E) Tick bite

76. **Patient Information**
Age: 30 years
Gender: F, self-identified
Race/Ethnicity: unspecified
Site of Care: emergency department (ED)

The patient is brought by police because of a 2-hour history of worsening abdominal pain, diarrhea, and joint pain. The symptoms began while the patient was incarcerated in the local jail, where she was placed 12 hours ago after she was arrested for shoplifting. Her medical history is unknown. On arrival at the ED, the patient is irritable and sleepy; she yawns frequently. She is diaphoretic. BMI is 18 kg/m^2. Temperature is 37.1°C (98.7°F), pulse is 110/min, respirations are 30/min, and blood pressure is 145/92 mm Hg. Oxygen saturation is 94% on room air. Pupils are moderately dilated. Bowel sounds are hyperactive. The remainder of the physical examination discloses no abnormalities. Results of urine toxicology screening in this patient will most likely be positive for which of the following?

(A) Cannabinoids
(B) Cocaine
(C) Methamphetamine
(D) Opioids

77. A 39-year-old woman comes to the office because of a 6-week history of malaise, fatigue, and a 4.5-kg (10-lb) weight loss. She has not had difficulty sleeping or depressed mood. Medical history is unremarkable and she takes no medications. She does not smoke cigarettes or drink alcoholic beverages. BMI is 28 kg/m^2. Temperature is 37.2°C (99.0°F), pulse is 110/min, respirations are 12/min, and blood pressure is 140/90 mm Hg. Physical examination discloses a fine tremor. Which of the following additional signs or symptoms would be most consistent with the underlying diagnosis in this patient?

(A) Constipation
(B) Delayed relaxation reflex
(C) Heat intolerance
(D) Tenting of the skin

NOTE: THIS IS THE END OF BLOCK 2.
ANY REMAINING TIME MAY BE USED TO CHECK ITEMS IN THIS BLOCK.

Block 3: ACM
Items 78–107; Time - 45 minutes

ALL ITEMS REQUIRE SELECTION OF ONE BEST CHOICE.

78. **Patient Information**
Age: 25 years
Gender: F, self-identified
Race/Ethnicity: White, self-identified
Site of Care: office

The patient who works as an attorney at a large law firm presents because of a 1-week history of pain in and around her left upper jaw. She is unable to open her mouth wide, and when she attempts to do so she feels a snapping sensation in the joint in front of her ear at the jawbone. She has not sustained any recent trauma. She was recently diagnosed with major depressive disorder, for which she has been taking paroxetine. She remembers being advised in the past by her dentist to consider braces for her teeth, but she never wore them because they were unaffordable. Medical history is otherwise unremarkable, and she takes no other medications. Temperature is 37.0°C (98.6°F), pulse is 80/min, respirations are 20/min, and blood pressure is 104/60 mm Hg. Physical examination discloses point tenderness at the left temporomandibular joint. Examination of the ears shows good light reflex bilaterally on the tympanic membranes. After the patient removes chewing gum from her mouth, examination of the oral cavity shows pink buccal mucosa and no evidence of dental abscess or trauma. The patient should be advised to do which of the following?

(A) Avoid chewing gum and eating hard or chewy foods
(B) Consult an orthodontist
(C) Cradle the phone receiver between shoulder and jaw
(D) Open and close her jaw widely three time daily
(E) Take oxycodone as needed for pain

79. A 68-year-old woman was admitted to the intensive care unit 36 hours ago after becoming obtunded due to respiratory distress. She is now intubated and chest x-ray is consistent with congestive heart failure and pneumonia. She has a long history of morbid obesity, chronic bronchitis, schizoaffective disorder, and cigarette smoking. She is reported to have been relatively stable on haloperidol, benztropine, and lithium carbonate. Her present medication regimen includes acetaminophen, methylprednisolone, ciprofloxacin, haloperidol, benztropine, and lithium carbonate. She also requires intravenous lorazepam every 2 hours for agitation that caused her to fight the ventilator. Vital signs now show a spike in temperature to 38.9°C (102.0°F). Physical examination discloses lead pipe rigidity. Serum creatine kinase concentration is 846 U/L and serum lithium concentration is 0.86 mEq/L (therapeutic=0.5–1.5). Arterial blood gas analysis while breathing 40% oxygen is shown:

P_{O_2}	68 mm Hg
P_{CO_2}	36 mm Hg
pH	7.46
O_2 saturation	91%

At this time it is most important to discontinue which of the following medications?

(A) Benztropine
(B) Ciprofloxacin
(C) Haloperidol
(D) Lorazepam
(E) Methylprednisolone

80. A 35-year-old woman comes to the office because she noticed a lump in her neck 2 months ago. She has not had pain, difficulty swallowing, or change in her voice. She is a native of Germany and immigrated to the United States 10 years ago. Medical history is significant for Hodgkin lymphoma at age 12 years treated with mantle radiation. Hypothyroidism was diagnosed at age 25 years. Her only routine medication is levothyroxine. The patient has smoked one pack of cigarettes daily for the past 10 years. She appears well. BMI is 26 kg/m^2. Temperature is 36.6°C (97.8°F), pulse is 82/min, respirations are 18/min, and blood pressure is 108/66 mm Hg. Physical examination discloses a 2.5-cm (1-in) nodule in the right lobe of the thyroid gland. There is also a 1-cm (0.4-in) nodule in the left thyroid lobe. Results of serum thyroid function tests show a thyroid-stimulating hormone concentration of 0.6 μU/mL and a free thyroxine concentration of 1.7 ng/dL (N=0.7–1.8). Which of the following factors in this patient is most indicative of a poor prognosis?

(A) Cigarette smoking
(B) Hyperthyroidism
(C) Iodine deficiency
(D) Presence of multiple nodules
(E) Radiation exposure

81. A 38-year-old man with type 1 diabetes mellitus and hemochromatosis comes to the office for a routine health maintenance examination. Medical history is otherwise unremarkable. His conditions have been well managed with therapeutic phlebotomy and insulin therapy. He currently feels well. BMI is 24 kg/m^2. Temperature is 36.7°C (98.1°F), pulse is 88/min, respirations are 16/min, and blood pressure is 130/84 mm Hg. The patient's skin is not discolored and sclerae are white. Physical examination discloses no abnormalities. Results of laboratory studies are shown:

Serum		Blood	
ALT	72 U/L	Hematocrit	30%
AST	64 U/L	Hemoglobin	9.8 g/dL
Bilirubin, total	0.7 mg/dL	Hemoglobin A_{1c}	6.6%
Glucose, fasting	134 mg/dL		

This patient should be counseled that he is at increased risk for mortality if he consumes which of the following foods?

(A) Bean sprouts
(B) Berries
(C) Raw oysters
(D) Undercooked eggs

82. A 24-year-old woman with asthma comes to the office because of a 2-week history of mild shortness of breath and a nonproductive cough that occurs occasionally during the day and frequently at night. During this time, she has required use of her albuterol inhaler twice daily. Medical history is otherwise unremarkable and her only other medication is an oral contraceptive. The patient drinks three to four alcoholic beverages weekly and does not smoke cigarettes. She appears alert and comfortable. BMI is 25 kg/m^2. Temperature is 37.2°C (99.0°F), pulse is 88/min, respirations are 16/min, and blood pressure is 114/72 mm Hg. Oxygen saturation is 97% on room air. FEV_1 is 70% of predicted. Physical examination discloses no abnormalities. Which of the following is the most appropriate next step in pharmacotherapy?

(A) Add inhaled fluticasone
(B) Add inhaled tiotropium
(C) Add oral azithromycin
(D) Add oral montelukast
(E) Increase the dosage of the inhaled albuterol to four times daily
(F) Prescribe a 5-day course of oral prednisone

83. A 36-year-old woman is admitted to the hospital because of a 1-day history of sore throat and fever with temperatures to 40.0°C (104.0°F). She has not had cough, dyspnea, headache, neck stiffness, rash, diarrhea, or pain. Medical history is significant for Graves disease diagnosed by her primary care physician 3 weeks ago. At that time, the patient had a goiter and reported generalized weakness and hair loss. Therapy with propranolol and methimazole was initiated, and the patient's symptoms had been improving. BMI is 20 kg/m^2. Temperature is 39.9°C (103.8°F), pulse is 123/min, respirations are 20/min, and blood pressure is 108/68 mm Hg. Oxygen saturation is 98% on room air. Physical examination shows pharyngeal erythema without exudate and a diffuse and symmetric goiter. Lungs are clear to auscultation. Cardiac examination discloses a grade 2/6 systolic murmur at the left upper sternal border. There is no hepatosplenomegaly. Results of laboratory studies are shown:

Blood		Urine	
Hematocrit	35%	Protein	1+
Hemoglobin	11.4 g/dL	WBCs	Negative
WBC	3300/mm^3		
Neutrophils, segmented	2%		
Neutrophils, bands	1%		
Eosinophils	4%		
Basophils	1%		
Lymphocytes	78%		
Monocytes	14%		
MCV	82 μm^3		
Platelet count	221,000/mm^3		

Blood cultures are ordered and methimazole is discontinued. Which of the following is the most appropriate next step?

(A) Antinuclear antibody test
(B) Bone marrow aspiration and biopsy
(C) Cefepime therapy
(D) Filgrastim therapy
(E) HIV antibody test
(F) Propylthiouracil therapy

84. A 27-year-old woman, gravida 1, para 1, has a meeting with her physician and a lactation consultant in the hospital 1 day after uncomplicated spontaneous vaginal delivery of a 3572-g (7-lb 14-oz) female newborn at 38 weeks' gestation. Pregnancy was uncomplicated. The patient has good social support. Counseling is provided. The patient has begun to produce colostrum, and the newborn latches well. The patient says she is interested in breast-feeding and is convinced that breast milk will be best for her baby, but she is worried about how long she will be able to breast-feed because she plans to return to work as soon as possible. Her job involves packing boxes in an assembly line, and she often must work for multiple hours without a break. Her employer expects her back to work in 4 weeks. Which of the following is the most appropriate recommendation?

(A) Advise continuing breast-feeding now and discussing with her employer a schedule that will allow her to pump during work hours
(B) Encourage the patient to focus on breast-feeding and find a new job once her infant is ready to discontinue breast-feeding
(C) Recommend that the patient not breast-feed her infant
(D) Remind the patient that she needs to concern herself with what is best for her baby's care
(E) Suggest using both breast-feeding and bottle-feeding so the transition to work is easier for the baby

85. A 14-year-old boy is brought to the emergency department by a school official 1 hour after he was sent to the school nurse for behaving strangely in class and then becoming sleepy and difficult to arouse. School records indicate that the patient has no health problems and takes no medications. On arrival at the emergency department, the patient is lethargic but has no signs of trauma. Temperature is 37.3°C (99.1°F), pulse is 62/min, respirations are 10/min, and blood pressure is 106/62 mm Hg. Pulse oximetry on room air shows an oxygen saturation of 96%. Pupils measure 2 mm in diameter and are minimally reactive to light. Cardiopulmonary examination discloses clear lungs and normal cardiac rhythm. Bowel sounds are decreased. The patient moans and moves all extremities to painful stimuli. The remainder of the physical examination discloses no abnormalities. The most likely cause of this patient's condition is abuse of which of the following medications?

(A) Dextromethorphan
(B) Diazepam
(C) Diphenhydramine
(D) Methylphenidate
(E) Phenylephrine

86. **Patient Information**
Age: 70 years
Gender: M, self-identified
Race/Ethnicity: unspecified
Site of Care: office

The patient who has coronary artery disease presents for an initial visit to establish care. He had a myocardial infarction 2 years ago but is now able to walk 2 miles daily with no chest discomfort. Medical history is otherwise unremarkable. Medications include atorvastatin, metoprolol, and an 81-mg aspirin daily. He smoked two packs of cigarettes daily for 40 years but quit at the time of his myocardial infarction. He drinks one glass of red wine daily and has never used other substances. Temperature is 37.0°C (98.6°F), pulse is 82/min, respirations are 16/min, and blood pressure is 150/95 mm Hg. Physical examination discloses no abnormalities. Which of the following is the most appropriate additional pharmacotherapy for this patient?

(A) Amlodipine
(B) Clonidine
(C) Hydrochlorothiazide
(D) Lisinopril
(E) No additional pharmacotherapy is indicated

87. A 27-year-old woman, who underwent resection of a grade 2 astrocytoma in her right parietal lobe 6 weeks ago, is evaluated in the outpatient rehabilitation facility where she has been receiving ongoing care. Medical history otherwise is unremarkable. Her only medication is levetiracetam. The patient is right-hand dominant. She works as a graphic designer. BMI is 22 kg/m^2. Temperature is 37.0°C (98.6°F), pulse is 72/min, respirations are 18/min, and blood pressure is 110/60 mm Hg. Cranial nerves are intact. Muscle strength is 4/5 in the left upper and lower extremities and 5/5 in the right upper and lower extremities. There is mild spasticity at the left knee and ankle. Sensation to pinprick and proprioception are intact, but the patient has left extinction to double simultaneous stimulation, both tactile and visual. She is alert and oriented to person, place, and time. Her short-term memory is intact, and she can spell "world" backward. Speech is fluent. Gait shows mild circumduction; she uses a 4-point cane. Finger-nose testing discloses no ataxia. In considering this patient's ability to resume work, focused evaluation to determine which of the following is most likely to be helpful?

(A) Capacity to articulate ideas and thoughts
(B) Degree of control over her left hand when typing on a keyboard
(C) Effect of hemi-inattention on her visual spatial skills
(D) Overall ability to ambulate

88. A 9-year-old boy is brought to the office by his mother for evaluation of a 6-month history of abnormal movements that the mother describes as sudden, repetitive jerks often involving his shoulders and arms. She also has witnessed multiple episodes of repetitive eye blinking and throat clearing. There is no aura prior to the events. Initially, the episodes occurred approximately once weekly, but now they seem to occur daily and are interfering with his daily activities. His school performance has been acceptable, but his teacher says he is easily distracted and has difficulty sitting through class without fidgeting. Medical history is unremarkable, and he takes no medications. The patient is 133 cm (4 ft 4 in; 50th percentile) tall and weighs 29 kg (64 lb; 50th percentile). Vital signs are within normal limits. The patient is restless but cooperative. Muscle tone, deep tendon reflexes, and gait are normal; there is no tremor. The remainder of the neurologic examination discloses no focal abnormalities. During the examination, the patient experiences an episode of brief repetitive eye blinking; there is no change in consciousness during or after the episode. Based on these findings, this patient is most likely to develop which of the following?

(A) Hepatolenticular degeneration (Wilson disease)
(B) Huntington disease
(C) Intellectual disability
(D) Obsessive-compulsive disorder
(E) Seizure disorder

89. A 32-year-old woman is brought to the emergency department by a friend because of a 2-hour history of nausea, vomiting, sweating, and malaise. On arrival, the patient appears depressed. She says she took "several handfuls" of extra-strength acetaminophen and drank a pint of vodka 3 hours ago in an attempt to hurt herself. Medical history is significant for type 1 diabetes mellitus, major depressive disorder, and seizure disorder. Medications are insulin lispro, insulin glargine, fluoxetine, and phenytoin. She also takes three extra-strength acetaminophen tablets three to four times weekly for headaches or other muscle aches and pains. Vital signs are within normal limits. Palpation of the abdomen discloses mild right upper quadrant tenderness. The remainder of the examination discloses no abnormalities. Results of laboratory studies are shown:

Serum		Blood	
ALT	160 U/L	PT	12 seconds
AST	420 U/L	INR	1.0
Amylase	70 U/L		
Bilirubin, total	1.1 mg/dL		
Urea nitrogen	10 mg/dL		
Creatinine	1.0 mg/dL		
Na^+	132 mEq/L		
K^+	3.2 mEq/L		
Cl^-	108 mEq/L		
HCO_3^-	18 mEq/L		
Acetaminophen	250 µg/mL		
Alcohol	327 mg/dL		
HBcAb	Positive		
HBsAb	Positive		
HBsAg	Negative		
Hepatitis C antibody	Negative		

Which of the following factors in this patient's history most strongly indicates a poor prognosis?

(A) Chronic hepatitis B infection
(B) Concurrent alcohol consumption
(C) Fluoxetine therapy
(D) Type 1 diabetes mellitus

Items #90–91 are part of a sequential item set. In the actual examination environment, you will **not** be able to view the second item until you click "Proceed to Next Item." After navigating to the second item, you will not be able to add or change an answer to the first item.

An 11-year-old girl is brought to the emergency department by her parents because of a 1-day history of shortness of breath, fatigue, excessive thirst, and frequent urination. The parents say the onset of these symptoms coincided with an upper respiratory tract infection. Medical history is significant for type 1 diabetes mellitus. The patient's only medication is insulin. BMI is 23 kg/m^2 (93rd percentile). Temperature is 36.0°C (96.8°F), pulse is 110/min, respirations are 28/min, and blood pressure is 95/65 mm Hg. Oxygen saturation is 97% on room air. The patient appears moderately dehydrated and drowsy, but she is able to speak easily when prompted and communicates in a lucid fashion. Her respiratory pattern is rapid and deep, and she is using accessory muscles of respiration. Results of laboratory studies are shown:

Serum		Arterial blood gas analysis on room air	
Acetone	45 mg/dL (N<10)	P_{O_2}	95 mm Hg
Glucose	693 mg/dL	P_{CO_2}	26 mm Hg
		PH	7.16
		Base excess	−16 mEq/L

90. In addition to volume expansion, which of the following is the most appropriate intervention at this time?

(A) Bolus of intravenous sodium bicarbonate
(B) Continuous infusion of intravenous short-acting insulin
(C) Intermittent boluses of intravenous short-acting insulin
(D) Subcutaneous administration of half short-acting and half intermediate-acting insulin
(E) Subcutaneous administration of intermediate-acting insulin

91. An infusion of intravenous short-acting insulin is begun. Four hours later, laboratory studies are obtained, and results are shown:

Serum		Arterial blood gas analysis on room air	
Acetone	30 mg/dL (N<10)	pH	7.24
Glucose	385 mg/dL		

Shortly thereafter, the patient develops a headache and then suddenly loses consciousness. Physical examination shows increased muscle tone and extensor posturing of the extremities in response to painful stimuli. Which of the following is the most appropriate next step in management?

(A) Administration of sodium bicarbonate
(B) CT scan of the head
(C) EEG
(D) Fosphenytoin therapy
(E) Mannitol therapy

END OF SET

92. A 67-year-old woman with osteopenia comes to the office because of a 6-month history of increasingly severe, constant pain in her left hip and thigh. Before her symptoms began, she walked 1 mile daily. She now walks with a limp and must use a walker. Medical history also is remarkable for hypertension. Medications are hydrochlorothiazide, ibuprofen, and calcium and vitamin D supplementation. Her DEXA scan T-score was –1.8 1 year ago. Vital signs are within normal limits. Examination of the left lower extremity produces mild groin tenderness. Passive range of motion of the left hip is limited by pain. Serum studies show a calcium concentration of 8.6 mg/dL, intact parathyroid hormone concentration of 55 pg/mL (N=10–65), and 25-hydroxyvitamin D concentration of 30 ng/mL (N>20). X-rays of the left hip and femur show cortical thickening of the subtrochanteric region. MRI of the left hip and femur shows moderate edema of the bone marrow and soft tissue in the subtrochanteric region; there is cortical discontinuity laterally. Which of the following is the most appropriate next step in management?

(A) Bisphosphonate therapy
(B) Brace stabilization
(C) Calcitonin therapy
(D) Cast immobilization
(E) Intra-articular dexamethasone injection
(F) Surgical fixation
(G) Observation only

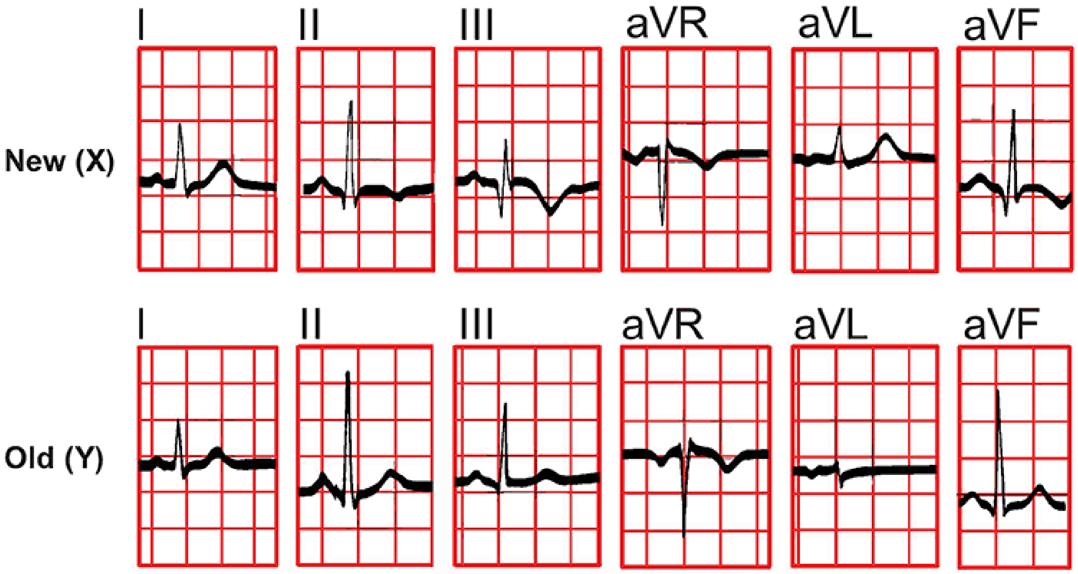

93. **<u>Patient Information</u>**
Age: 65 years
Gender: M, self-identified
Race/Ethnicity: unspecified
Site of Care: hospital

The patient is admitted because of a 4-week history of progressive shortness of breath on exertion and swelling of the lower extremities. The shortness of breath also awakens him at night. Medical history is remarkable for hypertension and type 2 diabetes mellitus. Medications are amlodipine, metformin, and daily aspirin. He does not smoke cigarettes, drink alcoholic beverages, or use other substances. Temperature is 37.0°C (98.6°F), pulse is 90/min and regular, respirations are 20/min, and blood pressure is 142/64 mm Hg. Oxygen saturation is 92% on room air. He appears mildly uncomfortable. Jugular venous pressure is 12 cm H_2O. Auscultation discloses inspiratory crackles to the mid lung fields bilaterally. Cardiac examination discloses an S_3 heard at the base. Abdominal examination shows no abnormalities. There is bilateral pitting edema to the knees. Results of laboratory studies are shown:

Serum		Blood	
Creatine kinase	35 U/L	Hematocrit	34%
Urea nitrogen	18 mg/dL	Hemoglobin	11.4 g/dL
Creatinine	1.3 mg/dL		
Na^+	133 mEq/L		
K^+	4.9 mEq/L		
Cl^-	102 mEq/L		
HCO_3^-	19 mEq/L		
Troponin I	<0.4		

ECG is shown. Administration of which of the following is contraindicated in this patient?

(A) Digoxin
(B) Furosemide
(C) Lisinopril
(D) Metoprolol
(E) Spironolactone

94. A 62-year-old man comes to the office for a routine follow-up examination 1 year after undergoing hemicolectomy for stage III colon cancer. Postoperative serum carcinoembryonic antigen concentration was 1 ng/mL (N≤3.0) at that time. The patient had completed a 6-month course of adjuvant chemotherapy. Today, he says he feels well and reports no symptoms. Medical history otherwise is unremarkable and he currently takes no medications. Vital signs are within normal limits. Physical examination shows only a well-healed midline surgical scar. Colonoscopy shows no lesions, polyps, or recurrence of cancer at the anastomosis site. Serum carcinoembryonic antigen concentration is 20 ng/mL. PET/CT scans of the chest, abdomen, and pelvis show a new 1-cm pulmonary nodule with irregular borders and increased uptake in the left lower lung lobe; the nodule does not involve the pleura or other major structures, and there is no pleural effusion. Which of the following is the most appropriate next step in management?

(A) Bronchoscopy
(B) Chemotherapy
(C) Discussion of palliative care
(D) Radiation therapy
(E) Resection of the nodule

95. A 64-year-old man is admitted to the hospital for evaluation of anemia and constant, nonradiating epigastric abdominal pain that began 3 days ago. He rates the pain as a 4 on a 10-point scale and says it partially improves after eating. He has not had nausea or vomiting. Medical history is remarkable for hypercholesterolemia and hypothyroidism. Medications are amlodipine and levothyroxine. He has no allergies. He drinks alcoholic beverages only occasionally and does not smoke cigarettes. Temperature is 36.7°C (98.0°F), pulse is 90/min, respirations are 14/min, and blood pressure is 110/70 mm Hg. Cardiopulmonary examination discloses no abnormalities. Abdominal examination discloses mild tenderness in the epigastrium with no guarding or rebound. Test of the stool for occult blood is positive. Hemoglobin concentration is 8.0 g/dL. Upper endoscopy shows a nonbleeding ulcer; biopsy of the ulcer is done, and results show a polymorphic infiltrate of small cells with reactive follicles that stain positive for B lymphocyte markers CD19 and CD20. In addition to prescribing omeprazole therapy, which of the following is the most appropriate initial treatment?

(A) Clarithromycin and amoxicillin therapy
(B) Cyclophosphamide and metronidazole therapy
(C) Radiation therapy
(D) Rituximab therapy
(E) Surgical resection

96. A 19-year-old man comes to the urgent care center because of severe lower abdominal and scrotal pain after he was elbowed in the groin 1 hour ago during a basketball game. The pain decreased 15 minutes after the incident and he continued to play in the game. Approximately 20 minutes later, the pain resumed with increased intensity, causing him to vomit. He has otherwise felt well. Medical history is unremarkable and he takes no medications. Family history is unremarkable. Temperature is 37.0°C (98.6°F), pulse is 100/min and regular, respirations are 14/min, and blood pressure is 150/88 mm Hg. The patient is writhing in pain. Bowel sounds are normoactive; the abdomen is soft with no tenderness. Genitourinary examination discloses an exquisitely tender left testicle that is situated in the transverse plane. The patient is fearful that this injury may cause him to be infertile. At this time it is most appropriate to inform the patient of which of the following?

(A) His fertility will be adversely affected because of loss of functional testicular tissue
(B) His fertility will most likely be unaffected following immediate reversal of the condition
(C) His fertility will not be adversely affected with conservative management
(D) Whether his fertility will be affected cannot be predicted without further evaluation and testing

97. A 39-year-old woman at 32 weeks' gestation returns to the office for a prenatal visit. She previously used intravenous heroin and has been in a methadone maintenance program for the past 2 years, including throughout this pregnancy. She admits to cigarette use and occasional cocaine and amphetamine use during the early part of this pregnancy. In the past 2 months she has tested negative for any drug use other than methadone. You have discussed with her the probability of a prolonged hospitalization of this neonate if there are signs of withdrawal after delivery. At this visit, she tells you that she wishes to breast-feed her baby. Which of the following is the most appropriate response?

(A) "Because of your high risk for HIV infection, breast-feeding is unsafe for your baby."
(B) "Because of your past history of drug abuse, breast-feeding is unsafe for your baby."
(C) "Because you have participated in a methadone maintenance program, I encourage you to breast-feed."
(D) "Many drugs enter the breast milk and can cause problems for the baby. Let's develop a plan to give your baby safe breast milk."
(E) "Methadone, cocaine, and amphetamines do not enter the breast milk in sufficient amounts to harm the baby, so breast-feeding can be permitted."

98. A 25-year-old nulligravid woman comes to the office for an annual health maintenance examination. She has a history of polycystic ovarian syndrome and has been taking an oral contraceptive to regulate her menses. Medical history is otherwise unremarkable. Family history is remarkable for obesity. She is 168 cm (5 ft 6 in) tall and weighs 67 kg (148 lb); BMI is 24 kg/m^2. Temperature is 37.0°C (98.6°F), pulse is 82/min, respirations are 16/min, and blood pressure is 126/76 mm Hg. Physical examination shows mild facial hirsutism that appears to have remained unchanged since her last visit 1 year ago. The remainder of the physical examination shows no abnormalities. The patient should be counseled that she is at greatest risk for acquiring which of the following conditions?

(A) Diabetes mellitus
(B) Hypertension
(C) Hypothyroidism
(D) Nephrotic syndrome
(E) Uterine cancer

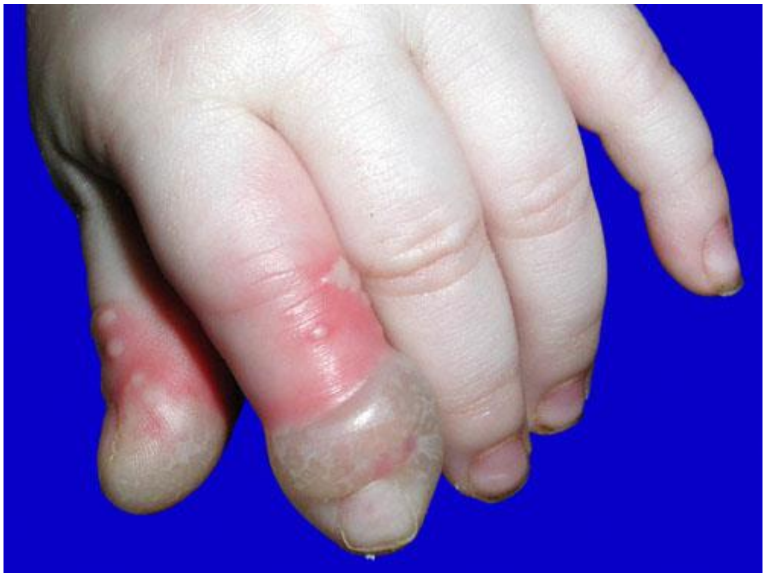

99. An 11-month-old infant is brought to the office by his mother because of a 24-hour history of progressive swelling and blistering of his left index finger and thumb. Two days ago, he developed fever and runny nose. Yesterday his mother noted sores on his left thumb and index finger, which have become larger and more blistered today. He has no known history of trauma. No one at home has similar lesions or history of skin problems. The infant attends day care 5 days weekly. Medical history is significant for recurrent diaper rashes and upper respiratory tract infections. Temperature is 38.8°C (101.8°F), pulse is 140/min, and respirations are 32/min. He appears nontoxic but fussy. Physical examination discloses rhinorrhea with clear mucus and several small erythematous papulovesicular lesions near his mouth. The left hand appears as shown. Which of the following is the most appropriate pharmacotherapy?

(A) Oral acyclovir
(B) Oral cephalexin
(C) Oral clindamycin
(D) Topical mupirocin
(E) Topical silver sulfadiazine

100. A 57-year-old woman comes to the office because of a 2-week history of intermittent palpitations. The palpitations are worse the morning of this visit and are associated with a "queasy feeling" in her upper abdomen. Her symptoms began after she started training for a 5-km race at the urging of her partner and have been increasing in intensity after each training session. Medical history is significant for type 2 diabetes mellitus treated with metformin. Family history is significant for myocardial infarction in her father. She has smoked one-half pack of cigarettes daily for the past 30 years; she does not drink alcoholic beverages. She is 152 cm (5 ft) tall and weighs 64 kg (141 lb); BMI is 28 kg/m^2. Temperature is 37.2°C (98.9°F), pulse is 98/min and irregular, respirations are 18/min, and blood pressure is 92/64 mm Hg. Oxygen saturation is 94% on room air. Auscultation of the lungs discloses bilateral basilar crackles. Cardiac examination discloses a normal S_1 and S_2, an S_4, and a grade 2/6 systolic murmur. The remainder of the physical examination discloses no abnormalities. ECG obtained today shows ST-segment elevation in the anterior leads and Q waves in leads II, III, and aVF; rhythm strip shows intermittent premature ventricular contractions. Which of the following is the most appropriate pharmacotherapy at this time?

(A) Intramuscular morphine sulfate
(B) Oral aspirin
(C) Oral diltiazem
(D) Subcutaneous enoxaparin
(E) Sublingual nitroglycerin

101. A 49-year-old woman is brought to the emergency department by her husband because of a 1-hour history of generalized weakness, dizziness, and palpitations. The patient says she has felt like she is going to faint. She also reports a 3-day history of an upper respiratory infection, for which she has taken over-the-counter cold remedies. Medical history is remarkable for hyperthyroidism diagnosed 1 month ago, for which she was given prescriptions for several medications, none of which she has filled. She is 160 cm (5 ft 3 in) tall and weighs 54 kg (119 lb); BMI is 21 kg/m^2. She appears anxious. Temperature is 38.9°C (102.0°F), pulse is 168/min and irregular, respirations are 26/min, and blood pressure is 124/56 mm Hg supine. Oxygen saturation is 97% on room air. Physical examination shows mild muscle wasting, mild pharyngeal erythema without exudate, and watery nasal discharge. Examination of the neck discloses a goiter, an audible bruit, and no jugular venous distention. Lungs are clear to auscultation with good breath sounds. Cardiac examination shows an irregularly irregular rhythm and a grade 3/6 systolic ejection murmur heard best along the left sternal border. The abdomen is soft with no tenderness. A tremor of the upper extremities is noted. There is no clubbing, cyanosis, or edema to any of the extremities. Neurologic examination shows no abnormalities. ECG shows atrial fibrillation with a rapid ventricular response. The patient is placed on a cardiac monitor and is given a 1 L bolus of isotonic saline. Which of the following is the most appropriate next step?

(A) Cardioversion
(B) Intravenous dexamethasone
(C) Intravenous diltiazem
(D) Intravenous propranolol
(E) Oral potassium iodide

102. **<u>Patient Information</u>**
Age: 20 years
Gender: M, self-identified
Race/Ethnicity: unspecified
Site of Care: student health center

The patient presents because of a 3-day history of burning on urination and penile discharge. He says he had unprotected sexual intercourse 1 week ago with a woman he met at a night club. He describes his usual state of health as excellent. Medical history is unremarkable. He takes no medications except for vitamin supplements and protein powder that he buys at the local health food store. He drinks two to three beers on weekends and smokes cannabis approximately once monthly. He is 185 cm (6 ft 1 in) tall and weighs 82 kg (180 lb); BMI is 24 kg/m^2. The patient appears embarrassed and uncomfortable. Temperature is 37.3°C (99.2°F), pulse is 80/min, respirations are 14/min, and blood pressure is 120/70 mm Hg. Physical examination discloses a circumcised penis with a whitish-yellow discharge at the urethral orifice. There are no penile lesions or ulcerations. Rapid HIV antibody test is negative. A swab of the urethral discharge is obtained and sent for culture. Antibiotic therapy is initiated for empiric treatment of chlamydia and gonorrhea, and the patient is notified that he will be contacted with the final results. Assuming that the patient's symptoms improve with the prescribed pharmacotherapy, which of the following is the most appropriate follow-up plan?

(A) Consult with the patient's parents before determining appropriate follow up
(B) Schedule follow-up examination in 1 week
(C) Schedule HIV serology testing in 3 months
(D) No specific follow up is necessary

103. A 36-year-old woman comes to the emergency department because of abdominal pain, nausea, and vomiting that began suddenly 12 hours ago and has worsened since that time. The patient rates the pain as 10 on a 10-point scale and describes it as stabbing and radiating to her back. She has not had hematemesis. Her last bowel movement was yesterday. She has never had a similar episode in the past. Medical history is unremarkable and she takes no medications. She drinks one to two alcoholic beverages weekly, usually on weekends. Temperature is 37.4°C (99.3°F), pulse is 116/min, respirations are 20/min, and blood pressure is 118/88 mm Hg. The patient appears uncomfortable. The conjunctivae are anicteric. Abdomen is mildly distended with guarding and tenderness to palpation. No telangiectasias are noted. Results of serum laboratory studies are shown:

Alkaline phosphatase	196 U/L
Bilirubin, total	1.8 mg/dL
Triglycerides	275 mg/dL
Lipase	846 U/L
Calcium	8.9 mg/dL
Urea nitrogen	16 mg/dL
Creatinine	1.8 mg/dL
Na^+	144 mEq/L
K^+	3.5 mEq/L
$Cl^−$	100 mEq/L
$HCO_3^−$	29 mEq/L
Glucose	125 mg/dL

In addition to beginning intravenous fluids, which of the following is the most appropriate next step in management?

(A) CT scan of the abdomen with contrast
(B) Fenofibrate therapy
(C) Imipenem cilastatin therapy
(D) Insulin therapy
(E) Ultrasonography of the right upper abdominal quadrant

104. A 4-month-old male infant is brought to the office by his parents because of a 1-day history of lethargy and constipation. The parents say the infant has not been breast-feeding well during the past 2 weeks and has not had a bowel movement in 2 days. He has not had fever and has not been exposed to anyone known to be ill. He was born at term to a 26-year-old woman, gravida 2, para 1, via uncomplicated spontaneous vaginal delivery. Birth weight was 3000 g (6 lb 10 oz; 25th percentile). Medical history is unremarkable and he receives no medications. Vaccinations are up-to-date. Family history is unremarkable. The patient is 59 cm (23 in; <3rd percentile) long and weighs 5.5 kg (12 lb; <3rd percentile); head circumference is 41 cm (16.1 in; 25th percentile). Temperature is 37.3°C (99.1°F), pulse is 116/min, respirations are 16/min, and blood pressure is 88/54 mm Hg. The patient is listless upon examination. Physical examination discloses weakness of the upper and lower extremities bilaterally against active resistance. Abdominal and rectal examinations disclose no abnormalities. Results of fasting laboratory studies are shown:

Serum		Urine	
Calcium	9.0 mg/dL	Specific gravity	1.012 (N<1.010)
Urea nitrogen	8 mg/dL	pH	5.5 (N=4.5−7.8)
Creatinine	0.4 mg/dL (N=0.03–0.5)	Glucose	2+
Na^+	133 mEq/L		
K^+	2.7 mEq/L		
Cl^-	105 mEq/L		
HCO_3^-	11 mEq/L		
Glucose	84 mg/dL (N=50–90)		
Phosphorus	2.5 mg/dL (N=3.8–8.2)		

Which of the following is most likely to develop in this patient?

(A) Congestive heart failure
(B) Cushing syndrome
(C) Osteomalacia
(D) Type 1 diabetes mellitus

105. **Patient Information**
Age: 44 years
Gender: F, self-identified
Ethnicity: unspecified
Site of Care: office

History
Reason for Visit/Chief Concern: "My neck and shoulder pain have come back again."

History of Present Illness:
• 5-year history of myofascial pain syndrome
• 12-month history of right-sided neck and shoulder pain; has not sustained any trauma
• rates current neck and right shoulder pain as 6/10
• daily stretching of the neck and right shoulder and naproxen therapy have not improved pain
• 6 months ago, received local anesthetic trigger point injection in the right upper trapezius muscle from another physician with complete resolution of pain for 4 months
• 48 hours after trigger point injection, developed severe itching and redness at injection site; symptoms resolved spontaneously
• informed she is allergic to local anesthetics but was not told which one was used in the injection
Past Medical History:
• 5-year history of major depressive disorder
• 10-year history of hypothyroidism
Medications:
• citalopram
• levothyroxine
• naproxen
Allergies:
• local anesthetics
Family History:
• mother: alive with fibromyalgia
Psychosocial History:
• does not smoke cigarettes or drink alcoholic beverages

Physical Examination

Temp	Pulse	Resp	BP	O_2 Sat	Ht	Wt	BMI
36.9°C (98.4°F)	77/min	15/min	117/67 mm Hg	98% on RA	163 cm (5 ft 4 in)	70 kg (155 lb)	27 kg/m^2

• Pulmonary: clear to auscultation
• Cardiac: regular rhythm; normal S_1 and S_2; no murmurs, rubs, or gallops
• Musculoskeletal: palpation of right upper trapezius muscle discloses a trigger point within a taut band of muscle that reproduces patient's neck and shoulder pain

Question: The patient requests a repeat trigger point injection. Which of the following is the most appropriate next step in management?

(A) Administer the injection using phenol as the anesthetic
(B) Administer the injection using prilocaine as the anesthetic
(C) Administer the injection using tetracaine as the anesthetic
(D) Pretreat the patient with loratadine, then administer the injection using any local anesthetic
(E) Refer the patient to an allergist for further evaluation

106. **Patient Information**
Age: 62 years
Gender: M, self-identified
Ethnicity: unspecified
Site of Care: hospital, post anesthesia care unit (PACU)

History
Reason for Admission/Chief Concern: postoperative knee pain 4 hours after undergoing total right knee arthroplasty

History of Present Illness:
- 2-year history of severe right knee pain
- patient is extubated postoperatively
- rates right knee pain 9/10
- knee pain difficult to control because administration of opioid medications causes sleepiness and episodes of apnea

Past Medical History:
- hypertension
- gastroesophageal reflux disease
- hypercholesterolemia
- hypothyroidism
- osteoarthritis

Medications:

• losartan	home, continued on admission
• hydrochlorothiazide	home, continued on admission
• omeprazole	home, continued on admission
• levothyroxine	home, continued on admission
• hydromorphone	initiated in PACU
• ketorolac	initiated in PACU
• acetaminophen	initiated in PACU

Allergies:
- no known drug allergies

Psychosocial History:
- drinks two cocktails daily
- does not smoke cigarettes or use any other substances
- retired school-teacher
- lives with his wife

Physical Examination

Temp	Pulse	Resp	BP	O_2 Sat	Ht	Wt	BMI
36.0°C	88/min awake	12/min awake	139/82 mm Hg awake	96% on 4LNC awake	178 cm	124 kg	39 kg/m^2
(96.8°F)	82/min sleeping	2/min sleeping	142/86 mm Hg sleeping	86% on 4LNC sleeping	(5 ft 10 in)	(273 lb)	

- Appearance: sleepy, but arousable to voice
- Pulmonary: decreased breath sounds at bases, otherwise clear to auscultation
- Cardiac: regular rhythm
- Extremities: dressing, wrap, and brace immobilization applied to right knee
- Neurologic: fully oriented

This item continues on the following page.

Question: Which of the following is the most appropriate next step to decrease this patient's risk for postoperative respiratory events?

(A) Administration of oxygen via high flow nasal cannula
(B) Bupivacaine femoral nerve block
(C) Chest wall impedance monitoring
(D) Continuous pulse oximetry
(E) Hydromorphone via patient-controlled analgesia

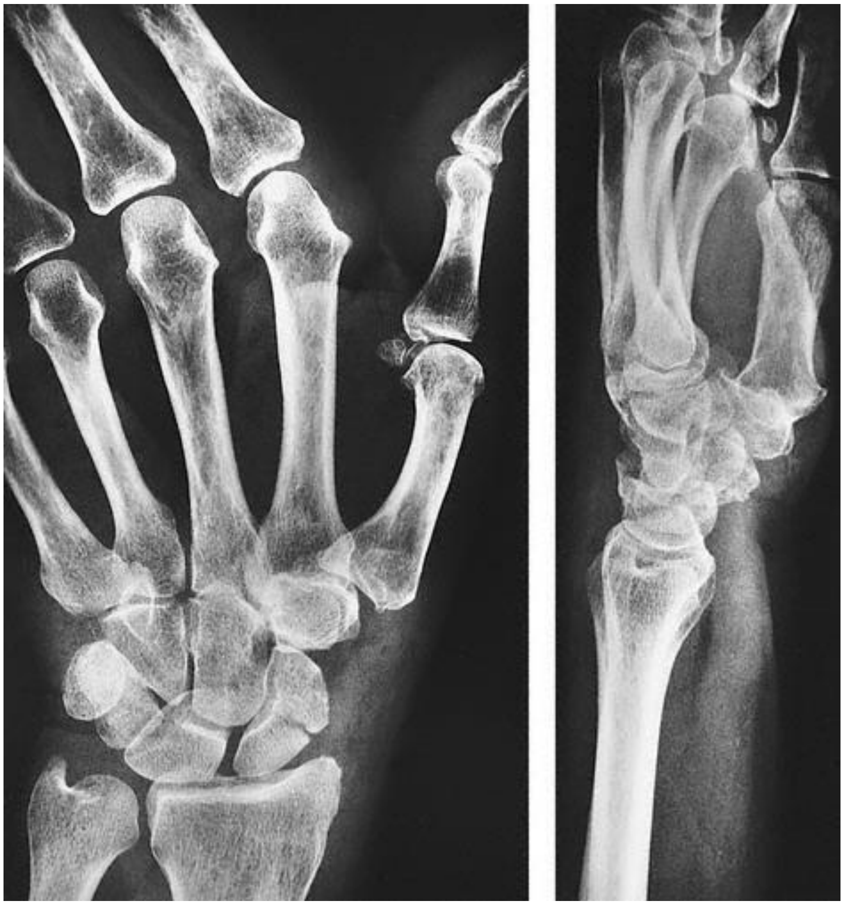

107. **<u>Patient Information</u>**
Age: 52 years
Gender: F, self-identified
Race/Ethnicity: Lebanese American, self-identified
Site of Care: office

The patient presents because of pain in her left wrist following a fall 1 week ago. She is a data entry clerk and you have been treating her for carpal tunnel syndrome for the past year. Physical examination today discloses little swelling, but there is pain with attempts to flex or extend the wrist and fingers. X-rays are shown. Which of the following is the most appropriate management?

(A) Apply an elastic wrist bandage
(B) Apply a plaster cast that incorporates the thumb
(C) Apply a plastic wrist splint
(D) Begin daily aspirin treatment and do not restrict use of the hand
(E) Refer her to an occupational therapist

NOTE: THIS IS THE END OF BLOCK 3.
ANY REMAINING TIME MAY BE USED TO CHECK ITEMS IN THIS BLOCK.

Block 4: ACM
Items 108–137; Time - 45 minutes

ALL ITEMS REQUIRE SELECTION OF ONE BEST CHOICE.

108. A 65-year-old woman who has been your patient for 8 years is being prepared for discharge from the hospital after receiving a prosthetic hip following a hip fracture. Postoperatively she received low-dose heparin therapy, and she has been able to move about with a walker. She has had diabetes mellitus for 12 years, which has been controlled with glipizide. Her hemoglobin A_{1c} 3 months ago was 7.6%. Other than the diabetes her health has been excellent and her weight has not changed in several years. She took estrogen for 4 years for hot flushes but discontinued it because of vaginal bleeding. She does not smoke cigarettes. She drinks a glass of wine or a bottle of beer daily. She lives with her 62-year-old sister, who has severe osteoarthritis but is able to prepare meals and assist her with activities of daily living. During this hospitalization the patient's blood glucose concentrations have ranged from 220 mg/dL to 280 mg/dL, for which she is being treated with insulin. She will continue insulin therapy at home. At this time it is most appropriate to arrange for home services to do which of the following?

(A) Administer daily insulin
(B) Assist the patient in building her upper-body strength
(C) Assist the patient in strengthening her lower extremity muscles
(D) Ensure that the patient follows the diabetic diet prescribed for her
(E) Measure the patient's blood glucose concentration daily

Items #109–110 are part of a sequential item set. In the actual examination environment, you will **not** be able to view the second item until you click "Proceed to Next Item." After navigating to the second item, you will not be able to add or change an answer to the first item.

A 32-year-old man comes to the office because of mild eye irritation, runny nose, nasal congestion, and postnasal drip that have occurred since he moved to his current apartment 10 months ago. He also reports occasional shortness of breath with wheezing and loss of his sense of smell. He has not had fever or cough. Medical history is unremarkable and he takes no medications. Family history is significant for nasal polyps in several family members who also have similar symptoms. The patient's vital signs are within normal limits. Physical examination discloses mildly injected conjunctivae, pale and swollen nasal turbinates, and a slightly injected pharynx. Lungs are clear to auscultation. The remainder of the examination discloses no abnormalities.

109. Which of the following factors in this patient's history most strongly predicts development of a more serious condition?

(A) Associated shortness of breath and wheezing
(B) Family history of similar symptoms
(C) Loss of sense of smell
(D) Perennial nature of symptoms

110. The patient is counseled regarding allergen avoidance, including the use of HEPA air filters, a mattress cover, and frequent vacuuming with dust removal. He returns to the office 1 month later with continued wheezing and shortness of breath. Which of the following is the most appropriate pharmacotherapy at this time?

(A) Inhaled fluticasone
(B) Nasal cromolyn
(C) Nasal oxymetazoline
(D) Oral fexofenadine

END OF SET

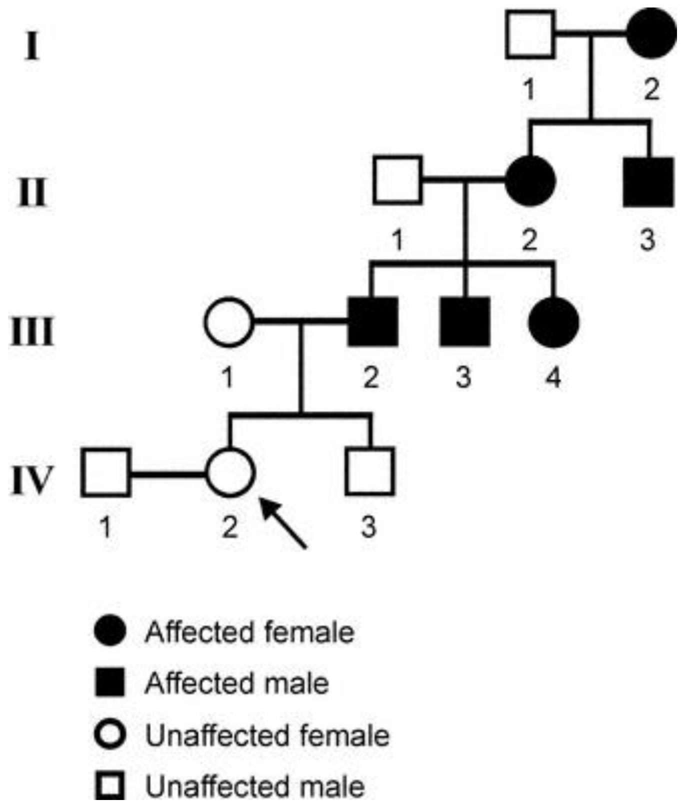

111. A 24-year-old woman comes to the office because she had a positive home pregnancy test 2 days ago. Urine pregnancy test in the office today is positive. Physical examination findings are consistent with a 14-week gestation. Ultrasonography shows a twin pregnancy with two female fetuses. This is her first pregnancy, and she says she is concerned because she has a family history of an eye disease in which family members lose their vision beginning in their 20s. Testing of her vision today discloses no abnormalities. The patient's pedigree is shown; the patient is identified as IV, 2. Which of the following is the most appropriate conclusion about the inheritance of this disorder?

(A) It is an autosomal dominant disorder
(B) It is a chromosomal aneuploidy
(C) It is indeterminant
(D) It is an X-linked dominant condition
(E) It is an X-linked recessive condition

112. An 84-year-old man, who has resided in a nursing care facility for the past 5 years, is being evaluated at the facility because a nurse discovered a sacral ulcer while bathing the patient 2 hours ago. The patient is not in pain. Medical history is significant for type 2 diabetes mellitus, congestive heart failure, chronic obstructive pulmonary disease, and peripheral vascular disease. Medications are insulin, furosemide, metoprolol, lisinopril, albuterol and ipratropium metered-dose inhalers, and 81-mg aspirin. The patient is unable to walk and spends the majority of the day in a manual wheelchair that he self-propels slowly with his feet. BMI is 19 kg/m^2. Temperature is 37.0°C (98.6°F), pulse is 62/min, respirations are 16/min, and blood pressure is 115/69 mm Hg. Oxygen saturation is 94% on oxygen at 2 L/min via nasal cannula. The patient appears frail. Lungs are clear to auscultation; breath sounds are decreased. Cardiac examination discloses no abnormalities. There is a 3 × 3 × 0.2-cm stage II ulcer on the patient's sacrum with yellowish exudate at the base and no surrounding erythema. Examination of the extremities shows edema to the calves bilaterally. Results of recent laboratory studies are shown:

Serum		Blood	
Urea nitrogen	10 mg/dL	Hematocrit	33%
Creatinine	1.1 mg/dL	Hemoglobin	10.8 mg/dL
Na^+	138 mEq/L	WBC	11,500/mm^3
K^+	4.0 mEq/L	Neutrophils	60%
Cl^-	100 mEq/L	Eosinophils	2%
HCO_3^-	24 mEq/L	Lymphocytes	33%
Cholesterol		Monocytes	5%
Total	105 mg/dL	Platelet count	135,000/mm^3

Which of the following treatment modalities is most likely to have the greatest effect on wound healing in this patient?

(A) Hypercaloric diet
(B) Pressure relief
(C) Silver sulfadiazine
(D) Wound debridement

113. A 36-year-old woman is referred to the office for evaluation of a fasting serum total cholesterol concentration of 249 mg/dL. She has a family history of early coronary artery disease (CAD) and her father died suddenly at age 46 years of myocardial infarction. She tells you that she has never had chest pain. She is not currently sexually active and has no children. The patient works as an advertising executive. She claims that her high-stress lifestyle makes it impossible for her to eat regular meals or to follow a special diet, and she usually eats fast food. She exercises two or three times a week for about 20 minutes on a treadmill. She has smoked one pack of cigarettes daily for the past 20 years. Her only medication is acetaminophen for tension headaches. She is 165 cm (5 ft 5 in) tall and weighs 76 kg (167 lb); BMI is 28 kg/m^2. Vital signs today are within normal limits. Physical examination discloses no abnormalities except for mild obesity. Institution of which of the following is the most essential step in the prevention of CAD in this patient?

(A) Biofeedback-based stress reduction program
(B) More rigorous and consistent exercise program
(C) Smoking cessation program
(D) Strict low-calorie diet
(E) Strict low-fat diet

114. A 21-year-old woman is brought to the emergency department by her parents after she told them she consumed an entire bottle of an unspecified pain medication 3 hours ago in a suicide attempt. The patient reports shortness of breath and ringing in her ears. Medical history is significant for major depressive disorder. Her only routine medication is escitalopram and she has no allergies. Temperature is 37.5°C (99.5°F), pulse is 96/min, respirations are 22/min, and blood pressure is 98/60 mm Hg. Oxygen saturation is 92% on room air. The patient appears tired and thin but is not in acute distress. She is slow to respond to questions and has difficulty following instructions. Skin is warm and dry. Examination of tympanic membranes discloses no abnormalities. Auscultation of the lungs discloses bilateral basilar crackles. Cardiac examination discloses no abnormalities. Abdomen is soft and nontender. The remainder of the physical examination discloses no abnormalities. Results of arterial blood gas analysis on room air are shown:

P_{O_2}	88 mm Hg
P_{CO_2}	26 mm Hg
pH	7.32

Which of the following is the most appropriate next step in management?

(A) Administration of lipid emulsion
(B) Administration of *N*-acetylcysteine
(C) Administration of sodium bicarbonate
(D) Endotracheal intubation
(E) Supportive care only

115. A 38-year-old woman comes to the office for an annual health maintenance examination. She has been healthy and her last cervical cytology 3 years ago was normal. The patient has been married for the past 15 years and the couple is monogamous. Today, the patient is afebrile. Pulse is 82/min and blood pressure is 130/82 mm Hg. Physical examination shows no abnormalities. Cervical cytology and human papillomavirus (HPV) testing are obtained. If results from the most recent cervical cytology and HPV test are normal, at which of the following times should this patient have her next cervical cytology?

(A) At the time of menopause
(B) In 1 year
(C) In 5 years
(D) Only if she gets pregnant
(E) The patient does not need another cervical cytology

116. A 21-year-old man, who is a member of the US Air Force, comes to the emergency department because of a 2-day history of shortness of breath on exertion and when lying down, as well as fever, chills, and swelling of his legs. Medical history is unremarkable and he takes no medications. Temperature is 38.1°C (100.5°F), pulse is 97/min, respirations are 20/min, and blood pressure is 140/91 mm Hg. Oxygen saturation is 92% on room air. The patient is dyspneic but able to speak in full sentences. Jugular venous pressure is 14 cm H_2O. Auscultation of the lungs discloses bilateral basilar crackles. Cardiac examination discloses a regular rhythm with an S_3 gallop but no murmur. Abdominal examination discloses no abnormalities. There is 1+ pitting edema of the lower extremities. Results of laboratory studies show a serum B-type natriuretic peptide concentration of 2000 pg/mL (N<100) and a leukocyte count of 13,000/mm^3. Results of serum chemistry profile and remainder of complete blood count are within the reference ranges. ECG shows no abnormalities except for nonspecific ST-segment changes. Echocardiography shows a dilated left ventricle with an ejection fraction of 0.40 and no pericardial effusion. Which of the following is the most likely long-term outcome for this patient?

(A) Complete recovery
(B) Mild diastolic dysfunction
(C) Pulmonary embolism
(D) Recurrent pericarditis
(E) Severe systolic heart failure requiring cardiac transplant

117. A 31-year-old woman who is in the US Air Force comes to the office because of a 1-day history of fever, sore throat, body aches, muscle weakness, and dehydration. Medical history is remarkable for systemic lupus erythematosus. She has no known allergies. Current medications are calcium, hydroxychloroquine, iron, and vitamin D. She has a 5-year-old son who currently has a febrile illness and a rash on his hands and feet. The patient's BMI is 24 kg/m^2. Temperature is 38.3°C (101.0°F), pulse is 96/min, respirations are 20/min, and blood pressure is 115/77 mm Hg. Physical examination shows an erythematous throat with vesicles on both tonsils. There is no ulceration or coating of the tongue. There is bilateral cervical lymphadenopathy. The lungs are clear. A rapid streptococcal test result is negative. Which of the following is the most appropriate treatment for this patient?

(A) Acyclovir
(B) Amoxicillin
(C) Amoxicillin-clavulanic acid
(D) Ibuprofen
(E) Prednisone

118. A 45-year-old woman comes to the office because of a 6-month history of progressively slurred speech. During this time, she has bitten her tongue frequently and has had difficulty swallowing food. She also has felt clumsy performing normal activities. She has not had changes in vision, sensory symptoms, or bladder dysfunction. Five years ago, she underwent cervical fusion for degenerative disc disease. She takes no medications. Vital signs are within normal limits. The patient is unable to protrude her tongue. There is deep furrowing and bilateral fasciculations of the tongue. She is dysarthric. When the patient is asked to say, “Ahh,” there is symmetric but incomplete elevation of the palate. Muscle tone is normal throughout. Muscle strength is 4/5 in the right upper extremity, and there are fasciculations in the right deltoid and biceps; muscle strength is 5/5 elsewhere. Deep tendon reflexes are 1+ in the right biceps and brachioradialis and 3+ in the triceps and right lower extremity. Electromyography and nerve conduction studies show motor denervation in the tongue and right extremities. Which of the following factors in this patient’s history most negatively impacts her life expectancy?

(A) Age
(B) Bulbar weakness
(C) Cervical fusion operation
(D) Fasciculations
(E) Gender

119. A 64-year-old woman comes to the clinic because of a 2-year history of constipation. She initially had bowel movements every other day and passed hard stools. During the past 4 months, she has had three bowel movements weekly. Fiber supplementation during the past 3 months has not increased the frequency of bowel movements. She has not had abdominal pain, fever, weight loss, or blood in the stool. She has hypertension, and her only medication is lisinopril. Colonoscopy 5 years ago and cervical cytology 3 years ago showed no abnormalities. There is no family history of colon cancer or bowel disorders. The patient does not appear to be in distress. She is 163 cm (5 ft 4 in) tall and weighs 66 kg (145 lb); BMI is 25 kg/m^2. Vital signs are within normal limits. The abdomen is soft, nontender, and nondistended. Bowel sounds are normal. Rectal examination shows decreased sphincter tone; there are no masses. Soft stool is noted in the rectal vault. Test of the stool for occult blood is negative. Which of the following is the most appropriate next step in management?

(A) Addition of bisacodyl to the medication regimen
(B) Anorectal manometry
(C) Colonoscopy
(D) Discontinuation of lisinopril
(E) Recommendation to maintain a daily bowel movement journal for 1 month

120. A 72-year-old man is brought to the emergency department by emergency medical services approximately 45 minutes after losing consciousness while attempting to stand from a seated position while watching television at home. He regained consciousness spontaneously after 5 minutes. He has had persistent light-headedness since this episode but no other symptoms. He has felt light-headed during postural changes in the past, but he has never before lost consciousness. Medical history is remarkable for ischemic cardiomyopathy with a left ventricular ejection fraction of 40%. Medications are daily metoprolol, lisinopril, spironolactone, atorvastatin, and 81-mg aspirin, as well as furosemide every other day. There have been no changes in his medication regimen during the past year. Temperature is 35.8°C (96.4°F), pulse is 30/min, respirations are 24/min, and blood pressure is 75/35 mm Hg. Oxygen saturation is 90% on room air. The patient is diaphoretic and pale. He occasionally does not respond appropriately to questions or commands, but he is oriented to person, place, and time. Skin is cool to the touch. Auscultation of the lungs discloses bilateral crackles. Which of the following is the most appropriate next step in management?

(A) Adenosine therapy
(B) Continuous infusion of dobutamine
(C) Continuous infusion of epinephrine
(D) Defibrillation
(E) Intubation and mechanical ventilation
(F) Transcutaneous pacing

121. A 25-year-old man comes to the clinic to request vaccination against human papillomavirus (HPV). Medical history is unremarkable. He takes no medications. He is sexually active with one male partner and uses condoms consistently. He is a member of the US Marine Corps. BMI is 21 kg/m^2. Vital signs are within normal limits. Physical examination shows no abnormalities. Which of the following is the most appropriate next step in management?

(A) Administer the HPV vaccine
(B) Obtain HPV serologic testing
(C) Perform an anal Pap smear for cytologic examination
(D) Use motivational interviewing to encourage abstinence

122. A 55-year-old woman with long-standing hypertension comes to the office because of a 5-month history of fatigue that worsens when she walks upstairs or exerts herself. At least three times weekly she has shortness of breath requiring the use of three pillows to sleep at night. She has not had chest pain. She has been an established patient for 10 years. During this time her hypertension had been well controlled with hydrochlorothiazide therapy; however, 2 months ago she developed increased blood pressure and lisinopril was added to her medication regimen. Medical history also is significant for mitral valve regurgitation that was evaluated by a cardiologist 3 years ago, at which time the patient was asymptomatic. BMI is 28 kg/m^2. Temperature is 36.7°C (98.0°F), pulse is 80/min, respirations are 16/min, and blood pressure is 150/85 mm Hg. Lungs are clear to auscultation. Cardiac examination discloses a diminished S_1, an audible S_3, and a high-pitched grade 4/6 systolic murmur radiating to the left axilla. Examination of the extremities shows 1+ pitting edema to the middle of the tibia bilaterally. Which of the following is the most appropriate next step in management?

(A) Add amlodipine to her medication regimen
(B) Evaluate her for valve replacement
(C) Increase the doses of hydrochlorothiazide and lisinopril
(D) Recommend a low-sodium diet that includes low-fat dairy products and fresh fruits and vegetables
(E) Recommend starting a low-impact aerobic exercise regimen for 30 minutes daily

123. A 68-year-old man is admitted to the hospital because of a 2-month history of gradually worsening dyspnea and swelling of the lower extremities. Medical history also is remarkable for obstructive sleep apnea. The patient takes no medications. BMI is 40 kg/m^2. On admission, temperature is 37.0°C (98.6°F), pulse is 84/min, respirations are 28/min, and blood pressure is 142/78 mm Hg. Oxygen saturation is 89% on room air. Auscultation of the lungs discloses decreased breath sounds on the right. Chest percussion discloses dullness and decreased fremitus. There is bilateral pedal edema. Chest x-ray shows a pleural effusion. Ultrasonography-guided thoracentesis is done and discloses serosanguineous fluid; results of laboratory studies are shown:

Serum		Pleural fluid	
Lactate dehydrogenase	189 U/L	pH	7.33
Protein		Lactate dehydrogenase	169 U/L
Total	5.3 g/dL	Protein	
		Total	3.9 g/dL

Cultures and cytology of the pleural fluid are negative. Which of the following is the most appropriate next step in management?

(A) Bronchoscopy
(B) CT scan of the chest
(C) Echocardiography
(D) Furosemide therapy
(E) Moxifloxacin therapy

124. A 46-year-old woman comes to the office for a routine health maintenance examination. She reports no symptoms. Medical history is remarkable for polycystic kidney disease, hypertension, type 2 diabetes mellitus, and hypothyroidism. The kidney disease was diagnosed 5 years ago; CT scan at that time showed enlarged kidneys with extensive cysts bilaterally. Medications are rosuvastatin, lisinopril, glyburide, and levothyroxine. BMI is 32 kg/m^2. Temperature is 37.0°C (98.6°F), pulse is 88/min, respirations are 16/min, and blood pressure is 138/82 mm Hg. Oxygen saturation is 100% on room air. Cardiopulmonary examination discloses no abnormalities. Muscle strength is 5/5 in all extremities and deep tendon reflexes are 2+ throughout all extremities. Results of fasting laboratory studies are shown:

Serum		Plasma	
Cholesterol		Copeptin	2.0 pmol/L (N=1–12)
Total	220 mg/dL	Urine	
HDL	82 mg/dL	Na^+	25 mEq/L (N<25)
LDL	110 mg/dL		
Triglycerides	140 mg/dL		
Urea nitrogen	30 mg/dL		
Creatinine	2.1 mg/dL		
Na^+	140 mEq/L		
K^+	4.0 mEq/L		
Cl^-	102 mEq/L		
HCO_3^-	26 mEq/L		
Uric acid	3.0 mg/dL		

Which of the following findings most strongly indicates a poor prognosis in this patient?

(A) BMI
(B) Kidney function
(C) Number of kidney cysts
(D) Plasma copeptin concentration
(E) Serum LDL concentration
(F) Urine sodium concentration

125. A 48-year-old woman is referred to the office because a fingerstick blood glucose concentration measured at a health fair 4 days ago was 200 mg/dL. Medical history is unremarkable and she takes no medications. BMI is 30 kg/m^2. Temperature is 37.0°C (98.6°F), pulse is 84/min, respirations are 16/min, and blood pressure is 129/79 mm Hg. The patient is not in acute distress. Physical examination discloses no abnormalities. Results of fasting laboratory studies obtained in preparation for today's visit are shown:

Serum		Blood	
Glucose	146 mg/dL	Hemoglobin A_{1c}	8.3%
		Urine	
		Microalbumin	<30 mg/24 h

The patient is counseled regarding lifestyle modifications. In addition to metformin, which of the following is the most appropriate pharmacotherapy?

(A) 81-mg Aspirin
(B) Chlorthalidone
(C) Lisinopril
(D) Metoprolol
(E) No additional pharmacotherapy is indicated

126. A 65-year-old man who has been your patient for 5 years comes to the office because he has noticed slowly progressive deformity of his left lower leg. He says, "My leg seems to be a lot more bowed than I remember; it didn't look this way last year." On further questioning, he reports that he has had aching pain in his left leg and hip for the past month, and that he has been taking ibuprofen daily for pain relief without much effect. He identifies as Swedish American. Physical examination is normal with the exception of valgus deformity of the left lower leg, with pain on palpation over the tibial shaft. X-rays of the lower leg show cortical thickening and intramedullary sclerosis in the tibia. Which of the following is the most appropriate pharmacotherapy?

(A) Alendronate, orally
(B) Calcitonin, intramuscularly
(C) Calcitonin, nasally
(D) Mithramycin, intravenously
(E) Naproxen, orally

127. A 46-year-old man with a 7-year history of spondylolisthesis and resulting chronic low back pain comes to the office for a refill of his sustained-release oxycodone. He reports persistent back pain for the past 2 months and requests an increase in the dose of oxycodone. He has had two previous unsuccessful back surgeries. The patient's friend, who drove him to the office, informed the nurse that he thinks the patient has been selling his oxycodone on the street. The patient's medical history is otherwise unremarkable and he takes no other medications. Vital signs are within normal limits. Physical examination discloses reproducible pain over the low back but no obvious muscle spasms. There is a well-healed surgical scar over the lumbar spine. Straight-leg raising test is negative bilaterally. The remainder of the physical examination, including neurologic examination, discloses no abnormalities. Which of the following is the most appropriate next step?

(A) Discontinue oxycodone
(B) Notify the police
(C) Order a random urine test for oxycodone
(D) Refer the patient to a methadone clinic
(E) Switch oxycodone to a different pain medication

128. A 40-year-old woman, gravida 2, para 2, comes to the office because of a 1-month history of weakness and intermittent diarrhea. She also reports frequent palpitations and says she has felt shaky and warm during the past 2 weeks. She has lost 9 kg (20 lb) during the past 2 months and was initially excited about the weight loss. Medical history is remarkable for two cesarean deliveries at ages 20 and 22 years. She takes no medications. Family history is remarkable for glaucoma diagnosed in her maternal grandfather at age 65 years. The patient does not smoke cigarettes, drink alcoholic beverages, or use other substances. She is 173 cm (5 ft 8 in) tall and weighs 54 kg (120 lb); BMI is 18 kg/m^2. She appears anxious and is sweating mildly. Temperature is 37.4°C (99.4°F), pulse is 115/min, respirations are 18/min, and blood pressure is 140/90 mm Hg. Physical examination discloses bilateral lid lag and exophthalmos. There is mildly diffuse enlargement of the thyroid with no discrete palpable nodules. Cardiac examination discloses a regular rate and rhythm. There is a fine resting tremor of both hands and hyperreflexia in all extremities. Results of laboratory studies show a serum thyroid-stimulating hormone concentration of 0.2 μU/mL. Without treatment, which of the following is most likely to develop in this patient?

(A) Chronic kidney disease
(B) Congestive heart failure
(C) Fibromyalgia
(D) Glaucoma
(E) Multiple sclerosis

129. A 32-year-old woman, gravida 2, para 2, who is 3 months post partum and breast-feeding, is admitted to the hospital because of a warm, tender, erythematous lump in her right breast that has increased in size during the past week despite applied heat and cephalexin therapy. She has repeatedly pumped milk from that breast but her symptoms have not improved. The patient's pregnancy was complicated by preterm labor, for which she was hospitalized for 1 week prior to giving birth via vaginal delivery at 32 weeks' gestation. Medical history is otherwise unremarkable. She takes a prenatal vitamin and is allergic to sulfa-containing medications. On admission, the patient appears flushed. Temperature is 38.4°C (101.1°F), pulse is 114/min, respirations are 18/min, and blood pressure is 120/76 mm Hg. Physical examination discloses a warm, 10 × 10-cm, erythematous, tender mass in the upper outer quadrant of the right breast. The mass is not fluctuant. Several enlarged, tender lymph nodes are palpable in the right axilla. Ultrasonography-guided aspiration of the mass is scheduled. Which of the following is the most appropriate pharmacotherapy at this time?

(A) Intravenous ampicillin-sulbactam
(B) Intravenous cefazolin
(C) Intravenous piperacillin-tazobactam
(D) Intravenous vancomycin
(E) Oral dicloxacillin

130. A 34-year-old man is referred to the office by his new employer because of a positive PPD skin test. Medical history is remarkable for three episodes of scabies during the past year. He previously harvested asparagus at a local farm but says he recently acquired a position at the local nursing home; part of the required examination was to have the PPD skin test. His test result was positive with 15 mm of induration. Chest x-ray shows no abnormalities. His 32-year-old wife and six children, who range in age from 12 years to 16 months, live in the same house. The patient is started on isoniazid therapy. Which of the following is the most appropriate next step?

(A) Administer PPD skin tests to the whole family
(B) Obtain interferon gamma release assay
(C) Order sputum cultures and gastric washings for the whole family
(D) Schedule another chest x-ray in 3 months
(E) Start the patient's children on isoniazid therapy

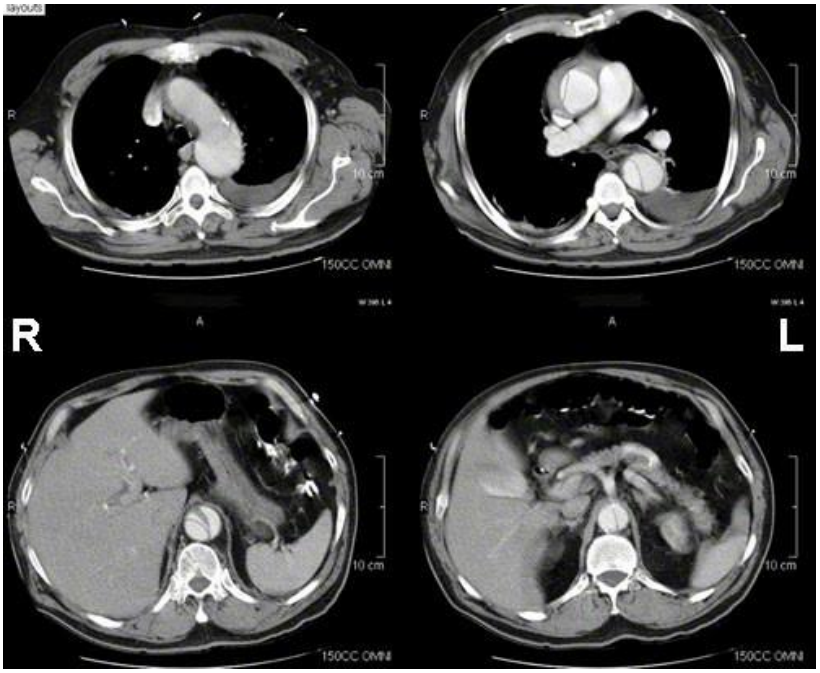

131. **Patient Information**
Age: 62 years
Gender: M, self-identified
Race/Ethnicity: African American, self-identified
Site of Care: emergency department

History
Reason for Visit/Chief Concern: "I passed out suddenly at home."

History of Present Illness:
- collapsed while making breakfast; quickly regained consciousness according to his wife
- does not recall any precipitating factors or symptoms prior to the episode
- the fall was not witnessed, but his wife found him within 1 minute of hearing him fall and called for ambulance
- has chest and back pain that does not radiate
- pain is constant and "crushing"
- pain rated as 10/10
- unable to get comfortable; nothing alleviates the pain

Past Medical History:
- hypertension

Medications:
- amlodipine

Allergies:
- no known drug allergies

Psychosocial History:
- drinks one to two alcoholic beverages daily
- does not smoke cigarettes

This item continues on the following page.

Physical Examination

Temp	Pulse	Resp	BP	O_2 Sat	Ht	Wt	BMI
36.4°C (97.5°F)	104/min	20/min	80/38 mm Hg	94% on RA	183 cm (6 ft)	100 kg (220 lb)	30 kg/m^2

• Appearance: alert; visibly uncomfortable
• HEENT: PERRL; ocular movements intact; oral mucosa pink and moist
• Pulmonary: shallow breath sounds
• Cardiac: tachycardia; diastolic murmur
• Abdominal: nondistended, nontender to palpation
• Extremities: radial pulses 1+; femoral pulses thready bilaterally
• Neurologic: fully oriented; cranial nerves grossly intact; no focal deficits

Diagnostic Studies
• ECG: nonspecific ST-segment and T-wave changes
• CT angiography of the chest: shown

Question: Which of the following is the most appropriate next step in management?

(A) Coronary angiography
(B) CT scan of the head
(C) Norepinephrine infusion
(D) Placement of an intra-aortic balloon pump
(E) Replacement of the aortic root

132. A 63-year-old man is brought to the emergency department by his wife because of a 2-day history of double vision, feeling off-balance, difficulty walking, and leaning to his right side while standing. He also has had sore throat, hoarseness, difficulty swallowing and talking, and fatigue during this time. He had severe stabbing pain on the right side of his face a few minutes before his other symptoms began. The severity of his symptoms has been constant since onset. The patient is right-hand dominant but he has had difficulty accurately using his right arm during this time. When he tries to eat, his right forearm and hand do not accurately guide food to his mouth. He has not had a fever. He has hypertension treated with lisinopril. Temperature is 37.2°C (99.0°F), pulse is 75/min, respirations are 16/min, and blood pressure is 148/98 mm Hg. Oxygen saturation is 98% on room air. On examination, the patient leans to the right while seated. The left pupil measures 6 mm and constricts to 4 mm in response to light; the right pupil measures 4 mm and constricts to 3 mm in response to light. There is no conjunctival icterus. There is horizontal and rotational nystagmus on right gaze. The fast phase of nystagmus is to the left. There is mild right ptosis. The patient can hear finger rubs and whispers bilaterally. The right soft palate does not elevate with phonation; the patient's voice is hoarse, and he has dysarthric speech. Peripheral pulses are 2+ throughout. Muscle strength, bulk, and tone are normal in all extremities. Deep tendon reflexes are 1+ throughout. Babinski sign is absent bilaterally. Sensation to pain and temperature is decreased over the right side of the face, the left extremities, and the left side of the trunk. Finger-nose testing shows dysmetria on the right only. Gait is broad based, and the patient leans to the right while walking. Based on these findings, this patient is most likely to develop which of the following?

(A) Achalasia and gastroparesis
(B) Angle-closure glaucoma
(C) Orthostatic hypertension
(D) Sleep-related hypoventilation
(E) Third-degree atrioventricular block

133. **Patient Information**
Age: 75 years
Gender: F, self-identified
Ethnicity: unspecified
Site of Care: hospital

History
Reason for Visit/Chief Concern: "I've felt tired for a while and I've been bruising easily."

History of Present Illness:
• 3-week history of progressively worsening bruising and fatigue
• gradual onset of symptoms
• no fever, cough, urinary symptoms, chest pain, dyspnea, orthopnea, paroxysmal nocturnal dyspnea, or lower extremity edema
Past Medical History:
• 5-year history of hyperthyroidism
• 10-year history of hyperlipidemia
• 15-year history of coronary artery disease
Medications:
• carvedilol
• lisinopril
• atorvastatin
• methimazole
• daily aspirin
Allergies:
• no known drug allergies
Family History:
• father: deceased at age 56 years from myocardial infarction
Psychosocial History:
• does not smoke cigarettes, drink alcoholic beverages, or use substances or non-prescribed drugs

Physical Examination

Temp	Pulse	Resp	BP	O_2 Sat	Ht	Wt	BMI
37.8°C (100.0°F)	63/min	14/min	135/78 mm Hg	96% on RA	155 cm (5 ft 1 in)	61 kg (135 lb)	26 kg/m^2

• Appearance: in no distress; alert
• HEENT: oropharynx without exudate
• Pulmonary: clear to auscultation
• Cardiac: normal S_1 and S_2 with no murmurs, rubs, or gallops
• Abdominal: bowel sounds normoactive; soft, nontender
• Extremities: no edema or tenderness
• Neurologic: oriented to person, place, and time

This item continues on the following page.

Diagnostic Studies:

Serum		Blood	
Na^+	137 mEq/L	Hematocrit	28%
K^+	4.3 mEq/L	Hemoglobin	9.3 g/dL
Cl^-	110 mEq/L	WBC	$1200/mm^3$
HCO_3^-	22 mEq/L	Platelet count	$43,000/mm^3$
Urea nitrogen	12 mg/dL	Urine	
Creatinine	1.2 mg/dL	Specific gravity	1.010 (N=1.003–1.029)
Albumin	3.6 g/dL	Leukocyte esterase	Negative
		Nitrite	Negative
		WBCs	0/hpf
		RBCs	0/hpf

• ECG: sinus rhythm without ST changes
• chest x-ray: no abnormalities

Question: Which of the following medications is the most likely cause of this patient's symptoms?

(A) Aspirin
(B) Atorvastatin
(C) Carvedilol
(D) Lisinopril
(E) Methimazole

134. A 26-year-old man is brought to the emergency department 30 minutes after his roommate found him confused at home with a bump on the left side of his head and fresh urine on the floor. On arrival, the patient is confused but his mental status returns to normal shortly after arrival. The patient is a medical student, and the last thing he remembers is coming home from class 30 minutes before he was found. He has not had dizziness, blurred vision, fever, or nausea. Medical history is remarkable for bacterial meningitis 5 years ago, an anterior cruciate ligament injury sustained while playing basketball 10 years ago, and unilateral renal agenesis with proteinuria. His only medication is lisinopril. He stayed awake all night twice during the past week to study for final examinations. He works the night shift at a fast-food restaurant on weekends. He has smoked one-half pack of cigarettes daily for 5 years. He drinks four 12-oz beers weekly. He uses cannabis three times weekly. He usually drinks one to two cups of coffee daily but has been drinking 48 oz of coffee daily for the past 2 days. BMI is 27 kg/m^2. Temperature is 36.9°C (98.5°F), pulse is 88/min, respirations are 16/min, and blood pressure is 110/70 mm Hg. Oxygen saturation is 96% on room air. Physical examination shows blood in the mouth and bite marks on the left lateral aspect of the tongue. The remainder of the physical examination, including a neurologic examination, discloses no abnormalities. Results of serum laboratory studies are shown:

Urea nitrogen	20 mg/dL
Creatinine	0.8 mg/dL
Na^+	134 mEq/L
K^+	4.8 mEq/L
Cl^-	104 mEq/L
HCO_3^-	22 mEq/L
Glucose	88 mg/dL

Urine toxicology screening results are negative, and blood alcohol concentration is 0 mg/dL. MRI of the brain and EEG disclose no abnormalities. In addition to decreasing his caffeine intake, which of the following recommendations is most likely to prevent recurrent seizures in this patient?

(A) Alcohol avoidance
(B) Discontinuation of lisinopril
(C) Maintenance of adequate sleep
(D) Occupation change
(E) Tobacco cessation

135. A 33-year-old woman is admitted to the hospital because of a 3-day history of fever, shortness of breath, and progressive cough productive of dark yellow sputum. She felt well before the onset of symptoms. Use of cough suppressant and acetaminophen has provided no relief. She has a 5-year history of myasthenia gravis. Her current medications are pyridostigmine and an oral contraceptive. Temperature is 38.9°C (102.0°F), pulse is 104/min, respirations are 16/min, and blood pressure is 138/72 mm Hg. Oxygen saturation is 94% on oxygen at 4 L/min by nasal cannula. She is alert and appears fatigued. She coughs intermittently throughout the examination. Mucous membranes are dry. Pulmonary examination discloses crackles and rhonchi in the left upper lung field. The remainder of the examination shows no abnormalities. X-ray of the chest shows a patchy infiltrate in the left upper lobe. Which of the following is the most appropriate pharmacotherapy for this patient at this time?

(A) Amoxicillin-clavulanic acid
(B) Azithromycin
(C) Cefepime and tobramycin
(D) Ceftriaxone and doxycycline
(E) Levofloxacin
(F) Trimethoprim-sulfamethoxazole

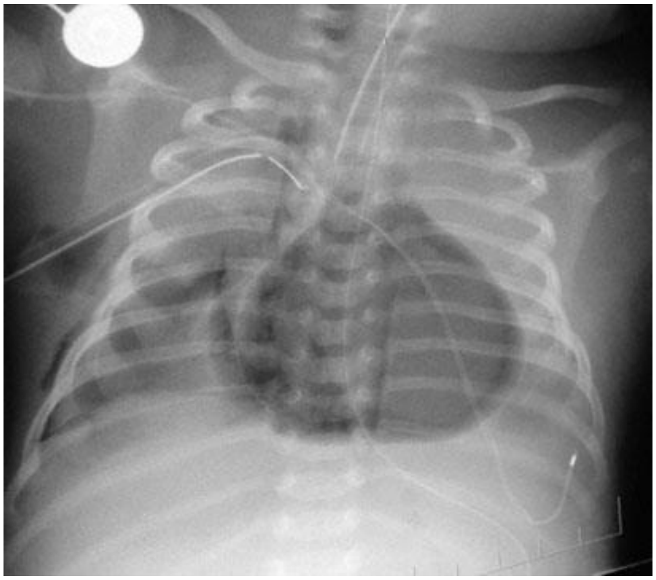

136. A 2-week-old male newborn, who was delivered at 34 weeks' gestation, is being urgently evaluated because the nurses have reported recent decreases in his oxygen saturation and blood pressure. The newborn was diagnosed with respiratory distress syndrome shortly after his birth and he has required continued adjustments to his mechanical ventilation settings, which now show a positive end-expiratory pressure (PEEP) of 10 cm H_2O, a ventilator rate of 50/min, a peak inspiratory pressure of 32 cm H_2O, and an FIO_2 of 0.80. The patient weighs 1600 g (3 lb 9 oz). Now temperature is 36.8°C (98.2°F), pulse is 200/min, respirations are 50/min, and palpable blood pressure is 22 mm Hg. Oxygen saturation is 78%. Physical examination shows cyanosis and poor perfusion to the extremities. Breath sounds are present bilaterally. A fluid bolus is initiated intravenously. Chest x-ray ordered 15 minutes ago is obtained and is shown. Which of the following is the most appropriate management of this patient's condition?

(A) Insertion of a central venous catheter
(B) Median sternotomy
(C) PEEP reduction to 6 cm H_2O
(D) Pericardiocentesis
(E) Placement of a left chest tube

137. A 14-year-old boy is brought to the office by his parents because of a 3-month history of fatigue, recurrent abdominal pain, and 7-kg (15-lb) weight loss. He rates the abdominal pain at its worst as a 6 on a 10-point scale and notes that the pain is associated with loose bowel movements. His stool has not contained mucus or blood. The symptoms have caused him to miss approximately 20 days of school during the past semester. In addition, the patient has developed two to three recurrent mouth ulcers. He has not had any rashes, chest pain, difficulty breathing or swallowing, or joint symptoms, though his heels ache slightly when he walks. Medical history is unremarkable and he takes no medications. He is 150 cm (4 ft 11 in; 5th percentile) tall and weighs 36 kg (80 lb; 3rd percentile). Vital signs are normal. Auscultation of the heart discloses an S_1 and normally split S_2, and no murmur. Abdominal examination discloses mild distention, diffuse tenderness to palpation, and mild fullness in the right lower quadrant. Nail beds appear normal. There is no peripheral edema or arthritis. Neurological examination shows no focal findings. Which of the following is the most likely complication of this patient's condition?

(A) Anal fistula
(B) Colonic carcinoma
(C) Diabetes mellitus
(D) Progressive pulmonary failure
(E) Toxic megacolon

NOTE: THIS IS THE END OF BLOCK 4.
ANY REMAINING TIME MAY BE USED TO CHECK ITEMS IN THIS BLOCK.

Answer Key for USMLE Step 3 Sample Questions

Block 1: FIP

1. B
2. C
3. A
4. C
5. C
6. D
7. A
8. A
9. C
10. B
11. C
12. D
13. D
14. D
15. D
16. B
17. B
18. D
19. B
20. C
21. C
22. B
23. B
24. A
25. E
26. C
27. B
28. C
29. D
30. E
31. B
32. A
33. F
34. B
35. A
36. A
37. B
38. C

Block 2: FIP

39. E
40. D
41. D
42. D
43. D
44. B
45. C
46. E
47. A
48. B
49. D
50. A
51. E
52. B
53. D
54. C
55. E
56. E
57. B
58. B
59. A
60. B
61. E
62. A
63. E
64. C
65. B
66. B
67. B
68. D
69. C
70. F
71. C
72. A
73. D
74. B
75. E
76. D
77. C

Block 3: ACM

78. A
79. C
80. E
81. C
82. A
83. C
84. A
85. A
86. D
87. C
88. D
89. B
90. B
91. E
92. F
93. D
94. E
95. A
96. B
97. D
98. A
99. A
100. B
101. D
102. C
103. E
104. C
105. E
106. B
107. B

Block 4: ACM

108. C
109. A
110. A
111. C
112. B
113. C
114. C
115. C
116. A
117. D
118. B
119. B
120. F
121. A
122. B
123. B
124. B
125. E
126. A
127. C
128. B
129. D
130. A
131. E
132. D
133. E
134. C
135. D
136. D
137. A
